# Gene Expression Differences in Peripheral Blood of Parkinson’s Disease Patients with Distinct Progression Profiles

**DOI:** 10.1371/journal.pone.0157852

**Published:** 2016-06-20

**Authors:** Raquel Pinho, Leonor C. Guedes, Lilach Soreq, Patrícia P. Lobo, Tiago Mestre, Miguel Coelho, Mário M. Rosa, Nilza Gonçalves, Pauline Wales, Tiago Mendes, Ellen Gerhardt, Christiane Fahlbusch, Vincenzo Bonifati, Michael Bonin, Gabriel Miltenberger-Miltényi, Fran Borovecki, Hermona Soreq, Joaquim J. Ferreira, Tiago F. Outeiro

**Affiliations:** 1 Department of NeuroDegeneration and Restorative Research, Center for Nanoscale Microscopy and Molecular Physiology of the Brain, University Medical Center Göttingen, Göttingen, Niedersachsen, Germany; 2 Faculty of Medicine, University of Porto, Porto, Portugal; 3 Clinical Pharmacology Unit, Instituto de Medicina Molecular, Faculty of Medicine, University of Lisbon, Lisbon, Portugal; 4 Department of Molecular Neuroscience, The Institute of Neurology, University College London, London, United Kingdom; 5 Department Clinical Genetics, Erasmus MC, Rotterdam, South Holland, The Netherlands; 6 Institute of Medical Genetics and Applied Genomics, Eberhard-Karls-University Tübingen, Tübingen, Baden-Württemberg, Germany; 7 Department for Functional Genomics, Center for Translational and Clinical Research, University Hospital Center Zagreb, University of Zagreb School of Medicine, Zagreb, Croatia; 8 The Edmond and Lily Safra Center for Brain Sciences, The Hebrew University of Jerusalem, Jerusalem, Israel; 9 Department of Biological Chemistry, The Life Sciences Institute, Jerusalem, Israel; 10 CEDOC, Faculdade de Ciências Médicas, Universidade Nova de Lisboa, Lisboa, Portugal; National Institutes of Health, UNITED STATES

## Abstract

The prognosis of neurodegenerative disorders is clinically challenging due to the inexistence of established biomarkers for predicting disease progression. Here, we performed an exploratory cross-sectional, case-control study aimed at determining whether gene expression differences in peripheral blood may be used as a signature of Parkinson’s disease (PD) progression, thereby shedding light into potential molecular mechanisms underlying disease development. We compared transcriptional profiles in the blood from 34 PD patients who developed postural instability within ten years with those of 33 patients who did not develop postural instability within this time frame. Our study identified >200 differentially expressed genes between the two groups. The expression of several of the genes identified was previously found deregulated in animal models of PD and in PD patients. Relevant genes were selected for validation by real-time PCR in a subset of patients. The genes validated were linked to nucleic acid metabolism, mitochondria, immune response and intracellular-transport. Interestingly, we also found deregulation of these genes in a dopaminergic cell model of PD, a simple paradigm that can now be used to further dissect the role of these molecular players on dopaminergic cell loss. Altogether, our study provides preliminary evidence that expression changes in specific groups of genes and pathways, detected in peripheral blood samples, may be correlated with differential PD progression. Our exploratory study suggests that peripheral gene expression profiling may prove valuable for assisting in prediction of PD prognosis, and identifies novel culprits possibly involved in dopaminergic cell death. Given the exploratory nature of our study, further investigations using independent, well-characterized cohorts will be essential in order to validate our candidates as predictors of PD prognosis and to definitively confirm the value of gene expression analysis in aiding patient stratification and therapeutic intervention.

## Introduction

Parkinson’s Disease (PD) is a multi-system, disabling, insidiously progressive neurodegenerative condition affecting 1–2% of the population over the age of 60 [1]. Currently, PD is incurable, and diagnosis relies on the clinical examination of the patient, as established diagnosis criteria are based on the identification of parkinsonism, namely bradykinesia in combination with either rest tremor, rigidity, or both [2, 3]. Also, once PD is diagnosed, there are no available non-clinical biomarkers to accurately predict the rate of disease progression and to achieve a prognosis. Clinical progression is heterogenous [4]. Major milestones of disease severity, as postural instability and dementia, when present, are considered to be sensible clinical predictors of disease progression [5], but there are no universally accepted clinical markers to differentiate slower or faster progression profiles before and after these milestones are achieved. Thus, identifying molecular signatures that allow discriminating between different progression rates might significantly assist the therapeutic strategy, and enable improved outcomes in clinical trials [6–8]. Expression studies performed in easily accessible tissue, such as blood, showed that peripheral cells recapitulate transcriptional changes occurring in neurodegenerative brain [9, 10], and are valuable to identify novel mechanisms underlying PD [11, 12]. However, these studies were designed to pinpoint relevant genes and pathways for the diagnosis of PD, and did not search for markers to distinguish between different disease progression rates. Our study aimed at investigating, for the first time, whether gene expression profiling of peripheral blood samples would enable differentiating patients with rapid or slow PD progression. By analysing our findings, together with those reported in previously published studies, we found that altered expression of genes involved in nucleic acids metabolic process, immune response, mitochondria, and intracellular-transport may form a strong signature discriminating slow from rapid PD progression. Furthermore, we confirmed identical expression changes in a neuronal, dopaminergic cell model of PD, suggesting that these peripheral transcriptional alterations may report on relevant molecular pathways involved in degeneration of dopaminergic neurons. Altogether, the findings from our exploratory study open novel perspectives for disease prognosis and for the development of new strategies for individualized intervention in PD, setting now the stage for future studies in other neurodegenerative conditions aimed at identifying molecular signatures of disease progression.

## Materials and Methods

A schematic representation of the overall experimental design of this study is presented in S1 Fig.

### Cohort assembly and clinical characterization of patients

Patients were consecutively recruited from the movement disorders outpatient clinic of the Lisbon University Hospital. The Research Ethics Board of the Lisbon Faculty of Medicine approved the study. All participants gave written informed consent. Neurologists with expertise in movement disorders interviewed and examined all cases. Inclusion criteria were a diagnosis of PD according to the UK Brain Bank criteria for idiopathic PD [13] and the presence of motor symptoms for 10 years or longer. This was selected as a cut-off point to perform a cross-sectional evaluation and separate patients into two distinct groups with “Slow” or “Fast” progression, depending on the absence or presence of postural instability, respectively. Postural instability was chosen to discriminate groups because, as an axial symptom, it evolves more rapidly than other motor features and is associated with poorer quality of life [5]. It is present in around three quarters of patients at 10 years of disease duration [14, 15] and, although heterogeneous in time to axial symptoms, patients with postural instability at 10 years represent a group that arrived faster to a level of higher disease burden as opposed to a group without postural instability at this time. Postural instability was defined by item 3.12 of the MDS-UPDRS, part III. Slow progression if scored 0 (no postural instability) and rapid progression if scored ≥ 1. The selection of this operational criterion was based on the consensual recognition that the onset of balance problems is associated with the risk of falls, which corresponds to a clear disability milestone in PD progression [16]. In addition, it is consensually accepted that the absence of postural instability after 10 or more years of symptom onset corresponds to a more benign disability profile that is associated with a slower progression of clinical factors related with loss of autonomy [15]. Our study design focused on the evaluation of gene expression differences in slow versus rapid progression patients and, therefore, we did not include clinically healthy subjects. A structured interview was used to obtain detailed information on PD history, family history of PD, antiparkinsonian treatment, comorbidities and concomitant medication. PD was assessed using the Movement Disorders Society Unified Parkinson’s Disease Rating Scale (MDS-UPDRS)[17] and the modified Hoehn and Yahr scales (mHY), the Mini-Mental State Examination and the Schwab and England activities of daily living scale (SE). If motor fluctuations were present, patients were assessed during *Best-on* period. Included patients (n = 70) were operationally divided into the two groups: slow (n = 35) or rapid (n = 35) progression according to their postural stability. Descriptive analysis was performed using means ± SD values for continuous variables, and absolute and relative frequencies for categorical variables. Statistical comparisons were performed using T-test for independent samples for continuous variables and Chi-square test for categorical variables, using R (version 3.0.3) and statistical significance set to p<0.05.

### Blood sample collection

Venous blood from 70 PD patients was collected in PAXgene tubes (Qiagen) and stored at -80°C prior to RNA extraction. Bias was minimized by collecting samples from both groups of patients in parallel until all 70 samples were collected. All samples were then processed in parallel by a technician blinded to diagnosis.

### Genetic Screening

Genomic DNA was extracted from peripheral blood using standard procedures. We screened for mutations in the SNCA, PARK2 and LRRK2 genes. Analyses were carried out on each gene via PCR using flanking intronic primers for all exons, followed by direct Sanger sequencing of the PCR products in a ABI Prism 3100 (Applied Biosystems), using a BigDye v3.1 sequence kit (Applied Biosystems).

### RNA extraction and assessment of RNA quality

RNA isolation and purification was performed using PAXgene Blood RNA Kit (Qiagen), according to the manufacturer’s instructions. RNA quality was monitored using a Bioanalyzer (Agilent Tecnologies) and determined based on the RNA Integrity Number (RIN) package.

### Microarray analyses

Gene expression profiling was performed using RNA expression data generated by genome array plates (Affymetrix Human Genome U219 platform, HG-U219) on total RNA extracted from whole blood samples of the patients. The microarray data set has been submitted to the GEO database (accession number GSE80599). The expression data was summarized and normalized using Probe Logarithmic Intensity ERror estimation (PLIER) method through Expression Console software (Affymetrix Inc) on all the platform Perfect Match (PM) probes, composed of 49,386 probe sets. Quality control parameters of the microarrays included 3’ to 5’ ratio, labeling matrices, PM mean and polyA spike mean and probe cell intensity (S2 Fig). Principle component analysis (PCA) identified two slow progression outlier samples, which were removed from downstream analyses. In terms of gene pre-filtering, the top 15% non-variant genes were removed prior to the analysis (leaving 17,011 of the 20,014 interrogated genes for analysis). The first method for the evaluation of differential expression included an F test (using the Bioconductor R package affylmGUI, R version 2.13.1)[18]. Additionally, we removed covariate effects including hybridization batch, age and gender by a linear empirical Bayes model [19] (S3 Fig). The expression data was analysed for differential expression using Analysis of Variance (ANOVA), using Parket Genomics Suit^TM^, as well as a t-test including bootstrapping for cross-validation using 1000 random iterations (p<0.001 considered as significant). The detected genes were further validated by permutation-based test. Bootstrapping and permutation t-tests were performed using Matlab^R^ (R2011) software.

### Pathway enrichment analysis

The functional enrichment analysis was conducted on genes detected as differentially expressed, using DAVID resource EASE tool [20]. The enrichment was calculated for GO terms and UP-SEQ features, SP-PIR keywords and KEGG pathways. In addition, GO network analysis was conducted on the GO Biological Processes, through the Cytoscape [21] (version 3.1.0) plugin ClueGO [22], using Kappa Score.

### Cell Culture

Lund Human Mesencephalic (LUHMES) cells were obtained, maintained and differentiated as previously described[23]. Briefly, proliferating LUHMES cells were cultured in Advanced Dulbecco’s modified Eagle’s medium/F12 (DMEM/F12, Gibco) supplemented with 1xN2 (Gibco), 2 mM L-glutamine (Gibco) and 40 ng/mL recombinant basic fibroblast growth factor (R&D Systems). For differentiation, proliferation medium was replaced by DMEM/F12 containing 1xN2, 2 mM L-glutamine, 1 mM dibutyryl cAMP (Sigma Aldrich), 1 μg/mL tetracyclin (Sigma-Aldrich) and 2 ng/mL recombinant human GDNF (R&D Systems). Two days (D2) after adding differentiation medium, cells were seeded in plates (Nunclon) pre-coated with 50 μg/mL poly-L-ornithine and 1 μg/mL fibronectin (Sigma-Aldrich), and grown at 37°C in humidified 5% CO_2_ atmosphere.

### 1-methyl-4-phenylpyridinium (MPP^+^) treatment and toxicity measurement

On differentiation day 5 (D5) cells were treated with 2.5 μM MPP^+^ (Sigma-Aldrich) or control dimethyl sulfoxide (DMSO, Roth). 72h later supernatants were collected and cell viability was assessed by quantitatively measuring adenylate kinase content, using ToxiLight bioassay kit (Lonza) according to the manufacture recommendations.

### RNA isolation and qPCR analysis

At day 8 (D8) of differentiation, total RNA extraction was performed using the RNeasy mini kit (Qiagen). Total RNA was reverse-transcribed into cDNA using QuantiTect Reverse Transcription Kit (Qiagen). For genes of interest, custom primers were designed using Primer 3 [24] (S1 Table). Human acidic ribosomal phosphoprotein P0 gene (RPLP0) was used as RNA loading control, which exhibited consistent expression across all samples and was shown to be the most suitable house keeping gene in peripheral blood mononuclear cells [25]. Equal amplification efficiencies were confirmed for target and reference genes as appropriate and dissociation curves were verified to ensure specific product generation. Samples were loaded in triplicate and no-template controls were run on every reaction plate to exclude for genomic DNA contamination. Amplification conditions were as follows: 5 minutes at 95°C, 40 cycles of 30 seconds at 95°C and 1.5 minutes at 60°C. Real-time PCRs (qPCRs) were performed using Mx300P cycler (Agilent Tecnology) and MESA Blue qPCR MasterMix Plus for SYBR® Assay (Eurogentec). Ct values were obtained from MxPro software and the comparative Ct method was used for relative quantification of target genes. Gene expression levels were calculated as relative expression compared to RPLP0, by subtracting Ct values from genes of interest to corresponding Ct values of the housekeeping gene (ΔCt). Subsequent ΔΔCt (ΔCt rapid progression-ΔCt slow progression) were calculated to assess fold change in rapid progression patients, using the formula 2^-ΔΔCt^. Statistical analysis was performed using GraphPad Prism version 5.00. T-test for independent samples was used with significance at p≤0.05. Data is shown as means ± SD of, at least, three independent experiments.

### Immunocytochemistry analysis

At D8 of overall differentiation, cells were fixed with 4% paraformaldehyde for 10 minutes at room temperature (RT). Following washing with PBS, cells were permeabilized in 0.5% Triton-X-100, for 15 minutes, and blocked with 1.5% normal goat serum in PBS (NGS), for 1h at RT. Primary antibodies diluted 1:2000 in NGS were incubated overnight, at 4°C, and secondary antibodies were diluted 1:1000 and incubated for 2h at RT. Cell nuclei were stained with Hoechst (Invitrogen), and imaging was performed using fluorescence microscopy Leica DMI 6000B microscope (Leica, Wetzlar). Primary antibodies against tyrosine hydroxylase (TH, polyclonal, rabbit, Millipore Corporation) and TUJ1 (monoclonal, mouse, Covance), and secondary antibodies Alexa-Fluor 488 and 555 (Life Technologies) were used in this study.

## Results

### Clinical characterization of rapid *versus* slow progression PD patients

First, we performed the clinical assessment of the 70 patients included in the study (35 slow and 35 rapid progression patients). No significant differences concerning gender, age at motor symptoms onset, age at examination, motor symptoms duration and family history between the two groups existed (Table 1). As expected by the inclusion criteria and cohort stratification, both disease staging (mHY scale) and postural stability (MDS-UPDRS 3.12) were significantly different between the two groups (p<0.001). There was also a statistical significant difference between the two groups on the total motor score (MDS-UPDRS III), on Gait (MDS-UPDRS 3.10) (p<0.001), on the Mini-Mental State Examination (MMSE) (p = 0.0030) and on the Schwab and England activities of daily living scale (p<0.001). The fact that the two groups have similar age of onset and duration of symptoms supports the pragmatic use of these criteria to assume different rate of clinical progression in a cross-sectional study.

**Table 1:** Clinical characterization of the cohort.

	Rapid progression		Slow progression		*p-value*
n	35		35		
Gender (% male)	45.7		51.4		0.811
	Mean	SD	Mean	SD	
**Age at motor symptoms onset**	51.2	12.2	50.6	11.9	0.8304
**Age at examination**	70.0	8.4	66.3	8.7	0.8024
**Motor symptoms duration**	18.8	8.2	15.7	6.7	0.09584
**MDS-UPDRS III**	50.7	16.1	32.2	12.9	<0.001
**Postural stability MDS-UPDRS 3.12**	2.1	1.1	0.0	0.0	<0.001
**Gait MDS-UPDRS 3.10**	1.9	1.1	0.9	0.8	<0.001
**Modified Hoehn and Yahr stage**	3.0	0.8	2.0	0.2	<0.001
**SE Scale**	59.3	20.6	81.8	13.8	<0.001
**MMSE**	24.6	4.6	27.5	2.9	0.00304
Levodopa equivalent dose (mg)*	1050.2	553.3	840.7	444.0	0.08976
**Family History (% yes)**	34.3%		37.1%		

Clinical assessment of the 70 patients included in the study. MDS-UPDRS: Movement Disorder Society- Unified Parkinson’s Disease Rating Scale; HY: Modified Hoehn and Yahr stage; SE: Schwab and England activities of daily living scale; MMSE- Mini mental stage examination

* Levodopa equivalent dose[26].

### Mutations in *SNCA*, *PARK2* and *LRRK2* do not discriminate between rapid and slow PD progression

In order to determine whether known familial PD mutations were associated with the rate of disease progression, we performed genetic screening on the *SNCA*, *PARK2* and *LRRK2* genes, known to cause a substantial fraction of monogenic PD cases [27]. From the 70 patients tested, we identified pathogenic mutations in the *PARK2* gene in 2 cases: 1 of the patients carried the previously described c.155delA, p.Asn52MetfsX29 mutation in heterozygous state[28], whereas the other individual carried the c.1288G>A, p.Gly430Ser alteration in homozygous form (S2 Table). To our knowledge, this particular mutation was not previously reported, and should therefore be investigated in future studies. We found the most common *LRRK2* mutation (c. G6055A, p.Gly2019Ser) in 1 slow and 4 rapid progression PD patients, and some additional polymorphisms in the *PARK2* and *LRRK2* genes. No mutations were detected in the *SNCA* gene.

### Identification of a molecular signature of PD progression in blood

**Fig 1:**
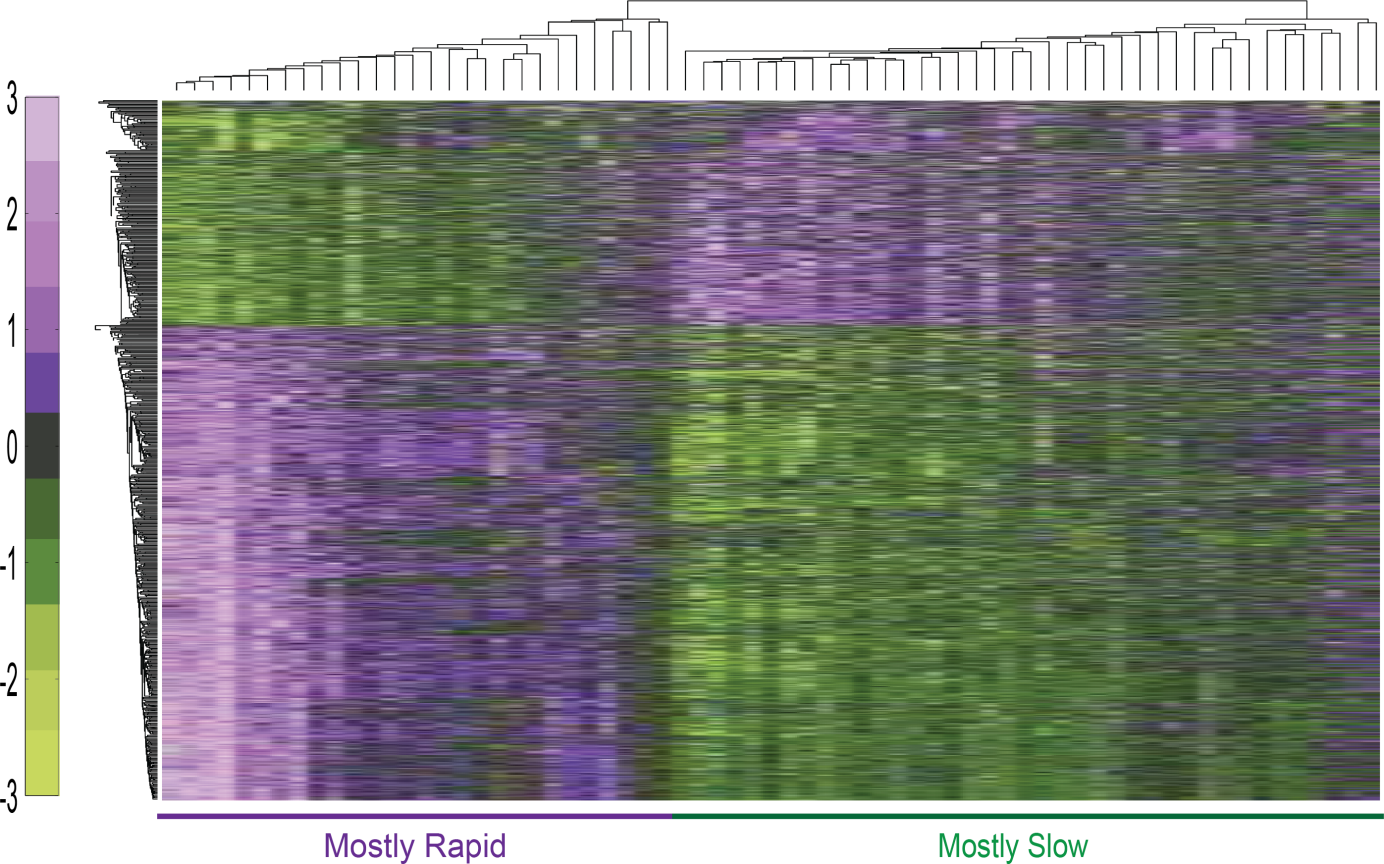
Hierarchical clustering of genes detected as differentially expressed in rapid *versus* slow progression PD patients. Classification based on expression signals of the bootstrapping t-test-detected genes resulted in only five mis-classified slow and 11 mis-classified rapid progression patients. The color bar denotes z-score adjusted expression values, green used for lower expression and purple for higher expression levels. Eucledian distance and average linkage methods were used.

Samples were processed and analysed in parallel to avoid bias induced by sample handling. RNA quality was assessed using RIN in the two groups of patients, which displayed mean RIN values of 7.14 (rapid progressors) and 7.02 (slow progressors). From the 70 RNA samples, 3 had RIN<6 and were not included in subsequent analysis (this corresponded to samples from 1 rapid and 2 slow progression patients). Thus, the expression analysis included 67 array samples. Clinical characterization of these 34 fast and 33 slow PD progression patients is described in S3 Table. Demographic and clinical variables reaching statistically significant differences between rapid vs slow progression groups remained the same, namely the total scores or sub-scores of MDS-UPDRS III, MDS-UPDRS 3.10, MDS- UPDRS 3.12, mHY, SE and MMSE scales. Also, age of onset and duration of symptoms between groups remained similar. All microarray analyses were conducted following extensive quality control including sample and probe filtering. Two outlier samples of two slow progression patients were detected based on PCA analysis and were removed from further analysis. The remaining samples served for all downstream data analysis. 1,251 probe sets were detected using ANOVA at significance level of p≤0.05 (138 at p<0.01), including the *SNCA* gene encoding for alpha-synuclein, a main protein component of Lewy bodies (S4 Table). T-test using bootstrapping was applied for cross-validation, identifying 1,080 differentially expressed genes (p<0.005) (S5 Table). 219 of those genes were also detected by a permutation based t-test, which identified overall 221 genes as altered (p<0.05, 25 at p<0.01), reflecting the robustness of the findings (S6 Table). Hierarchical clustering classification based on their expression signals correctly classified 28/33 of the slow progression PD patients and 23/34 of the rapid progression patients (Fig 1).

**Fig 2:**
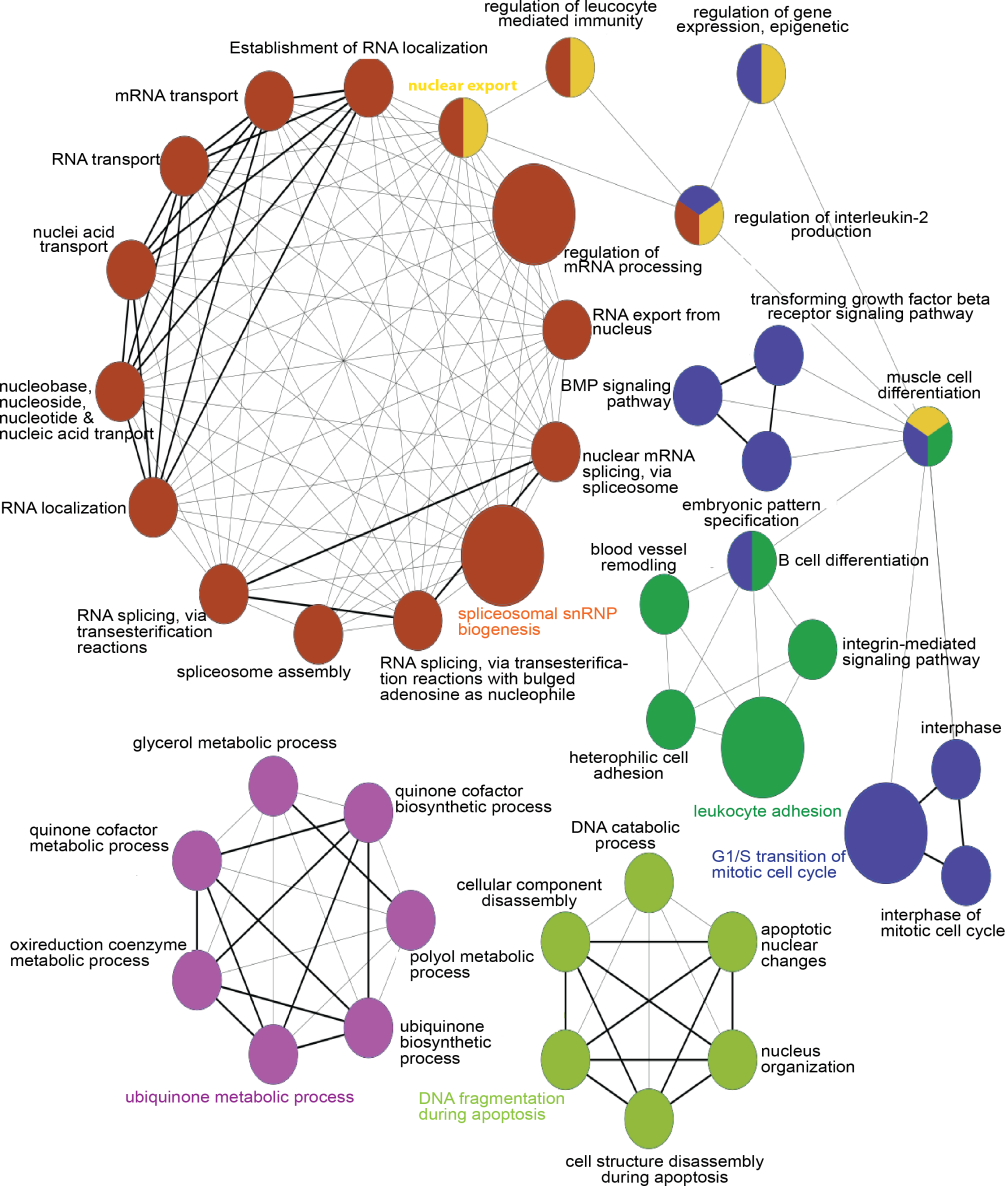
Functional network of the differentially expressed genes altered in PD patients having slow *versus* rapid disease progression. GO network analysis on the GO biological process terms that were found as highly enriched in blood cells from slow *versus* rapid progression patients. The node circle size represents the number of altered genes in the category, and the categories colours correspond to the functional group.

Interestingly, functional enrichment analysis revealed high enrichment of biological terms related to nucleic acid metabolism/repair, intracellular transport, transcription regulation, and immune function (S1 Appendix). Further analysis by the Cytoscape plugin ClueGO, using differential expressed genes on bootstrapping t-test, also yielded high enrichment in nucleic acids metabolic processing gene ontology (GO) terms (Fig 2).

Other studies showed that these molecular processes are affected in PD. Strikingly, microarray analysis of blood from PD patients and healthy controls identified 20 deregulated genes [11], 6 of which were also deregulated in our cohort (BCL11B, PRPF4B, NAP1L1, SERPINB9, LRPPRC, TCEA1). From those, PRPF4B, a pre-mRNA processing factor, NAP1L1, a nucleosome assembly protein, and TCEA1, a transcription elongation factor, are involved in nucleic acid metabolism. Two of the genes identified were also altered in PD patients upon Deep Brain Stimulation (DBS) [9]. One of the genes, LRRC8C, is a component of the volume-regulated anion channel and was also identified in an exon array study in PD leukocytes [29] and in mouse models of the disease [30]. The other, HNRPDL, belongs to the family of heterogeneous nuclear ribonuclearproteins and is involved in RNA processing. From 10 genes found deregulated in the prefrontal cortex of PD patients, 2 were also differentially deregulated between slow and rapid progression patients [31]. These were RNF138, an E3 ubiquitin protein ligase, and SEC24B, involved in vesicle trafficking. Another important vesicular trafficking gene, VAMP1, was also deregulated in our dataset and in the *susbtantia nigra* of PD patients [32]. A meta-analysis study on gene sets that combined data from several expression studies in peripheral and brain cells, revealed a major involvement of mitochondrial genes and bioenergetics changes in PD pathology [33]. Although PPARGC1A, suggested as a potential therapeutic target in PD, was not detected as differentially expressed in this study, our analysis revealed enrichment in genes involved in mitochondrial processes. Remarkably, we identified 42 genes (S7 Table) that were also deregulated in brain tissue from MPTP-treated mice, an established model of PD [30]. The common genes were mostly involved in nucleic acid processing, immune response, mitochondria, and metal ion transport. Additionally, a microarray study using SH-SY5Y cells treated with MPP^+^ reported strong deregulation genes involved in transcription [34]. Therefore, genes controlling nucleic acid metabolism, immune response and mitochondrial processes may be detrimental in dopaminergic cell death and disease progression.

### Validation of transcriptionally-deregulated genes by qPCR

**Fig 3:**
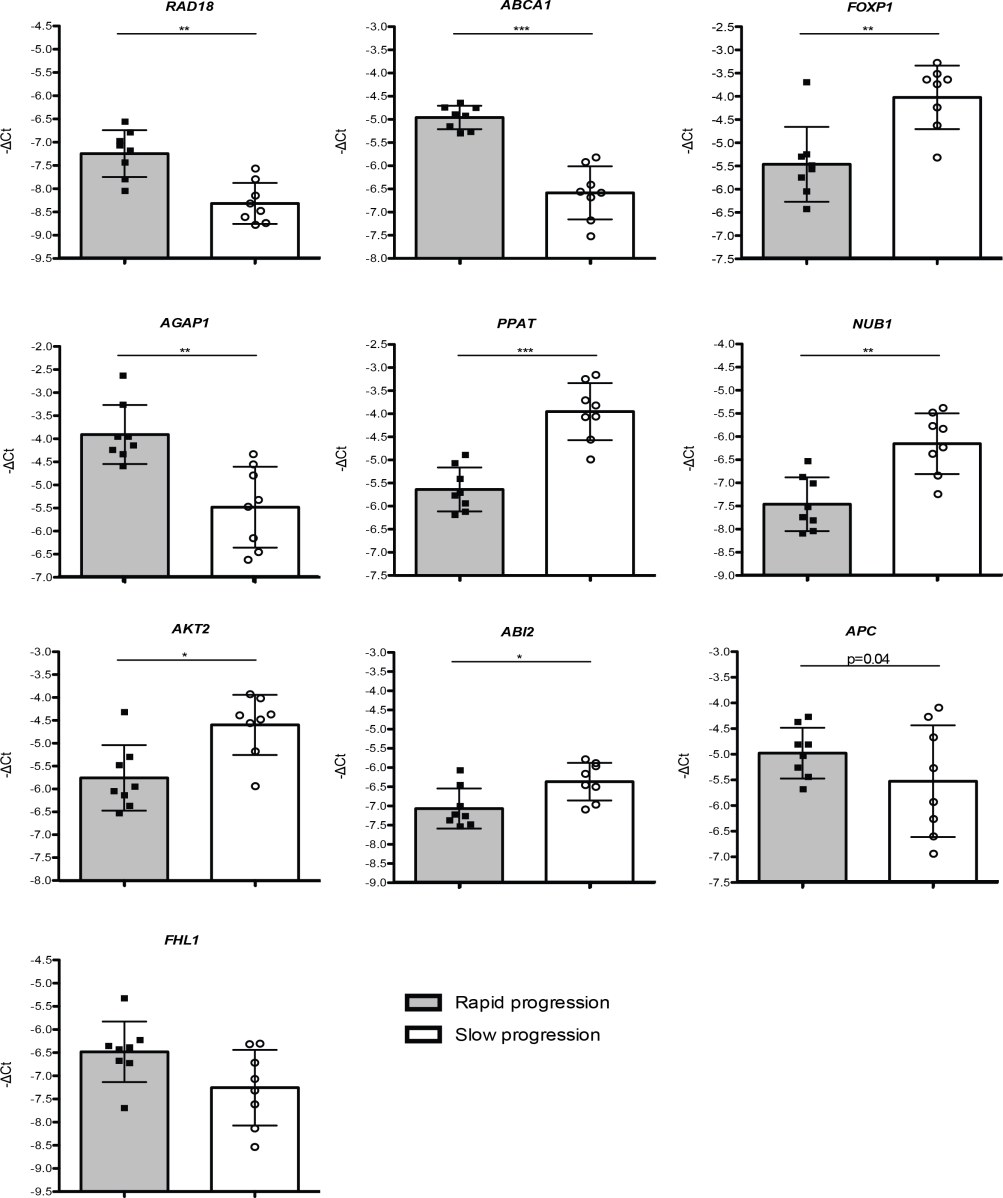
Selected genes investigated by qPCR in PD patients-derived samples. -ΔCts plotted for 10 genes chosen for qPCR validation in cDNA obtained from 16 patients with either slow or rapid progression of the disease. Data is expressed as means ± SD of triplicates. T-test was used for statistical analysis with significance level of p<0.05. *p<0.05; **p<0.01; ***p<0.0005.

Based on their functional relevance and potential involvement in PD progression, we selected 10 genes that showed differential expression between rapid and slow progression patients, and confirmed their pattern of expression using qPCR. Detailed GO analysis for the selected genes is displayed in S8 Table. The validation was conducted on samples from a subset of patients (8 rapid and 8 slow progression patients). The expression changes obtained in qPCR assays were consistent with those observed in the microarray experiments. 8 out of these 10 genes showed significant expression differences, whereas changes in 2 genes did not reach statistical significance (*FHL1*, 1.48 fold-change, p = 0.16; *APC* 1.02 fold-change, p = 0.34). Up-regulated genes included *RAD18* (2.19 fold-change, p = 0.002), *ABCA1* (3.09-fold change, p = 0.0009) and AGAP1 (2.92 fold-change, p = 0.003), while *FOXP1* (0.35 fold-change, p = 0.007), PPAT (0.31 fold-change, p = 0.0003), NUB1 (0.41 fold-change, p = 0.002), AKT2 (0.48 fold-change, p = 0.019 and ABI2 (0.69 fold-change, p = 0.02) were down-regulated (Fig 3).

We further evaluated the expression of 4 of these genes (RAD18, ABCA1, AGAP1 and FOXP1) in an additional set of samples– 5 rapid and 5 slow progressors. Importantly, we confirmed significant differences in the expression of these genes between the two groups of samples (S4 Fig).

### The molecular signature of blood cells from PD patients is reproduced in an acute cell model of PD

**Fig 4:**
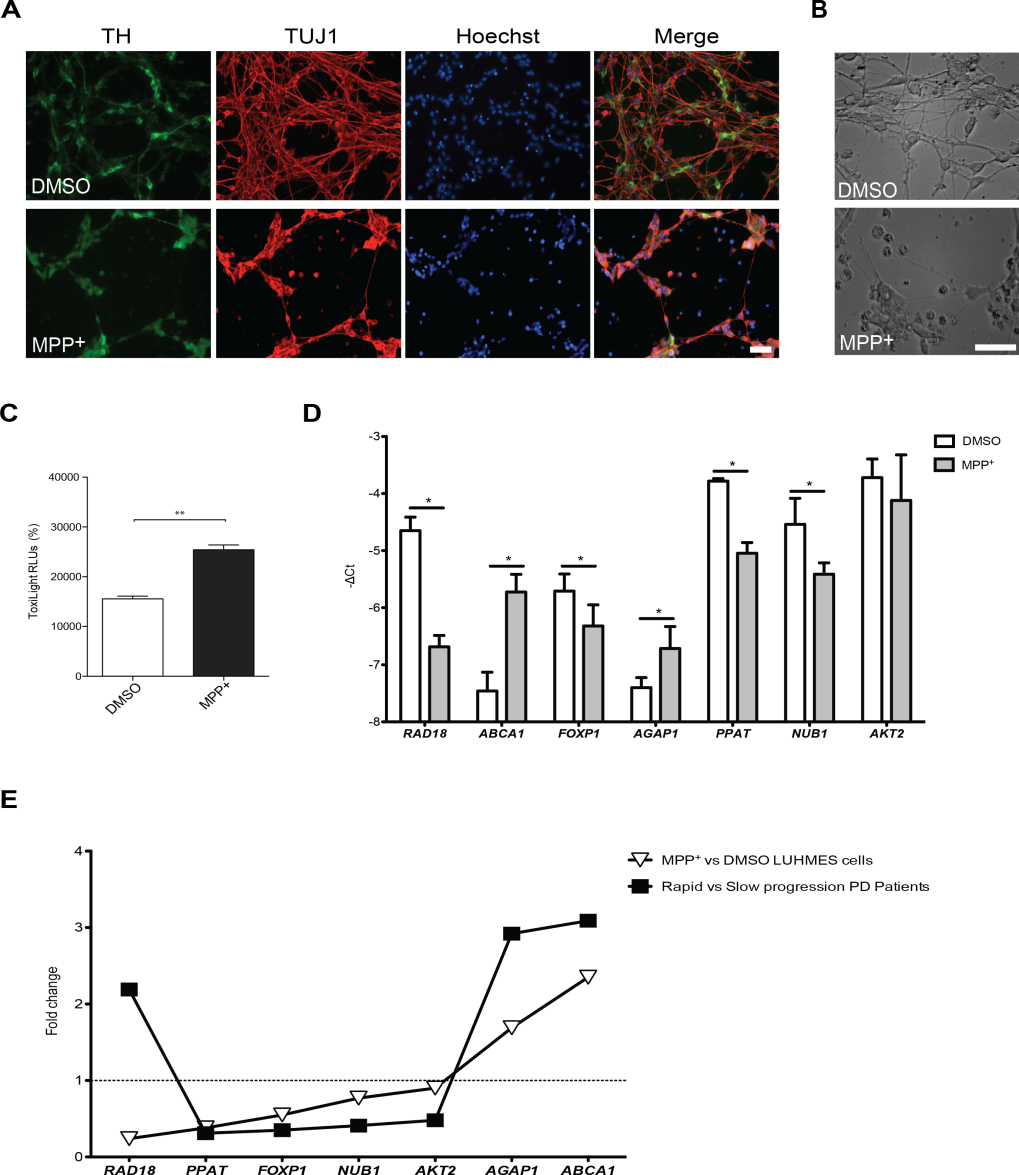
Selected genes investigated in a LUHMES/MPP^+^ cell model of PD. **(A)** LUHMES cells treated with solvent (DMSO) or 2.5μM MPP^+^ stained for Tyrosine Hydroxylase (TH), TUJ1 and nucleus or **(B)** observed in bright field, showed robust loss of neurite integrity upon MPP^+^ treatment. **(C)** Cell viability analysis assessed by ToxiLigh assay revealed a significant increase of adenylate kinase content in the supernatants of MPP^+^ treated cells. **(D)** -Δcts plotted for seven genes chosen for validation using D8 differentiated LUHMES cells exposed to 2.5μm MPP^+^. **(E)** Correlation between fold-change expression values obtained in LUHMES/MPP^+^ model and PD patients. Data is expressed as means ± SD of triplicate samples. T-test was used for statistical analysis with significance level of p<0.05. *p<0.05; **p<0.01. Scale bar 100 μm.

Next, we investigated the expression of the 7 genes exhibiting the largest expression differences in a cell model of PD based on the treatment of differentiated LUHMES cells with a toxin, MPP^+^. These dopaminergic-like cells exhibited high sensitivity to low concentrations of MPP^+^ (Fig 4A, 4B and 4C). qPCR analysis showed that 6 out of these 7 genes were differentially expressed (p<0.05) in cells treated with 2.5 μM MPP^+^. One of the genes did not reach significance (*AKT2*, 0.9 fold-change, p = 0.48). Expression changes of 5 genes were similar to those observed for rapid progression PD patients: *ABCA1* (2.35-fold change), *AGAP1* (1.69 fold-change), *FOXP1* (0.55 fold-change), *PPAT* (0.38-fold change) and *NUB1* (0.77-fold change). Interestingly, *RAD18* was down-regulated in MPP^+^ treated cells (0.24-fold change) and was up-regulated in rapid *versus* progression patients (Fig 4D). Overall, changes in LUHMES cells treated with MPP^+^ were highly consistent with the expression changes observed in PD patients with varying disease progression (Fig 4E).

## Discussion

Here, we report on a novel cross-sectional, exploratory study aiming at determining whether gene expression analysis of RNA samples from easily accessible body fluids (e.g. blood) may allow the identification of molecular signatures that discriminate disease progression in neurodegenerative conditions, such as in PD. Moreover, our translational approach establishes an important bridge between the clinics and the bench, demonstrating that deregulated pathways in patients with varying disease progression are similarly altered in a cell model of PD. In our view, future studies should focus on the detailed investigation of each of the identified genes in order to decipher the individual role of these genes in PD and, in particular, in dopaminergic cell death, and the validity of specific molecular signatures as peripheral predictors of PD progression.

With respect to the clinical criteria, we overcame the limitations of a cross-sectional study design to compare two groups of patients with assumed different rate of disease progression. The disease duration of 10 years as lower limit for recruitment enabled us to generate two groups with clear different disability but not significant difference on disease duration and age of onset. This is consistent with the consideration that the two groups have different rates of progression concerning clinically relevant outcomes, such as postural instability. Also, supporting our strategy of separating rapid *versus* slow progression based in postural instability is the fact that, although no significant differences in age at onset (rapid 51.6±12.1yr *versus* slow 51.5±11.6yr; p = 0.956) and in disease duration (rapid 18.3±7.8yr *versus* slow 15.5±6.8yr; p = 0.956) were observed between the two groups, the rapid progression group showed significant statistical differences in other clinical outcomes known to be associated with more advanced disease stages, such as higher score in MDS-UPDRS III, lower score in the SE scale, and lower score in MMSE. This reflects a more impaired motor status, stronger dependency in activities of daily living, and a worse cognitive profile, respectively. Although not statistically significant, mean levodopa equivalent dose was slightly higher in the rapid progression group, in line with the perception of the physicians for the need to treat higher motor impairment. Conversely, it does not allow concluding that the slower group has a better motor status due to medication levels.

To ascertain the molecular determinants of disease progression in PD, we investigated gene expression profiles of the two groups of PD patients. We identified several genes differentially expressed between rapid and slow progression patients. Functional enrichment analysis revealed multiple signalling pathways associated with PD progression. To further confirm if these were relevant pathways, we asked whether genes whose expression was found altered in our study had been previously identified in other studies, with samples from either PD patients or from models of the disease. Among the genes found to be altered, we found that *SNCA* was downregulated in rapid progressors. This data was intriguing since duplications and triplications of *SNCA* are known causes of familial PD. However, it was previously shown that peripheral changes may be considerably paradoxical. Along the same lines, a recent study revealed that, in blood, lower *SNCA* transcript levels are associated with early-stage PD and correlate with cognitive decline [35]. We also identified several differentially expressed genes that were previously found to be altered in blood cells from early PD patients, in leukocytes from advanced PD patients post DBS surgery, and in genetic and toxin-based mouse models of PD [9, 29, 30]. Particularly, common genes were mostly involved in nucleic acid metabolism, immune response and mitochondrial processes. The expression of selected genes, involved in those pathways, was confirmed by qPCR.

Although the current animal models of PD fail to fully recapitulate all aspects of the disease, models based on MPP^+^ intoxication are still valuable tools to assess the effects associated with selective impairment of dopaminergic neurons. MPP^+^ has been shown to cause acute parkinsonism and dopaminergic neurons death in humans [36], and to rapidly induce motor decline, resembling PD symptomology, in primates [37]. As dopaminergic cell loss and motor disease progression may be closely related, we evaluated the expression of deregulated genes in a dopaminergic cell-based model. LUHMES cells treated with MPP^+^ were used as a tractable in vitro PD model, affording a simple system to dissect the contribution of the selected pathways on dopaminergic cell death. In particular, our approach confirmed that genes controlling nucleic acid metabolism and DNA repair, mitochondria, immune response and intracellular-transport may be detrimental in dopaminergic cell viability.

Surprisingly, we found that while *RAD18* was up-regulated in rapidly-progressing PD patients, it was down-regulated in LUHMES cells treated with MPP^+^. *RAD18* is a highly conserved E3 ubiquitin-ligase that integrates a multi-protein complex responsible for post replication repair of multiple DNA lesions [38, 39]. Thus, the up-regulation of *RAD18* in blood cells of rapid progressing patients might reflect a response to cellular stress. Our findings suggest that the types of stress tested in LUHMES cells may induce different responses from those sensed in peripheral cells. Consistently, in a previous study using primary cortical neurons exposed to Aβ 1–40, a natural antisense transcript against *RAD18* (*NAT-RAD18*) was found to be up-regulated, whereas *RAD18* was down-regulated[40]. Notably, besides *RAD18*, we found several other genes involved in DNA repair, such as *RAD51*, *TOP1*, *TP53*, *POL2A* and multiple members of the POL family. Our study suggests that alterations in nucleic acid metabolism, particularly in DNA repair, may constitute a key event in PD. Thus, although there is growing evidence implicating DNA damage in neurodegenerative processes [41–44], we uncovered, for the first time, a role for DNA repair systems in PD progression.

Another relevant finding was that the forkhead box protein P1 (*FOXP1*), a transcription factor implicated in diverse roles in the immune system in peripheral and brain tissues[45, 46], was significantly down-regulated both in rapid progression patients as well as in LUHMES cells treated with MPP^+^. Decreased *FOXP1* expression may result in increased levels of pro-inflammatory mediators, including damaging cytokines and chemokines [47].

Several ATP-binding cassette (ABC) transporters, primarily known for their role in intracellular transport but also involved in DNA repair, were found altered in our dataset. *ABCA1*, a cholesterol and phospholipid transporter, was significantly up-regulated both in patients with rapid progression and in MPP^+^-treated LUHMES cells. Since up-regulation of *ABCA1* may lead to alter high-density lipoprotein biogenesis[48, 49] in blood, future studies should assess cholesterol levels in PD patients to determine whether these are related to differential disease progression.

Glutamine phosphoribosylpyrophosphate amidotransferase (*PPAT*) is a key enzyme of the purine biosynthesis and requires the incorporation of an iron-sulphur (Fe-S) cluster for its maturation[50]. Importantly, our finding that *PPAT* is consistently down-regulated both in the blood of rapid progression patients and in LUHMES cells treated with MPP^+^, underscores a pivotal role for DNA synthesis/repair systems, mitochondria and iron homeostasis in PD progression.

Given that Lewy Bodies (LBs) are a major hallmark of PD, it has long been hypothesized that protein quality control pathways might constitute attractive reporters for disease progression and targets for pharmacological intervention. Here, we found that *NUB1* was down-regulated in rapid progression patients and in LUHMES treated with MPP^+^. *NUB1*, a negative regulator of ubiquitin-like proteins, was shown to enhance proteasomal degradation of the aSyn-interacting protein synphilin-1[51], to enhance tau phosphorylation and aggregation[52], and to co-localize with presynaptic PK-resistant aSyn in the brains of patients with dementia with LB [53]. Thus, lower *NUB1* expression might impair protein degradation and lead to LB formation.

Also, a member of the ADP-ribosylation factor GTPase-activating protein family, *AGAP1*, was found up-regulated both in rapid progression patients and in LUHMES cells treated with MPP^+^, suggesting that cytoskeleton dynamics, and membrane trafficking may be important processes in PD. More importantly, *AGAP1* was recently shown to mediate the binding of the adapter protein-3 to the muscarin receptor 5 (M5), which are involved in dopamine neurotransmission in the striatum[54].

The strong correlation between the gene expression changes in PD patients and in cell models suggests that peripheral tissues may reflect important disease-related mechanisms taking place in the brain, despite obvious and inherent limitations of dissociated cell models.

To the best of our knowledge, this is the first study aimed at identifying molecular pathways distinguishing PD progression in peripheral blood. In total, we report novel, relevant genes that can be scrutinized in prospective studies, and that may prove valuable for distinguishing rapid *versus* slow PD progression phenotypes. Our findings suggest a possible mechanistic link between altered DNA synthesis/repair systems, intracellular transport, immune response, transcription regulation and the prediction of PD prognosis. Despite certain limitations, such as the need for fully validating the identified gene expression differences in well-characterized, independent cohorts, and that the classification of slow *versus* rapid progression may be a simplified view of the known heterogeneous rates of disease progression, we were able to identify a molecular signature that distinguishes both groups of patients. In future studies, one might perhaps attempt to use other disease progression classifiers. In our study, we used a classification based on previous reports showing that the absence of motor instability after 10 or more years of symptom onset is related to a slower progression phenotype, but it should be clarified that, to the best of our knowledge, there are no standardized clinical scales to unambiguously distinguish rapid from slow progressors thus far. Finally, our findings should be confirmed in longitudinal studies, due to obvious limitations of a cross-sectional study. In such longitudinal studies, it is possible that peripheral predictors of disease progression might be unequivocally identified, which would be an invaluable aid for both clinicians and patients suffering from PD.

Nevertheless, and despite the limitations, our exploratory study may also be relevant for other neurodegenerative disease with varying progression rates (such as Amyotrophic lateral sclerosis), and may open novel avenues for investigating and modelling PD progression in model systems, and the identification of targets for therapeutic intervention.

## Supporting Information

S1 Appendix Functional enrichment analysis conducted through the DAVID resource EASE [56] tool, on differentially expressed genes changed in PD progression. (XLSX)

S1 Fig Schematic representation of the overall experimental design. Gene expression analysis was conducted on RNA samples derived from PD patients and a cellular PD model. **(A)** The experimental design employed to investigate a gene signature of PD progression in patients is depicted on the left side of the schematic. PD patients (n = 70) were clinically characterized by either a slow or rapid disease progression. Gene expression analysis was performed on 67 samples using Affymetrix DNA 3’ U219 microarray plates. Multiple statistical approaches allowed the identification of differentially expressed genes. 10 genes were used for further validation by qPCR. **(B)** From those we selected the 7 most promising genes to further investigate their expression in a cellular model of PD based on MPP^+^ treatment of differentiated LUHMES cells.
(TIF)

S2 Fig Quality control measurements for the microarray data. Plots showing the concentration of the microarray control spike-in probes and the probe cell intensities for all samples (x-axis: sample 1 to 67). **(A)** The log of probe cell intensity after normalization and summary **(B)** A line graph of the PM probes mean with polyA spike RLE mean. **(C)** A line graph of Affymetrix labeling matrices and **(D)** a line graph of Affymetrix 3’ to 5’ ratio values.
(TIF)

S3 Fig Empirical Bayes batch effect parameter estimates using parametric empirical priors. The effects of age and gender covariates were removed (using ComBat software) prior to the differential expression analysis. The plots show the sample quantiles and density **(A)** prior to and **(B)** following the effects removal. The first step is gene-wise standardization of the normalized data (as the magnitude of expression values could differ across genes due to mRNA expression level and probe Sensitivity). This is followed by Empirical Bayes batch effect parameter estimates using parametric empirical priors. Dotted lines on the quantile–quantile plots correspond to the EB-based Normal or Inverse Gamma distributions.
(TIF)

S4 Fig Validation of 4 of the selected genes in additional samples derived from PD patients. **–**ΔCt values plotted for 4 genes (RAD18, ABCA1, FOXP1 and AGAP1) chosen for qPCR validation in 10 additional patients with either slow or rapid progression of the disease. Data is expressed as mean ± SD of triplicates. T-test was used for statistical analysis with significance level of p<0.05. *p<0.05; **p<0.01.
(TIF)

S1 Table Sequences of primers used in the study. Primers used in this study to measure gene expression level by qPCR.
(PDF)

S2 Table Genetic screening of the PD patients included in the study. Mutations in the SNCA, PARK2 and LRRK2 genes were screened in genomic DNA extracted from peripheral blood. Neg.: no mutations found.
(PDF)

S3 Table Clinical assessment of the 67 patients included in clinical and gene expression analysis. MDS-UPDRS: Movement Disorder Society- Unified Parkinson’s Disease Rating Scale; HY: Modified Hoehn and Yahr stage; SE: Schwab and England activities of daily living scale; MMSE- Mini mental stage examination; *Levodopa equivalent dose.
(PDF)

S4 Table Genes detected as differentially expressed in PD patients with rapid compared to slow progression disease, through ANOVA. (PDF)

S5 Table Genes differentially expressed detected by bootstrapping (n = 1000 iterations) t-test having p<0.005. The analysis was conducted on averaged gene expression values based on the summarized and normalized expression data following covariate correction.
(PDF)

S6 Table Genes detected as differentially expressed between slow and rapid progression PD patients identified by t-test with permutations. (PDF)

S7 Table Genes changed in PD progression and in MPTP/SNCA data sets microarray meta analysis[30]. (PDF)

S8 Table GO analysis of selected genes for qPCR validation. Analysis was conducted through DAVID[55].
(PDF)
