# Correction: Gene Expression Differences in Peripheral Blood of Parkinson's Disease Patients with Distinct Progression Profiles

**DOI:** 10.1371/journal.pone.0190552

**Published:** 2017-12-28

**Authors:** Raquel Pinho, Leonor C. Guedes, Lilach Soreq, Patrícia P. Lobo, Tiago Mestre, Miguel Coelho, Mário M. Rosa, Nilza Gonçalves, Pauline Wales, Tiago Mendes, Ellen Gerhardt, Christiane Fahlbusch, Vincenzo Bonifati, Michael Bonin, Gabriel Miltenberger-Miltényi, Fran Borovecki, Hermona Soreq, Joaquim J. Ferreira, Tiago F. Outeiro

The secondary affiliation for the fifth author is not indicated. Tiago Mestre is also affiliated with the Division of Neurology, Department of Medicine, The Ottawa Hospital Research Institute, University of Ottawa Brain and Mind Institute, Canada.
