# Supplementary figures and images for: Gene Expression Differences in Peripheral Blood of Parkinson’s Disease Patients with Distinct Progression Profiles
Source: PLoS One. 2016 Jun 20;11(6):e0157852. doi: 10.1371/journal.pone.0157852 (PMC4913914; doi:10.1371/journal.pone.0157852)

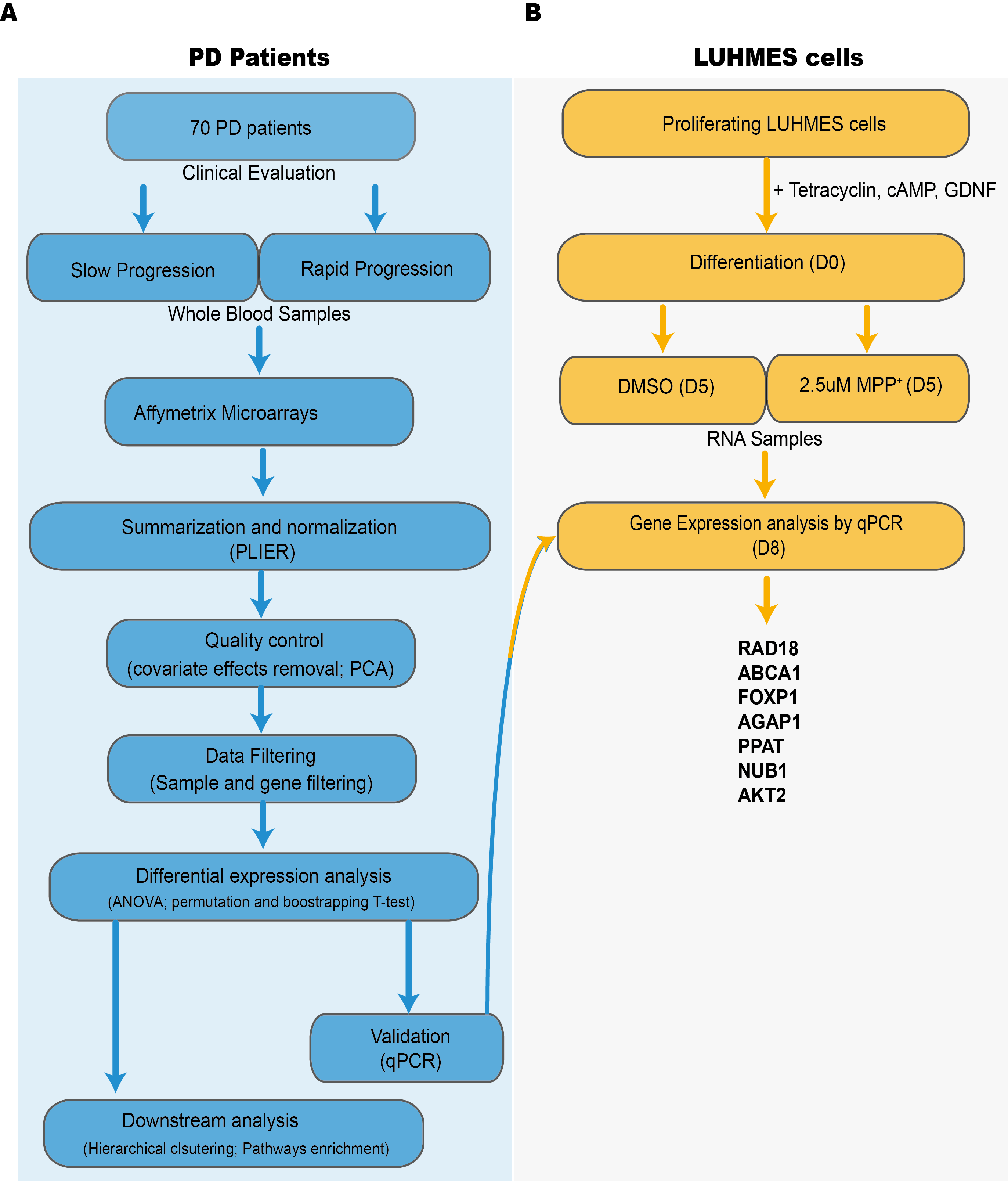

Supplement: S1 Fig — Gene expression analysis was conducted on RNA samples derived from PD patients and a cellular PD model. (A) The experimental design employed to investigate a gene signature of PD progression in patients is depicted on the left side of the schematic. PD patients (n = 70) were clinically characterized by either a slow or rapid disease progression. Gene expression analysis was performed on 67 samples using Affymetrix DNA 3’ U219 microarray plates. Multiple statistical approaches allowed the identification of differentially expressed genes. 10 genes were used for further validation by qPCR. (B) From those we selected the 7 most promising genes to further investigate their expression in a cellular model of PD based on MPP+ treatment of differentiated LUHMES cells. (TIF) [file pone.0157852.s002.tif]

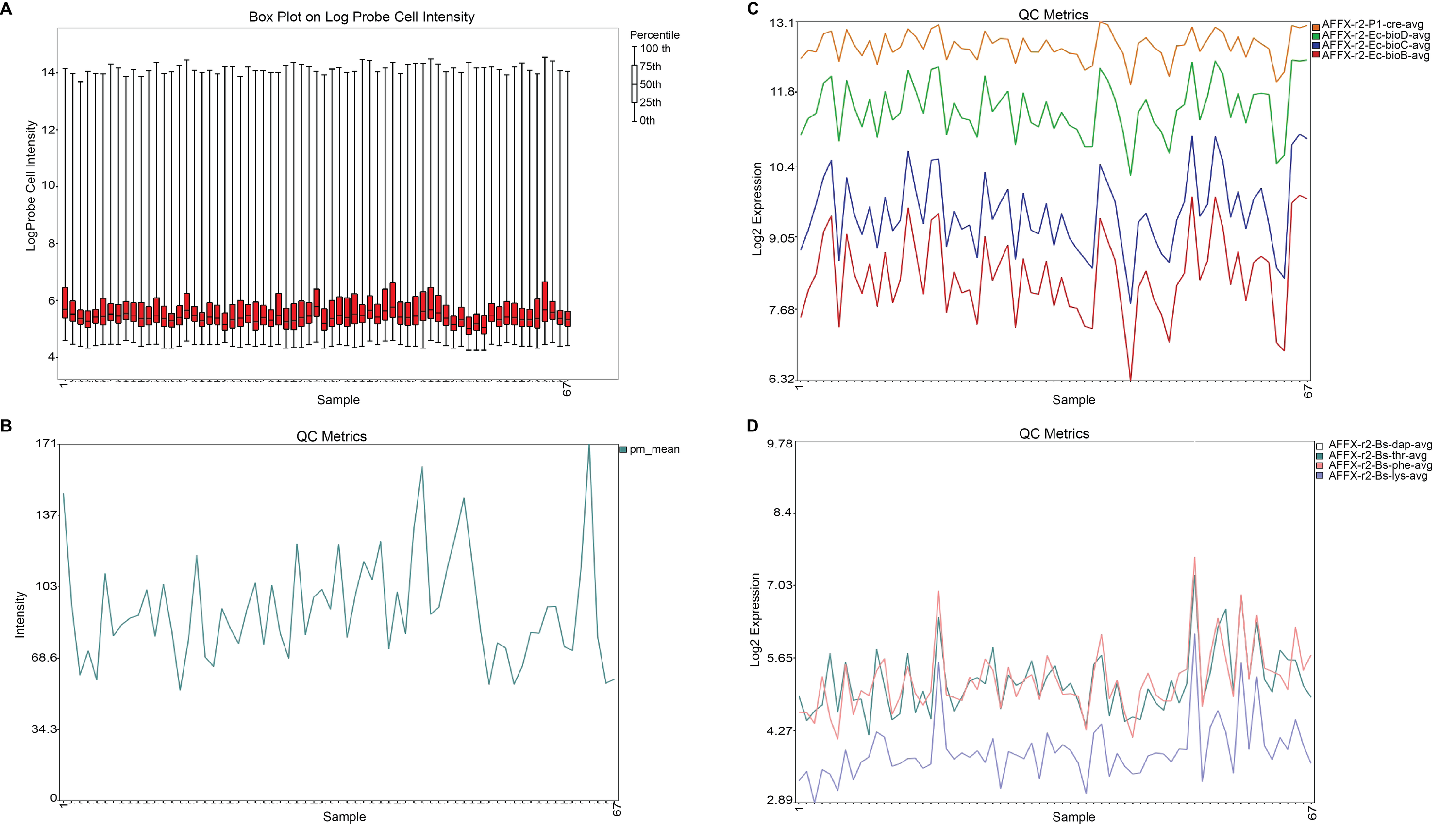

Supplement: S2 Fig — Plots showing the concentration of the microarray control spike-in probes and the probe cell intensities for all samples (x-axis: sample 1 to 67). (A) The log of probe cell intensity after normalization and summary (B) A line graph of the PM probes mean with polyA spike RLE mean. (C) A line graph of Affymetrix labeling matrices and (D) a line graph of Affymetrix 3’ to 5’ ratio values. (TIF) [file pone.0157852.s003.tif]

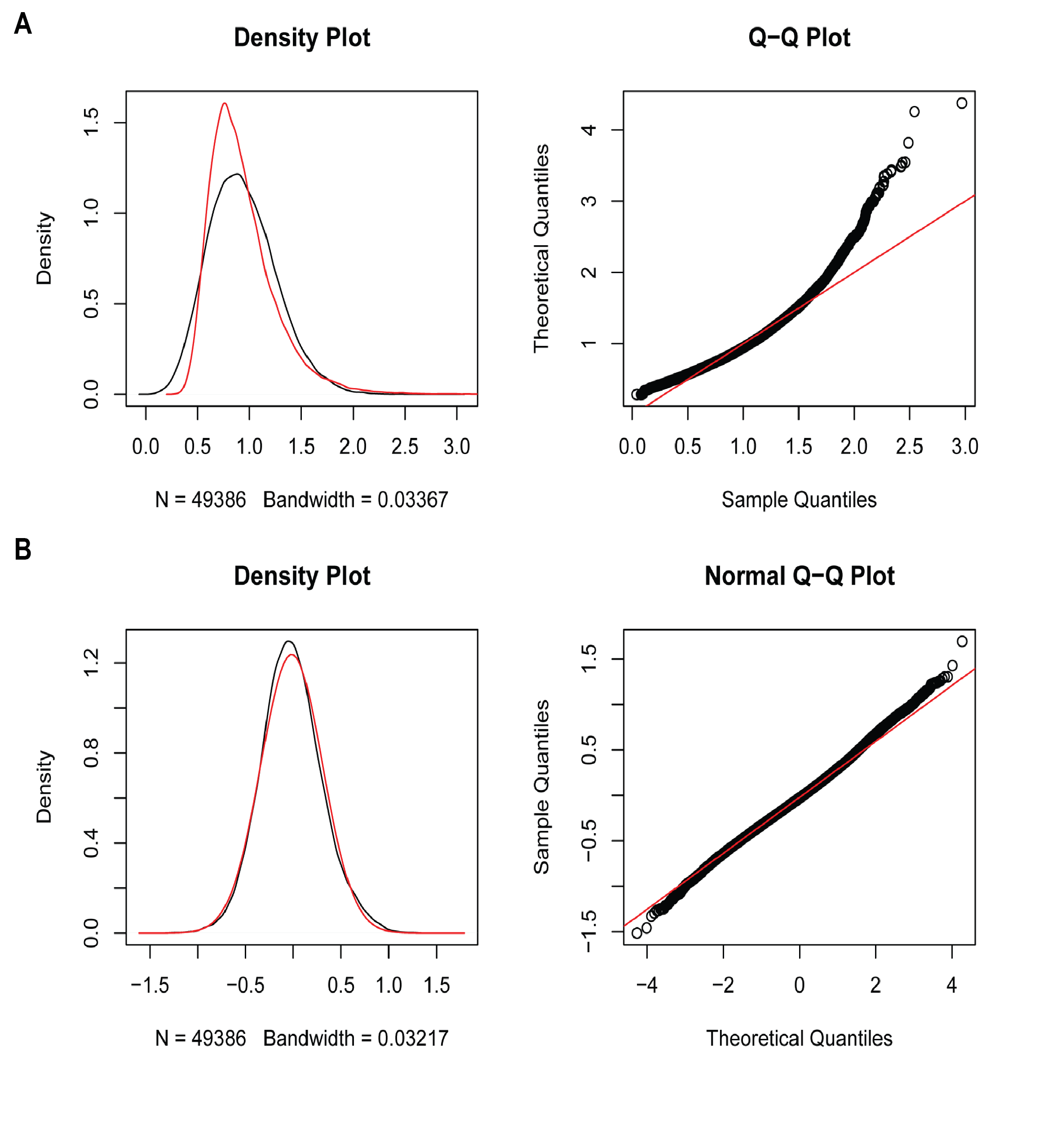

Supplement: S3 Fig — The effects of age and gender covariates were removed (using ComBat software) prior to the differential expression analysis. The plots show the sample quantiles and density (A) prior to and (B) following the effects removal. The first step is gene-wise standardization of the normalized data (as the magnitude of expression values could differ across genes due to mRNA expression level and probe Sensitivity). This is followed by Empirical Bayes batch effect parameter estimates using parametric empirical priors. Dotted lines on the quantile–quantile plots correspond to the EB-based Normal or Inverse Gamma distributions. (TIF) [file pone.0157852.s004.tif]

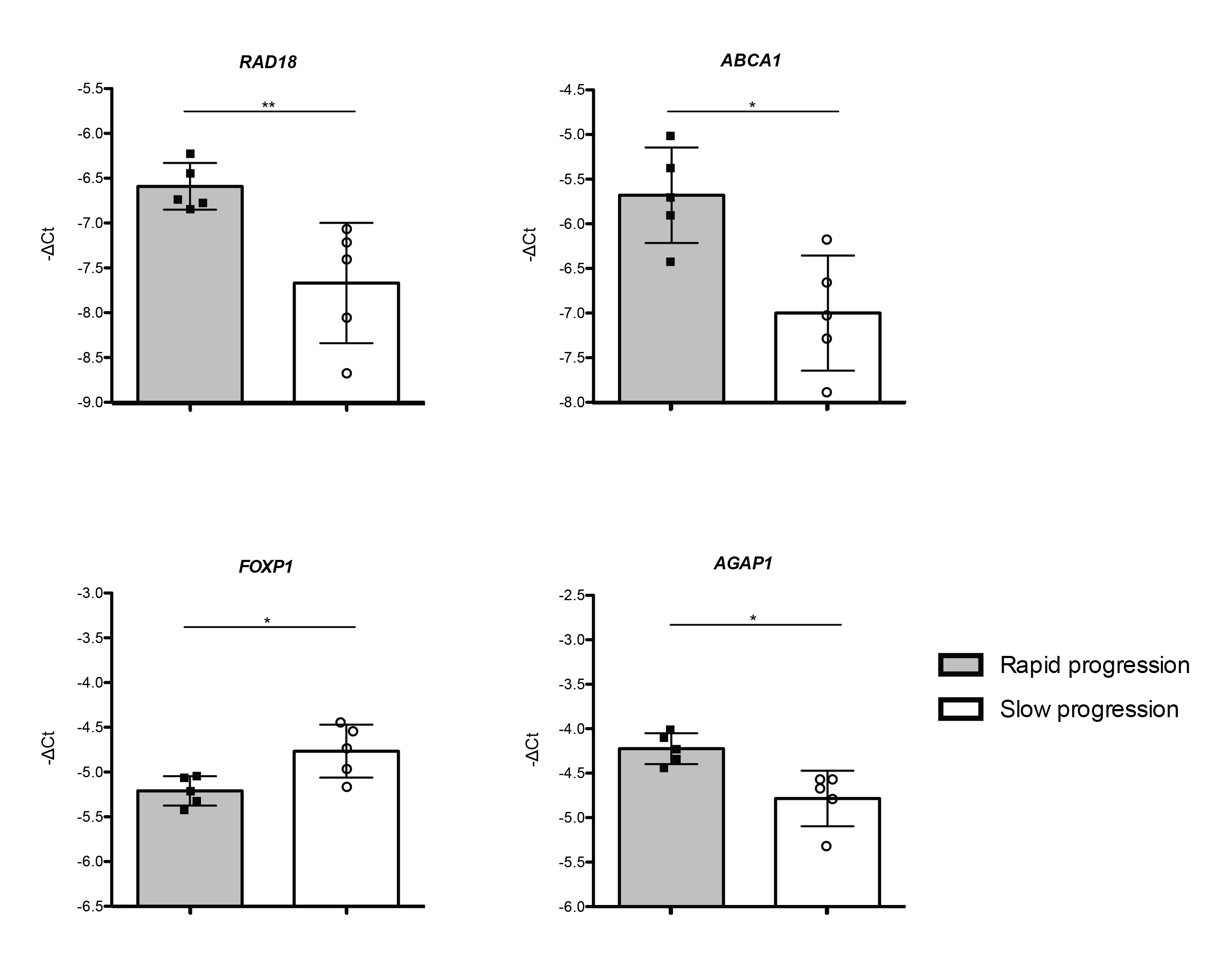

Supplement: S4 Fig — –ΔCt values plotted for 4 genes (RAD18, ABCA1, FOXP1 and AGAP1) chosen for qPCR validation in 10 additional patients with either slow or rapid progression of the disease. Data is expressed as mean ± SD of triplicates. T-test was used for statistical analysis with significance level of p<0.05. *p<0.05; **p<0.01. (TIF) [file pone.0157852.s005.tif]
