# Supplementary material for: Gene Expression Differences in Peripheral Blood of Parkinson’s Disease Patients with Distinct Progression Profiles
Source: PLoS One. 2016 Jun 20;11(6):e0157852. doi: 10.1371/journal.pone.0157852 (PMC4913914; doi:10.1371/journal.pone.0157852)
Supplement: S1 Table — Primers used in this study to measure gene expression level by qPCR. (PDF) [file pone.0157852.s006.pdf]

S1 Table. Sequences of primers used in the study. Primers used in this study to measure gene expression level by qPCR.

Gene Symbol	Primer sequence
RAD18	Forward: GGGAAGCATCACATAAAAACG
	Reverse: GCCCACATTAATTCCTATTACGC
ABCA1	Forward: AGCAAAAAGCGACTCCACAT
	Reverse: TGAGAACTGCAACGTCCACT
FOXP1	Forward: TGCTTTTTGTGCAACTGCTT
	Reverse: GGAGTTTCTCCCTCCCACAT
AGAP1	Forward: TAGCTAATCTGTCCAGGGAGAATAC
	Reverse: CGGCGATGACTAATCACGTT
PPAT	Forward: CATTTCCCAAGTCCATGCTT
	Reverse: CAGGGAAGCCAAGGTCATAA
NUB1	Forward: AATTGACCTTTCCTCCCTGT
	Reverse: TTCCGACTAGCAACATAGCA
AKT2	Forward: CCCCTGACCGCTATGACA
	Reverse: GGACACAAACCAAAAAGGCTAAG
ABI2	Forward: TTGGAAAAGGTTGTGGCAAT
	Reverse: CCAGGAAAAAGCCCAGTCAC
FHL1	Forward: CATCACTGGGTTTGGTAAAGG
	Reverse: GCTCCTGGTGGAAAACAAAG
APC	Forward: ATGAGGACCACAGGCAAATC
	Reverse: TTCCACAAAGTTCCACATGC
