# Supplementary material for: Gene Expression Differences in Peripheral Blood of Parkinson’s Disease Patients with Distinct Progression Profiles
Source: PLoS One. 2016 Jun 20;11(6):e0157852. doi: 10.1371/journal.pone.0157852 (PMC4913914; doi:10.1371/journal.pone.0157852)
Supplement: S2 Table — Mutations in the SNCA, PARK2 and LRRK2 genes were screened in genomic DNA extracted from peripheral blood. Neg.: no mutations found. (PDF) [file pone.0157852.s007.pdf]

S2 Table. Genetic screening of the PD patients included in the study. Mutations in the SNCA, PARK2 and LRRK2 genes were screened in genomic DNA extracted from peripheral blood. Neg.: no mutations found.

ID	SNCA	PARK2	LRRK2
**B0001**	Neg.	Ex 4 c.413-20>C (Homo. rs4709583);<br>Ex 10 c.1138G>C. p.Val380Leu (Het. rs1801582)	Neg.
**B0002**	Neg.	Ex 2 c.171+25T>C (Homo. rs2075923);<br>Ex 4 c.413-20>C (Homo. rs4709583);<br>Ex 10 c.1138G>C. p.Val380Leu (Het. rs1801582)	Neg.
**B0003**	Neg.	Ex 2 c.171+25T>C (Homo. rs2075923);<br>Ex 4 c.413-20>C (Homo. rs4709583)	Neg.
**B0005**	Neg.	Ex 4 c.413-20>C. (Homo. rs4709583)	Neg.
**B0008**	Neg.	Ex 2 c.171+25T>C (Het. rs2075923);<br>Ex 4 c.413-20>C (Homo. rs4709583); c.500G>A.<br>p.Ser167Asn (Het. rs1801474);<br>Ex 10 c.1138G>C. p.Val380Leu (Het. rs1801582)	Neg.
**B0009**	Neg.	Ex 4 c.413-20>C (Homo. rs4709583)	Neg.
**B0010**	Neg.	Ex 2 c.111G>A. p.Pro37Pro (Het. rs77795533);<br>c.171+25T>C (Het. rs2075923);<br>Ex 3 c.172-18T>A. (Het);<br>Ex 4 c.413-20>C (Het. rs4709583)	Neg.
**B0011**	Neg.	Ex 2 c.171+25T>C (Het. rs2075923);<br>Ex 4 c.413-20>C (Het. rs4709583); c.500G>A.<br>p.Ser167Asn (Het. rs1801474)	Neg.
**B0012**	Neg.	Ex 4 c.413-20>C (Homo. rs4709583);<br>Ex 10 c.1138G>C. p.Val380Leu (Het. rs1801582)	Neg.
**B0013**	Neg.	Ex 4 c.413-20>C (Homo. rs4709583);<br>Ex 10 c.1138G>C. p.Val380Leu (Het. rs1801582)	Neg.
**B0015**	Neg.	Ex 4 c.413-20>C (Homo. rs4709583)	Neg.
**B0016**	Neg.	Ex 4 c.413-20>C (Homo. rs4709583)	Neg.
**B0017**	Neg.	Ex 2 c.111G>A. p.Pro37Pro (Het. rs77795533);<br>c.155delA. p.Asn52MetfsX29 (Het);<br>Ex 4 c.413-20>C (Homo. rs4709583);<br>Ex 11 c.1180G>A. p.Asp394Asn (Het. rs1801334)	Neg.
**B0018**	Neg.	Ex 4 c.413-20>C (Het. rs4709583);<br>Ex 7 c.823C>T. p.Arg275Trp (Het. rs34424986)	Neg.
**B0019**	Neg.	Ex 2 c.171+25T>C (Het. rs2075923);<br>Ex 4 c.413-20>C (Homo. rs4709583);<br>Ex 10 c.1138G>C. p.Val380Leu (Het. rs1801582)	Neg.
**B0020**	Neg.	Ex 4 c.413-20>C (Homo. rs4709583)	Neg.
**B0021**	Neg.	Ex 2 c.171+25T>C (Het. rs2075923);<br>Ex 4 c.413-20>C (Homo. rs4709583)	Neg.
**B0022**	Neg.	Ex 2 c.171+25T>C (Het. rs2075923);<br>Ex 4 c.413-20>C (Homo. rs4709583);<br>Ex 10 c.1138G>C. p.Val380Leu (Het. rs1801582)	Neg.
**B0023**	Neg.	Ex 4 c.413-20>C (Homo. rs4709583)	Neg.
**B0024**	Neg.	Ex 4 c.413-20>C (Het. rs4709583)	Neg.
**B0025**	Neg.	Ex 2 c.171+25T>C (Het. rs2075923);<br>Ex 4 c.413-20>C (Homo. rs4709583)	Neg.
**B0026**	Neg.	Ex 2 c.171+25T>C (Het. rs2075923);<br>Ex 4 c.413-20>C (Homo. rs4709583);<br>Ex 10 c.1138G>C. p.Val380Leu (Het. rs1801582)	Neg.

**B0027**	Neg.	Ex 4 c.413-20>C (Het. rs4709583); c.500G>A. p.Ser167Asn (Het. rs1801474);<br>Ex 11 c.1180G>A. p.Asp394Asn (Het. rs1801334)	Neg.
**B0028**	Neg.	Ex 4 c.413-20>C (Homo. rs4709583)	Neg.
**B0031**	Neg.	Ex 2 c.171+25T>C (Het. rs2075923);<br>Ex 4 c.413-20>C (Homo. rs4709583)	c. G6055A. p.Gly2019Ser (Het)
**B0032**	Neg.	Ex 2 c.171+25T>C (Het. rs2075923);<br>Ex 4 c.413-20>C (Homo. rs4709583)	IVS46-49 C>T (Het)
**B0033**	Neg.	Ex 4 c.413-20>C (Homo. rs4709583)	c. G6055A. p.Gly2019Ser (Het)
**B0034**	Neg.	Ex 4 c.413-20>C (Homo. rs4709583)	IVS5 + 33 T>C (Het)
**B0035**	Neg.	Ex 2 c.171+25T>C (Het. rs2075923);<br>Ex 4 c.413-20>C (Homo. rs4709583)	Neg.
**B0036**	Neg.	Ex 2 c.171+25T>C (Homo. rs2075923);<br>Ex 4 c.413-20>C (Homo. rs4709583)	Neg.
**B0037**	Neg.	Ex 4 c.413-20>C (Homo. rs4709583)	c.A3843G. p.Leu1281Leu (Het)
**B0038**	Neg.	Ex 4 c.413-20>C (Homo. rs4709583)	Neg.
**B0039**	Neg.	Ex 2 c.171+25T>C (Het. rs2075923);<br>Ex 3 c.245C>A. p.Ala82Glu (Het. rs55774500);<br>Ex 4 c.413-20>C (Homo. rs4709583);<br>Ex7 c.823C>T. p.Arg275Trp (Het. rs34424986)	Neg.
**B0040**	Neg.	Ex 4 c.413-20>C (Homo. rs4709583);<br>Ex 10 c.1138G>C. p.Val380Leu (Homo. rs1801582)	Neg.
**B0041**	Neg.	Ex 2 c.171+25T>C (Het. rs2075923);<br>Ex3 c.172-18T>A (Het);<br>Ex 4 c.413-20>C (Homo. rs4709583); c.500G>A. p.Ser167Asn (Het. rs1801474);<br>Ex 10 c.1138G>C. p.Val380Leu (Het. rs1801582);<br>Ex11 c.1204C>T. p.Arg402Cys (Het. rs55830907)	Neg.
**B0043**	Neg.	Ex 2 c.171+25T>C (Het. rs2075923);<br>Ex 4 c.413-20>C (Homo. rs4709583)	Neg.
**B0044**	Neg.	Ex 4 c.413-20>C (Homo. rs4709583);<br>Ex 10 c.1138G>C. p.Val380Leu (Het. rs1801582)	Neg.
**B0045**	Neg.	Ex 4 c.413-20>C (Homo. rs4709583); c.500G>A. p.Ser167Asn (Het. rs1801474)	Neg.
**B0046**	Neg.	Ex 4 c.413-20>C (Homo. rs4709583);<br>Ex 10 c.1138G>C. p.Val380Leu (Het. rs1801582)	Neg.
**B0047**	Neg.	Ex 2 c.171+25T>C (Het. rs2075923);<br>Ex 4 c.413-20>C (Homo. rs4709583)	Neg.
**B0048**	Neg.	Ex 4 c.413-20>C (Homo. rs4709583)	Neg.
**B0049**	Neg.	Ex 2 c.171+25T>C (Het. rs2075923);<br>Ex 4 c.413-20>C (Homo. rs4709583);<br>Ex 10 c.1138G>C. p.Val380Leu (Het. rs1801582)	Neg.
**B0050**	Neg.	Ex 2 c.171+25T>C (Het. rs2075923);<br>Ex 4 c.413-20>C (Homo. rs4709583);<br>Ex11 c.1180G>A. p.Asp394Asn (Het. rs1801334)	IVS46-49 C>T (Homo)
**B0051**	Neg.	Ex 2 c.171+25T>C (Het. rs2075923);<br>Ex 4 c.413-20>C (Homo. rs4709583)	Neg.
**B0052**	Neg.	Ex 4 c.413-20>C (Homo. rs4709583)	Neg.
**B0053**	Neg.	Ex 4 c.413-20>C (Homo. rs4709583);<br>Ex 10 c.1138G>C. p.Val380Leu (Het. rs1801582)	Neg.
**B0054**	Neg.	Ex12 c.1288G>A. p.Gly430Ser (Homo)	Neg.
**B0055**	Neg.	Ex 2 c.171+25T>C (Het. rs2075923);<br>Ex 4 c.413-20>C (Homo. rs4709583)	Neg.
**B0056**	Neg.	Ex 2 c.171+25T>C (Het. rs2075923);<br>Ex 4 c.413-20>C (Homo. rs4709583);<br>Ex 10 c.1138G>C. p.Val380Leu (Het. rs1801582)	Neg.
**B0057**	Neg.	Ex 2 c.171+25T>C (Het. rs2075923);<br>Ex 4 c.413-20>C (Homo. rs4709583)	Neg.

**B0058**	Neg.	Ex 4 c.413-20>C (Homo. rs4709583)	c. G6055A.<br>p.Gly2019Ser (Het)
**B0059**	Neg.	Ex 4 c.413-20>C (Homo. rs4709583);<br>Ex 10 c.1138G>C. p.Val380Leu (Het. rs1801582)	Neg.
**B0060**	Neg.	Ex 2 c.171+25T>C (Het. rs2075923);<br>Ex 4 c.413-20>C (Het. rs4709583);<br>Ex 10 c.1138G>C. p.Val380Leu (Het. rs1801582)	Neg.
**B0061**	Neg.	Ex 2 c.171+25T>C (Het. rs2075923);<br>Ex 4 c.413-20>C (Het. rs4709583);<br>Ex 10 c.1138G>C. p.Val380Leu (Het. rs1801582)	Neg.
**B0063**	Neg.	Neg.	Neg.
**B0067**	Neg.	Ex 4 c.413-20>C (Het. rs4709583);<br>Ex 10 c.1138G>C. p.Val380Leu (Homo. rs1801582)	Neg.
**B0069**	Neg.	Ex 4 c.413-20>C (Homo. rs4709583)	Neg.
**B0070**	Neg.	Ex 4 c.413-20>C (Homo. rs4709583);<br>Ex 10 c.1138G>C. p.Val380Leu (Het. rs1801582)	Neg.
**B0083**	Neg.	Ex 4 c.413-20>C (Homo. rs4709583)	Neg.
**B0088**	Neg.	Ex 4 c.413-20>C (Homo. rs4709583);<br>Ex 10 c.1138G>C. p.Val380Leu (Het. rs1801582)	Neg.
**B0089**	Neg.	Ex 2 c.171+25T>C (Het. rs2075923);<br>Ex 4 c.413-20>C (Homo. rs4709583)	c. G6055A.<br>p.Gly2019Ser (Het)
**B0091**	Neg.	Ex 2 c.171+25T>C (Het. rs2075923);<br>Ex 4 c.413-20>C (Homo. rs4709583)	Neg.
**B0092**	Neg.	Ex 4 c.413-20>C (Homo. rs4709583)	Neg.
**B0093**	Neg.	Ex 4 c.413-20>C (Homo. rs4709583);<br>Ex 10 c.1138G>C. p.Val380Leu (Het. rs1801582)	Neg.
**B0094**	Neg.	Ex 4 c.413-20>C (Homo. rs4709583);<br>Ex 10 c.1138G>C. p.Val380Leu (Het. rs1801582)	Neg.
**B0095**	Neg.	Ex 2 c.171+25T>C (Het. rs2075923);<br>Ex 4 c.413-20>C (Homo. rs4709583)	Neg.
**B0096**	Neg.	Ex 2 c.171+25T>C (Het. rs2075923);<br>Ex 4 c.413-20>C (Homo. rs4709583)	c. G6055A.<br>p.Gly2019Ser (Het)
**B0097**	Neg.	Ex 2 c.171+25T>C (Het. rs2075923);<br>Ex 4 c.413-20>C (Homo. rs4709583)	Neg.
**B0101**	Neg.	Ex 4 c.413-20>C (Homo. rs4709583);<br>Ex 10 c.1138G>C. p.Val380Leu (Het. rs1801582)	Neg.
**B0103**	Neg.	Ex 4 c.413-20>C (Homo. rs4709583);<br>Ex 10 c.1138G>C. p.Val380Leu (Homo. rs1801582)	Neg.
