# Supplementary material for: Gene Expression Differences in Peripheral Blood of Parkinson’s Disease Patients with Distinct Progression Profiles
Source: PLoS One. 2016 Jun 20;11(6):e0157852. doi: 10.1371/journal.pone.0157852 (PMC4913914; doi:10.1371/journal.pone.0157852)
Supplement: S3 Table — MDS-UPDRS: Movement Disorder Society- Unified Parkinson’s Disease Rating Scale; HY: Modified Hoehn and Yahr stage; SE: Schwab and England activities of daily living scale; MMSE- Mini mental stage examination; *Levodopa equivalent dose. (PDF) [file pone.0157852.s008.pdf]

S3 Table. Clinical assessment of the 67 patients included in clinical and gene expression analysis. MDS-UPDRS: Movement Disorder Society- Unified Parkinson's Disease Rating Scale; HY: Modified Hoehn and Yahr stage; SE : Schwab and England activities of daily living scale; MMSE- Mini mental stage examination; *Levodopa equivalent dose.

	Rapid progression		Slow progression		*p-value*
**n**	34		33		
**Gender** (% male)	44.1		51.5		0.7178
	**Mean**	**SD**	**Mean**	**SD**	
**Age at motor symptoms onset**	51.6	12.1	51.5	11.6	0.956
**Age at examination**	69.9	8.5	66.9	8.5	0.1592
**Motor symptoms duration**	18.3	7.8	15.5	6.8	0.1214
**MDS-UPDRS III**	51.0	16.3	32.9	13.0	<0.001
**Postural stability MDS-UPDRS 3.12**	2.0	1.1	0.0	0.0	<0.001
**Gait MDS-UPDRS 3.10**	1.9	1.1	1,0	0.8	<0.001
**Modified Hoehn and Yahr stage**	3.0	0.8	2.0	0.2	<0.001
**SE Scale**	59.3	20.9	81.3	14.1	<0.001
**MMSE**	24.5	4.6	27.3	3.0	0.00398
**Levodopa equivalent dose** (mg)*	1045.8	560.8	818.1	446.9	0.07462
**Family History** (% yes)	35.3%		39.4%		
