# Supplementary material for: Gene Expression Differences in Peripheral Blood of Parkinson’s Disease Patients with Distinct Progression Profiles
Source: PLoS One. 2016 Jun 20;11(6):e0157852. doi: 10.1371/journal.pone.0157852 (PMC4913914; doi:10.1371/journal.pone.0157852)
Supplement: S4 Table — (PDF) [file pone.0157852.s009.pdf]

S4 Table: Genes detected as differentially expressed in PD patients having fast compared to slow progressive disease, through Analysis of Variance (ANOVA)

Column #	Probeset ID	Gene Symbol	Gene Title	RefSeq Transcript ID	p-value (Target)	p-value (Scan Date)	p-value (rapid vs Slow)	Ratio (rapid vs Slow)	Fold-Change (rapid vs Slow)	Estimate (rapid vs Slow)	F (rapid vs Slow)	T (rapid vs Slow)	F (Target)	SS (Target)	F (Scan Date)	SS (Scan Date)	SS (Error)
41657	11756747_a_at	RBMS2	RNA binding motif, single stranded interacting protein 2	NM_002898	4,04E-05	0,0216922	4,04E-05	1,257	1,257	rapid up vs Slow	0,329	19,691	4,437	19,691	1,626	3,111	1,027
31752	11746842_x_at	ABI2	abl-interactor 2	NM_005759	0,000108553	0,142251	0,000108553	0,885	-1,130	rapid down vs Slow	-0,176	17,217	-4,149	17,217	0,463	1,793	0,193
17975	11733065_a_at	NFYB	nuclear transcription factor Y, beta	NM_006166	0,000642248	0,228925	0,000642248	0,881	-1,135	rapid down vs Slow	-0,182	12,998	-3,605	12,998	0,497	1,450	0,222
29720	11744810_a_at	ZBTB24	zinc finger and BTB domain containing 24	NM_001164313 /// NM_014797	0,000717219	0,129609	0,000717219	1,112	1,112	rapid up vs Slow	0,153	12,746	3,570	12,746	0,350	1,859	0,204
43788	11758878_x_at	FABP1	fatty acid binding protein 1, liver	NM_001443	0,000721389	0,199266	0,000721389	1,094	1,094	rapid up vs Slow	0,129	12,733	3,568	12,733	0,250	1,551	0,122
13522	11728612_at	ADAMTS6	ADAM metallopeptidase with thrombospondin type 1 motif, 6	NM_197941	0,000826031	0,0319425	0,000826031	1,145	1,145	rapid up vs Slow	0,195	12,425	3,525	12,425	0,571	2,840	0,522
32939	11748029_a_at	TFE3	transcription factor binding to IGHM enhancer 3	NM_006521	0,00110587	0,577154	0,00110587	1,178	1,178	rapid up vs Slow	0,236	11,768	3,430	11,768	0,837	0,727	0,207
35299	11750389_a_at	SLC26A7	solute carrier family 26, member 7	NM_052832 /// NM_134266	0,00116465	0,0599053	0,00116465	1,135	1,135	rapid up vs Slow	0,182	11,653	3,414	11,653	0,497	2,402	0,410
7219	11722309_a_at	TRPM4	transient receptor potential cation channel, subfamily M, member 4	NM_001195227 /// NM_017636	0,00119279	0,0568757	0,00119279	1,139	1,139	rapid up vs Slow	0,187	11,599	3,406	11,599	0,526	2,438	0,442
19171	11734261_at	TRPM5	transient receptor potential cation channel, subfamily M, member 5	NM_014555	0,00138278	0,00683487	0,00138278	1,103	1,103	rapid up vs Slow	0,142	11,271	3,357	11,271	0,301	3,925	0,419
44688	11759778_at	ASXL1	additional sex combs like 1 (Drosophila)	NM_001164603 /// NM_015338	0,0014336	0,603649	0,0014336	0,860	-1,163	rapid down vs Slow	-0,218	11,191	-3,345	11,191	0,713	0,687	0,175
34043	11749133_a_at	NLRX1	NLR family member X1	NM_024618 /// NM_170722	0,00156722	0,159579	0,00156722	1,153	1,153	rapid up vs Slow	0,205	10,994	3,316	10,994	0,631	1,711	0,393
48701	11763791_a_at	IL22RA1	interleukin 22 receptor, alpha 1	NM_021258	0,00161378	0,554203	0,00161378	1,142	1,142	rapid up vs Slow	0,192	10,930	3,306	10,930	0,552	0,762	0,154
41764	11756854_a_at	EHBP1	EH domain binding protein 1	NM_001142614 /// NM_001142615 /// NM_001142616 /// NM_015252	0,00167486	0,880105	0,00167486	1,136	1,136	rapid up vs Slow	0,184	10,848	3,294	10,848	0,505	0,295	0,055
32545	11747635_x_at	PYGB	phosphorylase, glycogen; brain	NM_002862	0,00178161	0,802387	0,00178161	1,131	1,131	rapid up vs Slow	0,177	10,713	3,273	10,713	0,471	0,408	0,072
44050	11759140_at	ITGB6	integrin, beta 6	NM_000888	0,001798	0,428322	0,001798	1,117	1,117	rapid up vs Slow	0,160	10,693	3,270	10,693	0,385	0,975	0,140
24087	11739177_	FAM108B	family with	NM_0010257	0,001831	0,003025	0,00183144	1,142	1,142	rapid up vs	0,191	10,65	3,264	10,65	0,547	4,509	0,926

	at	1	sequence similarity 108, member B1	80 /// NM_016014	44	06				Slow		2		2			
37328	11752418_a_at	AFAP1L1	actin filament associated protein 1-like 1	NM_001146337 /// NM_152406	0,00185666	0,019472	0,00185666	1,122	1,122	rapid up vs Slow	0,166	10,622	3,259	10,622	0,412	3,186	0,494
5602	11720692_at	DIRAS1	DIRAS family, GTP-binding RAS-like 1	NM_145173	0,00198289	0,413707	0,00198289	1,124	1,124	rapid up vs Slow	0,169	10,479	3,237	10,479	0,426	1,002	0,163
41915	11757005_a_at	FUCA1	fucosidase, alpha-L- 1, tissue	NM_000147	0,00223143	0,183823	0,00223143	1,142	1,142	rapid up vs Slow	0,191	10,222	3,197	10,222	0,549	1,610	0,346
4735	11719825_a_at	ZAK	sterile alpha motif and leucine zipper containing kinase AZK	NM_016653 /// NM_133646	0,00229976	0,0382781	0,00229976	1,137	1,137	rapid up vs Slow	0,185	10,157	3,187	10,157	0,512	2,714	0,547
1118	11716208_s_at	GLUD1	glutamate dehydrogenase 1	NM_005271	0,00230551	0,0726126	0,00230551	0,811	-1,233	rapid down vs Slow	-0,302	10,151	-3,186	10,151	1,366	2,267	1,221
20857	11735947_x_at	ZNF221	zinc finger protein 221	NM_013359	0,00238281	0,136102	0,00238281	1,113	1,113	rapid up vs Slow	0,154	10,080	3,175	10,080	0,355	1,825	0,257
48478	11763568_a_at	FAM55B	family with sequence similarity 55, member B	NM_182495	0,00246022	0,111668	0,00246022	1,174	1,174	rapid up vs Slow	0,231	10,011	3,164	10,011	0,801	1,965	0,629
21158	11736248_at	AMIGO2	adhesion molecule with Ig-like domain 2	NM_001143668 /// NM_181847	0,00248497	0,0275656	0,00248497	1,117	1,117	rapid up vs Slow	0,160	9,989	3,161	9,989	0,383	2,943	0,452
27463	11742553_at	OR52N1	olfactory receptor, family 52, subfamily N, member 1	NM_001001913	0,00263324	0,0188508	0,00263324	1,121	1,121	rapid up vs Slow	0,164	9,865	3,141	9,865	0,405	3,209	0,526
18069	11733159_a_at	FAM84A	family with sequence similarity 84, member A	NM_145175	0,00285733	0,126031	0,00285733	0,908	-1,102	rapid down vs Slow	-0,140	9,689	-3,113	9,689	0,293	1,879	0,227
13944	11729034_s_at	ITPRIPL2	inositol 1,4,5-triphosphate receptor interacting protein-like 2	NM_001034841 /// NR_028028	0,00286696	0,317035	0,00286696	1,155	1,155	rapid up vs Slow	0,208	9,682	3,112	9,682	0,650	1,208	0,324
3647	11718737_a_at	ST3GAL1	ST3 beta-galactoside alpha-2,3-sialyltransferase 1	NM_003033 /// NM_173344	0,0030494	0,686148	0,0030494	0,682	-1,467	rapid down vs Slow	-0,553	9,550	-3,090	9,550	4,576	0,569	1,090
48360	11763450_s_at	MPHOSPH8	M-phase phosphoprotein 8	NM_017520	0,00309939	0,0176183	0,00309939	0,831	-1,203	rapid down vs Slow	-0,267	9,515	-3,085	9,515	1,069	3,256	1,464
20546	11735636_a_at	ZMYM5	zinc finger, MYM-type 5	NM_001039649 /// NM_001039650 /// NM_001142684	0,00320814	0,0330828	0,00320814	1,122	1,122	rapid up vs Slow	0,166	9,442	3,073	9,442	0,415	2,816	0,495
15961	11731051_at	KIAA1377	KIAA1377	NM_020802	0,0032749	0,00272754	0,0032749	1,108	1,108	rapid up vs Slow	0,147	9,398	3,066	9,398	0,326	4,584	0,635
47607	11762697_at	INPP5E	inositol polyphosphate-5-phosphatase, 72 kDa	NM_019892	0,00332086	0,273923	0,00332086	0,872	-1,147	rapid down vs Slow	-0,198	9,368	-3,061	9,368	0,589	1,318	0,332
36648	11751738_a_at	PPP1R3E	protein phosphatase 1, regulatory (inhibitor) subunit 3E	NR_026862	0,00334409	0,0309975	0,00334409	0,885	-1,130	rapid down vs Slow	-0,176	9,353	-3,058	9,353	0,464	2,861	0,568

15217	11730307_a_at	RIC3	resistance to inhibitors of cholinesterase 3 homolog (C. elegans)	NM_001135109 /// NM_024557	0,0033955	0,09024	0,0033955	1,117	1,117	rapid up vs Slow	0,160	9,321	3,053	9,321	0,384	2,115	0,348
26794	11741884_a_at	SMOX	spermine oxidase	NM_175839 /// NM_175840 /// NM_175841 /// NM_175842	0,00341272	0,0726555	0,00341272	1,112	1,112	rapid up vs Slow	0,153	9,310	3,051	9,310	0,351	2,267	0,342
25629	11740719_a_at	PRMT3	protein arginine methyltransferase 3	NM_001145166 /// NM_001145167 /// NM_005788	0,00347912	0,994157	0,00347912	1,126	1,126	rapid up vs Slow	0,171	9,269	3,045	9,269	0,440	0,055	0,011
48189	11763279_a_at	TMEM136	transmembrane protein 136	NM_001198670 /// NM_001198671 /// NM_001198672 /// NM_001198673 /// NM_001198674 /// NM	0,00350936	0,0845107	0,00350936	1,160	1,160	rapid up vs Slow	0,214	9,251	3,041	9,251	0,689	2,161	0,644
25402	11740492_a_at	AGAP1	ArfGAP with GTPase domain, ankyrin repeat and PH domain 1	NM_001037131 /// NM_014914	0,00352906	0,332497	0,00352906	1,160	1,160	rapid up vs Slow	0,214	9,239	3,040	9,239	0,688	1,172	0,349
35185	11750275_a_at	CYTH4	cytohesin 4	NM_013385	0,00354716	0,0528588	0,00354716	1,187	1,187	rapid up vs Slow	0,248	9,228	3,038	9,228	0,919	2,489	0,992
47615	11762705_at	LOC100128226	hypothetic protein	XM_001725696 /// XM_001725850 /// XM_001725905	0,00355276	0,0974609	0,00355276	1,328	1,328	rapid up vs Slow	0,409	9,225	3,037	9,225	2,509	2,061	2,242
9360	11724450_at	ZDHHC22	zinc finger, DHHC-type containing 22	NM_174976	0,00375483	0,185317	0,00375483	1,124	1,124	rapid up vs Slow	0,168	9,107	3,018	9,107	0,425	1,604	0,299
37329	11752419_a_at	HIVEP1	human immunodeficiency virus type I enhancer binding protein 1	NM_002114	0,00379011	0,756267	0,00379011	1,106	1,106	rapid up vs Slow	0,146	9,088	3,015	9,088	0,317	0,472	0,066
5475	11720565_a_at	NAV2	neuron navigator 2	NM_001111018 /// NM_001111019 /// NM_145117 /// NM_182964	0,00388486	0,0169247	0,00388486	1,113	1,113	rapid up vs Slow	0,155	9,035	3,006	9,035	0,359	3,285	0,523
20853	11735943_at	KRTAP19-6	keratin associated protein 19-6	NM_181612	0,00409444	0,00449053	0,00409444	1,111	1,111	rapid up vs Slow	0,152	8,925	2,987	8,925	0,344	4,225	0,651
26944	11742034_a_at	ERG	v-ets erythroblastosis virus E26 oncogene homolog (avian)	NM_001136154 /// NM_001136155 /// NM_004449 /// NM_182918	0,00414351	0,00285649	0,00414351	1,138	1,138	rapid up vs Slow	0,186	8,899	2,983	8,899	0,518	4,550	1,060
12026	11727116_	PLA1A	phospholipase	NM_015900	0,004165	0,053127	0,00416581	1,099	1,099	rapid up vs	0,137	8,888	2,981	8,888	0,280	2,486	0,314

	a_at		A1 member A		81	7				Slow							
30127	11745217_a_at	MKL2	MKL/myocardin-like 2	NM_014048	0,00422941	0,0285227	0,00422941	1,121	1,121	rapid up vs Slow	0,164	8,856	2,976	8,856	0,404	2,920	0,533
4101	11719191_s_at	FBXO9	F-box protein 9	NM_012347 /// NM_033480 /// NM_033481	0,00426786	0,83661	0,00426786	0,721	-1,387	rapid down vs Slow	-0,472	8,837	-2,973	8,837	3,340	0,359	0,543
42077	11757167_x_at	SNORD14D	small nucleolar RNA, C/D box 14D	NR_001454	0,00460683	0,0295683	0,00460683	1,112	1,112	rapid up vs Slow	0,154	8,677	2,946	8,677	0,354	2,894	0,472
29501	11744591_a_at	TTC8	tetratricopeptide repeat domain 8	NM_144596 /// NM_198309 /// NM_198310	0,00465996	0,0871264	0,00465996	1,089	1,089	rapid up vs Slow	0,123	8,653	2,942	8,653	0,225	2,140	0,223
26688	11741778_a_at	HSD11B1	hydroxysteroid (11-beta) dehydrogenase 1	NM_005525 /// NM_181755	0,00467156	0,0813728	0,00467156	1,087	1,087	rapid up vs Slow	0,120	8,648	2,941	8,648	0,217	2,187	0,220
38822	11753912_a_at	NPRL3	nitrogen permease regulator-like 3 (S. cerevisiae)	NM_001039476 /// NM_001077350	0,0047011	0,0433509	0,0047011	0,634	-1,577	rapid down vs Slow	-0,657	8,634	-2,938	8,634	6,466	2,627	7,871
26611	11741701_x_at	HIST1H2BJ	histone cluster 1, H2bj	NM_021058	0,00478507	0,356345	0,00478507	1,126	1,126	rapid up vs Slow	0,171	8,597	2,932	8,597	0,438	1,119	0,228
30021	11745111_a_at	DLGAP1	discs, large (Drosophila) homolog-associated protein 1	NM_001003809 /// NM_004746	0,00489002	0,67728	0,00489002	0,886	-1,129	rapid down vs Slow	-0,175	8,552	-2,924	8,552	0,458	0,581	0,125
47341	11762431_at	RSPH10B2	radial spoke head 10 homolog B2 (Chlamydomonas)	NM_001099697	0,00491967	0,233785	0,00491967	0,847	-1,181	rapid down vs Slow	-0,240	8,539	-2,922	8,539	0,861	1,435	0,579
24569	11739659_x_at	YIF1B	Yip1 interacting factor homolog B (S. cerevisiae)	NM_001039671 /// NM_001039672 /// NM_001039673 /// NM_001145461 /// NM_001145462 /// NM	0,00501988	0,320878	0,00501988	1,170	1,170	rapid up vs Slow	0,227	8,497	2,915	8,497	0,770	1,199	0,434
35290	11750380_a_at	ZNF423	zinc finger protein 423	NM_015069	0,00506384	0,101042	0,00506384	1,150	1,150	rapid up vs Slow	0,202	8,479	2,912	8,479	0,609	2,035	0,585
20932	11736022_at	FKBP4	FK506 binding protein 4, 59kDa	NM_002014	0,00513462	0,00860239	0,00513462	1,110	1,110	rapid up vs Slow	0,151	8,450	2,907	8,450	0,341	3,761	0,606
6568	11721658_at	LMO4	LIM domain only 4	NM_006769	0,00521107	0,229	0,00521107	1,122	1,122	rapid up vs Slow	0,166	8,419	2,902	8,419	0,414	1,450	0,285
9000	11724090_s_at	EDN3	endothelin 3	NM_000114 /// NM_207032 /// NM_207033 /// NM_207034	0,00524969	0,252288	0,00524969	1,088	1,088	rapid up vs Slow	0,122	8,404	2,899	8,404	0,221	1,379	0,145
9161	11724251_x_at	SLC39A8	solute carrier family 39 (zinc transporter), member 8	NM_001135146 /// NM_001135147 /// NM_001135148 /// NM_022154	0,00533673	0,0850183	0,00533673	1,089	1,089	rapid up vs Slow	0,123	8,370	2,893	8,370	0,228	2,157	0,235

31586	11746676_a_at	REXO1	REX1, RNA exonuclease 1 homolog (S. cerevisiae)	NM_020695	0,0053667	0,0728657	0,0053667	1,137	1,137	rapid up vs Slow	0,185	8,358	2,891	8,358	0,514	2,265	0,557
23265	11738355_x_at	NR1I3	nuclear receptor subfamily 1, group I, member 3	NM_001077469 /// NM_001077470 /// NM_001077471 /// NM_001077472 /// NM_001077473 /// NM	0,00540145	0,009585	0,00540145	1,150	1,150	rapid up vs Slow	0,202	8,345	2,889	8,345	0,611	3,685	1,079
42602	11757692_s_at	APC	adenomatous polyposis coli	NM_000038 /// NM_001127510 /// NM_001127511	0,00541829	0,200663	0,00541829	1,129	1,129	rapid up vs Slow	0,175	8,338	2,888	8,338	0,461	1,546	0,342
35409	11750499_s_at	LOC100131539 /// LOC100132396 /// ZNF705A /// ZNF705D /// ZNF705F	putative zinc finger protein 705E-like /// zinc finger protein 705D-like /// zinc finge	NM_001004328 /// NM_001039615 /// NM_001193630 /// XM_001723234 /// XM_001724873 /// XM	0,00554674	0,119589	0,00554674	1,080	1,080	rapid up vs Slow	0,111	8,290	2,879	8,290	0,186	1,916	0,172
18991	11734081_a_at	CCDC33	coiled-coil domain containing 33	NM_025055 /// NM_182791	0,00556933	0,0662236	0,00556933	1,112	1,112	rapid up vs Slow	0,153	8,281	2,878	8,281	0,349	2,332	0,393
31689	11746779_a_at	NCOA7	nuclear receptor coactivator 7	NM_001122842 /// NM_181782	0,00560521	0,207268	0,00560521	1,110	1,110	rapid up vs Slow	0,150	8,268	2,875	8,268	0,338	1,523	0,249
33587	11748677_a_at	ULK3	unc-51-like kinase 3 (C. elegans)	NM_001099436	0,00569005	0,160904	0,00569005	0,904	-1,107	rapid down vs Slow	-0,146	8,237	-2,870	8,237	0,320	1,705	0,265
25020	11740110_a_at	ATXN7L1	ataxin 7-like 1	NM_020725 /// NM_138495 /// NM_152749	0,00569911	0,719129	0,00569911	0,875	-1,142	rapid down vs Slow	-0,192	8,233	-2,869	8,233	0,552	0,523	0,140
33327	11748417_a_at	PROX1	prospero homeobox 1	NM_002763	0,00578481	0,0335577	0,00578481	1,150	1,150	rapid up vs Slow	0,202	8,203	2,864	8,203	0,610	2,806	0,835
48362	11763452_at	ATPBD4	ATP binding domain 4	NM_001141972 /// NM_080650	0,00581516	0,872844	0,00581516	1,081	1,081	rapid up vs Slow	0,113	8,192	2,862	8,192	0,190	0,306	0,028
9931	11725021_at	COL23A1	collagen, type XXIII, alpha 1	NM_173465	0,00601913	0,189932	0,00601913	1,129	1,129	rapid up vs Slow	0,175	8,120	2,850	8,120	0,460	1,586	0,359
15369	11730459_a_at	C17orf74	chromosome 17 open reading frame 74	NM_175734	0,00606936	0,0588473	0,00606936	1,091	1,091	rapid up vs Slow	0,125	8,103	2,847	8,103	0,236	2,414	0,281
21631	11736721_x_at	RPL32	ribosomal protein L32	NM_000994 /// NM_001007073 /// NM_001007074	0,0061136	0,371794	0,0061136	0,850	-1,177	rapid down vs Slow	-0,235	8,088	-2,844	8,088	0,827	1,086	0,444
34368	11749458_a_at	RAP1GAP2	RAP1 GTPase activating protein 2	NM_001100398 /// NM_015085	0,00612288	0,305319	0,00612288	1,101	1,101	rapid up vs Slow	0,139	8,085	2,843	8,085	0,288	1,236	0,176
11030	11726120_a_at	BMP2K	BMP2 inducible kinase	NM_017593 /// NM_198892	0,00615012	0,655458	0,00615012	0,776	-1,289	rapid down vs Slow	-0,366	8,076	-2,842	8,076	2,008	0,612	0,609

20949	11736039_a_at	KIF1A	kinesin family member 1A	NM_004321	0,00619185	0,449453	0,00619185	1,110	1,110	rapid up vs Slow	0,150	8,062	2,839	8,062	0,339	0,936	0,157
34479	11749569_a_at	CAPRIN1	cell cycle associated protein 1	NM_005898 /// NM_203364	0,00619444	0,760195	0,00619444	1,140	1,140	rapid up vs Slow	0,189	8,061	2,839	8,061	0,537	0,466	0,124
40665	11755755_a_at	DDX26B	DEAD/H (Asp-Glu-Ala-Asp/His) box polypeptide 26B	NM_182540	0,00625895	0,000649743	0,00625895	0,873	-1,146	rapid down vs Slow	-0,196	8,040	-2,835	8,040	0,576	5,641	1,616
16967	11732057_a_at	CDC7	cell division cycle 7 homolog (S. cerevisiae)	NM_001134419 /// NM_001134420 /// NM_003503	0,00633667	0,104086	0,00633667	1,102	1,102	rapid up vs Slow	0,141	8,014	2,831	8,014	0,296	2,014	0,298
29938	11745028_a_at	NKRF	NFKB repressing factor	NM_001173487 /// NM_001173488 /// NM_017544	0,00638828	0,0168329	0,00638828	0,775	-1,290	rapid down vs Slow	-0,367	7,998	-2,828	7,998	2,022	3,288	3,326
36976	11752066_a_at	RAI1	retinoic acid induced 1	NM_030665	0,00653318	0,017855	0,00653318	1,148	1,148	rapid up vs Slow	0,199	7,951	2,820	7,951	0,593	3,247	0,968
24859	11739949_at	PFKFB2	6-phosphofructo-2-kinase/fructose-2,6-biphosphatase 2	NM_001018053 /// NM_006212	0,0066141	0,699537	0,0066141	1,139	1,139	rapid up vs Slow	0,188	7,926	2,815	7,926	0,527	0,550	0,146
15372	11730462_s_at	OTX2	orthodenticle homeobox 2	NM_021728 /// NM_172337	0,00665306	0,121405	0,00665306	1,096	1,096	rapid up vs Slow	0,132	7,914	2,813	7,914	0,260	1,906	0,251
42734	11757824_s_at	RGL1	ral guanine nucleotide dissociation stimulator-like 1	NM_015149	0,00668462	0,111829	0,00668462	1,146	1,146	rapid up vs Slow	0,196	7,904	2,811	7,904	0,576	1,964	0,573
37251	11752341_a_at	F8	coagulation factor VIII, procoagulant component	NM_000132 /// NM_019863	0,00670587	0,025277	0,00670587	1,167	1,167	rapid up vs Slow	0,222	7,898	2,810	7,898	0,741	3,004	1,128
34567	11749657_a_at	NAV2	neuron navigator 2	NM_001111018 /// NM_001111019 /// NM_145117 /// NM_182964	0,00671595	0,0781043	0,00671595	1,133	1,133	rapid up vs Slow	0,180	7,895	2,810	7,895	0,488	2,216	0,548
43806	11758896_s_at	CCNI	cyclin I	NM_006835	0,00681867	0,392303	0,00681867	0,806	-1,241	rapid down vs Slow	-0,312	7,863	-2,804	7,863	1,456	1,044	0,773
37441	11752531_a_at	MDH1B	malate dehydrogenase 1B, NAD (soluble)	NM_001039845	0,00694513	0,0100916	0,00694513	1,150	1,150	rapid up vs Slow	0,201	7,826	2,797	7,826	0,608	3,649	1,133
37973	11753063_a_at	SIDT2	SID1 transmembrane family, member 2	NM_001040455	0,00711784	0,294656	0,00711784	1,145	1,145	rapid up vs Slow	0,196	7,775	2,788	7,775	0,573	1,263	0,372
22967	11738057_x_at	TMEM136	transmembrane protein 136	NM_001198670 /// NM_001198671 /// NM_001198672 /// NM_001198673 /// NM_001198674 /// NM	0,00719949	0,310027	0,00719949	0,925	-1,081	rapid down vs Slow	-0,113	7,752	-2,784	7,752	0,190	1,225	0,120
18323	11733413_a_at	SLC26A6	solute carrier family 26,	NM_001040454 ///	0,00725671	0,984488	0,00725671	0,852	-1,174	rapid down vs Slow	-0,231	7,736	-2,781	7,736	0,801	0,092	0,038

			member 6	NM_022911 /// NM_134263 /// NM_134426												
35730	11750820_a_at	MIPOL1	mirror-image polydactyly 1	NM_001195296 /// NM_001195297 /// NM_138731	0,00727134	0,101014	0,00727134	1,066	1,066	rapid up vs Slow	0,092	7,732	2,781	7,732	0,128	2,036	0,134
4542	11719632_a_at	ABCC4	ATP-binding cassette, sub-family C (CFTR/MRP), member 4	NM_001105515 /// NM_005845	0,00728775	0,692387	0,00728775	1,115	1,115	rapid up vs Slow	0,157	7,727	2,780	7,727	0,369	0,560	0,107
37092	11752182_a_at	DLG1	discs, large homolog 1 (Drosophila)	NM_001098424 /// NM_004087	0,00732067	0,477404	0,00732067	1,125	1,125	rapid up vs Slow	0,170	7,718	2,778	7,718	0,434	0,887	0,200
17187	11732277_a_at	FOXP1	forkhead box P1	NM_001012505 /// NM_032682	0,00732984	0,170973	0,00732984	0,813	-1,230	rapid down vs Slow	-0,299	7,715	-2,778	7,715	1,337	1,662	1,152
38248	11753338_x_at	FHL1	four and a half LIM domains 1	NM_001159699 /// NM_001159700 /// NM_001159701 /// NM_001159702 /// NM_001159703 /// NM	0,00733308	0,298648	0,00733308	1,119	1,119	rapid up vs Slow	0,162	7,714	2,777	7,714	0,394	1,253	0,256
39294	11754384_x_at	COG4	component of oligomeric golgi complex 4	NM_001195139 /// NM_015386	0,00734474	0,0130373	0,00734474	0,847	-1,181	rapid down vs Slow	-0,239	7,711	-2,777	7,711	0,859	3,468	1,546
20709	11735799_at	SOX14	SRY (sex determining region Y)-box 14	NM_004189	0,00736556	0,126105	0,00736556	1,113	1,113	rapid up vs Slow	0,154	7,705	2,776	7,705	0,356	1,879	0,348
26011	11741101_a_at	ZNF655	zinc finger protein 655	NM_001009958 /// NM_001009960 /// NM_001083956 /// NM_001085366 /// NM_001085367 /// NM	0,00748116	0,414187	0,00748116	1,123	1,123	rapid up vs Slow	0,167	7,673	2,770	7,673	0,417	1,001	0,218
34715	11749805_a_at	MASP1	mannan-binding lectin serine peptidase 1 (C4/C2 activating component of Ra-reactive fac	NM_001031849 /// NM_001879 /// NM_139125 /// NR_033519	0,00754731	0,023908	0,00754731	1,134	1,134	rapid up vs Slow	0,182	7,655	2,767	7,655	0,494	3,043	0,785
16410	11731500_a_at	KRT19	keratin 19	NM_002276	0,0075656	0,34093	0,0075656	1,131	1,131	rapid up vs Slow	0,177	7,651	2,766	7,651	0,472	1,153	0,284
14978	11730068_a_at	HGF	hepatocyte growth factor (hepapoietin A; scatter factor)	NM_000601 /// NM_001010931 /// NM_001010932 /// NM_001010933 /// NM_001010934	0,00760476	0,713098	0,00760476	1,090	1,090	rapid up vs Slow	0,125	7,640	2,764	7,640	0,232	0,531	0,065

326	11715416_s_at	DDB1	damage-specific DNA binding protein 1, 127kDa	NM_001923	0,0078693	0,526776	0,0078693	0,788	-1,269	rapid down vs Slow	-0,344	7,570	-2,751	7,570	1,770	0,805	0,753
41576	11756666_x_at	MCM2	minichromosome maintenance complex component 2	NM_004526	0,00791284	0,0162331	0,00791284	0,866	-1,155	rapid down vs Slow	-0,208	7,559	-2,749	7,559	0,647	3,314	1,135
21897	11736987_a_at	NUBPL	nucleotide binding protein-like	NM_025152	0,00791454	0,00482232	0,00791454	1,115	1,115	rapid up vs Slow	0,157	7,559	2,749	7,559	0,368	4,173	0,813
35181	11750271_a_at	PANX2	pannexin 2	NM_001160300 /// NM_052839 /// NR_027691	0,0079541	0,0146421	0,0079541	1,112	1,112	rapid up vs Slow	0,153	7,548	2,747	7,548	0,349	3,386	0,626
47645	11762735_a_at	BEST3	bestrophin 3	NM_032735 /// NM_152439	0,00796871	0,179086	0,00796871	1,124	1,124	rapid up vs Slow	0,168	7,545	2,747	7,545	0,425	1,628	0,367
39380	11754470_a_at	CHTF18	CTF18, chromosome transmission fidelity factor 18 homolog (S. cerevisiae)	NM_022092	0,00800922	0,46303	0,00800922	0,796	-1,256	rapid down vs Slow	-0,329	7,534	-2,745	7,534	1,618	0,912	0,783
26125	11741215_at	HIST1H2AB	histone cluster 1, H2ab	NM_003513	0,00803025	0,177394	0,00803025	1,127	1,127	rapid up vs Slow	0,173	7,529	2,744	7,529	0,447	1,635	0,388
30395	11745485_a_at	OPALIN	oligodendrocytic myelin paranodal and inner loop protein	NM_001040102 /// NM_001040103 /// NM_033207	0,00828929	0,0553507	0,00828929	1,104	1,104	rapid up vs Slow	0,143	7,464	2,732	7,464	0,306	2,457	0,403
12064	11727154_at	LRRC1	leucine rich repeat containing 1	NM_018214	0,00832074	0,0014305	0,00832074	1,099	1,099	rapid up vs Slow	0,136	7,457	2,731	7,457	0,276	5,055	0,747
13178	11728268_a_at	C17orf70	chromosome 17 open reading frame 70	NM_025161 /// NR_033338	0,00851889	0,0809689	0,00851889	1,115	1,115	rapid up vs Slow	0,157	7,409	2,722	7,409	0,370	2,191	0,437
3329	11718419_at	FCER1G	Fc fragment of IgE, high affinity I, receptor for; gamma polypeptide	NM_004106	0,00856649	0,920334	0,00856649	1,190	1,190	rapid up vs Slow	0,251	7,398	2,720	7,398	0,945	0,230	0,118
4524	11719614_a_at	LARP4	La ribonucleoprotein domain family, member 4	NM_001170803 /// NM_001170804 /// NM_001170808 /// NM_052879 /// NM_199188 /// NM_19919	0,00863631	0,0446609	0,00863631	0,867	-1,154	rapid down vs Slow	-0,206	7,381	-2,717	7,381	0,636	2,607	0,899
8418	11723508_at	ZNF609	zinc finger protein 609	NM_015042	0,00873542	0,85708	0,00873542	0,887	-1,127	rapid down vs Slow	-0,173	7,358	-2,713	7,358	0,449	0,329	0,080
36576	11751666_a_at	TUFT1	tuftelin 1	NM_001126337 /// NM_020127	0,00876047	0,404716	0,00876047	1,110	1,110	rapid up vs Slow	0,151	7,352	2,711	7,352	0,341	1,020	0,189
47123	11762213_x_at	ZNF66	Zinc finger protein 66	---	0,00881033	0,0963391	0,00881033	1,143	1,143	rapid up vs Slow	0,192	7,341	2,709	7,341	0,555	2,069	0,626
34147	11749237_a_at	MRPS27	mitochondrial ribosomal protein S27	NM_015084	0,00895611	0,359088	0,00895611	1,109	1,109	rapid up vs Slow	0,150	7,307	2,703	7,307	0,336	1,113	0,205
21900	11736990_at	LPAL2	lipoprotein, Lp(a)-like 2, pseudogene	NR_028092 /// NR_028093	0,00901017	0,00208736	0,00901017	1,117	1,117	rapid up vs Slow	0,160	7,295	2,701	7,295	0,383	4,778	1,003
5843	11720933_s	DAGLB	diacylglycerol	NM_0011429	0,009157	0,793785	0,00915703	1,103	1,103	rapid up vs	0,141	7,263	2,695	7,263	0,299	0,420	0,069

	_at		lipase, beta	36 /// NM_139179	03					Slow							
10841	11725931_at	HSPA5	heat shock 70kDa protein 5 (glucose-regulated protein, 78kDa)	NM_005347	0,00917461	0,239459	0,00917461	0,858	-1,166	rapid down vs Slow	-0,222	7,259	-2,694	7,259	0,736	1,417	0,575
26705	11741795_at	TECTA	tectorin alpha	NM_005422	0,00928267	0,167307	0,00928267	1,098	1,098	rapid up vs Slow	0,135	7,235	2,690	7,235	0,273	1,677	0,253
10538	11725628_a_at	CNP	2',3'-cyclic nucleotide 3' phosphodiesterase	NM_033133	0,00932744	0,127333	0,00932744	1,153	1,153	rapid up vs Slow	0,205	7,225	2,688	7,225	0,631	1,872	0,653
32031	11747121_a_at	MFSD9	major facilitator superfamily domain containing 9	NM_032718	0,00936887	0,173726	0,00936887	1,117	1,117	rapid up vs Slow	0,160	7,216	2,686	7,216	0,382	1,650	0,349
17410	11732500_a_at	FAM3B	family with sequence similarity 3, member B	NM_058186 /// NM_206964	0,00941838	0,198181	0,00941838	1,441	1,441	rapid up vs Slow	0,527	7,206	2,684	7,206	4,159	1,555	3,591
35253	11750343_a_at	MACC1	metastasis associated in colon cancer 1	NM_182762	0,00943872	0,104678	0,00943872	1,110	1,110	rapid up vs Slow	0,151	7,201	2,684	7,201	0,340	2,010	0,380
30276	11745366_x_at	POMGNT1	protein O-linked mannose beta1,2-N-acetylglucosaminyltransferase	NM_017739 /// NR_024332	0,00949127	0,712549	0,00949127	0,805	-1,242	rapid down vs Slow	-0,313	7,190	-2,681	7,190	1,463	0,532	0,433
47222	11762312_at	---	---	---	0,00949434	0,0526313	0,00949434	1,156	1,156	rapid up vs Slow	0,209	7,190	2,681	7,190	0,656	2,492	0,909
9480	11724570_at	APPBP2	amyloid beta precursor protein (cytoplasmic tail) binding protein 2	NM_006380	0,00962739	0,328721	0,00962739	0,878	-1,138	rapid down vs Slow	-0,187	7,161	-2,676	7,161	0,524	1,181	0,345
28730	11743820_a_at	C1orf57	chromosome 1 open reading frame 57	NM_032324	0,00969328	0,401088	0,00969328	0,814	-1,229	rapid down vs Slow	-0,297	7,148	-2,674	7,148	1,324	1,027	0,761
35124	11750214_a_at	RNF114	ring finger protein 114	NM_018683	0,00980488	0,0138509	0,00980488	0,862	-1,161	rapid down vs Slow	-0,215	7,125	-2,669	7,125	0,693	3,425	1,333
27102	11742192_a_at	CMKLR1	chemokine-like receptor 1	NM_001142343 /// NM_001142344 /// NM_001142345 /// NM_004072	0,00982419	0,0740434	0,00982419	1,121	1,121	rapid up vs Slow	0,165	7,121	2,668	7,121	0,408	2,254	0,516
43981	11759071_at	PROX1	prospero homeobox 1	NM_002763	0,00982646	0,179947	0,00982646	1,130	1,130	rapid up vs Slow	0,176	7,120	2,668	7,120	0,465	1,625	0,424
19753	11734843_x_at	MPHOSPH8	M-phase phosphoprotein 8	NM_017520	0,00990421	0,0845237	0,00990421	0,811	-1,233	rapid down vs Slow	-0,302	7,104	-2,665	7,104	1,366	2,161	1,662
23422	11738512_a_at	GADL1	glutamate decarboxylase-like 1	NM_207359	0,00994031	0,00633643	0,00994031	1,077	1,077	rapid up vs Slow	0,107	7,097	2,664	7,097	0,172	3,979	0,386
7650	11722740_a_at	DDC	dopa decarboxylase (aromatic L-amino acid decarboxylase)	NM_000790 /// NM_001082971	0,00997431	0,365761	0,00997431	1,103	1,103	rapid up vs Slow	0,141	7,090	2,663	7,090	0,297	1,099	0,184
6762	11721852_at	NCAPH	non-SMC condensin I complex, subunit H	NM_015341	0,0100395	0,0295383	0,0100395	1,147	1,147	rapid up vs Slow	0,198	7,077	2,660	7,077	0,590	2,895	0,965
17722	11732812_at	C11orf45	chromosome 11 open reading	NM_145013	0,0100978	0,146234	0,0100978	1,173	1,173	rapid up vs Slow	0,230	7,065	2,658	7,065	0,796	1,774	0,799

			frame 45														
26873	11741963_x_at	GLRA3	glycine receptor, alpha 3	NM_001042543 /// NM_006529	0,0101433	0,146474	0,0101433	1,103	1,103	rapid up vs Slow	0,142	7,056	2,656	7,056	0,302	1,772	0,304
45205	11760295_at	EBNA1BP2	EBNA1 binding protein 2	NM_001159936 /// NM_006824	0,0101507	0,163668	0,0101507	0,894	-1,119	rapid down vs Slow	-0,162	7,055	-2,656	7,055	0,394	1,693	0,378
37714	11752804_a_at	SERPINB3	serpin peptidase inhibitor, clade B (ovalbumin), member 3	NM_006919	0,0102294	0,0804571	0,0102294	1,148	1,148	rapid up vs Slow	0,199	7,039	2,653	7,039	0,596	2,195	0,743
5905	11720995_at	ADCY2	adenylate cyclase 2 (brain)	NM_020546	0,0104361	0,0580126	0,0104361	1,098	1,098	rapid up vs Slow	0,135	6,999	2,646	6,999	0,275	2,424	0,381
11391	11726481_a_at	CES4A	carboxylesterase 4A	NM_001190201 /// NM_001190202 /// NM_173815	0,0105029	0,013461	0,0105029	1,149	1,149	rapid up vs Slow	0,201	6,986	2,643	6,986	0,604	3,445	1,191
11770	11726860_a_at	DCAKD	dephospho-CoA kinase domain containing	NM_001128631 /// NM_024819	0,0105156	0,219353	0,0105156	1,106	1,106	rapid up vs Slow	0,146	6,984	2,643	6,984	0,318	1,481	0,269
32184	11747274_x_at	40057	septin 9	NM_001113491 /// NM_001113492 /// NM_001113493 /// NM_001113494 /// NM_001113495 /// NM	0,0105473	0,793635	0,0105473	1,149	1,149	rapid up vs Slow	0,201	6,978	2,642	6,978	0,603	0,420	0,145
32193	11747283_a_at	NDRG4	NDRG family member 4	NM_001130487 /// NM_020465 /// NM_022910	0,0106241	0,107704	0,0106241	1,108	1,108	rapid up vs Slow	0,148	6,963	2,639	6,963	0,327	1,990	0,374
37741	11752831_s_at	ZNF747	zinc finger protein 747	NM_023931	0,0106928	0,0159269	0,0106928	1,114	1,114	rapid up vs Slow	0,155	6,950	2,636	6,950	0,362	3,327	0,693
15769	11730859_a_at	SLC6A7	solute carrier family 6 (neurotransmitter transporter, L-proline), member 7	NM_014228	0,0106981	0,0451549	0,0106981	1,131	1,131	rapid up vs Slow	0,177	6,949	2,636	6,949	0,472	2,599	0,706
5358	11720448_at	SOX9	SRY (sex determining region Y)-box 9	NM_000346	0,0108102	0,0248508	0,0108102	1,116	1,116	rapid up vs Slow	0,159	6,929	2,632	6,929	0,377	3,016	0,656
13008	11728098_x_at	CROCC	ciliary rootlet coiled-coil, rootletin	NM_014675	0,0108312	0,407094	0,0108312	0,870	-1,150	rapid down vs Slow	-0,201	6,925	-2,631	6,925	0,605	1,015	0,355
18035	11733125_a_at	PEX19	peroxisomal biogenesis factor 19	NM_001193644 /// NM_002857 /// NR_036492 /// NR_036493	0,0108434	0,358914	0,0108434	1,099	1,099	rapid up vs Slow	0,137	6,922	2,631	6,922	0,279	1,113	0,180
1334	11716424_a_at	PCDHGA1 /// PCDHGA10 /// PCDHGA11 /// PCDHGA12 /// PCDHGA2 /// PCDHGA	protocadherin gamma subfamily A, 1 /// protocadherin gamma subfamily A, 10 /// protocad	NM_002588 /// NM_003735 /// NM_003736 /// NM_014004 /// NM_018912 /// NM_018913	0,0108834	0,530389	0,0108834	1,159	1,159	rapid up vs Slow	0,213	6,915	2,630	6,915	0,679	0,799	0,314

		3 /// PCDHGA4 /// PCDHGA5 /// PCDHGA6 /// PCDHGA7 /// PCDHGA8 /// PCDHGA9 /// PCDHGB1 /// PCDHGB2 /// PCDHGB3 /// PCDHGB4 /// PCDHGB5 /// PCDHGB6 /// PCDHGB7 /// PCDHGC3 /// PCDHGC4 /// PCDHGC5		/// NM_													
12173	11727263_a_at	ENTPD7	ectonucleoside triphosphate diphosphohydrolase 7	NM_020354	0,0109241	0,297887	0,0109241	1,109	1,109	rapid up vs Slow	0,150	6,908	2,628	6,908	0,336	1,255	0,244
32699	11747789_a_at	ZNF394	zinc finger protein 394	NM_032164	0,0109764	0,240475	0,0109764	1,120	1,120	rapid up vs Slow	0,163	6,898	2,626	6,898	0,399	1,414	0,327
30168	11745258_s_at	RBM14	RNA binding motif protein 14	NM_001198836 /// NM_001198837 /// NM_006328	0,0109796	0,972016	0,0109796	0,847	-1,181	rapid down vs Slow	-0,240	6,897	-2,626	6,897	0,865	0,127	0,064
21105	11736195_a_at	CREB1	cAMP responsive element binding protein 1	NM_004379 /// NM_134442	0,0111845	0,134475	0,0111845	0,815	-1,227	rapid down vs Slow	-0,295	6,860	-2,619	6,860	1,300	1,833	1,390
33909	11748999_a_at	PARP11	poly (ADP-ribose) polymerase family, member 11	NM_020367	0,0112709	0,230769	0,0112709	1,139	1,139	rapid up vs Slow	0,187	6,845	2,616	6,845	0,526	1,444	0,444
21456	11736546_at	FANCD2	Fanconi anemia, complementation group D2	NM_001018115 /// NM_033084	0,0112936	0,0834141	0,0112936	1,124	1,124	rapid up vs Slow	0,169	6,841	2,616	6,841	0,429	2,170	0,544
32767	11747857_a_at	MPDZ	multiple PDZ domain protein	NM_003829	0,0113941	0,0352204	0,0113941	1,140	1,140	rapid up vs Slow	0,189	6,823	2,612	6,823	0,537	2,772	0,873
6429	11721519_at	ZDHHC17	zinc finger, DHHC-type containing 17	NM_015336	0,0114601	0,368443	0,0114601	1,126	1,126	rapid up vs Slow	0,172	6,812	2,610	6,812	0,441	1,093	0,283
38599	11753689_x_at	MDM2	Mdm2 p53 binding protein homolog (mouse)	NM_002392	0,0115763	0,254652	0,0115763	1,096	1,096	rapid up vs Slow	0,132	6,792	2,606	6,792	0,262	1,372	0,212
240	11715330_x	ZNF735	zinc finger	NM_0011595	0,011582	0,115365	0,0115821	1,142	1,142	rapid up vs	0,191	6,791	2,606	6,791	0,549	1,942	0,628

	_at		protein 735	24	1					Slow							
17786	11732876_at	TRPM8	transient receptor potential cation channel, subfamily M, member 8	NM_024080	0,0115965	0,0756499	0,0115965	1,082	1,082	rapid up vs Slow	0,113	6,788	2,605	6,788	0,192	2,239	0,254
19494	11734584_at	TAAR2	trace amine associated receptor 2	NM_001033080 /// NM_014626	0,0116036	0,0587954	0,0116036	1,160	1,160	rapid up vs Slow	0,214	6,787	2,605	6,787	0,686	2,415	0,977
46251	11761341_at	SLC9A1	solute carrier family 9 (sodium/hydrogen exchanger), member 1	NM_003047	0,0116173	0,566485	0,0116173	1,134	1,134	rapid up vs Slow	0,182	6,785	2,605	6,785	0,496	0,743	0,217
3971	11719061_a_at	ATRN	attractin	NM_139321 /// NM_139322	0,0116392	0,321981	0,0116392	1,106	1,106	rapid up vs Slow	0,145	6,781	2,604	6,781	0,316	1,196	0,223
31920	11747010_a_at	VSIG4	V-set and immunoglobulin domain containing 4	NM_001100431 /// NM_001184830 /// NM_001184831 /// NM_007268	0,0116842	0,608633	0,0116842	1,101	1,101	rapid up vs Slow	0,139	6,773	2,603	6,773	0,291	0,680	0,117
19878	11734968_a_at	THEMIS	thymocyte selection associated	NM_001010923 /// NM_001164685 /// NM_001164687	0,0116961	0,017542	0,0116961	0,915	-1,093	rapid down vs Slow	-0,128	6,771	-2,602	6,771	0,246	3,260	0,475
7688	11722778_s_at	ENPP2	ectonucleotide pyrophosphatase/phosphodiesterase 2	NM_001040092 /// NM_001130863 /// NM_006209	0,01172	0,497157	0,01172	1,090	1,090	rapid up vs Slow	0,125	6,767	2,601	6,767	0,233	0,854	0,117
3823	11718913_s_at	PDZD2	PDZ domain containing 2	NM_178140	0,0117456	0,073251	0,0117456	1,140	1,140	rapid up vs Slow	0,189	6,763	2,601	6,763	0,537	2,261	0,718
26103	11741193_a_at	ACP2	acid phosphatase 2, lysosomal	NM_001131064 /// NM_001610	0,0117612	0,00436447	0,0117612	1,098	1,098	rapid up vs Slow	0,135	6,760	2,600	6,760	0,273	4,245	0,686
43928	11759018_at	MATR3	matrin 3	NM_001194954 /// NM_001194955 /// NM_001194956 /// NM_018834 /// NM_199189 /// NR_03653	0,0118248	0,751756	0,0118248	0,822	-1,216	rapid down vs Slow	-0,282	6,749	-2,598	6,749	1,191	0,478	0,337
14382	11729472_a_at	FANCA	Fanconi anemia, complementation group A	NM_000135 /// NM_001018112	0,0121279	0,00477954	0,0121279	1,081	1,081	rapid up vs Slow	0,112	6,699	2,588	6,699	0,188	4,180	0,468
18734	11733824_at	AGPAT3	1-acylglycerol-3-phosphate O-acyltransferase 3	NM_001037553 /// NM_020132	0,0122161	0,16386	0,0122161	1,154	1,154	rapid up vs Slow	0,206	6,684	2,585	6,684	0,638	1,692	0,646
26799	11741889_at	DUXA	double homeobox A	NM_001012729	0,0122378	0,439882	0,0122378	1,126	1,126	rapid up vs Slow	0,171	6,681	2,585	6,681	0,440	0,953	0,251
35311	11750401_a_at	UVRAG	UV radiation resistance associated gene	NM_003369	0,0123218	0,776472	0,0123218	1,099	1,099	rapid up vs Slow	0,136	6,667	2,582	6,667	0,277	0,444	0,074
31058	11746148_a_at	SLC25A29	solute carrier family 25,	NM_001039355	0,0123962	0,47671	0,0123962	1,137	1,137	rapid up vs Slow	0,185	6,655	2,580	6,655	0,514	0,888	0,274

			member 29														
20129	11735219_at	COL10A1	collagen, type X, alpha 1	NM_000493	0,0124469	0,0124149	0,0124469	1,135	1,135	rapid up vs Slow	0,182	6,647	2,578	6,647	0,498	3,502	1,049
16809	11731899_s_at	PPAT	phosphoribosyl pyrophosphate amidotransferase	NM_002703	0,0125639	0,371392	0,0125639	0,858	-1,166	rapid down vs Slow	-0,221	6,629	-2,575	6,629	0,733	1,087	0,480
37044	11752134_a_at	MED12L	mediator complex subunit 12-like	NM_053002	0,0126667	0,0175445	0,0126667	1,118	1,118	rapid up vs Slow	0,161	6,612	2,571	6,612	0,388	3,259	0,765
2081	11717171_at	BCL2L2	BCL2-like 2	NM_004050	0,012744	0,838988	0,012744	1,114	1,114	rapid up vs Slow	0,155	6,600	2,569	6,600	0,360	0,356	0,078
20073	11735163_x_at	OSBPL5	oxysterol binding protein-like 5	NM_001144063 /// NM_020896 /// NM_145638	0,012761	0,704786	0,012761	1,131	1,131	rapid up vs Slow	0,178	6,598	2,569	6,598	0,476	0,543	0,157
21852	11736942_x_at	DUOXA1	dual oxidase maturation factor 1	NM_144565	0,0129484	0,00164992	0,0129484	1,109	1,109	rapid up vs Slow	0,149	6,569	2,563	6,569	0,335	4,950	1,008
13167	11728257_at	RAD18	RAD18 homolog (S. cerevisiae)	NM_020165	0,0129721	0,221056	0,0129721	1,118	1,118	rapid up vs Slow	0,161	6,565	2,562	6,565	0,389	1,476	0,350
8419	11723509_s_at	ZNF609	zinc finger protein 609	NM_015042	0,01302	0,964801	0,01302	0,833	-1,200	rapid down vs Slow	-0,263	6,558	-2,561	6,558	1,037	0,144	0,091
5181	11720271_at	RIMS4	regulating synaptic membrane exocytosis 4	NM_182970	0,0131012	0,0126727	0,0131012	1,122	1,122	rapid up vs Slow	0,167	6,546	2,558	6,546	0,416	3,488	0,887
7486	11722576_at	AP1G1	adaptor-related protein complex 1, gamma 1 subunit	NM_001030007 /// NM_001128	0,0131695	0,0450378	0,0131695	0,885	-1,130	rapid down vs Slow	-0,176	6,535	-2,556	6,535	0,465	2,601	0,740
46134	11761224_x_at	KRT16P1	keratin 16 pseudogene 1	---	0,0131732	0,0315389	0,0131732	1,147	1,147	rapid up vs Slow	0,198	6,535	2,556	6,535	0,586	2,849	1,022
5152	11720242_a_at	TCN2	transcobalamin II	NM_000355 /// NM_001184726	0,0133117	0,0212387	0,0133117	1,165	1,165	rapid up vs Slow	0,220	6,514	2,552	6,514	0,725	3,126	1,391
16064	11731154_x_at	MTRR	5-methyltetrahydrofolate-homocysteine methyltransferase reductase	NM_002454 /// NM_024010	0,013331	0,10996	0,013331	1,175	1,175	rapid up vs Slow	0,233	6,511	2,552	6,511	0,810	1,976	0,984
8578	11723668_at	SEMA3C	sema domain, immunoglobulin domain (Ig), short basic domain, secreted, (semaphorin) 3C	NM_006379	0,0133511	0,603444	0,0133511	1,110	1,110	rapid up vs Slow	0,151	6,508	2,551	6,508	0,340	0,688	0,144
30582	11745672_a_at	NFATC4	nuclear factor of activated T-cells, cytoplasmic, calcineurin-dependent 4	NM_001136022 /// NM_004554	0,0133901	0,351127	0,0133901	1,108	1,108	rapid up vs Slow	0,149	6,502	2,550	6,502	0,330	1,130	0,230
30051	11745141_a_at	PADI2	peptidyl arginine deiminase, type II	NM_007365	0,0134232	0,340672	0,0134232	1,188	1,188	rapid up vs Slow	0,249	6,498	2,549	6,498	0,929	1,153	0,659
9649	11724739_s_at	TMEM204	transmembrane protein 204	NM_024600	0,0134286	0,492326	0,0134286	0,766	-1,306	rapid down vs Slow	-0,385	6,497	-2,549	6,497	2,220	0,862	1,178
48181	11763271_at	PEX11A	peroxisomal biogenesis factor 11 alpha	NM_003847	0,0134402	0,0260153	0,0134402	1,108	1,108	rapid up vs Slow	0,148	6,495	2,549	6,495	0,330	2,984	0,606
11264	11726354_at	ENPP1	ectonucleotide pyrophosphatas	NM_006208	0,0134609	0,17059	0,0134609	1,152	1,152	rapid up vs Slow	0,204	6,492	2,548	6,492	0,626	1,663	0,642

			e/phosphodiesterase 1															
30780	11745870_a_at	MTHFD2	methylenetetrahydrofolate dehydrogenase (NADP+ dependent) 2, methenyltetrahydrofolate c	NM_006636 /// NR_027405	0,0135486	0,107717	0,0135486	0,857	-1,167	rapid down vs Slow	-0,223	6,479	-2,545	6,479	0,744	1,990	0,914
25695	11740785_x_at	RGPD4	RANBP2-like and GRIP domain containing 4	NM_182588	0,013554	0,0227532	0,013554	0,881	-1,135	rapid down vs Slow	-0,183	6,478	-2,545	6,478	0,502	3,077	0,955
7910	11723000_at	MYL7	myosin, light chain 7, regulatory	NM_021223	0,0135633	0,0581106	0,0135633	1,102	1,102	rapid up vs Slow	0,140	6,477	2,545	6,477	0,294	2,423	0,440
14965	11730055_a_at	NPC1L1	NPC1 (Niemann-Pick disease, type C1, gene)-like 1	NM_001101648 /// NM_013389	0,0135739	0,0917836	0,0135739	1,108	1,108	rapid up vs Slow	0,148	6,475	2,545	6,475	0,327	2,103	0,424
36744	11751834_a_at	CLEC2A	C-type lectin domain family 2, member A	NM_001130711	0,0135888	0,143275	0,0135888	1,078	1,078	rapid up vs Slow	0,108	6,473	2,544	6,473	0,175	1,788	0,193
44767	11759857_at	LOC554223	hypothetical LOC554223	XR_001113 /// XR_001114 /// XR_001115 /// XR_001116	0,0136654	0,342793	0,0136654	1,106	1,106	rapid up vs Slow	0,145	6,462	2,542	6,462	0,315	1,149	0,224
20036	11735126_at	N4BP3	NEDD4 binding protein 3	NM_015111	0,0136747	0,313302	0,0136747	1,111	1,111	rapid up vs Slow	0,152	6,461	2,542	6,461	0,344	1,217	0,259
8184	11723274_a_at	SLC35F2	solute carrier family 35, member F2	NM_017515	0,0137071	0,856478	0,0137071	1,153	1,153	rapid up vs Slow	0,206	6,456	2,541	6,456	0,635	0,330	0,130
45800	11760890_x_at	LOC220729	succinate dehydrogenase complex, subunit A, flavoprotein pseudogene	NR_003266	0,0137648	0,476236	0,0137648	1,076	1,076	rapid up vs Slow	0,105	6,448	2,539	6,448	0,167	0,889	0,092
20781	11735871_s_at	CCR8	chemokine (C-C motif) receptor 8	NM_005201	0,0137954	0,32346	0,0137954	1,110	1,110	rapid up vs Slow	0,150	6,443	2,538	6,443	0,338	1,193	0,251
47874	11762964_at	---	---	---	0,0138006	0,00339994	0,0138006	1,082	1,082	rapid up vs Slow	0,114	6,443	2,538	6,443	0,194	4,425	0,534
16926	11732016_x_at	TTC23L	tetratricopeptide repeat domain 23-like	NM_144725	0,0138861	0,0604484	0,0138861	1,078	1,078	rapid up vs Slow	0,109	6,431	2,536	6,431	0,177	2,395	0,263
47683	11762773_x_at	C6orf54	chromosome 6 open reading frame 54	NM_014354	0,0139145	0,544757	0,0139145	1,161	1,161	rapid up vs Slow	0,215	6,426	2,535	6,426	0,696	0,777	0,336
15612	11730702_a_at	PDE4C	phosphodiesterase 4C, cAMP-specific	NM_000923 /// NM_001098818 /// NM_001098819	0,0139589	0,0171752	0,0139589	1,119	1,119	rapid up vs Slow	0,163	6,420	2,534	6,420	0,396	3,274	0,808
33672	11748762_x_at	KLF5	Kruppel-like factor 5 (intestinal)	NM_001730	0,014038	0,0264045	0,014038	1,146	1,146	rapid up vs Slow	0,196	6,409	2,532	6,409	0,576	2,973	1,069
44559	11759649_x_at	CREB3L2	cAMP responsive element binding protein 3-like 2	NM_194071	0,0140907	0,647378	0,0140907	1,199	1,199	rapid up vs Slow	0,261	6,402	2,530	6,402	1,024	0,624	0,399
23035	11738125_a_at	SLC25A24	solute carrier family 25 (mitochondrial carrier; phosphate carrier), member	NM_013386 /// NM_213651	0,0141247	0,939403	0,0141247	1,096	1,096	rapid up vs Slow	0,132	6,397	2,529	6,397	0,262	0,196	0,032

			24														
15353	11730443_a_at	CCNJ	cyclin J	NM_001134375 /// NM_001134376 /// NM_019084	0,0141469	0,121277	0,0141469	1,084	1,084	rapid up vs Slow	0,117	6,394	2,529	6,394	0,204	1,907	0,243
37433	11752523_x_at	ST8SIA5	ST8 alpha-N-acetyl-neuraminide alpha-2,8-sialyltransferase 5	NM_013305	0,0141756	0,260231	0,0141756	1,142	1,142	rapid up vs Slow	0,192	6,390	2,528	6,390	0,553	1,356	0,469
9673	11724763_s_at	DEPDC6	DEP domain containing 6	NM_022783	0,014186	0,108441	0,014186	1,141	1,141	rapid up vs Slow	0,190	6,388	2,528	6,388	0,542	1,986	0,673
39550	11754640_a_at	EPHX2	epoxide hydrolase 2, cytoplasmic	NM_001979	0,0142161	0,230827	0,0142161	0,727	-1,376	rapid down vs Slow	-0,460	6,384	-2,527	6,384	3,172	1,444	2,870
45261	11760351_x_at	UBXN11	UBX domain protein 11	NM_001077262 /// NM_145345 /// NM_183008	0,0142704	0,0842446	0,0142704	1,139	1,139	rapid up vs Slow	0,188	6,377	2,525	6,377	0,528	2,163	0,716
158	11715248_s_at	CRISP2	cysteine-rich secretory protein 2	NM_001142407 /// NM_001142408 /// NM_001142417 /// NM_001142435 /// NM_003296	0,0143102	0,0135181	0,0143102	1,098	1,098	rapid up vs Slow	0,135	6,371	2,524	6,371	0,271	3,442	0,587
28653	11743743_a_at	ATP11A	ATPase, class VI, type 11A	NM_015205 /// NM_032189	0,0143186	0,228444	0,0143186	1,148	1,148	rapid up vs Slow	0,199	6,370	2,524	6,370	0,594	1,452	0,541
35266	11750356_a_at	FAM163A	family with sequence similarity 163, member A	NM_173509	0,0144059	0,00803504	0,0144059	1,100	1,100	rapid up vs Slow	0,137	6,358	2,522	6,358	0,283	3,810	0,679
23862	11738952_at	LCN9	lipocalin 9	NM_001001676	0,0144501	0,679522	0,0144501	1,106	1,106	rapid up vs Slow	0,146	6,352	2,520	6,352	0,317	0,578	0,116
40819	11755909_a_at	FAM154B	family with sequence similarity 154, member B	NM_001008226	0,0144704	0,00260869	0,0144704	1,085	1,085	rapid up vs Slow	0,117	6,349	2,520	6,349	0,205	4,616	0,597
22250	11737340_at	DBF4B	DBF4 homolog B (S. cerevisiae)	NM_025104 /// NM_145663 /// NR_036623	0,0144732	0,526415	0,0144732	1,120	1,120	rapid up vs Slow	0,164	6,349	2,520	6,349	0,403	0,806	0,205
11480	11726570_at	SPOCK3	sparc/osteonectin, cwcv and kazal-like domains proteoglycan (testican) 3	NM_001040159 /// NM_016950	0,0146008	0,0331381	0,0146008	1,085	1,085	rapid up vs Slow	0,117	6,332	2,516	6,332	0,206	2,815	0,367
46536	11761626_x_at	RFC4	replication factor C (activator 1) 4, 37kDa	NM_002916 /// NM_181573	0,0146208	0,593607	0,0146208	1,108	1,108	rapid up vs Slow	0,148	6,329	2,516	6,329	0,327	0,702	0,145
7189	11722279_a_at	PHC3	polyhomeotic homolog 3 (Drosophila)	NM_024947	0,0146547	0,260209	0,0146547	1,104	1,104	rapid up vs Slow	0,143	6,324	2,515	6,324	0,305	1,356	0,261
29713	11744803_x_at	MDK	midkine (neurite growth-promoting factor 2)	NM_001012333 /// NM_001012334 /// NM_002391	0,0146566	0,178094	0,0146566	1,173	1,173	rapid up vs Slow	0,230	6,324	2,515	6,324	0,795	1,632	0,821
33095	11748185_	CCDC57	coiled-coil	NM_198082	0,014667	0,000330	0,014667	1,099	1,099	rapid up vs	0,136	6,323	2,514	6,323	0,277	6,152	1,079

	a_at		domain containing 57			876				Slow							
29256	11744346_a_at	MYO10	myosin X	NM_012334	0,0146769	0,346655	0,0146769	1,131	1,131	rapid up vs Slow	0,178	6,321	2,514	6,321	0,473	1,140	0,341
5613	11720703_at	MYL4	myosin, light chain 4, alkali; atrial, embryonic	NM_001002841 /// NM_002476	0,0147288	0,0289957	0,0147288	0,718	-1,393	rapid down vs Slow	-0,478	6,314	-2,513	6,314	3,421	2,908	6,301
39096	11754186_x_at	ST7L	suppression of tumorigenicity 7 like	NM_017744 /// NM_138727 /// NM_138728 /// NM_138729	0,0147317	0,0372504	0,0147317	1,094	1,094	rapid up vs Slow	0,130	6,314	2,513	6,314	0,252	2,733	0,436
18459	11733549_at	TEC	tec protein tyrosine kinase	NM_003215	0,0147533	0,58072	0,0147533	1,091	1,091	rapid up vs Slow	0,125	6,311	2,512	6,311	0,236	0,721	0,108
38259	11753349_a_at	NRG3	neuregulin 3	NM_001010848 /// NM_001165972 /// NM_001165973	0,0147584	0,320577	0,0147584	1,099	1,099	rapid up vs Slow	0,136	6,310	2,512	6,310	0,277	1,200	0,210
19808	11734898_at	IPP	intracisternal A particle-promoted polypeptide	NM_001145349 /// NM_005897	0,0147621	0,866857	0,0147621	0,815	-1,228	rapid down vs Slow	-0,296	6,310	-2,512	6,310	1,312	0,315	0,262
27608	11742698_at	SCG5	secretogranin V (7B2 protein)	NM_001144757 /// NM_003020	0,0148597	0,0540775	0,0148597	1,104	1,104	rapid up vs Slow	0,143	6,297	2,509	6,297	0,306	2,473	0,480
37838	11752928_x_at	PPIA	peptidylprolyl isomerase A (cyclophilin A)	NM_021130	0,0148775	0,228602	0,0148775	0,888	-1,126	rapid down vs Slow	-0,171	6,295	-2,509	6,295	0,440	1,451	0,406
25573	11740663_a_at	GXYLT1	glucoside xylosyltransferase 1	NM_001099650 /// NM_173601	0,0148809	0,275148	0,0148809	0,929	-1,076	rapid down vs Slow	-0,106	6,294	-2,509	6,294	0,169	1,314	0,141
14273	11729363_at	ZPBP	zona pellucida binding protein	NM_001159878 /// NM_007009	0,0150112	0,0356529	0,0150112	1,154	1,154	rapid up vs Slow	0,207	6,277	2,505	6,277	0,642	2,764	1,131
8569	11723659_a_at	UBIAD1	UbiA prenyltransferase domain containing 1	NM_013319	0,0150551	0,599511	0,0150551	0,891	-1,122	rapid down vs Slow	-0,166	6,271	-2,504	6,271	0,415	0,693	0,184
32024	11747114_a_at	PLK4	polo-like kinase 4	NM_001190799 /// NM_001190801 /// NM_014264	0,0150569	0,140604	0,0150569	1,126	1,126	rapid up vs Slow	0,171	6,271	2,504	6,271	0,438	1,802	0,503
5719	11720809_x_at	MZT2B	mitotic spindle organizing protein 2B	NM_025029	0,0151671	0,643378	0,0151671	0,867	-1,153	rapid down vs Slow	-0,205	6,257	-2,501	6,257	0,631	0,629	0,254
23074	11738164_at	C11orf42	chromosome 11 open reading frame 42	NM_173525	0,0151901	0,179861	0,0151901	1,118	1,118	rapid up vs Slow	0,161	6,254	2,501	6,254	0,386	1,625	0,402
36086	11751176_a_at	PRUNE2	prune homolog 2 (Drosophila)	NM_015225	0,0152134	0,00778493	0,0152134	1,100	1,100	rapid up vs Slow	0,137	6,251	2,500	6,251	0,281	3,832	0,688
38291	11753381_a_at	BTBD19 /// PLK3	BTB (POZ) domain containing 19 /// polo-like kinase 3	NM_001136537 /// NM_004073	0,0152153	0,249366	0,0152153	1,116	1,116	rapid up vs Slow	0,158	6,250	2,500	6,250	0,373	1,387	0,331
3133	11718223_a_at	GSPT1	G1 to S phase transition 1	NM_001130006 /// NM_001130007 /// NM_002094	0,0152459	0,0638808	0,0152459	0,674	-1,483	rapid down vs Slow	-0,569	6,247	-2,499	6,247	4,847	2,357	7,315
13461	11728551_	TULP1	tubby like protein	NM_003322	0,015311	0,131932	0,0153115	1,120	1,120	rapid up vs	0,163	6,238	2,498	6,238	0,400	1,847	0,474

	a_at		1		5					Slow							
18262	11733352_a_at	NF2	neurofibromin 2 (merlin)	NM_000268 /// NM_016418 /// NM_181825 /// NM_181828 /// NM_181829 /// NM_181830 /// NM_	0,0153308	0,199554	0,0153308	1,099	1,099	rapid up vs Slow	0,136	6,236	2,497	6,236	0,276	1,550	0,275
29569	11744659_a_at	CASC1	cancer susceptibility candidate 1	NM_001082972 /// NM_001082973 /// NM_018272	0,0153609	0,0348574	0,0153609	1,126	1,126	rapid up vs Slow	0,172	6,232	2,496	6,232	0,441	2,780	0,787
34308	11749398_a_at	B9D1	B9 protein domain 1	NM_015681	0,0154372	0,117416	0,0154372	1,106	1,106	rapid up vs Slow	0,146	6,222	2,494	6,222	0,318	1,929	0,395
2036	11717126_at	SH3PXD2A	SH3 and PX domains 2A	NM_014631	0,0155149	0,162947	0,0155149	1,152	1,152	rapid up vs Slow	0,205	6,212	2,492	6,212	0,627	1,696	0,685
34879	11749969_a_at	TSPAN5	tetraspanin 5	NM_005723	0,015521	0,0488358	0,015521	0,689	-1,451	rapid down vs Slow	-0,537	6,211	-2,492	6,211	4,320	2,544	7,078
22111	11737201_at	CSF1	colony stimulating factor 1 (macrophage)	NM_000757 /// NM_172210 /// NM_172211 /// NM_172212	0,0155337	0,227982	0,0155337	0,909	-1,100	rapid down vs Slow	-0,138	6,210	-2,492	6,210	0,284	1,453	0,266
40830	11755920_a_at	SLC22A14	solute carrier family 22, member 14	NM_004803	0,0155777	0,114849	0,0155777	1,124	1,124	rapid up vs Slow	0,169	6,204	2,491	6,204	0,428	1,945	0,537
7989	11723079_at	C2orf47	chromosome 2 open reading frame 47	NM_024520	0,015666	0,0644348	0,015666	1,113	1,113	rapid up vs Slow	0,154	6,193	2,489	6,193	0,357	2,351	0,541
48266	11763356_at	LOC157503	hypothetical protein LOC157503	---	0,0156925	0,0464765	0,0156925	1,128	1,128	rapid up vs Slow	0,174	6,190	2,488	6,190	0,456	2,579	0,759
19387	11734477_s_at	SPATA9	spermatogenesis associated 9	NM_031952	0,0157385	0,0712528	0,0157385	1,080	1,080	rapid up vs Slow	0,111	6,184	2,487	6,184	0,186	2,280	0,275
3288	11718378_s_at	PRKAG2	protein kinase, AMP-activated, gamma 2 non-catalytic subunit	NM_001040633 /// NM_016203 /// NM_024429	0,0157559	0,0553671	0,0157559	0,813	-1,230	rapid down vs Slow	-0,298	6,182	-2,486	6,182	1,333	2,457	2,118
10800	11725890_s_at	ABCC6 /// ABCC6P2	ATP-binding cassette, sub-family C (CFTR/MRP), member 6 /// ATP-binding cassette, sub-f	NM_001079528 /// NM_001171 /// NR_023387	0,015903	0,0386712	0,015903	1,109	1,109	rapid up vs Slow	0,150	6,164	2,483	6,164	0,336	2,707	0,590
12223	11727313_at	BHLHE22	basic helix-loop-helix family, member e22	NM_152414	0,0159354	0,0145452	0,0159354	1,107	1,107	rapid up vs Slow	0,147	6,160	2,482	6,160	0,324	3,391	0,713
33136	11748226_a_at	CCDC64	coiled-coil domain containing 64	NM_207311	0,0159523	0,0615522	0,0159523	1,129	1,129	rapid up vs Slow	0,174	6,158	2,481	6,158	0,456	2,383	0,706
48759	11763849_at	STYXL1	serine/threonine/tyrosine interacting-like 1	NM_016086	0,0159835	0,0286097	0,0159835	1,139	1,139	rapid up vs Slow	0,188	6,154	2,481	6,154	0,529	2,917	1,002
36854	11751944_a_at	COL22A1	collagen, type XXII, alpha 1	NM_152888	0,0160018	0,0287133	0,0160018	1,119	1,119	rapid up vs Slow	0,162	6,152	2,480	6,152	0,395	2,915	0,749
24610	11739700_	STON1	stonin 1	NM_0011985	0,016064	0,670121	0,0160648	1,100	1,100	rapid up vs	0,137	6,144	2,479	6,144	0,282	0,591	0,109

	a_at			95 /// NM_006873	8					Slow							
47520	11762610_ at	YIF1B	Yip1 interacting factor homolog B (S. cerevisiae)	NM_0010396 71 /// NM_0010396 72 /// NM_0010396 73 /// NM_0011454 61 /// NM_0011454 62 /// NM	0,016101 9	0,000323 235	0,0161019	1,123	1,123	rapid up vs Slow	0,168	6,139	2,478	6,139	0,421	6,170	1,693
5331	11720421_ at	AFF4	AF4/FMR2 family, member 4	NM_014423	0,016142 8	0,015446 1	0,0161428	1,147	1,147	rapid up vs Slow	0,198	6,134	2,477	6,134	0,585	3,349	1,276
39530	11754620_s _at	PKP4	plakophilin 4	NM_0010054 76 /// NM_003628	0,016180 6	0,064283 1	0,0161806	0,872	-1,147	rapid down vs Slow	-0,198	6,130	-2,476	6,130	0,586	2,352	0,900
8321	11723411_s _at	PLP1	proteolipid protein 1	NM_000533 /// NM_0011288 34 /// NM_199478	0,016184 3	0,466454	0,0161843	1,128	1,128	rapid up vs Slow	0,174	6,129	2,476	6,129	0,454	0,906	0,268
10404	11725494_ at	RSPH9	radial spoke head 9 homolog (Chlamydomona s)	NM_0011933 41 /// NM_152732	0,016193 2	0,501965	0,0161932	1,132	1,132	rapid up vs Slow	0,179	6,128	2,476	6,128	0,482	0,846	0,266
40650	11755740_ a_at	PRM2	protamine 2	NM_002762	0,016253 4	0,042091 7	0,0162534	1,089	1,089	rapid up vs Slow	0,123	6,121	2,474	6,121	0,225	2,648	0,390
8473	11723563_ at	VWA5A	von Willebrand factor A domain containing 5A	NM_0011301 42 /// NM_014622 /// NM_198315	0,016255 3	0,809854	0,0162553	1,124	1,124	rapid up vs Slow	0,169	6,121	2,474	6,121	0,426	0,397	0,111
18381	11733471_ at	PLEKHG 4B	pleckstrin homology domain containing, family G (with RhoGef domain) member 4B	NM_052909	0,016333 5	0,024202 1	0,0163335	0,893	-1,119	rapid down vs Slow	-0,163	6,112	-2,472	6,112	0,396	3,034	0,786
16636	11731726_ a_at	CASP5	caspase 5, apoptosis-related cysteine peptidase	NM_0011361 09 /// NM_0011361 10 /// NM_0011361 12 /// NM_004347 /// NR_024239 /// NR_03656	0,016474 1	0,75484	0,0164741	1,236	1,236	rapid up vs Slow	0,306	6,095	2,469	6,095	1,401	0,474	0,435
34770	11749860_ a_at	SMTN	smoothelin	NM_006932 /// NM_134269 /// NM_134270	0,016809 2	0,012388 1	0,0168092	1,114	1,114	rapid up vs Slow	0,156	6,055	2,461	6,055	0,363	3,504	0,840
35044	11750134_ a_at	DNMBP	dynamin binding protein	NM_015221	0,016811 1	0,594673	0,0168111	1,118	1,118	rapid up vs Slow	0,160	6,055	2,461	6,055	0,386	0,701	0,178
42118	11757208_s _at	C22orf34	chromosome 22 open reading frame 34	NR_026997	0,016822 8	0,924723	0,0168228	0,849	-1,178	rapid down vs Slow	-0,236	6,054	-2,460	6,054	0,835	0,223	0,123
37750	11752840_ a_at	ELL2	elongation factor, RNA polymerase II, 2	NM_012081	0,016839 8	0,127485	0,0168398	0,861	-1,161	rapid down vs Slow	-0,215	6,052	-2,460	6,052	0,694	1,871	0,858
29823	11744913_x _at	NFATC4	nuclear factor of activated T-cells, cytoplasmic,	NM_0011360 22 /// NM_004554	0,017019 9	0,016499 5	0,0170199	1,098	1,098	rapid up vs Slow	0,134	6,031	2,456	6,031	0,270	3,303	0,592

			calcineurin-dependent 4														
28672	11743762_at	SNIP1	Smad nuclear interacting protein 1	NM_024700	0,017022	0,0180444	0,017022	0,860	-1,162	rapid down vs Slow	-0,217	6,031	-2,456	6,031	0,704	3,240	1,513
21781	11736871_s_at	TRPC6	transient receptor potential cation channel, subfamily C, member 6	NM_004621	0,0170573	0,102987	0,0170573	1,084	1,084	rapid up vs Slow	0,116	6,027	2,455	6,027	0,202	2,022	0,271
31726	11746816_a_at	PIAS2	protein inhibitor of activated STAT, 2	NM_004671 /// NM_173206	0,0170864	0,164124	0,0170864	1,110	1,110	rapid up vs Slow	0,151	6,024	2,454	6,024	0,339	1,691	0,381
36072	11751162_x_at	TTYH1	tweety homolog 1 (Drosophila)	NM_001005367 /// NM_020659	0,0170937	0,00418538	0,0170937	1,107	1,107	rapid up vs Slow	0,147	6,023	2,454	6,023	0,323	4,275	0,916
24755	11739845_a_at	MSR1	macrophage scavenger receptor 1	NM_002445 /// NM_138715 /// NM_138716	0,0170947	0,951376	0,0170947	0,916	-1,092	rapid down vs Slow	-0,127	6,023	-2,454	6,023	0,241	0,173	0,028
10073	11725163_x_at	CYP39A1	cytochrome P450, family 39, subfamily A, polypeptide 1	NM_016593	0,0171309	0,834699	0,0171309	1,096	1,096	rapid up vs Slow	0,132	6,018	2,453	6,018	0,260	0,362	0,063
25972	11741062_s_at	NUMB	numb homolog (Drosophila)	NM_001005743 /// NM_001005744 /// NM_001005745 /// NM_003744	0,0171836	0,0693935	0,0171836	1,172	1,172	rapid up vs Slow	0,229	6,012	2,452	6,012	0,783	2,299	1,198
28658	11743748_a_at	LAT /// SPNS1	linker for activation of T cells /// spinster homolog 1 (Drosophila)	NM_001014987 /// NM_001014988 /// NM_001014989 /// NM_001142448 /// NM_001142449 /// NM	0,0172393	0,631833	0,0172393	0,824	-1,213	rapid down vs Slow	-0,279	6,006	-2,451	6,006	1,166	0,646	0,502
21423	11736513_s_at	NMT2	N-myristoyltransferase 2	NM_004808	0,0172591	0,0214766	0,0172591	0,802	-1,247	rapid down vs Slow	-0,319	6,004	-2,450	6,004	1,521	3,118	3,159
32492	11747582_x_at	NFATC4	nuclear factor of activated T-cells, cytoplasmic, calcineurin-dependent 4	NM_001136022 /// NM_004554	0,0173543	0,00262408	0,0173543	1,080	1,080	rapid up vs Slow	0,110	5,993	2,448	5,993	0,183	4,612	0,563
31291	11746381_x_at	PHLDB1	pleckstrin homology-like domain, family B, member 1	NM_001144758 /// NM_001144759 /// NM_015157	0,0173596	0,187721	0,0173596	1,105	1,105	rapid up vs Slow	0,144	5,993	2,448	5,993	0,311	1,594	0,331
31909	11746999_a_at	CDHR3	cadherin-related family member 3	NM_152750	0,0173979	0,182747	0,0173979	1,078	1,078	rapid up vs Slow	0,108	5,988	2,447	5,988	0,175	1,614	0,188
11700	11726790_a_at	CNTN2	contactin 2 (axonal)	NM_005076	0,0174123	0,335736	0,0174123	1,089	1,089	rapid up vs Slow	0,123	5,987	2,447	5,987	0,228	1,164	0,178
26158	11741248_a_at	PPP2R3A	protein phosphatase 2, regulatory subunit B'', alpha	NM_001190447 /// NM_002718 /// NM_181897	0,0175028	0,00364508	0,0175028	1,165	1,165	rapid up vs Slow	0,220	5,977	2,445	5,977	0,727	4,374	2,130
36230	11751320_	POLL	polymerase	NM_0011740	0,017503	0,076334	0,0175037	0,769	-1,300	rapid down	-0,378	5,977	-2,445	5,977	2,144	2,232	3,202

	a_at		(DNA directed), lambda	84 /// NM_0011740 85 /// NM_013274 /// NR_033406	7	4				vs Slow						
26437	11741527_ a_at	GAGE1 /// GAGE12 B /// GAGE12 C /// GAGE12 D /// GAGE12 E /// GAGE12 F /// GAGE12 G /// GAGE12 H /// GAGE12I /// GAGE12J /// GAGE13 /// GAGE2A /// GAGE2B /// GAGE2C /// GAGE2D /// GAGE2E /// GAGE4 /// GAGE5 /// GAGE6 /// GAGE7 /// GAGE8	G antigen 1 /// G antigen 12B /// G antigen 12C /// G antigen 12D /// G antigen 12E ///	NM_0010406 63 /// NM_0010984 05 /// NM_0010984 06 /// NM_0010984 07 /// NM_0010984 08 /// NM	0,017516 3	0,269444	0,0175163	1,078	1,078	rapid up vs Slow	0,109	5,975	2,444	5,975	0,177	1,330	0,158
37214	11752304_ a_at	ITGB4	integrin, beta 4	NM_000213 /// NM_0010056 19 /// NM_0010057 31	0,017541 2	0,197798	0,0175412	1,093	1,093	rapid up vs Slow	0,129	5,972	2,444	5,972	0,248	1,557	0,259
3809	11718899_ at	POLG	polymerase (DNA directed), gamma	NM_0011261 31 /// NM_002693	0,017647 8	0,047598 6	0,0176478	0,855	-1,170	rapid down vs Slow	-0,226	5,961	-2,441	5,961	0,767	2,562	1,319
27888	11742978_ at	CHMP4C	chromatin modifying protein 4C	NM_152284	0,017650 3	0,019239 1	0,0176503	1,141	1,141	rapid up vs Slow	0,190	5,960	2,441	5,960	0,544	3,195	1,166
19039	11734129_ at	SLC30A4	solute carrier family 30 (zinc transporter), member 4	NM_013309	0,017677 6	0,017678	0,0176776	1,092	1,092	rapid up vs Slow	0,127	5,957	2,441	5,957	0,243	3,254	0,531
48521	11763611_ a_at	GLI2	GLI family zinc finger 2	NM_005270	0,017692 6	0,217906	0,0176926	0,917	-1,090	rapid down vs Slow	-0,125	5,956	-2,440	5,956	0,234	1,486	0,233
2552	11717642_ a_at	CSTF3	cleavage stimulation factor, 3' pre-	NM_0010335 05 /// NM_0010335	0,017816 9	0,093601 5	0,0178169	0,851	-1,175	rapid down vs Slow	-0,233	5,942	-2,438	5,942	0,810	2,089	1,139

			RNA, subunit 3, 77kDa	06 /// NM_001326													
18561	11733651_x_at	LOC375190	hypothetical protein LOC375190	NM_001145710	0,0179175	0,108065	0,0179175	1,107	1,107	rapid up vs Slow	0,147	5,931	2,435	5,931	0,325	1,988	0,436
4200	11719290_at	TMEM63A	transmembrane protein 63A	NM_014698	0,0179581	0,572473	0,0179581	0,834	-1,200	rapid down vs Slow	-0,263	5,927	-2,434	5,927	1,033	0,734	0,512
9527	11724617_a_at	MOCS1	molybdenum cofactor synthesis 1	NM_001075098 /// NM_005943 /// NR_033233	0,0179808	0,00848851	0,0179808	1,089	1,089	rapid up vs Slow	0,124	5,924	2,434	5,924	0,229	3,771	0,583
9418	11724508_s_at	FGG	fibrinogen gamma chain	NM_000509 /// NM_021870	0,018026	0,0358365	0,018026	1,115	1,115	rapid up vs Slow	0,157	5,919	2,433	5,919	0,368	2,760	0,687
24959	11740049_x_at	ZNF461	zinc finger protein 461	NM_153257	0,0180741	0,140921	0,0180741	1,100	1,100	rapid up vs Slow	0,137	5,914	2,432	5,914	0,283	1,800	0,344
27658	11742748_at	SPPL3	signal peptide peptidase 3	NM_139015	0,0180887	0,0370734	0,0180887	0,853	-1,172	rapid down vs Slow	-0,229	5,913	-2,432	5,913	0,783	2,737	1,449
26797	11741887_a_at	WDR64	WD repeat domain 64	NM_144625	0,0180966	0,146368	0,0180966	1,132	1,132	rapid up vs Slow	0,179	5,912	2,431	5,912	0,482	1,773	0,578
15355	11730445_x_at	CCNJ	cyclin J	NM_001134375 /// NM_001134376 /// NM_019084	0,0181072	0,662439	0,0181072	1,088	1,088	rapid up vs Slow	0,122	5,911	2,431	5,911	0,223	0,602	0,091
44932	11760022_x_at	IFT172	intraflagellar transport 172 homolog (Chlamydomonas)	NM_015662	0,0181112	0,192052	0,0181112	1,147	1,147	rapid up vs Slow	0,197	5,910	2,431	5,910	0,584	1,578	0,623
22539	11737629_s_at	TLR6	toll-like receptor 6	NM_006068	0,0181116	0,0473758	0,0181116	1,131	1,131	rapid up vs Slow	0,177	5,910	2,431	5,910	0,472	2,566	0,819
20930	11736020_a_at	OPTN	optineurin	NM_001008211 /// NM_001008212 /// NM_001008213 /// NM_021980	0,0181217	0,000940761	0,0181217	0,737	-1,357	rapid down vs Slow	-0,440	5,909	-2,431	5,909	2,906	5,365	10,552
36720	11751810_x_at	GINS4	GINS complex subunit 4 (Sld5 homolog)	NM_032336	0,0181425	0,479924	0,0181425	1,100	1,100	rapid up vs Slow	0,137	5,907	2,430	5,907	0,281	0,883	0,168
2763	11717853_at	CCL21	chemokine (C-C motif) ligand 21	NM_002989	0,0181536	0,0411061	0,0181536	1,102	1,102	rapid up vs Slow	0,141	5,906	2,430	5,906	0,297	2,665	0,535
21857	11736947_a_at	ZNF334	zinc finger protein 334	NM_018102 /// NM_199441	0,0181659	0,378043	0,0181659	1,101	1,101	rapid up vs Slow	0,138	5,904	2,430	5,904	0,286	1,073	0,208
20542	11735632_at	KRT82	keratin 82	NM_033033	0,0182274	0,763958	0,0182274	1,081	1,081	rapid up vs Slow	0,112	5,898	2,429	5,898	0,188	0,461	0,059
14822	11729912_at	C1orf69	chromosome 1 open reading frame 69	NM_001010867	0,0182901	0,952925	0,0182901	0,891	-1,122	rapid down vs Slow	-0,166	5,891	-2,427	5,891	0,415	0,170	0,048
22644	11737734_x_at	CAPS2	calcyphosine 2	NM_032606	0,018406	0,478	0,018406	1,113	1,113	rapid up vs Slow	0,154	5,879	2,425	5,879	0,357	0,886	0,215
40709	11755799_a_at	TIE1	tyrosine kinase with immunoglobulin-like and EGF-like domains 1	NM_005424	0,0184211	5,16E-05	0,0184211	1,083	1,083	rapid up vs Slow	0,115	5,877	2,424	5,877	0,200	7,610	1,034
20430	11735520_a_at	FGF18	fibroblast growth factor 18	NM_003862	0,0184389	0,588756	0,0184389	1,111	1,111	rapid up vs Slow	0,151	5,875	2,424	5,875	0,344	0,709	0,166
25685	11740775_at	SOX1	SRY (sex determining region Y)-box 1	NM_005986	0,018449	0,119587	0,018449	1,093	1,093	rapid up vs Slow	0,129	5,874	2,424	5,874	0,248	1,916	0,323
46780	11761870_at	LOC100131642	PP5241	---	0,0184778	0,0766851	0,0184778	1,110	1,110	rapid up vs Slow	0,150	5,871	2,423	5,871	0,338	2,229	0,513

29567	11744657_x_at	FGFR4	fibroblast growth factor receptor 4	NM_002011 /// NM_022963 /// NM_213647	0,0184898	0,60512	0,0184898	1,085	1,085	rapid up vs Slow	0,118	5,870	2,423	5,870	0,208	0,685	0,097
23671	11738761_x_at	OR2M7	olfactory receptor, family 2, subfamily M, member 7	NM_001004691	0,0185119	0,0399123	0,0185119	1,118	1,118	rapid up vs Slow	0,161	5,868	2,422	5,868	0,387	2,685	0,708
13526	11728616_at	CARD6	caspase recruitment domain family, member 6	NM_032587	0,0185298	0,292653	0,0185298	1,119	1,119	rapid up vs Slow	0,162	5,866	2,422	5,866	0,392	1,268	0,339
7436	11722526_at	PTPN21	protein tyrosine phosphatase, non-receptor type 21	NM_007039	0,0185998	0,549673	0,0185998	1,101	1,101	rapid up vs Slow	0,139	5,858	2,420	5,858	0,288	0,769	0,151
33640	11748730_x_at	CDC14B	CDC14 cell division cycle 14 homolog B (S. cerevisiae)	NM_001077181 /// NM_003671 /// NM_033331	0,018668	0,245123	0,018668	1,091	1,091	rapid up vs Slow	0,125	5,851	2,419	5,851	0,235	1,400	0,225
44843	11759933_at	C22orf15	chromosome 22 open reading frame 15	NM_182520	0,0187027	0,119233	0,0187027	1,090	1,090	rapid up vs Slow	0,124	5,848	2,418	5,848	0,232	1,919	0,304
42078	11757168_at	SNORD97	small nucleolar RNA, C/D box 97	NR_004403	0,0187516	0,235652	0,0187516	0,905	-1,105	rapid down vs Slow	-0,144	5,843	-2,417	5,843	0,309	1,429	0,303
16606	11731696_a_at	MDH1B	malate dehydrogenase 1B, NAD (soluble)	NM_001039845	0,0187891	0,514976	0,0187891	1,094	1,094	rapid up vs Slow	0,130	5,839	2,416	5,839	0,253	0,824	0,143
22164	11737254_a_at	ZCWPW2	zinc finger, CW type with PWWP domain 2	NM_001040432	0,0188285	0,149486	0,0188285	1,070	1,070	rapid up vs Slow	0,098	5,835	2,416	5,835	0,143	1,758	0,172
16048	11731138_a_at	MYPN	myopalladin	NM_032578	0,0188328	0,011088	0,0188328	1,090	1,090	rapid up vs Slow	0,124	5,834	2,415	5,834	0,230	3,582	0,566
215	11715305_s_at	HOXA10	homeobox A10	NM_018951 /// NM_153715	0,0188377	0,0435101	0,0188377	1,093	1,093	rapid up vs Slow	0,129	5,834	2,415	5,834	0,249	2,625	0,448
45073	11760163_s_at	WSB1	WD repeat and SOCS box-containing 1	NM_015626 /// NM_134265	0,0188412	0,241833	0,0188412	0,898	-1,114	rapid down vs Slow	-0,155	5,833	-2,415	5,833	0,361	1,410	0,349
46530	11761620_at	---	---	---	0,0188753	0,101365	0,0188753	1,115	1,115	rapid up vs Slow	0,157	5,830	2,415	5,830	0,368	2,033	0,513
16802	11731892_a_at	MACROD2	MACRO domain containing 2	NM_001033087 /// NM_080676	0,0189159	0,0279908	0,0189159	0,878	-1,138	rapid down vs Slow	-0,187	5,826	-2,414	5,826	0,524	2,933	1,055
6535	11721625_s_at	GLUL	glutamate-ammonia ligase	NM_001033044 /// NM_001033056 /// NM_002065	0,0189398	0,719071	0,0189398	0,809	-1,236	rapid down vs Slow	-0,306	5,823	-2,413	5,823	1,401	0,523	0,504
41478	11756568_a_at	MEST	mesoderm specific transcript homolog (mouse)	NM_002402 /// NM_177524 /// NM_177525	0,0189601	0,000115874	0,0189601	1,143	1,143	rapid up vs Slow	0,192	5,821	2,413	5,821	0,554	6,966	2,650
34285	11749375_a_at	VAMP1	vesicle-associated membrane protein 1 (synaptobrevin 1)	NM_014231 /// NM_016830 /// NM_199245	0,0189839	0,899615	0,0189839	1,107	1,107	rapid up vs Slow	0,147	5,819	2,412	5,819	0,324	0,264	0,059
33167	11748257_a_at	NPRL3	nitrogen permease	NM_001039476 ///	0,0190953	0,0434435	0,0190953	0,670	-1,493	rapid down vs Slow	-0,578	5,807	-2,410	5,807	5,007	2,626	9,057

			regulator-like 3 (S. cerevisiae)	NM_001077350												
9486	11724576_at	MYCBPAP	MYCBP associated protein	NM_032133	0,0191107	3,65E-05	0,0191107	1,094	1,094	rapid up vs Slow	0,129	5,806	2,410	5,806	0,250	7,890	1,358
7220	11722310_a_at	TMC4	transmembrane channel-like 4	NM_001145303 /// NM_144686	0,019315	0,384772	0,019315	1,189	1,189	rapid up vs Slow	0,250	5,785	2,405	5,785	0,936	1,059	0,685
3639	11718729_at	CCDC97	coiled-coil domain containing 97	NM_052848	0,0193641	0,0267399	0,0193641	1,109	1,109	rapid up vs Slow	0,149	5,780	2,404	5,780	0,334	2,965	0,685
26660	11741750_a_at	FBXO9	F-box protein 9	NM_012347 /// NM_033480 /// NM_033481	0,0193778	0,433128	0,0193778	0,767	-1,304	rapid down vs Slow	-0,383	5,779	-2,404	5,779	2,204	0,966	1,473
40865	11755955_a_at	FAP	fibroblast activation protein, alpha	NM_004460	0,019518	0,276439	0,019518	1,137	1,137	rapid up vs Slow	0,185	5,765	2,401	5,765	0,513	1,311	0,466
45597	11760687_at	C18orf34	chromosome 18 open reading frame 34	NM_001105528 /// NM_198995	0,0195413	0,0117713	0,0195413	1,111	1,111	rapid up vs Slow	0,152	5,763	2,401	5,763	0,348	3,540	0,854
46331	11761421_at	CD70	CD70 molecule	NM_001252	0,0195489	0,0368927	0,0195489	1,107	1,107	rapid up vs Slow	0,146	5,762	2,400	5,762	0,319	2,740	0,607
4864	11719954_a_at	POLR3H	polymerase (RNA) III (DNA directed) polypeptide H (22.9kD)	NM_001018050 /// NM_001018052 /// NM_138338	0,0195552	0,267822	0,0195552	1,112	1,112	rapid up vs Slow	0,153	5,761	2,400	5,761	0,351	1,335	0,326
45450	11760540_at	C6orf138	chromosome 6 open reading frame 138	NM_001013732 /// NM_207499	0,0196122	0,0332093	0,0196122	1,094	1,094	rapid up vs Slow	0,129	5,756	2,399	5,756	0,251	2,813	0,491
30181	11745271_x_at	OPTN	optineurin	NM_001008211 /// NM_001008212 /// NM_001008213 /// NM_021980	0,0196582	0,000689691	0,0196582	0,754	-1,327	rapid down vs Slow	-0,408	5,751	-2,398	5,751	2,491	5,596	9,694
33134	11748224_x_at	AMDHD2	amidohydrolase domain containing 2	NM_001145815 /// NM_015944	0,0196773	0,14202	0,0196773	1,141	1,141	rapid up vs Slow	0,191	5,749	2,398	5,749	0,546	1,794	0,681
18576	11733666_a_at	C14orf118	chromosome 14 open reading frame 118	NM_017926 /// NM_017972	0,0198104	0,105448	0,0198104	0,922	-1,085	rapid down vs Slow	-0,118	5,736	-2,395	5,736	0,207	2,005	0,290
13269	11728359_at	TSPYL6	TSPY-like 6	NM_001003937	0,0198159	0,126103	0,0198159	1,134	1,134	rapid up vs Slow	0,181	5,736	2,395	5,736	0,492	1,879	0,644
45652	11760742_x_at	SYT8	synaptotagmin VIII	NM_138567	0,0198457	0,209233	0,0198457	1,097	1,097	rapid up vs Slow	0,134	5,733	2,394	5,733	0,269	1,516	0,285
7481	11722571_at	CCNA2	cyclin A2	NM_001237	0,0198517	0,0248293	0,0198517	0,907	-1,103	rapid down vs Slow	-0,141	5,732	-2,394	5,732	0,299	3,016	0,630
42407	11757497_s_at	CMPK1	cytidine monophosphate (UMP-CMP) kinase 1, cytosolic	NM_001136140 /// NM_016308	0,0198766	0,0130098	0,0198766	0,809	-1,236	rapid down vs Slow	-0,306	5,730	-2,394	5,730	1,403	3,469	3,398
42883	11757973_x_at	NMT2	N-myristoyltransferase 2	NM_004808	0,0200057	0,478621	0,0200057	0,805	-1,243	rapid down vs Slow	-0,313	5,717	-2,391	5,717	1,472	0,885	0,911
7405	11722495_a_at	MYBBP1A	MYB binding protein (P160) 1a	NM_001105538 /// NM_014520	0,0200384	0,577596	0,0200384	0,851	-1,174	rapid down vs Slow	-0,232	5,714	-2,390	5,714	0,806	0,726	0,410
288	11715378_x_at	TG	thyroglobulin	NM_003235	0,0200736	0,185853	0,0200736	1,103	1,103	rapid up vs Slow	0,141	5,711	2,390	5,711	0,299	1,602	0,336
26455	11741545_x_at	FBXO11	F-box protein 11	NM_001190274 ///	0,0202335	0,25705	0,0202335	1,120	1,120	rapid up vs Slow	0,163	5,696	2,387	5,696	0,398	1,365	0,381

				NM_025133													
45863	11760953_x_at	UBXN11	UBX domain protein 11	NM_001077262 /// NM_145345 /// NM_183008	0,0202394	0,145131	0,0202394	1,103	1,103	rapid up vs Slow	0,142	5,695	2,386	5,695	0,302	1,779	0,378
21894	11736984_a_at	UBASH3A	ubiquitin associated and SH3 domain containing A	NM_001001895 /// NM_018961	0,0202938	0,0237264	0,0202938	0,819	-1,221	rapid down vs Slow	-0,288	5,690	-2,385	5,690	1,244	3,048	2,666
23060	11738150_a_at	AVIL	advillin	NM_006576	0,0202951	0,0108243	0,0202951	0,833	-1,201	rapid down vs Slow	-0,264	5,690	-2,385	5,690	1,047	3,599	2,648
7574	11722664_a_at	GABRB3	gamma-aminobutyric acid (GABA) A receptor, beta 3	NM_000814 /// NM_001191320 /// NM_001191321 /// NM_021912	0,020349	0,0943536	0,020349	1,113	1,113	rapid up vs Slow	0,155	5,685	2,384	5,685	0,360	2,084	0,528
34760	11749850_a_at	RAB37	RAB37, member RAS oncogene family	NM_001006638 /// NM_001163989 /// NM_001163990 /// NM_175738	0,0203646	0,433437	0,0203646	1,137	1,137	rapid up vs Slow	0,186	5,683	2,384	5,683	0,517	0,965	0,351
29977	11745067_a_at	ODF2	outer dense fiber of sperm tails 2	NM_002540 /// NM_153432 /// NM_153433 /// NM_153435 /// NM_153436 /// NM_153437 /// NM_	0,020562	0,0718279	0,020562	0,821	-1,218	rapid down vs Slow	-0,285	5,665	-2,380	5,665	1,217	2,275	1,956
8661	11723751_at	TFF2	trefoil factor 2	NM_005423	0,0206239	0,158072	0,0206239	1,125	1,125	rapid up vs Slow	0,170	5,659	2,379	5,659	0,434	1,718	0,526
38301	11753391_a_at	CLEC4G	C-type lectin domain family 4, member G	NM_198492	0,0206284	0,0795737	0,0206284	1,129	1,129	rapid up vs Slow	0,176	5,658	2,379	5,658	0,462	2,203	0,719
31111	11746201_a_at	NAB1	NGFI-A binding protein 1 (EGR1 binding protein 1)	NM_005966	0,0206455	0,132441	0,0206455	1,116	1,116	rapid up vs Slow	0,159	5,657	2,378	5,657	0,379	1,844	0,494
30285	11745375_x_at	ZNF43	zinc finger protein 43	NM_003423	0,0206468	0,894878	0,0206468	1,088	1,088	rapid up vs Slow	0,121	5,657	2,378	5,657	0,221	0,272	0,042
26286	11741376_a_at	LRP8	low density lipoprotein receptor-related protein 8, apolipoprotein e receptor	NM_001018054 /// NM_004631 /// NM_017522 /// NM_033300	0,0207545	0,235566	0,0207545	1,137	1,137	rapid up vs Slow	0,185	5,647	2,376	5,647	0,514	1,429	0,520
48952	11764042_at	BLOC1S3	Biogenesis of lysosomal organelles complex-1, subunit 3	NM_212550	0,0207947	0,0206102	0,0207947	1,119	1,119	rapid up vs Slow	0,162	5,643	2,375	5,643	0,392	3,147	0,874
25274	11740364_a_at	PTPN3	protein tyrosine phosphatase, non-receptor type 3	NM_001145368 /// NM_001145369 /// NM_0011453	0,0208574	0,0389929	0,0208574	1,090	1,090	rapid up vs Slow	0,125	5,637	2,374	5,637	0,233	2,701	0,447

				70 /// NM_0011453 71 /// NM_0011453 72 /// NM													
30637	11745727_a_at	TCF12	transcription factor 12	NM_003205 /// NM_207036 /// NM_207037 /// NM_207038 /// NM_207040	0,0208954	0,823601	0,0208954	1,076	1,076	rapid up vs Slow	0,106	5,634	2,374	5,634	0,167	0,378	0,045
7624	11722714_a_at	STK35	serine/threonine kinase 35	NM_080836	0,0208969	0,0892357	0,0208969	1,109	1,109	rapid up vs Slow	0,150	5,633	2,373	5,633	0,336	2,123	0,506
38740	11753830_a_at	MLLT11	myeloid/lymphoid or mixed-lineage leukemia (trithorax homolog, Drosophila); translocate	NM_006818	0,0209786	0,760839	0,0209786	1,089	1,089	rapid up vs Slow	0,122	5,626	2,372	5,626	0,225	0,465	0,074
13518	11728608_x_at	NSUN5P2	NOP2/Sun domain family, member 5 pseudogene 2	NR_033323	0,0210878	0,0452029	0,0210878	0,821	-1,218	rapid down vs Slow	-0,285	5,616	-2,370	5,616	1,216	2,598	2,251
48090	11763180_x_at	UBE2F	ubiquitin-conjugating enzyme E2F (putative)	NM_080678	0,0211511	0,378879	0,0211511	0,813	-1,230	rapid down vs Slow	-0,298	5,610	-2,369	5,610	1,332	1,071	1,017
10290	11725380_at	POGLUT1	protein O-glucosyltransferase 1	NM_152305 /// NR_024265	0,0211584	0,0202295	0,0211584	0,837	-1,195	rapid down vs Slow	-0,257	5,609	-2,368	5,609	0,992	3,160	2,235
13330	11728420_a_at	CCDC134	coiled-coil domain containing 134	NM_024821	0,0212259	0,0236006	0,0212259	1,094	1,094	rapid up vs Slow	0,130	5,603	2,367	5,603	0,253	3,052	0,551
24118	11739208_x_at	DTNB	dystrobrevin, beta	NM_021907 /// NM_033147 /// NM_033148 /// NM_183360 /// NM_183361	0,0212462	0,0810658	0,0212462	1,153	1,153	rapid up vs Slow	0,206	5,601	2,367	5,601	0,634	2,190	0,992
36985	11752075_a_at	ABCA4	ATP-binding cassette, sub-family A (ABC1), member 4	NM_000350	0,0213079	0,096153	0,0213079	1,130	1,130	rapid up vs Slow	0,176	5,596	2,366	5,596	0,467	2,070	0,691
38485	11753575_at	COQ7	coenzyme Q7 homolog, ubiquinone (yeast)	NM_001190983 /// NM_016138	0,0214627	0,0665828	0,0214627	1,104	1,104	rapid up vs Slow	0,142	5,582	2,363	5,582	0,303	2,328	0,506
47946	11763036_x_at	ZNF761	zinc finger protein 761	NM_001008401	0,0214657	0,05727	0,0214657	1,145	1,145	rapid up vs Slow	0,195	5,582	2,363	5,582	0,570	2,433	0,994
35896	11750986_a_at	TNIP1	TNFAIP3 interacting protein 1	NM_006058	0,0214868	0,0199962	0,0214868	1,136	1,136	rapid up vs Slow	0,184	5,580	2,362	5,580	0,505	3,168	1,146
9746	11724836_at	NCEH1	neutral cholesterol ester hydrolase 1	NM_001146276 /// NM_001146277 /// NM_001146278 /// NM_020792	0,0214896	0,83033	0,0214896	0,892	-1,121	rapid down vs Slow	-0,165	5,580	-2,362	5,580	0,406	0,368	0,107

45582	11760672_at	AGGF1	angiogenic factor with G patch and FHA domains 1	NM_018046	0,0215129	0,133173	0,0215129	0,903	-1,108	rapid down vs Slow	-0,147	5,578	-2,362	5,578	0,325	1,840	0,429
25834	11740924_x_at	IMPDH2	IMP (inosine 5'-monophosphate) dehydrogenase 2	NM_000884	0,0215253	0,62085	0,0215253	0,847	-1,180	rapid down vs Slow	-0,239	5,576	-2,361	5,576	0,858	0,662	0,408
30165	11745255_a_at	FBXO16	F-box protein 16	NM_172366	0,0215541	0,634596	0,0215541	1,112	1,112	rapid up vs Slow	0,153	5,574	2,361	5,574	0,353	0,642	0,162
44930	11760020_x_at	PIGP	phosphatidylinositol glycan anchor biosynthesis, class P	NM_153681 /// NM_153682 /// NR_028352	0,0216981	0,0252284	0,0216981	0,853	-1,172	rapid down vs Slow	-0,229	5,561	-2,358	5,561	0,787	3,005	1,700
15742	11730832_at	RANBP10	RAN binding protein 10	NM_020850	0,021712	0,145715	0,021712	0,710	-1,408	rapid down vs Slow	-0,493	5,560	-2,358	5,560	3,645	1,776	4,657
7587	11722677_a_at	STEAP3	STEAP family member 3	NM_001008410 /// NM_018234 /// NM_182915	0,0217529	0,0832955	0,0217529	1,106	1,106	rapid up vs Slow	0,145	5,556	2,357	5,556	0,316	2,171	0,495
34081	11749171_a_at	UPB1	ureidopropionase, beta	NM_016327	0,0217717	0,584726	0,0217717	1,131	1,131	rapid up vs Slow	0,178	5,555	2,357	5,555	0,474	0,715	0,244
20596	11735686_a_at	ADH6	alcohol dehydrogenase 6 (class V)	NM_000672 /// NM_001102470	0,0219018	0,183852	0,0219018	1,067	1,067	rapid up vs Slow	0,093	5,543	2,354	5,543	0,130	1,610	0,150
32048	11747138_a_at	DCC	deleted in colorectal carcinoma	NM_005215	0,0219314	0,383479	0,0219314	1,102	1,102	rapid up vs Slow	0,140	5,540	2,354	5,540	0,294	1,062	0,226
48427	11763517_at	FILIP1L	Filamin A interacting protein 1-like	NM_001042459 /// NM_014890 /// NM_182909	0,0219437	0,33113	0,0219437	1,138	1,138	rapid up vs Slow	0,186	5,539	2,354	5,539	0,520	1,175	0,441
28803	11743893_x_at	ZNF780A	zinc finger protein 780A	NM_001010880 /// NM_001142577 /// NM_001142578 /// NM_001142579	0,0220312	0,391232	0,0220312	1,113	1,113	rapid up vs Slow	0,154	5,532	2,352	5,532	0,356	1,046	0,270
43323	11758413_s_at	ZC3H12B	zinc finger CCCH-type containing 12B	NM_001010888	0,0220521	0,491619	0,0220521	1,084	1,084	rapid up vs Slow	0,116	5,530	2,352	5,530	0,201	0,863	0,125
42140	11757230_at	NCRNA00157	non-protein coding RNA 157	NR_024354	0,0220562	0,182279	0,0220562	1,081	1,081	rapid up vs Slow	0,112	5,530	2,352	5,530	0,189	1,616	0,220
31335	11746425_x_at	ZSCAN2	zinc finger and SCAN domain containing 2	NM_001007072 /// NM_017894 /// NM_181877	0,0220593	0,00802806	0,0220593	1,087	1,087	rapid up vs Slow	0,120	5,529	2,351	5,529	0,216	3,810	0,595
33011	11748101_a_at	RUNX1T1	runt-related transcription factor 1; translocated to, 1 (cyclin D-related)	NM_001198625 /// NM_001198626 /// NM_001198627 /// NM_001198628 /// NM_001198629 /// NM	0,0221009	0,0237756	0,0221009	1,069	1,069	rapid up vs Slow	0,096	5,526	2,351	5,526	0,138	3,047	0,303
29805	11744895_a_at	FAM20A	family with sequence	NM_017565 /// NR_027751	0,0221043	0,609136	0,0221043	1,117	1,117	rapid up vs Slow	0,159	5,525	2,351	5,525	0,379	0,679	0,187

			similarity 20, member A														
7043	11722133_a_at	MGAT3	mannosyl (beta-1,4-)-glycoprotein beta-1,4-N-acetylglucosaminyltransferase	NM_001098270 /// NM_002409	0,0221361	0,00467982	0,0221361	1,137	1,137	rapid up vs Slow	0,186	5,523	2,350	5,523	0,516	4,195	1,569
24770	11739860_a_at	ITGA6	integrin, alpha 6	NM_000210 /// NM_001079818	0,0221559	0,00597419	0,0221559	0,819	-1,221	rapid down vs Slow	-0,288	5,521	-2,350	5,521	1,246	4,020	3,628
11314	11726404_x_at	IL17RC	interleukin 17 receptor C	NM_032732 /// NM_153460 /// NM_153461	0,022223	0,0341086	0,022223	1,115	1,115	rapid up vs Slow	0,157	5,515	2,348	5,515	0,371	2,795	0,752
28958	11744048_at	MAP1B	microtubule-associated protein 1B	NM_005909	0,0222262	0,0687442	0,0222262	1,113	1,113	rapid up vs Slow	0,155	5,515	2,348	5,515	0,359	2,306	0,601
10430	11725520_s_at	ICA1	islet cell autoantigen 1, 69kDa	NM_001136020 /// NM_004968 /// NM_022307	0,0222329	0,368671	0,0222329	1,113	1,113	rapid up vs Slow	0,154	5,514	2,348	5,514	0,355	1,092	0,281
21171	11736261_a_at	GABRG2	gamma-aminobutyric acid (GABA) A receptor, gamma 2	NM_000816 /// NM_198903 /// NM_198904	0,0222947	0,258497	0,0222947	1,154	1,154	rapid up vs Slow	0,206	5,509	2,347	5,509	0,637	1,361	0,629
30089	11745179_x_at	DCAF11	DDB1 and CUL4 associated factor 11	NM_001163484 /// NM_025230 /// NM_181357 /// NR_028099 /// NR_028100	0,0225344	0,703614	0,0225344	0,838	-1,194	rapid down vs Slow	-0,255	5,488	-2,343	5,488	0,976	0,545	0,388
45730	11760820_at	IDS	iduronate 2-sulfatase	NM_000202 /// NM_001166550 /// NM_006123	0,0225618	0,871808	0,0225618	1,075	1,075	rapid up vs Slow	0,104	5,486	2,342	5,486	0,163	0,308	0,037
21859	11736949_at	THEM5	thioesterase superfamily member 5	NM_182578	0,0226176	0,0600948	0,0226176	1,185	1,185	rapid up vs Slow	0,245	5,481	2,341	5,481	0,898	2,400	1,573
30918	11746008_x_at	NEXN	nexilin (F actin binding protein)	NM_001172309 /// NM_144573	0,0226701	0,525874	0,0226701	1,148	1,148	rapid up vs Slow	0,200	5,477	2,340	5,477	0,597	0,807	0,352
40265	11755355_s_at	RCSD1	RCSD domain containing 1	NM_052862	0,0226944	0,10091	0,0226944	0,846	-1,182	rapid down vs Slow	-0,241	5,475	-2,340	5,475	0,871	2,036	1,296
3274	11718364_a_at	ST6GALNAC6	ST6 (alpha-N-acetyl-neuraminyl-2,3-beta-galactosyl-1,3)-N-acetylgalactosaminide alpha-2	NM_013443	0,0227463	0,796153	0,0227463	1,097	1,097	rapid up vs Slow	0,134	5,471	2,339	5,471	0,270	0,416	0,082
20772	11735862_at	DLEU7	deleted in lymphocytic leukemia, 7	NM_198989	0,0227473	0,566714	0,0227473	1,111	1,111	rapid up vs Slow	0,152	5,470	2,339	5,470	0,347	0,743	0,188
14513	11729603_a_at	MCM7	minichromosome maintenance complex component 7	NM_005916 /// NM_182776	0,0227706	0,231266	0,0227706	0,869	-1,150	rapid down vs Slow	-0,202	5,469	-2,338	5,469	0,612	1,443	0,646
4952	11720042_a_at	RRP7A /// RRP7B	ribosomal RNA processing 7	NM_015703 /// NR_002184	0,0227975	0,0786745	0,0227975	1,281	1,281	rapid up vs Slow	0,357	5,466	2,338	5,466	1,914	2,211	3,097

			homolog A (S. cerevisiae) /// ribosomal RNA processing 7 hom														
2121	11717211_a_at	NEO1	neogenin 1	NM_001172623 /// NM_001172624 /// NM_002499	0,0228043	0,013388	0,0228043	1,100	1,100	rapid up vs Slow	0,138	5,466	2,338	5,466	0,284	3,449	0,717
26351	11741441_a_at	FGF13	fibroblast growth factor 13	NM_001139498 /// NM_001139500 /// NM_001139501 /// NM_001139502 /// NM_004114 /// NM_03	0,0228327	0,307396	0,0228327	1,113	1,113	rapid up vs Slow	0,154	5,463	2,337	5,463	0,357	1,231	0,322
23620	11738710_at	OR2AE1	olfactory receptor, family 2, subfamily AE, member 1	NM_001005276	0,0228337	0,00805674	0,0228337	1,093	1,093	rapid up vs Slow	0,128	5,463	2,337	5,463	0,246	3,808	0,686
15536	11730626_at	KLB	klotho beta	NM_175737	0,0228574	0,0499654	0,0228574	1,102	1,102	rapid up vs Slow	0,140	5,461	2,337	5,461	0,294	2,528	0,545
1485	11716575_a_at	CCDC6	coiled-coil domain containing 6	NM_005436	0,0228592	0,283334	0,0228592	1,099	1,099	rapid up vs Slow	0,136	5,461	2,337	5,461	0,277	1,293	0,263
34676	11749766_a_at	KCNH5	potassium voltage-gated channel, subfamily H (eag-related), member 5	NM_139318 /// NM_172375 /// NM_172376	0,0228882	0,0852468	0,0228882	1,145	1,145	rapid up vs Slow	0,195	5,459	2,336	5,459	0,569	2,155	0,899
28836	11743926_a_at	SEC24A	SEC24 family, member A (S. cerevisiae)	NM_021982	0,022948	0,146922	0,022948	1,073	1,073	rapid up vs Slow	0,101	5,454	2,335	5,454	0,154	1,770	0,199
35167	11750257_a_at	EEA1	early endosome antigen 1	NM_003566	0,0229621	0,114396	0,0229621	1,087	1,087	rapid up vs Slow	0,121	5,452	2,335	5,452	0,218	1,948	0,311
18248	11733338_x_at	FANCE	Fanconi anemia, complementation group E	NM_021922	0,0229837	0,394329	0,0229837	0,870	-1,150	rapid down vs Slow	-0,201	5,451	-2,335	5,451	0,607	1,040	0,463
8420	11723510_a_at	PLEKHH3	pleckstrin homology domain containing, family H (with MyTH4 domain) member 3	NM_024927	0,0230112	0,108732	0,0230112	1,174	1,174	rapid up vs Slow	0,231	5,448	2,334	5,448	0,800	1,984	1,165
21438	11736528_a_at	SMC2	structural maintenance of chromosomes 2	NM_001042550 /// NM_001042551 /// NM_006444	0,0231146	0,55822	0,0231146	0,909	-1,101	rapid down vs Slow	-0,138	5,440	-2,332	5,440	0,287	0,756	0,160
15383	11730473_at	PDE3B	phosphodiesterase 3B, cGMP-inhibited	NM_000922	0,0231187	0,0358412	0,0231187	0,841	-1,189	rapid down vs Slow	-0,249	5,439	-2,332	5,439	0,932	2,760	1,891
47597	11762687_x_at	MUC1	mucin 1, cell surface associated	NM_001018016 /// NM_001018017 /// NM_001044390 /// NM_001044391 ///	0,023154	0,0149857	0,023154	1,163	1,163	rapid up vs Slow	0,218	5,437	2,332	5,437	0,709	3,370	1,758

				NM_0010443 92 /// NM													
26881	11741971_at	KRT38	keratin 38	NM_006771	0,023158	0,00826491	0,023158	1,132	1,132	rapid up vs Slow	0,178	5,436	2,332	5,436	0,476	3,790	1,329
43588	11758678_s_at	ZNF423	zinc finger protein 423	NM_015069	0,0231966	0,480038	0,0231966	1,091	1,091	rapid up vs Slow	0,126	5,433	2,331	5,433	0,237	0,883	0,154
22896	11737986_s_at	ARHGAP11A /// ARHGAP11B	Rho GTPase activating protein 11A /// Rho GTPase activating protein 11B	NM_001039841 /// NM_014783 /// NM_199357	0,02321	0,173461	0,02321	1,114	1,114	rapid up vs Slow	0,156	5,432	2,331	5,432	0,363	1,651	0,442
34017	11749107_a_at	SLC4A11	solute carrier family 4, sodium borate transporter, member 11	NM_001174089 /// NM_001174090 /// NM_032034	0,023233	0,0160429	0,023233	1,102	1,102	rapid up vs Slow	0,141	5,430	2,330	5,430	0,296	3,322	0,724
33843	11748933_a_at	CCDC150	coiled-coil domain containing 150	NM_001080539	0,0233045	0,53577	0,0233045	1,084	1,084	rapid up vs Slow	0,117	5,424	2,329	5,424	0,204	0,791	0,119
22239	11737329_at	SLC26A4	solute carrier family 26, member 4	NM_000441	0,0233414	0,0457505	0,0233414	1,106	1,106	rapid up vs Slow	0,145	5,421	2,328	5,421	0,316	2,590	0,605
7458	11722548_at	HIVEP1	human immunodeficiency virus type I enhancer binding protein 1	NM_002114	0,0233764	0,729376	0,0233764	0,880	-1,136	rapid down vs Slow	-0,184	5,418	-2,328	5,418	0,507	0,509	0,191
11924	11727014_at	PAPPA	pregnancy-associated plasma protein A, pappalysin 1	NM_002581	0,0234064	0,0304585	0,0234064	1,090	1,090	rapid up vs Slow	0,125	5,416	2,327	5,416	0,233	2,874	0,494
3148	11718238_a_at	CAPRIN1	cell cycle associated protein 1	NM_005898 /// NM_203364	0,0235576	0,0379359	0,0235576	1,077	1,077	rapid up vs Slow	0,106	5,404	2,325	5,404	0,170	2,721	0,341
35138	11750228_x_at	RSAD1	radical S-adenosyl methionine domain containing 1	NM_018346	0,0235746	0,29089	0,0235746	1,090	1,090	rapid up vs Slow	0,124	5,402	2,324	5,402	0,230	1,273	0,217
15637	11730727_at	OMG	oligodendrocyte myelin glycoprotein	NM_002544	0,0237302	0,196434	0,0237302	1,112	1,112	rapid up vs Slow	0,153	5,390	2,322	5,390	0,350	1,562	0,405
39739	11754829_s_at	PI4KA /// PI4KAP1 /// PI4KAP2	phosphatidylinositol 4-kinase, catalytic, alpha /// phosphatidylinositol 4-kinase, cata	NM_002650 /// NM_058004 /// NR_003563 /// NR_003700	0,0237606	0,262594	0,0237606	0,880	-1,136	rapid down vs Slow	-0,185	5,387	-2,321	5,387	0,510	1,349	0,511
13480	11728570_at	ZFPM2	zinc finger protein, multitype 2	NM_012082	0,0237716	0,154546	0,0237716	1,083	1,083	rapid up vs Slow	0,115	5,386	2,321	5,386	0,197	1,734	0,253
4193	11719283_a_at	UBR7	ubiquitin protein ligase E3 component n-recognin 7 (putative)	NM_001100417 /// NM_175748	0,023814	0,1301	0,023814	0,899	-1,113	rapid down vs Slow	-0,154	5,383	-2,320	5,383	0,356	1,857	0,491
43294	11758384_s_at	CYP2U1	cytochrome P450, family 2, subfamily U, polypeptide 1	NM_183075	0,0238156	0,381442	0,0238156	0,903	-1,108	rapid down vs Slow	-0,148	5,383	-2,320	5,383	0,327	1,066	0,259
16938	11732028_at	AVP	arginine vasopressin	NM_000490	0,0238171	0,00443644	0,0238171	1,108	1,108	rapid up vs Slow	0,148	5,383	2,320	5,383	0,327	4,233	1,027
38282	11753372_x_at	CYP4F2	cytochrome P450, family 4,	NM_001082	0,0238706	0,0533968	0,0238706	1,091	1,091	rapid up vs Slow	0,126	5,378	2,319	5,378	0,237	2,482	0,437

			subfamily F, polypeptide 2														
37067	11752157_a_at	RAPGEF3	Rap guanine nucleotide exchange factor (GEF) 3	NM_001098531 /// NM_001098532 /// NM_006105	0,0238799	0,0111194	0,0238799	1,096	1,096	rapid up vs Slow	0,132	5,378	2,319	5,378	0,262	3,580	0,698
30008	11745098_a_at	SLC44A5	solute carrier family 44, member 5	NM_001130058 /// NM_152697	0,0239082	0,297411	0,0239082	1,107	1,107	rapid up vs Slow	0,147	5,375	2,318	5,375	0,323	1,256	0,302
1723	11716813_a_at	GATM	glycine amidinotransferase (L-arginine:glycine amidinotransferase)	NM_001482	0,0239167	0,658428	0,0239167	1,072	1,072	rapid up vs Slow	0,100	5,375	2,318	5,375	0,150	0,608	0,068
24357	11739447_a_at	PAK7	p21 protein (Cdc42/Rac)-activated kinase 7	NM_020341 /// NM_177990	0,0239502	0,0539204	0,0239502	1,137	1,137	rapid up vs Slow	0,186	5,372	2,318	5,372	0,516	2,475	0,951
24249	11739339_s_at	FAM46C	family with sequence similarity 46, member C	NM_017709	0,0239521	0,716872	0,0239521	0,615	-1,625	rapid down vs Slow	-0,700	5,372	-2,318	5,372	7,352	0,526	2,881
4722	11719812_a_at	SLC48A1	solute carrier family 48 (heme transporter), member 1	NM_017842	0,0240495	0,0343328	0,0240495	1,113	1,113	rapid up vs Slow	0,154	5,364	2,316	5,364	0,357	2,790	0,742
21768	11736858_a_at	ZNF182	zinc finger protein 182	NM_001007088 /// NM_001178099 /// NM_006962	0,0240678	0,00320456	0,0240678	1,127	1,127	rapid up vs Slow	0,173	5,363	2,316	5,363	0,446	4,467	1,486
27499	11742589_at	OR4L1	olfactory receptor, family 4, subfamily L, member 1	NM_001004717	0,0241177	0,00373689	0,0241177	1,152	1,152	rapid up vs Slow	0,205	5,359	2,315	5,359	0,628	4,356	2,042
39821	11754911_x_at	NEXN	nexilin (F actin binding protein)	NM_001172309 /// NM_144573	0,0241373	0,786366	0,0241373	1,169	1,169	rapid up vs Slow	0,225	5,357	2,315	5,357	0,761	0,430	0,244
19570	11734660_a_at	CLSTN3	calsyntenin 3	NM_014718	0,024187	0,00615565	0,024187	1,091	1,091	rapid up vs Slow	0,126	5,353	2,314	5,353	0,236	3,999	0,706
25024	11740114_a_at	CGN	cingulin	NM_020770	0,024187	0,0866594	0,024187	1,091	1,091	rapid up vs Slow	0,125	5,353	2,314	5,353	0,235	2,143	0,376
27307	11742397_at	ANP32C	acidic (leucine-rich) nuclear phosphoprotein 32 family, member C	NM_012403	0,0241907	0,271968	0,0241907	1,113	1,113	rapid up vs Slow	0,154	5,353	2,314	5,353	0,357	1,323	0,353
1454	11716544_at	TNNI1	troponin I type 1 (skeletal, slow)	NM_003281	0,0242351	7,35E-05	0,0242351	1,119	1,119	rapid up vs Slow	0,162	5,349	2,313	5,349	0,395	7,327	2,165
16855	11731945_a_at	WDR38	WD repeat domain 38	NM_001045476	0,0243134	0,0354179	0,0243134	1,129	1,129	rapid up vs Slow	0,175	5,343	2,312	5,343	0,459	2,768	0,952
16704	11731794_x_at	CDAN1	congenital dyserythropoietic anemia, type I	NM_138477	0,0243395	0,297029	0,0243395	0,897	-1,115	rapid down vs Slow	-0,158	5,341	-2,311	5,341	0,372	1,257	0,350
35139	11750229_x_at	TMEM25	transmembrane protein 25	NM_001144034 /// NM_001144035 /// NM_001144036 /// NM_001144037 /// NM_001144038 /// NM	0,0243455	0,125162	0,0243455	1,107	1,107	rapid up vs Slow	0,146	5,341	2,311	5,341	0,320	1,884	0,451

25191	11740281_at	TSEN2	tRNA splicing endonuclease 2 homolog (S. cerevisiae)	NM_001145392 /// NM_001145393 /// NM_001145394 /// NM_001145395 /// NM_025265	0,0243613	0,0157895	0,0243613	1,102	1,102	rapid up vs Slow	0,140	5,340	2,311	5,340	0,294	3,333	0,735
23971	11739061_at	SLC7A5	solute carrier family 7 (cationic amino acid transporter, y+ system), member 5	NM_003486	0,0243682	0,627921	0,0243682	0,867	-1,153	rapid down vs Slow	-0,205	5,339	-2,311	5,339	0,631	0,652	0,308
3672	11718762_s_at	POMP	proteasome maturation protein	NM_015932	0,0243694	0,0162171	0,0243694	1,070	1,070	rapid up vs Slow	0,098	5,339	2,311	5,339	0,143	3,315	0,356
25711	11740801_a_at	ACAD10	acyl-CoA dehydrogenase family, member 10	NM_001136538 /// NM_025247	0,0243951	0,934674	0,0243951	1,113	1,113	rapid up vs Slow	0,154	5,337	2,310	5,337	0,356	0,205	0,055
9015	11724105_a_at	C8orf44 /// SGK3	chromosome 8 open reading frame 44 /// serum/glucocorticoid regulated kinase family, me	NM_001033578 /// NM_013257 /// NM_019607 /// NM_170709	0,0244515	0,814093	0,0244515	1,104	1,104	rapid up vs Slow	0,143	5,332	2,309	5,332	0,306	0,391	0,090
8311	11723401_x_at	STK4	serine/threonine kinase 4	NM_006282	0,0245095	0,188329	0,0245095	0,907	-1,102	rapid down vs Slow	-0,141	5,328	-2,308	5,328	0,296	1,592	0,354
39051	11754141_x_at	FXYD6	FXYD domain containing ion transport regulator 6	NM_001164831 /// NM_001164832 /// NM_001164836 /// NM_001164837 /// NM_022003	0,0245365	0,0254756	0,0245365	1,112	1,112	rapid up vs Slow	0,154	5,326	2,308	5,326	0,353	2,998	0,796
1037	11716127_a_at	BAG1	BCL2-associated athanogene	NM_001172415 /// NM_004323	0,024604	0,00217551	0,024604	0,708	-1,412	rapid down vs Slow	-0,498	5,321	-2,307	5,321	3,712	4,748	13,249
29364	11744454_a_at	PARD3	par-3 partitioning defective 3 homolog (C. elegans)	NM_001184785 /// NM_001184786 /// NM_001184787 /// NM_001184788 /// NM_001184789 /// NM	0,0246378	0,0112534	0,0246378	1,106	1,106	rapid up vs Slow	0,146	5,318	2,306	5,318	0,318	3,572	0,853
22727	11737817_a_at	CDKL5	cyclin-dependent kinase-like 5	NM_001037343 /// NM_003159	0,0246435	0,0168095	0,0246435	1,089	1,089	rapid up vs Slow	0,124	5,318	2,306	5,318	0,229	3,289	0,567
12945	11728035_at	RS1	retinoschisin 1	NM_000330	0,0246754	0,491102	0,0246754	0,928	-1,077	rapid down vs Slow	-0,107	5,315	-2,305	5,315	0,173	0,864	0,112
20236	11735326_at	MYH15	myosin, heavy chain 15	NM_014981	0,0246867	0,112592	0,0246867	1,094	1,094	rapid up vs Slow	0,130	5,314	2,305	5,314	0,252	1,959	0,371
12636	11727726_a_at	DMRT2	doublesex and mab-3 related transcription factor 2	NM_001130865 /// NM_006557 /// NM_181872	0,0246905	0,101898	0,0246905	1,096	1,096	rapid up vs Slow	0,132	5,314	2,305	5,314	0,260	2,029	0,397
25332	11740422_x	HTR7	5-	NM_000872	0,024728	0,000282	0,0247282	1,095	1,095	rapid up vs	0,131	5,311	2,305	5,311	0,258	6,274	1,218

	_at		hydroxytryptamine (serotonin) receptor 7 (adenylate cyclase-coupled)	/// NM_019859 /// NM_019860	2	36				Slow							
24063	11739153_a_at	PSMD5	proteasome (prosome, macropain) 26S subunit, non-ATPase, 5	NM_005047	0,0247476	0,665099	0,0247476	1,094	1,094	rapid up vs Slow	0,129	5,310	2,304	5,310	0,251	0,599	0,113
32821	11747911_x_at	PKD1P1	polycystic kidney disease 1 (autosomal dominant) pseudogene 1	NR_036447	0,0249151	0,000717766	0,0249151	1,102	1,102	rapid up vs Slow	0,141	5,297	2,301	5,297	0,297	5,567	1,248
44493	11759583_at	C9orf102	chromosome 9 open reading frame 102	NM_001010895	0,0249221	0,778649	0,0249221	0,869	-1,150	rapid down vs Slow	-0,202	5,296	-2,301	5,296	0,612	0,441	0,204
10776	11725866_at	EBF3	early B-cell factor 3	NM_001005463	0,0249302	0,00507919	0,0249302	1,095	1,095	rapid up vs Slow	0,131	5,296	2,301	5,296	0,258	4,136	0,807
13095	11728185_at	ZSWIM5	zinc finger, SWIM-type containing 5	NM_020883	0,0249556	0,31856	0,0249556	1,145	1,145	rapid up vs Slow	0,195	5,294	2,301	5,294	0,572	1,204	0,521
7829	11722919_a_at	DOLPP1	dolichyl pyrophosphate phosphatase 1	NM_001135917 /// NM_020438	0,0250094	0,000938848	0,0250094	1,080	1,080	rapid up vs Slow	0,111	5,290	2,300	5,290	0,183	5,366	0,743
10422	11725512_a_at	SLC38A4	solute carrier family 38, member 4	NM_001143824 /// NM_018018	0,0250262	0,0986838	0,0250262	1,103	1,103	rapid up vs Slow	0,141	5,288	2,300	5,288	0,300	2,052	0,465
7994	11723084_x_at	SCN1B	sodium channel, voltage-gated, type I, beta	NM_001037 /// NM_199037	0,0250521	0,00347777	0,0250521	1,134	1,134	rapid up vs Slow	0,181	5,286	2,299	5,286	0,493	4,408	1,643
5517	11720607_a_at	SFRP2	secreted frizzled-related protein 2	NM_003013	0,0250602	0,00157731	0,0250602	0,745	-1,343	rapid down vs Slow	-0,425	5,286	-2,299	5,286	2,712	4,983	10,228
18472	11733562_a_at	KLHDC10	kelch domain containing 10	NM_014997	0,025113	0,27973	0,025113	1,097	1,097	rapid up vs Slow	0,133	5,282	2,298	5,282	0,266	1,302	0,262
20353	11735443_at	ZNF781	zinc finger protein 781	NM_152605	0,0251277	0,560634	0,0251277	1,105	1,105	rapid up vs Slow	0,144	5,281	2,298	5,281	0,311	0,752	0,177
45070	11760160_at	USP36	Ubiquitin specific peptidase 36	NM_025090	0,0251735	0,14788	0,0251735	0,821	-1,217	rapid down vs Slow	-0,284	5,277	-2,297	5,277	1,207	1,766	1,616
12532	11727622_a_at	VIT	vitrin	NM_001177969 /// NM_001177970 /// NM_001177971 /// NM_001177972 /// NM_053276	0,0252367	0,165518	0,0252367	1,103	1,103	rapid up vs Slow	0,141	5,272	2,296	5,272	0,299	1,685	0,382
17209	11732299_at	KIAA1715	KIAA1715	NM_030650	0,0252892	0,301756	0,0252892	1,076	1,076	rapid up vs Slow	0,106	5,268	2,295	5,268	0,169	1,245	0,160
5042	11720132_a_at	SPIRE1	spire homolog 1 (Drosophila)	NM_001128626 /// NM_001128627 /// NM_020148	0,0253118	0,0867214	0,0253118	1,088	1,088	rapid up vs Slow	0,122	5,267	2,295	5,267	0,224	2,143	0,364
39298	11754388_a_at	QSOX2	quiescin Q6 sulfhydryl oxidase 2	NM_181701	0,0253203	0,00074627	0,0253203	0,862	-1,160	rapid down vs Slow	-0,214	5,266	-2,295	5,266	0,687	5,537	2,889
22876	11737966_s_at	C15orf54	chromosome 15 open reading frame 54	NM_207445	0,025329	0,571877	0,025329	1,117	1,117	rapid up vs Slow	0,160	5,265	2,295	5,265	0,383	0,735	0,214
25312	11740402_at	C12orf69	chromosome 12 open reading frame 69	NM_001013698	0,0253451	0,0367175	0,0253451	0,916	-1,092	rapid down vs Slow	-0,127	5,264	-2,294	5,264	0,243	2,743	0,506

24248	11739338_at	FAM46C	family with sequence similarity 46, member C	NM_017709	0,0254328	0,785732	0,0254328	0,578	-1,729	rapid down vs Slow	-0,790	5,258	-2,293	5,258	9,359	0,431	3,068
18530	11733620_a_at	BCL11B	B-cell CLL/lymphoma 11B (zinc finger protein)	NM_022898 /// NM_138576	0,0255244	0,0664621	0,0255244	0,744	-1,345	rapid down vs Slow	-0,427	5,251	-2,291	5,251	2,738	2,329	4,859
14089	11729179_x_at	POLH	polymerase (DNA directed), eta	NM_006502	0,0256834	0,185913	0,0256834	0,901	-1,110	rapid down vs Slow	-0,151	5,239	-2,289	5,239	0,342	1,601	0,418
2226	11717316_x_at	RPL38	ribosomal protein L38	NM_000999 /// NM_001035258	0,0256927	0,433488	0,0256927	0,884	-1,131	rapid down vs Slow	-0,177	5,238	-2,289	5,238	0,471	0,965	0,347
15633	11730723_a_at	NCAM1	neural cell adhesion molecule 1	NM_000615 /// NM_001076682 /// NM_181351	0,0257167	0,0693564	0,0257167	1,203	1,203	rapid up vs Slow	0,267	5,236	2,288	5,236	1,064	2,299	1,869
33229	11748319_x_at	TP53	tumor protein p53	NM_000546 /// NM_001126112 /// NM_001126113 /// NM_001126114 /// NM_001126115 /// NM_00	0,0257256	0,202463	0,0257256	1,093	1,093	rapid up vs Slow	0,128	5,236	2,288	5,236	0,247	1,540	0,291
19789	11734879_a_at	TTLL2	tubulin tyrosine ligase-like family, member 2	NM_031949	0,0257645	0,688233	0,0257645	1,100	1,100	rapid up vs Slow	0,137	5,233	2,288	5,233	0,281	0,566	0,121
46188	11761278_at	FLT3LG	fms-related tyrosine kinase 3 ligand	NM_001459	0,0258007	0,549254	0,0258007	1,130	1,130	rapid up vs Slow	0,177	5,230	2,287	5,230	0,468	0,770	0,275
10648	11725738_at	37681	membrane-associated ring finger (C3HC4) 3	NM_178450	0,0258744	0,0177623	0,0258744	0,869	-1,151	rapid down vs Slow	-0,203	5,225	-2,286	5,225	0,618	3,251	1,538
47480	11762570_at	GPCRLTM7	putative olfactory receptor GPCRLTM7	NM_001195021	0,0258885	0,031494	0,0258885	1,106	1,106	rapid up vs Slow	0,146	5,224	2,286	5,224	0,318	2,850	0,694
33851	11748941_a_at	TOM1L2	target of myb1-like 2 (chicken)	NM_001033551 /// NM_001082968	0,0259776	0,079445	0,0259776	1,101	1,101	rapid up vs Slow	0,138	5,217	2,284	5,217	0,286	2,204	0,484
15072	11730162_s_at	SLC6A10P /// SLC6A8	solute carrier family 6 (neurotransmitter transporter, creatine), member 10 (pseudogene	NM_001142805 /// NM_001142806 /// NM_005629 /// NR_003083	0,0259857	0,387309	0,0259857	0,620	-1,614	rapid down vs Slow	-0,690	5,217	-2,284	5,217	7,142	1,054	5,773
21380	11736470_at	SLC35D1	solute carrier family 35 (UDP-glucuronic acid/UDP-N-acetylgalactosamine dual transporte	NM_015139	0,0260123	0,814552	0,0260123	0,807	-1,240	rapid down vs Slow	-0,310	5,215	-2,284	5,215	1,439	0,391	0,431
9978	11725068_x_at	CAMK2B	calcium/calmodulin-dependent protein kinase II beta	NM_001220 /// NM_172078 /// NM_172079	0,0260906	0,000647341	0,0260906	1,105	1,105	rapid up vs Slow	0,144	5,209	2,282	5,209	0,311	5,644	1,350

				/// NM_172080 /// NM_172081 /// NM_172082 /// NM_													
7012	11722102_a_at	TYRO3	TYRO3 protein tyrosine kinase	NM_006293	0,0261094	0,0722758	0,0261094	1,094	1,094	rapid up vs Slow	0,130	5,208	2,282	5,208	0,254	2,270	0,442
38686	11753776_x_at	LAIR2	leukocyte-associated immunoglobulin-like receptor 2	NM_002288 /// NM_021270	0,0261358	0,947104	0,0261358	1,310	1,310	rapid up vs Slow	0,390	5,206	2,282	5,206	2,275	0,182	0,317
39660	11754750_a_at	NLGN4X	neuroligin 4, X-linked	NM_020742 /// NM_181332	0,026186	0,0702215	0,026186	1,084	1,084	rapid up vs Slow	0,116	5,202	2,281	5,202	0,203	2,291	0,357
4144	11719234_at	RASL12	RAS-like, family 12	NM_016563	0,0263477	0,16963	0,0263477	1,114	1,114	rapid up vs Slow	0,156	5,190	2,278	5,190	0,364	1,667	0,468
14175	11729265_at	SPRR2D	small proline-rich protein 2D	NM_006945	0,0263801	0,141499	0,0263801	1,097	1,097	rapid up vs Slow	0,133	5,188	2,278	5,188	0,265	1,797	0,367
27349	11742439_s_at	ADAL	adenosine deaminase-like	NM_001012969 /// NM_001159280	0,0264263	0,183617	0,0264263	1,106	1,106	rapid up vs Slow	0,145	5,185	2,277	5,185	0,316	1,610	0,392
21305	11736395_a_at	BPGM	2,3-bisphosphoglycerate mutase	NM_001724 /// NM_199186	0,0264319	0,69105	0,0264319	0,638	-1,568	rapid down vs Slow	-0,648	5,184	-2,277	5,184	6,301	0,562	2,733
2419	11717509_at	IRF4	interferon regulatory factor 4	NM_001195286 /// NM_002460 /// NR_036585	0,0264722	0,617385	0,0264722	1,089	1,089	rapid up vs Slow	0,123	5,182	2,276	5,182	0,226	0,667	0,116
15248	11730338_a_at	SMAD6	SMAD family member 6	NM_001142861 /// NM_005585 /// NR_027654	0,0266584	0,167262	0,0266584	1,114	1,114	rapid up vs Slow	0,156	5,168	2,273	5,168	0,362	1,678	0,471
16182	11731272_a_at	KCNE1	potassium voltage-gated channel, Isk-related family, member 1	NM_000219 /// NM_001127668 /// NM_001127669 /// NM_001127670	0,0267033	0,00817288	0,0267033	1,131	1,131	rapid up vs Slow	0,177	5,165	2,273	5,165	0,470	3,798	1,383
14880	11729970_a_at	SPTB	spectrin, beta, erythrocytic	NM_000347 /// NM_001024858	0,0267914	0,112573	0,0267914	0,748	-1,336	rapid down vs Slow	-0,418	5,159	-2,271	5,159	2,618	1,959	3,977
43539	11758629_s_at	SPTBN1	spectrin, beta, non-erythrocytic 1	NM_003128 /// NM_178313	0,0268481	0,0157428	0,0268481	1,109	1,109	rapid up vs Slow	0,149	5,155	2,270	5,155	0,334	3,335	0,864
21570	11736660_a_at	MAP3K13	mitogen-activated protein kinase kinase kinase 13	NM_004721	0,0268481	0,368083	0,0268481	1,082	1,082	rapid up vs Slow	0,114	5,155	2,270	5,155	0,193	1,094	0,164
22809	11737899_a_at	TOX2	TOX high mobility group box family member 2	NM_001098796 /// NM_001098797 /// NM_001098798 /// NM_032883	0,0269278	0,092041	0,0269278	1,146	1,146	rapid up vs Slow	0,196	5,149	2,269	5,149	0,576	2,101	0,939
23293	11738383_a_at	IL22RA2	interleukin 22 receptor, alpha 2	NM_052962 /// NM_181309 /// NM_181310	0,0270228	0,070729	0,0270228	1,110	1,110	rapid up vs Slow	0,150	5,143	2,268	5,143	0,338	2,286	0,600

17117	11732207_a_at	GLRA2	glycine receptor, alpha 2	NM_001118885 /// NM_001118886 /// NM_001171942 /// NM_002063	0,0270632	0,409731	0,0270632	1,125	1,125	rapid up vs Slow	0,170	5,140	2,267	5,140	0,431	1,010	0,339
44579	11759669_x_at	CEP57	centrosomal protein 57kDa	NM_014679	0,0270951	0,107896	0,0270951	1,110	1,110	rapid up vs Slow	0,151	5,138	2,267	5,138	0,342	1,989	0,529
28394	11743484_at	NAA25	N(alpha)-acetyltransferase 25, NatB auxiliary subunit	NM_024953	0,0271141	0,345739	0,0271141	0,926	-1,080	rapid down vs Slow	-0,110	5,136	-2,266	5,136	0,183	1,142	0,163
18159	11733249_a_at	ARHGAP31	Rho GTPase activating protein 31	NM_020754	0,027151	0,119941	0,027151	1,152	1,152	rapid up vs Slow	0,204	5,134	2,266	5,134	0,625	1,914	0,932
45986	11761076_x_at	ACOT8	acyl-CoA thioesterase 8	NM_005469	0,0271588	0,306901	0,0271588	1,143	1,143	rapid up vs Slow	0,193	5,133	2,266	5,133	0,556	1,233	0,534
45499	11760589_a_at	RGS12	regulator of G-protein signaling 12	NM_002926 /// NM_198227 /// NM_198229	0,0271952	0,226868	0,0271952	1,137	1,137	rapid up vs Slow	0,185	5,131	2,265	5,131	0,512	1,457	0,581
13381	11728471_at	LPPR5	lipid phosphate phosphatase-related protein type 5	NM_001010861 /// NM_001037317	0,0272511	0,169144	0,0272511	1,076	1,076	rapid up vs Slow	0,106	5,127	2,264	5,127	0,168	1,669	0,219
23530	11738620_a_at	NLGN4Y	neuroligin 4, Y-linked	NM_001164238 /// NM_014893 /// NR_028318 /// NR_028319	0,0273013	0,150622	0,0273013	1,086	1,086	rapid up vs Slow	0,119	5,123	2,263	5,123	0,212	1,753	0,290
16142	11731232_a_at	TUSC5	tumor suppressor candidate 5	NM_172367	0,0273398	0,219871	0,0273398	1,109	1,109	rapid up vs Slow	0,149	5,120	2,263	5,120	0,333	1,480	0,385
22525	11737615_at	FAM194B	family with sequence similarity 194, member B	NM_182542	0,0273507	0,724267	0,0273507	1,115	1,115	rapid up vs Slow	0,158	5,120	2,263	5,120	0,372	0,516	0,150
11049	11726139_at	MTMR9	myotubularin related protein 9	NM_015458	0,0273541	0,0250612	0,0273541	0,845	-1,184	rapid down vs Slow	-0,243	5,120	-2,263	5,120	0,887	3,010	2,085
25756	11740846_s_at	C10orf71	chromosome 10 open reading frame 71	NM_001135196 /// NM_199459	0,0273943	0,236822	0,0273943	1,112	1,112	rapid up vs Slow	0,153	5,117	2,262	5,117	0,350	1,425	0,390
21249	11736339_a_at	PCSK1	proprotein convertase subtilisin/kexin type 1	NM_000439 /// NM_001177875 /// NM_001177876	0,0274575	0,0599801	0,0274575	1,087	1,087	rapid up vs Slow	0,121	5,112	2,261	5,112	0,218	2,401	0,409
20280	11735370_a_at	IQSEC2	IQ motif and Sec7 domain 2	NM_001111125 /// NM_015075 /// NR_024449	0,0274784	0,0586883	0,0274784	1,091	1,091	rapid up vs Slow	0,126	5,111	2,261	5,111	0,237	2,416	0,449
35294	11750384_a_at	APP	amyloid beta (A4) precursor protein	NM_000484 /// NM_001136129 /// NM_001136130 /// NM_201413 /// NM_201414	0,0275191	0,353179	0,0275191	1,081	1,081	rapid up vs Slow	0,112	5,108	2,260	5,108	0,189	1,126	0,166
1808	11716898_at	ARL3	ADP-ribosylation factor-like 3	NM_004311	0,0275275	0,0141612	0,0275275	1,096	1,096	rapid up vs Slow	0,133	5,108	2,260	5,108	0,264	3,410	0,706
32610	11747700_x	UBAP1	ubiquitin	NM_0011712	0,027538	0,582669	0,0275385	0,835	-1,197	rapid down	-0,260	5,107	-2,260	5,107	1,011	0,719	0,569

	_at		associated protein 1	01 /// NM_001171202 /// NM_001171203 /// NM_001171204 /// NM_016525 /// NR_03	5					vs Slow							
35209	11750299_a_at	IL13RA1	interleukin 13 receptor, alpha 1	NM_001560	0,0275687	0,0492622	0,0275687	1,220	1,220	rapid up vs Slow	0,287	5,105	2,259	5,105	1,232	2,538	2,451
35182	11750272_a_at	LARS	leucyl-tRNA synthetase	NM_020117	0,0275691	0,00889523	0,0275691	0,807	-1,239	rapid down vs Slow	-0,309	5,105	-2,259	5,105	1,430	3,738	4,189
25445	11740535_a_at	AARSD1	alanyl-tRNA synthetase domain containing 1	NM_001136042 /// NM_001142653 /// NM_001142654 /// NM_025267	0,027594	0,864356	0,027594	1,082	1,082	rapid up vs Slow	0,113	5,103	2,259	5,103	0,192	0,319	0,048
33601	11748691_a_at	KIAA0319L	KIAA0319-like	NM_024874	0,0275994	0,594553	0,0275994	1,122	1,122	rapid up vs Slow	0,166	5,103	2,259	5,103	0,415	0,701	0,228
48929	11764019_s_at	NAB1	NGFI-A binding protein 1 (EGR1 binding protein 1)	NM_005966	0,0276093	0,521788	0,0276093	0,741	-1,349	rapid down vs Slow	-0,432	5,102	-2,259	5,102	2,790	0,813	1,779
21360	11736450_x_at	GIN1	gypsy retrotransposon integrase 1	NM_017676	0,0276357	0,539471	0,0276357	0,932	-1,073	rapid down vs Slow	-0,101	5,100	-2,258	5,100	0,154	0,785	0,095
14836	11729926_at	ZNF510	zinc finger protein 510	NM_014930	0,0276918	0,170403	0,0276918	0,905	-1,105	rapid down vs Slow	-0,144	5,096	-2,258	5,096	0,312	1,664	0,408
48219	11763309_at	TCF4	transcription factor 4	NM_001083962 /// NM_003199	0,0278142	0,897691	0,0278142	1,144	1,144	rapid up vs Slow	0,194	5,088	2,256	5,088	0,562	0,268	0,118
42681	11757771_a_at	ABCC8	ATP-binding cassette, sub-family C (CFTR/MRP), member 8	NM_000352	0,0279695	0,0021447	0,0279695	1,078	1,078	rapid up vs Slow	0,108	5,077	2,253	5,077	0,174	4,758	0,652
22109	11737199_at	TMPRSS15	transmembrane protease, serine 15	NM_002772	0,0280116	0,362048	0,0280116	1,097	1,097	rapid up vs Slow	0,134	5,075	2,253	5,075	0,267	1,107	0,233
36994	11752084_a_at	KLHL6	kelch-like 6 (Drosophila)	NM_130446	0,0280221	0,43717	0,0280221	0,920	-1,086	rapid down vs Slow	-0,120	5,074	-2,253	5,074	0,214	0,958	0,162
19720	11734810_a_at	DTNA	dystrobrevin, alpha	NM_001128175 /// NM_001390 /// NM_001391 /// NM_001392 /// NM_032975 /// NM_032978 ///	0,0280567	0,0471489	0,0280567	1,107	1,107	rapid up vs Slow	0,147	5,072	2,252	5,072	0,325	2,569	0,658
22316	11737406_at	C2orf66	chromosome 2 open reading frame 66	NM_213608	0,0281232	0,00144082	0,0281232	1,107	1,107	rapid up vs Slow	0,147	5,067	2,251	5,067	0,323	5,050	1,288
27767	11742857_at	TXNDC17	thioredoxin domain containing 17	NM_032731	0,0281928	0,951029	0,0281928	0,877	-1,140	rapid down vs Slow	-0,190	5,062	-2,250	5,062	0,539	0,174	0,074
29555	11744645_a_at	TCN2	transcobalamin II	NM_000355 /// NM_001184726	0,0282122	0,014261	0,0282122	1,137	1,137	rapid up vs Slow	0,185	5,061	2,250	5,061	0,515	3,405	1,385

28585	11743675_x_at	ABL2	v-abl Abelson murine leukemia viral oncogene homolog 2	NM_001136000 /// NM_001136001 /// NM_001168236 /// NM_001168237 /// NM_001168238 /// NM	0,0282615	0,712217	0,0282615	0,854	-1,170	rapid down vs Slow	-0,227	5,058	-2,249	5,058	0,773	0,533	0,325
17146	11732236_a_at	GYPA	glycophorin A (MNS blood group)	NM_002099	0,0283022	0,411782	0,0283022	0,763	-1,311	rapid down vs Slow	-0,391	5,055	-2,248	5,055	2,290	1,006	1,823
27012	11742102_x_at	DPF1	D4, zinc and double PHD fingers family 1	NM_001135155 /// NM_001135156 /// NM_004647	0,0283417	0,0101569	0,0283417	1,141	1,141	rapid up vs Slow	0,190	5,053	2,248	5,053	0,542	3,644	1,564
1694	11716784_at	TRAK2	trafficking protein, kinesin binding 2	NM_015049	0,028348	0,928586	0,028348	0,665	-1,503	rapid down vs Slow	-0,588	5,052	-2,248	5,052	5,174	0,216	0,884
38218	11753308_s_at	RAET1G /// RAET1L /// ULBP2	retinoic acid early transcript 1G /// retinoic acid early transcript 1L /// UL16 bindin	NM_001001788 /// NM_025217 /// NM_130900	0,0283533	0,429755	0,0283533	1,112	1,112	rapid up vs Slow	0,153	5,052	2,248	5,052	0,351	0,972	0,270
9030	11724120_a_at	TRIM59	tripartite motif-containing 59	NM_173084	0,0284108	0,0440481	0,0284108	0,865	-1,156	rapid down vs Slow	-0,209	5,048	-2,247	5,048	0,655	2,616	1,358
29850	11744940_s_at	FAM108A1 /// FAM108A11P /// FAM108A4	family with sequence similarity 108, member A1 /// family with sequence similarity 108,	NM_001130111 /// NM_031213 /// XR_110836 /// XR_112007 /// XR_115090	0,0284334	0,402108	0,0284334	1,087	1,087	rapid up vs Slow	0,121	5,046	2,246	5,046	0,218	1,025	0,177
12858	11727948_a_at	RAB28	RAB28, member RAS oncogene family	NM_001017979 /// NM_001159601 /// NM_004249	0,0284382	0,00171299	0,0284382	0,858	-1,165	rapid down vs Slow	-0,220	5,046	-2,246	5,046	0,728	4,923	2,842
48475	11763565_x_at	ATP10B	ATPase, class V, type 10B	NM_025153	0,0284686	0,0270504	0,0284686	1,112	1,112	rapid up vs Slow	0,153	5,044	2,246	5,044	0,352	2,957	0,825
14809	11729899_a_at	C4BPB	complement component 4 binding protein, beta	NM_000716 /// NM_001017364 /// NM_001017365 /// NM_001017366 /// NM_001017367	0,0284803	0,0269056	0,0284803	1,079	1,079	rapid up vs Slow	0,109	5,043	2,246	5,043	0,178	2,960	0,419
13056	11728146_a_at	AQP11	aquaporin 11	NM_173039	0,0285091	0,20694	0,0285091	1,110	1,110	rapid up vs Slow	0,151	5,041	2,245	5,041	0,340	1,524	0,411
31000	11746090_a_at	SLK	STE20-like kinase	NM_014720	0,0285345	0,224555	0,0285345	0,891	-1,122	rapid down vs Slow	-0,166	5,040	-2,245	5,040	0,414	1,464	0,481
35745	11750835_a_at	PHACTR1	phosphatase and actin regulator 1	NM_030948	0,0285803	0,40953	0,0285803	1,070	1,070	rapid up vs Slow	0,098	5,037	2,244	5,037	0,144	1,010	0,115
15421	11730511_a_at	MAMSTR	MEF2 activating motif and SAP domain containing transcriptional regulator	NM_001130915 /// NM_182574	0,0286174	0,125673	0,0286174	1,104	1,104	rapid up vs Slow	0,143	5,034	2,244	5,034	0,306	1,881	0,457

20354	11735444_a_at	NEU3	sialidase 3 (membrane sialidase)	NM_006656	0,028626	0,0722711	0,028626	1,108	1,108	rapid up vs Slow	0,147	5,034	2,244	5,034	0,326	2,271	0,588
19785	11734875_a_at	PPFIBP1	PTPRF interacting protein, binding protein 1 (liprin beta 1)	NM_003622 /// NM_177444	0,0286323	0,101375	0,0286323	1,071	1,071	rapid up vs Slow	0,099	5,033	2,244	5,033	0,146	2,033	0,236
49388	AFFX-r2-TagIN-5_at	---	---	---	0,0286416	0,137833	0,0286416	1,114	1,114	rapid up vs Slow	0,156	5,033	2,243	5,033	0,365	1,816	0,527
21963	11737053_s_at	HSPD1	heat shock 60kDa protein 1 (chaperonin)	NM_002156 /// NM_199440	0,0286466	0,0246162	0,0286466	0,828	-1,208	rapid down vs Slow	-0,272	5,032	-2,243	5,032	1,110	3,022	2,666
22715	11737805_a_at	TMPRSS11D	transmembrane protease, serine 11D	NM_004262	0,0286815	0,131942	0,0286815	1,114	1,114	rapid up vs Slow	0,155	5,030	2,243	5,030	0,361	1,847	0,530
14155	11729245_a_at	GGCX	gamma-glutamyl carboxylase	NM_000821 /// NM_001142269	0,0286926	0,230245	0,0286926	1,079	1,079	rapid up vs Slow	0,110	5,029	2,243	5,029	0,181	1,446	0,208
31274	11746364_a_at	RNF207	ring finger protein 207	NM_207396	0,0287136	0,0397215	0,0287136	1,102	1,102	rapid up vs Slow	0,140	5,028	2,242	5,028	0,293	2,688	0,626
42198	11757288_s_at	CST3	cystatin C	NM_000099	0,028783	0,592427	0,028783	1,193	1,193	rapid up vs Slow	0,254	5,023	2,241	5,023	0,969	0,704	0,543
23438	11738528_a_at	IL31RA	interleukin 31 receptor A	NM_139017	0,0288084	0,123567	0,0288084	1,100	1,100	rapid up vs Slow	0,138	5,022	2,241	5,022	0,285	1,893	0,430
14930	11730020_a_at	ACSM2A	acyl-CoA synthetase medium-chain family member 2A	NM_001010845	0,0288098	0,0203214	0,0288098	1,112	1,112	rapid up vs Slow	0,153	5,022	2,241	5,022	0,352	3,157	0,886
783	11715873_a_at	OLFML3	olfactomedin-like 3	NM_020190	0,0288262	0,000866107	0,0288262	1,134	1,134	rapid up vs Slow	0,181	5,021	2,241	5,021	0,493	5,426	2,131
37934	11753024_a_at	NFIB	nuclear factor I/B	NM_001190737 /// NM_001190738 /// NM_005596	0,0288518	0,0429727	0,0288518	1,079	1,079	rapid up vs Slow	0,109	5,019	2,240	5,019	0,178	2,634	0,374
34488	11749578_a_at	COX10	COX10 homolog, cytochrome c oxidase assembly protein, heme A: farnesyltransferase (yeas	NM_001303	0,0288827	0,0241373	0,0288827	1,118	1,118	rapid up vs Slow	0,161	5,017	2,240	5,017	0,390	3,036	0,944
3134	11718224_s_at	GSPT1	G1 to S phase transition 1	NM_001130006 /// NM_001130007 /// NM_002094	0,0289023	0,1947	0,0289023	0,719	-1,390	rapid down vs Slow	-0,475	5,016	-2,240	5,016	3,382	1,568	4,230
8867	11723957_x_at	LSM5	LSM5 homolog, U6 small nuclear RNA associated (S. cerevisiae)	NM_001130710 /// NM_001139499 /// NM_012322 /// NR_024466	0,028952	0,565234	0,028952	0,919	-1,088	rapid down vs Slow	-0,122	5,012	-2,239	5,012	0,221	0,745	0,132
5584	11720674_a_at	PPM1A	protein phosphatase, Mg2+/Mn2+ dependent, 1A	NM_021003 /// NM_177951 /// NM_177952	0,0289704	0,55249	0,0289704	1,130	1,130	rapid up vs Slow	0,177	5,011	2,239	5,011	0,469	0,765	0,286
33638	11748728_a_at	CDC14B	CDC14 cell division cycle 14 homolog B (S. cerevisiae)	NM_001077181 /// NM_003671 ///	0,0290796	0,780462	0,0290796	1,130	1,130	rapid up vs Slow	0,176	5,004	2,237	5,004	0,464	0,438	0,162

				NM_033331													
7558	11722648_a_at	TNNI3	troponin I type 3 (cardiac)	NM_000363	0,0290946	0,0138514	0,0290946	1,107	1,107	rapid up vs Slow	0,146	5,003	2,237	5,003	0,320	3,425	0,877
29336	11744426_x_at	KIAA0101	KIAA0101	NM_001029989 /// NM_014736	0,0291236	0,0605715	0,0291236	0,861	-1,161	rapid down vs Slow	-0,215	5,001	-2,236	5,001	0,694	2,394	1,329
6610	11721700_a_at	RERG	RAS-like, estrogen-regulated, growth inhibitor	NM_001190726 /// NM_032918	0,029161	0,0646926	0,029161	1,068	1,068	rapid up vs Slow	0,094	4,999	2,236	4,999	0,134	2,348	0,251
2291	11717381_a_at	C16orf63	chromosome 16 open reading frame 63	NM_144600	0,0291885	0,00710686	0,0291885	0,819	-1,221	rapid down vs Slow	-0,288	4,997	-2,235	4,997	1,240	3,897	3,869
15082	11730172_a_at	GRM3	glutamate receptor, metabotropic 3	NM_000840	0,0292194	0,0699933	0,0292194	1,090	1,090	rapid up vs Slow	0,124	4,995	2,235	4,995	0,232	2,293	0,425
12010	11727100_a_at	NUMBL	numb homolog (Drosophila)-like	NM_004756	0,0293445	0,0398286	0,0293445	1,099	1,099	rapid up vs Slow	0,136	4,987	2,233	4,987	0,276	2,687	0,595
23520	11738610_at	OR10C1	olfactory receptor, family 10, subfamily C, member 1	NM_013941	0,0294123	0,196184	0,0294123	1,089	1,089	rapid up vs Slow	0,123	4,983	2,232	4,983	0,228	1,563	0,286
9531	11724621_a_at	WDR78	WD repeat domain 78	NM_024763 /// NM_207014	0,0294486	0,223801	0,0294486	1,105	1,105	rapid up vs Slow	0,144	4,980	2,232	4,980	0,311	1,467	0,366
14850	11729940_x_at	TMEM56	transmembrane protein 56	NM_152487	0,0294791	0,872663	0,0294791	0,786	-1,272	rapid down vs Slow	-0,348	4,978	-2,231	4,978	1,809	0,306	0,445
21426	11736516_a_at	SERAC1	serine active site containing 1	NM_032861	0,0295254	0,0417457	0,0295254	1,093	1,093	rapid up vs Slow	0,128	4,975	2,231	4,975	0,247	2,654	0,527
2469	11717559_a_at	ABLIM1	actin binding LIM protein 1	NM_001003407 /// NM_001003408 /// NM_002313 /// NM_006720	0,0295428	0,471602	0,0295428	0,789	-1,268	rapid down vs Slow	-0,342	4,974	-2,230	4,974	1,757	0,897	1,267
16718	11731808_at	CACNA1S	calcium channel, voltage-dependent, L type, alpha 1S subunit	NM_000069	0,0295482	0,00343613	0,0295482	1,099	1,099	rapid up vs Slow	0,136	4,974	2,230	4,974	0,276	4,417	0,980
48515	11763605_a_at	WNT11	wingless-type MMTV integration site family, member 11	NM_004626	0,0295505	0,0259861	0,0295505	1,136	1,136	rapid up vs Slow	0,184	4,974	2,230	4,974	0,510	2,985	1,224
23218	11738308_x_at	C8orf86	chromosome 8 open reading frame 86	NM_207412	0,0295568	0,276344	0,0295568	1,130	1,130	rapid up vs Slow	0,176	4,973	2,230	4,973	0,464	1,311	0,489
14281	11729371_a_at	ZBTB16	zinc finger and BTB domain containing 16	NM_001018011 /// NM_006006	0,0296316	0,718368	0,0296316	1,271	1,271	rapid up vs Slow	0,346	4,969	2,229	4,969	1,790	0,524	0,755
5250	11720340_a_at	STX1A	syntaxin 1A (brain)	NM_001165903 /// NM_004603	0,029672	0,00863115	0,029672	0,914	-1,094	rapid down vs Slow	-0,129	4,966	-2,228	4,966	0,250	3,759	0,757
25505	11740595_at	EDDM3B	epididymal protein 3B	NM_022360	0,029694	0,0164567	0,029694	1,093	1,093	rapid up vs Slow	0,128	4,965	2,228	4,965	0,245	3,304	0,652
25817	11740907_at	RTL1	retrotransposon-like 1	NM_001134888	0,0297224	0,00142194	0,0297224	1,093	1,093	rapid up vs Slow	0,129	4,963	2,228	4,963	0,249	5,059	1,014
40428	11755518_a_at	RNF165	ring finger protein 165	NM_152470	0,0297568	0,866798	0,0297568	1,131	1,131	rapid up vs Slow	0,178	4,961	2,227	4,961	0,475	0,315	0,121
14981	11730071_a_at	CNGA1	cyclic nucleotide gated channel alpha 1	NM_000087 /// NM_001142564	0,0297843	0,290634	0,0297843	1,066	1,066	rapid up vs Slow	0,092	4,959	2,227	4,959	0,128	1,274	0,131
39386	11754476_x	DNM1	dynamin 1	NM_0010053	0,029797	0,729355	0,0297977	1,095	1,095	rapid up vs	0,130	4,958	2,227	4,958	0,254	0,509	0,104

	_at			36 /// NM_004408	7					Slow							
2879	11717969_a_at	FGFR3	fibroblast growth factor receptor 3	NM_000142 /// NM_001163213 /// NM_022965	0,0298302	0,332749	0,0298302	1,092	1,092	rapid up vs Slow	0,127	4,956	2,226	4,956	0,241	1,171	0,228
22392	11737482_x_at	CDR1	cerebellar degeneration-related protein 1, 34kDa	NM_004065	0,0298421	0,181125	0,0298421	1,082	1,082	rapid up vs Slow	0,113	4,955	2,226	4,955	0,193	1,620	0,252
18853	11733943_at	IL2	interleukin 2	NM_000586	0,0298525	0,532825	0,0298525	1,118	1,118	rapid up vs Slow	0,161	4,955	2,226	4,955	0,387	0,796	0,249
19395	11734485_at	PRDM12	PR domain containing 12	NM_021619	0,0300012	0,0890047	0,0300012	1,097	1,097	rapid up vs Slow	0,133	4,945	2,224	4,945	0,266	2,125	0,458
15071	11730161_a_at	TEX14	testis expressed 14	NM_031272 /// NM_198393	0,0300119	0,214718	0,0300119	1,087	1,087	rapid up vs Slow	0,120	4,945	2,224	4,945	0,216	1,497	0,261
33499	11748589_a_at	EFCAB6	EF-hand calcium binding domain 6	NM_022785 /// NM_198856	0,0300242	0,197852	0,0300242	1,081	1,081	rapid up vs Slow	0,113	4,944	2,224	4,944	0,191	1,556	0,240
16432	11731522_a_at	ZNF592	zinc finger protein 592	NM_014630	0,0300276	0,3312	0,0300276	0,883	-1,132	rapid down vs Slow	-0,179	4,944	-2,223	4,944	0,482	1,175	0,458
40847	11755937_a_at	AKAP4	A kinase (PRKA) anchor protein 4	NM_003886 /// NM_139289	0,0300485	0,533653	0,0300485	1,118	1,118	rapid up vs Slow	0,161	4,942	2,223	4,942	0,389	0,794	0,250
45634	11760724_at	TBX3	T-box 3	NM_005996 /// NM_016569	0,0300566	0,123045	0,0300566	1,135	1,135	rapid up vs Slow	0,183	4,942	2,223	4,942	0,499	1,896	0,766
24513	11739603_x_at	BIRC5	baculoviral IAP repeat-containing 5	NM_001012270 /// NM_001012271 /// NM_001168	0,0300733	0,305372	0,0300733	1,100	1,100	rapid up vs Slow	0,138	4,941	2,223	4,941	0,285	1,236	0,286
41863	11756953_a_at	IL1R1	interleukin 1 receptor, type I	NM_000877	0,0300865	0,251412	0,0300865	1,105	1,105	rapid up vs Slow	0,143	4,940	2,223	4,940	0,308	1,381	0,345
12718	11727808_s_at	FECH	ferrochelatase	NM_000140 /// NM_001012515	0,0301155	0,717425	0,0301155	0,624	-1,603	rapid down vs Slow	-0,681	4,938	-2,222	4,938	6,943	0,525	2,955
33257	11748347_a_at	ANKRD28	ankyrin repeat domain 28	NM_001195098 /// NM_001195099 /// NM_015199	0,0302182	0,571208	0,0302182	1,082	1,082	rapid up vs Slow	0,113	4,932	2,221	4,932	0,193	0,736	0,115
48047	11763137_at	HSP90AA6P	heat shock protein 90kDa alpha (cytosolic), class A member 6 (pseudogene)	NR_036751	0,0302245	0,261269	0,0302245	1,097	1,097	rapid up vs Slow	0,134	4,931	2,221	4,931	0,268	1,353	0,294
43598	11758688_s_at	OLA1	Obg-like ATPase 1	NM_001011708 /// NM_013341	0,0302402	0,659178	0,0302402	0,841	-1,189	rapid down vs Slow	-0,250	4,930	-2,220	4,930	0,938	0,607	0,462
11102	11726192_at	TFAP2C	transcription factor AP-2 gamma (activating enhancer binding protein 2 gamma)	NM_003222	0,0302754	0,0216998	0,0302754	1,092	1,092	rapid up vs Slow	0,126	4,928	2,220	4,928	0,240	3,111	0,605
14632	11729722_a_at	PIAS2	protein inhibitor of activated STAT, 2	NM_004671 /// NM_173206	0,0302939	0,793281	0,0302939	1,081	1,081	rapid up vs Slow	0,113	4,927	2,220	4,927	0,190	0,420	0,065
26950	11742040_a_at	KIAA1543	KIAA1543	NM_001080429 /// NM_020902	0,0303218	0,00252399	0,0303218	1,144	1,144	rapid up vs Slow	0,195	4,925	2,219	4,925	0,567	4,640	2,138

13394	11728484_s_at	LOC100510742 /// LOC647859 /// OCLN	occludin-like /// occludin pseudogene /// occludin	NM_002538 /// NR_026578 /// XM_003118446 /// XM_003118447 /// XM_003118448 /// XM_00311	0,0303337	0,00701705	0,0303337	1,145	1,145	rapid up vs Slow	0,195	4,925	2,219	4,925	0,569	3,906	1,804
39486	11754576_a_at	LEAP2	liver expressed antimicrobial peptide 2	NM_052971	0,0304288	0,482536	0,0304288	1,095	1,095	rapid up vs Slow	0,131	4,919	2,218	4,919	0,257	0,878	0,184
22546	11737636_at	DOC2B	double C2-like domains, beta	NM_003585	0,0304309	0,038806	0,0304309	1,104	1,104	rapid up vs Slow	0,143	4,919	2,218	4,919	0,304	2,705	0,669
47958	11763048_at	GTSCR1	Gilles de la Tourette syndrome chromosome region, candidate 1	XM_496277 /// XM_936478	0,0305146	0,257455	0,0305146	0,908	-1,101	rapid down vs Slow	-0,139	4,914	-2,217	4,914	0,289	1,364	0,321
37932	11753022_a_at	CD84	CD84 molecule	NM_001184879 /// NM_001184881 /// NM_001184882 /// NM_003874	0,0305692	0,00859856	0,0305692	1,141	1,141	rapid up vs Slow	0,190	4,910	2,216	4,910	0,542	3,762	1,661
25971	11741061_a_at	SYBU	syntabulin (syntaxin-interacting)	NM_001099743 /// NM_001099744 /// NM_001099745 /// NM_001099746 /// NM_001099747 /// NM	0,0307228	0,0382455	0,0307228	1,103	1,103	rapid up vs Slow	0,142	4,901	2,214	4,901	0,300	2,715	0,665
46464	11761554_at	POLR2C	Polymerase (RNA) II (DNA directed) polypeptide C, 33kDa	NM_032940	0,0307481	0,0701534	0,0307481	1,129	1,129	rapid up vs Slow	0,175	4,899	2,213	4,899	0,458	2,291	0,857
22834	11737924_at	LECT2	leukocyte cell-derived chemotaxin 2	NM_002302	0,0307823	0,0134444	0,0307823	1,099	1,099	rapid up vs Slow	0,136	4,897	2,213	4,897	0,278	3,446	0,782
13184	11728274_a_at	PMP22	peripheral myelin protein 22	NM_000304 /// NM_153321 /// NM_153322	0,030798	0,115083	0,030798	1,101	1,101	rapid up vs Slow	0,139	4,896	2,213	4,896	0,288	1,944	0,458
21112	11736202_a_at	CDKAL1	CDK5 regulatory subunit associated protein 1-like 1	NM_017774	0,0308772	0,826625	0,0308772	1,091	1,091	rapid up vs Slow	0,125	4,891	2,212	4,891	0,234	0,373	0,072
42947	11758037_s_at	MTMR12	myotubularin related protein 12	NM_001040446	0,0308777	0,0126156	0,0308777	0,810	-1,235	rapid down vs Slow	-0,305	4,891	-2,212	4,891	1,391	3,491	3,970
15468	11730558_a_at	BCO2	beta-carotene oxygenase 2	NM_001037290 /// NM_031938	0,0309355	0,132693	0,0309355	1,085	1,085	rapid up vs Slow	0,117	4,888	2,211	4,888	0,207	1,843	0,311
13432	11728522_a_at	MYCL1	v-myc myelocytomatosis viral oncogene homolog 1, lung carcinoma	NM_001033081 /// NM_001033082 /// NM_005376	0,0309694	0,0389592	0,0309694	1,083	1,083	rapid up vs Slow	0,115	4,886	2,210	4,886	0,197	2,702	0,435

			derived (avian)													
5093	11720183_s_at	EEF1B2	eukaryotic translation elongation factor 1 beta 2	NM_001037663 /// NM_001959 /// NM_021121	0,0309713	0,159888	0,0309713	0,790	-1,265	rapid down vs Slow	-0,340	4,886	-2,210	4,886	1,729	1,710	2,420
29877	11744967_x_at	HSD11B1L	hydroxysteroid (11-beta) dehydrogenase 1-like	NM_198533 /// NM_198704 /// NM_198705 /// NM_198706 /// NM_198707 /// NM_198708	0,0309769	0,00495626	0,0309769	1,119	1,119	rapid up vs Slow	0,163	4,885	2,210	4,885	0,397	4,154	1,350
8554	11723644_a_at	PLN	phospholamban	NM_002667	0,0310808	0,434627	0,0310808	1,084	1,084	rapid up vs Slow	0,117	4,879	2,209	4,879	0,204	0,963	0,161
16649	11731739_a_at	PROX1	prospero homeobox 1	NM_002763	0,0311162	0,190633	0,0311162	1,165	1,165	rapid up vs Slow	0,220	4,877	2,208	4,877	0,725	1,583	0,941
23651	11738741_at	MYL10	myosin, light chain 10, regulatory	NM_138403	0,0311234	0,0120305	0,0311234	1,113	1,113	rapid up vs Slow	0,155	4,876	2,208	4,876	0,358	3,525	1,035
21130	11736220_x_at	C22orf25	chromosome 22 open reading frame 25	NM_152906	0,0311572	0,0658767	0,0311572	0,836	-1,196	rapid down vs Slow	-0,258	4,874	-2,208	4,874	0,997	2,335	1,912
24692	11739782_a_at	SNCA	synuclein, alpha (non A4 component of amyloid precursor)	NM_000345 /// NM_001146054 /// NM_001146055 /// NM_007308	0,0312153	0,395778	0,0312153	0,667	-1,498	rapid down vs Slow	-0,583	4,871	-2,207	4,871	5,101	1,037	4,345
22827	11737917_at	PDLIM2	PDZ and LIM domain 2 (mystique)	NM_021630 /// NM_176871 /// NM_198042	0,0312408	0,0480733	0,0312408	1,084	1,084	rapid up vs Slow	0,116	4,869	2,207	4,869	0,203	2,555	0,425
47375	11762465_x_at	LOC100271836 /// LOC440354 /// LOC595101 /// LOC641298 /// SLC7A5P1 /// SLC7A5P2	SMG1 homolog, phosphatidylinositol 3-kinase-related kinase pseudogene /// PI-3-kinase-r	NR_002453 /// NR_002473 /// NR_002593 /// NR_002594 /// NR_027154 /// NR_027155	0,0312712	0,0861861	0,0312712	1,081	1,081	rapid up vs Slow	0,112	4,868	2,206	4,868	0,187	2,147	0,331
14607	11729697_at	C2orf69	chromosome 2 open reading frame 69	NM_153689	0,0313284	0,350695	0,0313284	0,829	-1,207	rapid down vs Slow	-0,271	4,864	-2,205	4,864	1,102	1,131	1,025
41258	11756348_a_at	ERCC3	excision repair cross-complementing rodent repair deficiency, complementation group 3 (	NM_000122	0,0313459	0,189447	0,0313459	1,154	1,154	rapid up vs Slow	0,206	4,863	2,205	4,863	0,638	1,588	0,833
39804	11754894_a_at	KIAA1409	KIAA1409	NM_020818	0,0313549	0,316064	0,0313549	1,133	1,133	rapid up vs Slow	0,180	4,863	2,205	4,863	0,488	1,210	0,486
8061	11723151_s_at	C7orf10	chromosome 7 open reading frame 10	NM_001193311 /// NM_0011933	0,0315101	0,643439	0,0315101	1,093	1,093	rapid up vs Slow	0,129	4,853	2,203	4,853	0,249	0,629	0,129

				12 /// NM_001193313 /// NM_024728													
5686	11720776_a_at	RNF8	ring finger protein 8	NM_003958 /// NM_183078	0,0315287	0,0191092	0,0315287	1,074	1,074	rapid up vs Slow	0,103	4,852	2,203	4,852	0,160	3,200	0,422
31845	11746935_a_at	WARS2	tryptophanyl tRNA synthetase 2, mitochondrial	NM_015836 /// NM_201263	0,031537	0,0906617	0,031537	1,106	1,106	rapid up vs Slow	0,146	4,852	2,203	4,852	0,319	2,112	0,555
36042	11751132_a_at	TAF7L	TAF7-like RNA polymerase II, TATA box binding protein (TBP)-associated factor, 50kDa	NM_001168474 /// NM_024885	0,0315419	0,0911465	0,0315419	1,078	1,078	rapid up vs Slow	0,108	4,851	2,203	4,851	0,175	2,108	0,305
10762	11725852_at	ZNF319	zinc finger protein 319	NM_020807	0,0315641	0,593088	0,0315641	1,089	1,089	rapid up vs Slow	0,123	4,850	2,202	4,850	0,228	0,703	0,132
45441	11760531_at	MLL4	Myeloid/lymphoid or mixed-lineage leukemia 4	NM_014727	0,0315764	0,000890673	0,0315764	1,111	1,111	rapid up vs Slow	0,152	4,849	2,202	4,849	0,346	5,405	1,544
2461	11717551_a_at	LOXL1	lysyl oxidase-like 1	NM_005576	0,0315955	0,316842	0,0315955	1,091	1,091	rapid up vs Slow	0,125	4,848	2,202	4,848	0,236	1,208	0,235
36044	11751134_x_at	MAT2A	methionine adenosyltransferase II, alpha	NM_005911	0,0316484	0,0272027	0,0316484	1,100	1,100	rapid up vs Slow	0,137	4,845	2,201	4,845	0,281	2,953	0,684
23798	11738888_x_at	MICA	MHC class I polypeptide-related sequence A	NM_000247 /// NM_001177519 /// NR_036523 /// NR_036524 /// XM_003118562 /// XM_00311856	0,0317087	0,108285	0,0317087	1,141	1,141	rapid up vs Slow	0,190	4,842	2,200	4,842	0,540	1,987	0,887
44427	11759517_a_at	GRIP2	glutamate receptor interacting protein 2	NM_001080423	0,0317185	0,0693664	0,0317185	1,109	1,109	rapid up vs Slow	0,150	4,841	2,200	4,841	0,336	2,299	0,638
12069	11727159_a_at	LRP5	low density lipoprotein receptor-related protein 5	NM_002335	0,0317189	0,476614	0,0317189	1,087	1,087	rapid up vs Slow	0,120	4,841	2,200	4,841	0,216	0,888	0,159
32425	11747515_a_at	SLC38A2	solute carrier family 38, member 2	NM_018976	0,0317872	0,188046	0,0317872	1,113	1,113	rapid up vs Slow	0,154	4,837	2,199	4,837	0,357	1,593	0,471
29962	11745052_a_at	TMEM132B	transmembrane protein 132B	NM_052907	0,0318029	0,119679	0,0318029	1,123	1,123	rapid up vs Slow	0,168	4,836	2,199	4,836	0,422	1,916	0,668
37683	11752773_a_at	PAX4	paired box 4	NM_006193	0,0318209	0,121169	0,0318209	1,108	1,108	rapid up vs Slow	0,148	4,835	2,199	4,835	0,328	1,907	0,518
47988	11763078_a_at	---	---	---	0,0318293	0,229311	0,0318293	1,073	1,073	rapid up vs Slow	0,102	4,834	2,199	4,834	0,156	1,449	0,188
29689	11744779_a_at	FAM13A	family with sequence similarity 13, member A	NM_001015045 /// NM_014883	0,031849	0,0761117	0,031849	0,823	-1,216	rapid down vs Slow	-0,282	4,833	-2,198	4,833	1,189	2,234	2,198
42092	11757182_a_at	UHMK1	U2AF homology motif (UHM) kinase 1	NM_001184763 /// NM_144624 /// NM_175866	0,0319151	0,265767	0,0319151	0,818	-1,223	rapid down vs Slow	-0,291	4,829	-2,198	4,829	1,266	1,340	1,405
16716	11731806_a_at	CHRM3	cholinergic receptor, muscarinic 3	NM_000740	0,0319222	0,0183155	0,0319222	1,126	1,126	rapid up vs Slow	0,172	4,829	2,198	4,829	0,442	3,229	1,183

47885	11762975_at	TMEM64	transmembrane protein 64	NM_001008495 /// NM_001146273	0,0319259	0,0641818	0,0319259	1,100	1,100	rapid up vs Slow	0,138	4,829	2,197	4,829	0,284	2,354	0,554
32475	11747565_a_at	PPP2R4	protein phosphatase 2A activator, regulatory subunit 4	NM_001193397 /// NM_021131 /// NM_178000 /// NM_178001 /// NM_178003	0,0319612	0,00106371	0,0319612	1,096	1,096	rapid up vs Slow	0,132	4,827	2,197	4,827	0,260	5,274	1,137
6179	11721269_a_at	SCG3	secretogranin III	NM_001165257 /// NM_013243	0,0319648	0,023839	0,0319648	1,065	1,065	rapid up vs Slow	0,091	4,827	2,197	4,827	0,125	3,045	0,316
29845	11744935_a_at	LINGO2	leucine rich repeat and Ig domain containing 2	NM_152570	0,0319953	0,381067	0,0319953	1,086	1,086	rapid up vs Slow	0,120	4,825	2,197	4,825	0,214	1,067	0,190
35069	11750159_a_at	BATF2	basic leucine zipper transcription factor, ATF-like 2	NM_138456	0,0320163	0,450724	0,0320163	1,118	1,118	rapid up vs Slow	0,161	4,824	2,196	4,824	0,388	0,934	0,301
15004	11730094_x_at	FTCD	formiminotransferase cyclodeaminase	NM_006657 /// NM_206965	0,0320482	0,153349	0,0320482	1,124	1,124	rapid up vs Slow	0,169	4,822	2,196	4,822	0,428	1,740	0,617
31847	11746937_a_at	CTDSP2	CTD (carboxy-terminal domain, RNA polymerase II, polypeptide A) small phosphatase 2	NM_005730	0,0320523	0,206297	0,0320523	0,793	-1,261	rapid down vs Slow	-0,335	4,821	-2,196	4,821	1,679	1,526	2,126
10083	11725173_at	MN1	meningioma (disrupted in balanced translocation) 1	NM_002430	0,0320732	0,0336266	0,0320732	1,154	1,154	rapid up vs Slow	0,206	4,820	2,196	4,820	0,638	2,805	1,484
38280	11753370_s_at	FGFR2	fibroblast growth factor receptor 2	NM_000141 /// NM_001144913 /// NM_001144914 /// NM_001144915 /// NM_001144916 /// NM_00	0,0320975	0,0460229	0,0320975	1,091	1,091	rapid up vs Slow	0,125	4,819	2,195	4,819	0,236	2,586	0,506
5583	11720673_a_at	PPM1A	protein phosphatase, Mg2+/Mn2+ dependent, 1A	NM_021003 /// NM_177951 /// NM_177952	0,0321049	0,617676	0,0321049	0,751	-1,331	rapid down vs Slow	-0,412	4,818	-2,195	4,818	2,547	0,667	1,410
43079	11758169_s_at	HSPA5	heat shock 70kDa protein 5 (glucose-regulated protein, 78kDa)	NM_005347	0,0321486	0,0418912	0,0321486	0,837	-1,194	rapid down vs Slow	-0,256	4,816	-2,195	4,816	0,983	2,651	2,164
22071	11737161_a_at	CCNT2	cyclin T2	NM_001241 /// NM_058241	0,0322627	0,0977075	0,0322627	0,929	-1,077	rapid down vs Slow	-0,106	4,809	-2,193	4,809	0,170	2,059	0,290
38788	11753878_s_at	IL6ST	interleukin 6 signal transducer (gp130, oncostatin M	NM_001190981 /// NM_002184 /// NM_175767	0,0322907	0,354338	0,0322907	0,784	-1,276	rapid down vs Slow	-0,351	4,808	-2,193	4,808	1,847	1,123	1,726

			receptor)														
10636	11725726_a_at	C1orf56	chromosome 1 open reading frame 56	NM_017860	0,0323345	0,775434	0,0323345	1,137	1,137	rapid up vs Slow	0,185	4,805	2,192	4,805	0,512	0,445	0,190
5758	11720848_a_at	PHF6	PHD finger protein 6	NM_001015877 /// NM_032335 /// NM_032458	0,0323453	0,280729	0,0323453	1,090	1,090	rapid up vs Slow	0,124	4,804	2,192	4,804	0,231	1,299	0,250
48749	11763839_a_at	CYP2U1	cytochrome P450, family 2, subfamily U, polypeptide 1	NM_183075	0,0323454	0,0800533	0,0323454	1,081	1,081	rapid up vs Slow	0,113	4,804	2,192	4,804	0,191	2,199	0,350
43727	11758817_x_at	RRP7A	ribosomal RNA processing 7 homolog A (S. cerevisiae)	NM_015703	0,0323625	0,293589	0,0323625	1,229	1,229	rapid up vs Slow	0,298	4,803	2,192	4,803	1,327	1,266	1,399
29955	11745045_at	C1orf170	chromosome 1 open reading frame 170	NR_027693	0,0323863	0,00424211	0,0323863	1,104	1,104	rapid up vs Slow	0,143	4,802	2,191	4,802	0,307	4,265	1,089
14902	11729992_at	DQX1	DEAQ box RNA-dependent ATPase 1	NM_133637	0,0323869	0,057322	0,0323869	1,120	1,120	rapid up vs Slow	0,163	4,802	2,191	4,802	0,398	2,433	0,807
18743	11733833_a_at	MAP2	microtubule-associated protein 2	NM_001039538 /// NM_002374 /// NM_031845 /// NM_031847	0,0323923	0,0138968	0,0323923	1,117	1,117	rapid up vs Slow	0,160	4,802	2,191	4,802	0,382	3,423	1,088
33393	11748483_a_at	P2RX7	purinergic receptor P2X, ligand-gated ion channel, 7	NM_002562 /// NR_033948 /// NR_033949 /// NR_033950 /// NR_033951 /// NR_033952 /// NR_	0,0323931	0,718034	0,0323931	1,132	1,132	rapid up vs Slow	0,179	4,802	2,191	4,802	0,478	0,525	0,209
14330	11729420_a_at	TTC26	tetratricopeptide repeat domain 26	NM_001144920 /// NM_001144923 /// NM_024926	0,0324148	0,296587	0,0324148	1,116	1,116	rapid up vs Slow	0,159	4,800	2,191	4,800	0,377	1,258	0,395
32519	11747609_x_at	P2RX7	purinergic receptor P2X, ligand-gated ion channel, 7	NM_002562 /// NR_033948 /// NR_033949 /// NR_033950 /// NR_033951 /// NR_033952 /// NR_	0,0324298	0,904721	0,0324298	1,101	1,101	rapid up vs Slow	0,139	4,800	2,191	4,800	0,288	0,256	0,061
44777	11759867_at	C20orf152	chromosome 20 open reading frame 152	NM_080834	0,0324555	0,0712899	0,0324555	1,120	1,120	rapid up vs Slow	0,164	4,798	2,190	4,798	0,401	2,280	0,763
22755	11737845_x_at	FAM102A	family with sequence similarity 102, member A	NM_001035254 /// NM_203305	0,0324901	0,192548	0,0324901	0,786	-1,272	rapid down vs Slow	-0,347	4,796	-2,190	4,796	1,807	1,576	2,375
16407	11731497_a_at	ZNF134	zinc finger protein 134	NM_003435	0,0325264	0,0279897	0,0325264	0,901	-1,109	rapid down vs Slow	-0,150	4,794	-2,190	4,794	0,335	2,933	0,821
11453	11726543_at	TULP2	tubby like protein 2	NM_003323	0,0325434	0,101026	0,0325434	1,081	1,081	rapid up vs Slow	0,113	4,793	2,189	4,793	0,191	2,035	0,325
8327	11723417_s_at	HSP90AB1 /// HSP90AB3P	heat shock protein 90kDa alpha (cytosolic), class B member 1 /// heat shock protein 90k	NM_007355 /// NR_036694	0,0325502	0,113292	0,0325502	0,854	-1,170	rapid down vs Slow	-0,227	4,793	-2,189	4,793	0,772	1,955	1,259

28900	11743990_at	BRD4	bromodomain containing 4	NM_014299 /// NM_058243	0,0325892	0,0110547	0,0325892	0,806	-1,240	rapid down vs Slow	-0,310	4,790	-2,189	4,790	1,443	3,584	4,320
27053	11742143_s_at	RHCE /// RHD	Rh blood group, CcEe antigens /// Rh blood group, D antigen	NM_001127691 /// NM_016124 /// NM_020485 /// NM_138616 /// NM_138617 /// NM_138618	0,0325905	0,701855	0,0325905	0,781	-1,281	rapid down vs Slow	-0,357	4,790	-2,189	4,790	1,907	0,547	0,871
27360	11742450_a_at	COL13A1	collagen, type XIII, alpha 1	NM_001130103 /// NM_080798 /// NM_080800 /// NM_080801 /// NM_080802 /// NM_080805	0,0325918	0,0546631	0,0325918	1,136	1,136	rapid up vs Slow	0,184	4,790	2,189	4,790	0,509	2,466	1,048
1779	11716869_at	UTP18	UTP18, small subunit (SSU) processome component, homolog (yeast)	NM_016001	0,0327148	0,142489	0,0327148	1,103	1,103	rapid up vs Slow	0,141	4,783	2,187	4,783	0,298	1,792	0,446
13259	11728349_a_at	STIM2	stromal interaction molecule 2	NM_001169117 /// NM_001169118 /// NM_020860	0,0327376	0,0958406	0,0327376	1,168	1,168	rapid up vs Slow	0,223	4,782	2,187	4,782	0,748	2,073	1,297
16561	11731651_x_at	SNX15	sorting nexin 15	NM_013306 /// NM_147777	0,0328064	0,469303	0,0328064	0,844	-1,185	rapid down vs Slow	-0,245	4,778	-2,186	4,778	0,902	0,901	0,680
19742	11734832_at	GPR22	G protein-coupled receptor 22	NM_005295	0,0328081	0,174776	0,0328081	1,128	1,128	rapid up vs Slow	0,174	4,778	2,186	4,778	0,453	1,646	0,624
34802	11749892_a_at	RGS11	regulator of G-protein signaling 11	NM_003834 /// NM_183337	0,0328375	0,0229908	0,0328375	1,086	1,086	rapid up vs Slow	0,119	4,776	2,185	4,776	0,210	3,070	0,541
43072	11758162_s_at	FDX1	ferredoxin 1	NM_004109	0,0328893	0,92212	0,0328893	0,903	-1,108	rapid down vs Slow	-0,148	4,773	-2,185	4,773	0,328	0,227	0,062
22821	11737911_a_at	GPR84	G protein-coupled receptor 84	NM_020370	0,032924	0,550861	0,032924	1,224	1,224	rapid up vs Slow	0,292	4,771	2,184	4,771	1,275	0,767	0,820
42941	11758031_s_at	BHLHE41	basic helix-loop-helix family, member e41	NM_030762	0,0329787	0,534663	0,0329787	0,898	-1,113	rapid down vs Slow	-0,155	4,768	-2,184	4,768	0,359	0,793	0,239
6413	11721503_a_at	A4GALT	alpha 1,4-galactosyltransferase	NM_017436	0,0329909	0,276243	0,0329909	1,129	1,129	rapid up vs Slow	0,175	4,768	2,183	4,768	0,461	1,311	0,507
16380	11731470_at	AKT2	v-akt murine thymoma viral oncogene homolog 2	NM_001626	0,0330172	0,00419552	0,0330172	0,887	-1,127	rapid down vs Slow	-0,173	4,766	-2,183	4,766	0,447	4,273	1,603
14340	11729430_at	CCDC121	coiled-coil domain containing 121	NM_001142683 /// NM_024584	0,0330986	0,309201	0,0330986	1,067	1,067	rapid up vs Slow	0,093	4,761	2,182	4,761	0,130	1,227	0,134
25726	11740816_a_at	IL5RA	interleukin 5 receptor, alpha	NM_000564 /// NM_175724 ///	0,0331652	0,062371	0,0331652	1,090	1,090	rapid up vs Slow	0,125	4,758	2,181	4,758	0,233	2,374	0,464

				NM_175725 /// NM_175726 /// NM_175727 /// NM_175728												
5301	11720391_a_at	SLC39A10	solute carrier family 39 (zinc transporter), member 10	NM_001127257 /// NM_020342	0,0331674	0,0147607	0,0331674	0,755	-1,324	rapid down vs Slow	-0,405	4,758	-2,181	4,758	2,454	3,381	6,976
45544	11760634_at	ARHGEF10	Rho guanine nucleotide exchange factor (GEF) 10	NM_014629	0,0331697	0,587579	0,0331697	1,140	1,140	rapid up vs Slow	0,189	4,757	2,181	4,757	0,537	0,711	0,321
43993	11759083_at	RAB11FIP3	RAB11 family interacting protein 3 (class II)	NM_001142272 /// NM_014700	0,0331967	0,0225442	0,0331967	1,118	1,118	rapid up vs Slow	0,161	4,756	2,181	4,756	0,390	3,084	1,011
20351	11735441_a_at	ABCB5	ATP-binding cassette, sub-family B (MDR/TAP), member 5	NM_001163941 /// NM_001163942 /// NM_001163993 /// NM_178559	0,0332056	0,0464101	0,0332056	1,107	1,107	rapid up vs Slow	0,147	4,755	2,181	4,755	0,322	2,580	0,699
22923	11738013_a_at	CCDC7	coiled-coil domain containing 7	NM_001026383 /// NM_145023	0,0332509	0,894623	0,0332509	1,088	1,088	rapid up vs Slow	0,122	4,753	2,180	4,753	0,224	0,272	0,051
22931	11738021_a_at	PLCB4	phospholipase C, beta 4	NM_000933 /// NM_001172646 /// NM_182797	0,0332687	0,158496	0,0332687	1,088	1,088	rapid up vs Slow	0,122	4,752	2,180	4,752	0,222	1,716	0,321
36574	11751664_a_at	KCNIP2	Kv channel interacting protein 2	NM_014591 /// NM_173191 /// NM_173192 /// NM_173193 /// NM_173194 /// NM_173195 /// NM_	0,0332794	0,727879	0,0332794	1,082	1,082	rapid up vs Slow	0,114	4,751	2,180	4,751	0,196	0,511	0,084
17270	11732360_a_at	CTPS2	CTP synthase II	NM_001144002 /// NM_019857 /// NM_175859	0,033321	0,911345	0,033321	1,080	1,080	rapid up vs Slow	0,111	4,749	2,179	4,749	0,186	0,245	0,038
23891	11738981_x_at	CD4	CD4 molecule	NM_000616 /// NM_001195014 /// NM_001195015 /// NM_001195016 /// NM_001195017 /// NR_03	0,0333231	0,402167	0,0333231	1,091	1,091	rapid up vs Slow	0,126	4,749	2,179	4,749	0,238	1,025	0,205
29577	11744667_a_at	DENND5B	DENN/MADD domain containing 5B	NM_144973	0,0333457	0,0553872	0,0333457	1,103	1,103	rapid up vs Slow	0,141	4,748	2,179	4,748	0,298	2,456	0,616
45808	11760898_at	CCDC40	coiled-coil domain	NM_017950	0,0333667	0,429295	0,0333667	1,108	1,108	rapid up vs Slow	0,148	4,746	2,179	4,746	0,330	0,973	0,271

			containing 40														
226	11715316_x_at	HLA-C	major histocompatibility complex, class I, C	NM_002117 /// XM_003119244 /// XM_003119245 /// XM_003119246 /// XM_003119247 /// XM_00	0,0333701	0,573308	0,0333701	0,275	-3,636	rapid down vs Slow	-1,862	4,746	-2,179	4,746	51,959	0,733	32,085
12772	11727862_at	LIX1	Lix1 homolog (chicken)	NM_153234	0,0334392	0,0290356	0,0334392	0,926	-1,080	rapid down vs Slow	-0,112	4,742	-2,178	4,742	0,187	2,907	0,458
15598	11730688_a_at	FOXP3	forkhead box P3	NM_001114377 /// NM_014009	0,033477	0,188889	0,033477	1,098	1,098	rapid up vs Slow	0,134	4,740	2,177	4,740	0,271	1,590	0,363
43014	11758104_s_at	RAB2B	RAB2B, member RAS oncogene family	NM_001163380 /// NM_032846 /// NR_028074	0,0336855	0,696162	0,0336855	0,722	-1,386	rapid down vs Slow	-0,471	4,729	-2,175	4,729	3,318	0,555	1,558
39476	11754566_a_at	RNF217	ring finger protein 217	NM_152553	0,0337562	0,245151	0,0337562	1,079	1,079	rapid up vs Slow	0,110	4,725	2,174	4,725	0,180	1,400	0,214
14196	11729286_a_at	MSL3	male-specific lethal 3 homolog (Drosophila)	NM_001193270 /// NM_006800 /// NM_078628 /// NM_078629	0,033762	0,261523	0,033762	1,114	1,114	rapid up vs Slow	0,155	4,724	2,174	4,724	0,361	1,352	0,414
19010	11734100_x_at	ZNF578	zinc finger protein 578	NM_001099694	0,0337637	0,309471	0,0337637	1,088	1,088	rapid up vs Slow	0,122	4,724	2,174	4,724	0,224	1,226	0,233
39916	11755006_x_at	BCAR3	breast cancer anti-estrogen resistance 3	NM_003567	0,0337954	0,0303776	0,0337954	1,066	1,066	rapid up vs Slow	0,092	4,723	2,173	4,723	0,128	2,876	0,312
2718	11717808_s_at	EXD2	exonuclease 3'-5' domain containing 2	NM_001193360 /// NM_001193361 /// NM_001193362 /// NM_001193363 /// NM_018199 /// NR_03	0,0338137	0,0552187	0,0338137	0,904	-1,106	rapid down vs Slow	-0,145	4,722	-2,173	4,722	0,314	2,459	0,655
16285	11731375_a_at	CAMKK2	calcium/calmodulin-dependent protein kinase kinase 2, beta	NM_006549 /// NM_153499 /// NM_153500 /// NM_172214 /// NM_172215 /// NM_172216 /// NM_	0,0338249	0,0014714	0,0338249	1,111	1,111	rapid up vs Slow	0,151	4,721	2,173	4,721	0,343	5,034	1,464
13007	11728097_a_at	CROCC	ciliary rootlet coiled-coil, rootletin	NM_014675	0,033859	0,702819	0,033859	0,856	-1,168	rapid down vs Slow	-0,224	4,719	-2,172	4,719	0,752	0,546	0,348
24628	11739718_a_at	ZFP1	zinc finger protein 1 homolog (mouse)	NM_153688	0,0339396	0,302505	0,0339396	0,892	-1,121	rapid down vs Slow	-0,165	4,715	-2,171	4,715	0,408	1,243	0,430
14517	11729607_at	PGM2	phosphoglucomutase 2	NM_018290	0,0339556	0,151557	0,0339556	1,110	1,110	rapid up vs Slow	0,150	4,714	2,171	4,714	0,337	1,748	0,500
2534	11717624_	TBL2	transducin	NM_012453	0,03396	0,408593	0,03396	1,129	1,129	rapid up vs	0,175	4,714	2,171	4,714	0,459	1,012	0,394

	at		(beta)-like 2							Slow								
15073	11730163_x_at	SLC6A8	solute carrier family 6 (neurotransmitter transporter, creatine), member 8	NM_001142805 /// NM_001142806 /// NM_005629	0,0339626	0,14057	0,0339626	0,692	-1,445	rapid down vs Slow	-0,531	4,713	-2,171	4,713	4,221	1,802	6,454	
21173	11736263_x_at	SNX20	sorting nexin 20	NM_001144972 /// NM_153337 /// NM_182854	0,034028	0,129314	0,034028	1,097	1,097	rapid up vs Slow	0,134	4,710	2,170	4,710	0,267	1,861	0,423	
3013	11718103_a_at	RELA	v-rel reticuloendotheliosis viral oncogene homolog A (avian)	NM_001145138 /// NM_021975	0,0340315	0,887492	0,0340315	1,075	1,075	rapid up vs Slow	0,105	4,710	2,170	4,710	0,165	0,284	0,040	
38657	11753747_x_at	CHRAC1	chromatin accessibility complex 1	NM_017444 /// NR_023360	0,0340654	0,31869	0,0340654	0,913	-1,095	rapid down vs Slow	-0,131	4,708	-2,170	4,708	0,257	1,204	0,263	
11315	11726405_a_at	ASPA	aspartoacylase	NM_000049 /// NM_001128085	0,0340811	0,0694873	0,0340811	1,074	1,074	rapid up vs Slow	0,103	4,707	2,170	4,707	0,158	2,298	0,309	
23994	11739084_at	FKBP5	FK506 binding protein 5	NM_001145775 /// NM_001145776 /// NM_001145777 /// NM_004117	0,0341133	0,6126	0,0341133	1,108	1,108	rapid up vs Slow	0,148	4,705	2,169	4,705	0,330	0,674	0,189	
41383	11756473_a_at	CELF2	CUGBP, Elav-like family member 2	NM_001025076 /// NM_001025077 /// NM_001083591 /// NM_006561	0,0342028	0,00427131	0,0342028	0,808	-1,237	rapid down vs Slow	-0,307	4,700	-2,168	4,700	1,411	4,260	5,115	
21931	11737021_at	GRIN2B	glutamate receptor, ionotropic, N-methyl D-aspartate 2B	NM_000834	0,0342333	0,0016722	0,0342333	1,081	1,081	rapid up vs Slow	0,113	4,699	2,168	4,699	0,191	4,940	0,805	
23044	11738134_a_at	ZNF80	zinc finger protein 80	NM_007136	0,0342376	0,132145	0,0342376	1,082	1,082	rapid up vs Slow	0,113	4,698	2,168	4,698	0,192	1,846	0,302	
17647	11732737_at	NUP62CL	nucleoporin 62kDa C-terminal like	NM_017681 /// NR_033676	0,0342553	0,0140658	0,0342553	1,149	1,149	rapid up vs Slow	0,201	4,697	2,167	4,697	0,604	3,415	1,756	
14289	11729379_s_at	CASK	calcium/calmodulin-dependent serine protein kinase (MAGUK family)	NM_001126054 /// NM_001126055 /// NM_003688	0,0342918	0,750824	0,0342918	0,872	-1,147	rapid down vs Slow	-0,198	4,695	-2,167	4,695	0,587	0,479	0,240	
42644	11757734_x_at	APOA1	apolipoprotein A-I	NM_000039	0,0343368	0,0984411	0,0343368	0,930	-1,076	rapid down vs Slow	-0,105	4,693	-2,166	4,693	0,167	2,054	0,292	
21887	11736977_x_at	ZNF431	zinc finger protein 431	NM_133473	0,0343689	0,0649081	0,0343689	0,892	-1,121	rapid down vs Slow	-0,165	4,691	-2,166	4,691	0,409	2,346	0,817	
43525	11758615_s_at	FRMD4B	FERM domain containing 4B	NM_015123	0,034371	0,154094	0,034371	1,083	1,083	rapid up vs Slow	0,115	4,691	2,166	4,691	0,197	1,736	0,292	
16846	11731936_at	KCNN1	potassium intermediate/small conductance calcium-activated channel,	NM_002248	0,0343908	0,0312079	0,0343908	1,093	1,093	rapid up vs Slow	0,128	4,690	2,166	4,690	0,247	2,857	0,602	

			subfamily N, member														
42940	11758030_s_at	FOXG1	forkhead box G1	NM_005249	0,0344108	0,0701858	0,0344108	1,109	1,109	rapid up vs Slow	0,149	4,689	2,165	4,689	0,332	2,291	0,648
43732	11758822_at	KIF2A	kinesin heavy chain member 2A	NM_001098511 /// NM_004520	0,0344396	0,76893	0,0344396	0,874	-1,145	rapid down vs Slow	-0,195	4,687	-2,165	4,687	0,569	0,454	0,220
22267	11737357_a_at	CNGA3	cyclic nucleotide gated channel alpha 3	NM_001079878 /// NM_001298	0,0344963	0,0572186	0,0344963	1,120	1,120	rapid up vs Slow	0,164	4,684	2,164	4,684	0,402	2,434	0,835
13751	11728841_a_at	PLEKHA5	pleckstrin homology domain containing, family A member 5	NM_001143821 /// NM_001190860 /// NM_019012	0,0344974	0,134599	0,0344974	1,099	1,099	rapid up vs Slow	0,136	4,684	2,164	4,684	0,275	1,833	0,431
23190	11738280_x_at	KLK12	kallikrein-related peptidase 12	NM_019598 /// NM_145894 /// NM_145895	0,0345147	0,128975	0,0345147	1,093	1,093	rapid up vs Slow	0,129	4,683	2,164	4,683	0,248	1,863	0,395
4779	11719869_a_at	MYCN	v-myc myelocytomatosis viral related oncogene, neuroblastoma derived (avian)	NM_005378	0,0345162	0,165754	0,0345162	1,102	1,102	rapid up vs Slow	0,141	4,683	2,164	4,683	0,296	1,684	0,426
27077	11742167_at	BSX	brain-specific homeobox	NM_001098169	0,0345792	0,210695	0,0345792	1,122	1,122	rapid up vs Slow	0,167	4,680	2,163	4,680	0,416	1,511	0,537
17376	11732466_a_at	CXCL11	chemokine (C-X-C motif) ligand 11	NM_005409	0,0346164	0,179049	0,0346164	1,060	1,060	rapid up vs Slow	0,084	4,678	2,163	4,678	0,105	1,629	0,147
48713	11763803_a_at	TMEM67	transmembrane protein 67	NM_001142301 /// NM_153704 /// NR_024522	0,0346861	0,369474	0,0346861	1,088	1,088	rapid up vs Slow	0,122	4,674	2,162	4,674	0,223	1,091	0,209
35499	11750589_a_at	HFM1	HFM1, ATP-dependent DNA helicase homolog (S. cerevisiae)	NM_001017975	0,0347153	0,45067	0,0347153	1,120	1,120	rapid up vs Slow	0,163	4,673	2,162	4,673	0,399	0,934	0,319
34198	11749288_a_at	ST5	suppression of tumorigenicity 5	NM_005418 /// NM_139157 /// NM_213618	0,0347179	0,00467173	0,0347179	1,092	1,092	rapid up vs Slow	0,127	4,673	2,162	4,673	0,244	4,196	0,875
20733	11735823_at	ZP4	zona pellucida glycoprotein 4	NM_021186	0,034737	0,00644971	0,034737	1,098	1,098	rapid up vs Slow	0,135	4,671	2,161	4,671	0,274	3,966	0,930
7408	11722498_s_at	RPTOR	regulatory associated protein of MTOR, complex 1	NM_001163034 /// NM_020761	0,0347637	0,198281	0,0347637	1,115	1,115	rapid up vs Slow	0,158	4,670	2,161	4,670	0,372	1,555	0,496
16992	11732082_s_at	STOML3	stomatin (EPB72)-like 3	NM_001144033 /// NM_145286	0,0347691	0,0520585	0,0347691	1,132	1,132	rapid up vs Slow	0,179	4,670	2,161	4,670	0,479	2,500	1,025
17404	11732494_at	C1orf135	chromosome 1 open reading frame 135	NM_024037	0,0347944	0,00463524	0,0347944	1,073	1,073	rapid up vs Slow	0,101	4,668	2,161	4,668	0,154	4,202	0,554
32596	11747686_x_at	ARFIP2	ADP-ribosylation factor interacting protein 2	NM_012402	0,0348562	0,000779945	0,0348562	1,104	1,104	rapid up vs Slow	0,142	4,665	2,160	4,665	0,303	5,504	1,428
25832	11740922_a_at	C17orf80	chromosome 17 open reading frame 80	NM_001100621 /// NM_001100622 ///	0,0348719	0,369208	0,0348719	1,089	1,089	rapid up vs Slow	0,122	4,664	2,160	4,664	0,225	1,091	0,210

				NM_017941													
24012	11739102_a_at	SYF2	SYF2 homolog, RNA splicing factor (S. cerevisiae)	NM_015484 /// NM_207170	0,0349666	0,267782	0,0349666	1,084	1,084	rapid up vs Slow	0,117	4,659	2,159	4,659	0,205	1,335	0,235
22447	11737537_at	SAMD12	sterile alpha motif domain containing 12	NM_001101676 /// NM_207506	0,0350655	0,341365	0,0350655	1,092	1,092	rapid up vs Slow	0,127	4,654	2,157	4,654	0,243	1,152	0,241
34380	11749470_a_at	TRPM8	transient receptor potential cation channel, subfamily M, member 8	NM_024080	0,0351225	0,207291	0,0351225	1,078	1,078	rapid up vs Slow	0,109	4,651	2,157	4,651	0,178	1,523	0,233
20436	11735526_x_at	C3orf35	chromosome 3 open reading frame 35	NM_178339 /// NM_178342	0,0351355	0,0874555	0,0351355	1,096	1,096	rapid up vs Slow	0,132	4,650	2,156	4,650	0,260	2,137	0,478
32885	11747975_x_at	RNF145	ring finger protein 145	NM_144726	0,035166	0,0787645	0,035166	1,075	1,075	rapid up vs Slow	0,104	4,649	2,156	4,649	0,163	2,210	0,309
31248	11746338_a_at	RAB35	RAB35, member RAS oncogene family	NM_001167606 /// NM_006861	0,0351739	0,285456	0,0351739	1,098	1,098	rapid up vs Slow	0,135	4,648	2,156	4,648	0,272	1,287	0,301
8765	11723855_a_at	CASKIN2	CASK interacting protein 2	NM_001142643 /// NM_020753	0,0351798	0,0159556	0,0351798	1,090	1,090	rapid up vs Slow	0,124	4,648	2,156	4,648	0,232	3,326	0,663
32131	11747221_a_at	FBXO10	F-box protein 10	NM_012166	0,0352069	0,0179769	0,0352069	1,103	1,103	rapid up vs Slow	0,141	4,647	2,156	4,647	0,298	3,242	0,831
26566	11741656_a_at	SLCO1C1	solute carrier organic anion transporter family, member 1C1	NM_001145944 /// NM_001145945 /// NM_001145946 /// NM_017435	0,0353183	0,221186	0,0353183	1,104	1,104	rapid up vs Slow	0,143	4,641	2,154	4,641	0,304	1,475	0,387
9239	11724329_a_at	DGCR8	DiGeorge syndrome critical region gene 8	NM_001190326 /// NM_022720	0,0353612	6,82E-05	0,0353612	1,057	1,057	rapid up vs Slow	0,080	4,638	2,154	4,638	0,097	7,387	0,618
6367	11721457_a_at	SERPINA1	serpin peptidase inhibitor, clade A (alpha-1 antiproteinase, antitrypsin), member 1	NM_000295 /// NM_001002235 /// NM_001002236 /// NM_001127700 /// NM_001127701 /// NM_00	0,0353763	0,360604	0,0353763	1,130	1,130	rapid up vs Slow	0,177	4,638	2,154	4,638	0,468	1,110	0,448
32209	11747299_a_at	CALCOCO1	calcium binding and coiled-coil domain 1	NM_001143682 /// NM_020898 /// NR_026554	0,0354185	0,0470948	0,0354185	1,089	1,089	rapid up vs Slow	0,123	4,635	2,153	4,635	0,226	2,570	0,501
35105	11750195_a_at	UEVLD	UEV and lactate/malate dehyrogenase domains	NM_001040697 /// NM_018314	0,0354538	0,701839	0,0354538	1,108	1,108	rapid up vs Slow	0,148	4,634	2,153	4,634	0,327	0,547	0,155
39069	11754159_a_at	CD28	CD28 molecule	NM_006139	0,0354846	0,127269	0,0354846	0,904	-1,106	rapid down vs Slow	-0,146	4,632	-2,152	4,632	0,318	1,872	0,514
1867	11716957_x_at	CTDSP2	CTD (carboxy-terminal domain, RNA polymerase II, polypeptide A) small phosphatase 2	NM_005730	0,035543	0,741947	0,035543	0,832	-1,201	rapid down vs Slow	-0,265	4,629	-2,151	4,629	1,050	0,491	0,446
35096	11750186_a_at	ALDH1A3	aldehyde dehydrogenase 1 family,	NM_000693	0,0355625	0,142991	0,0355625	1,095	1,095	rapid up vs Slow	0,131	4,628	2,151	4,628	0,257	1,790	0,398

			member A3														
48845	11763935_at	DISC1 /// TSNAX /// TSNAX-DISC1	disrupted in schizophrenia 1 /// translin-associated factor X /// TSNAX-DISC1 gene	NM_001012957 /// NM_001012958 /// NM_001012959 /// NM_001164537 /// NM_001164538 /// NM	0,0356207	0,0266914	0,0356207	1,106	1,106	rapid up vs Slow	0,145	4,625	2,151	4,625	0,316	2,966	0,809
2166	11717256_at	PIM1	pim-1 oncogene	NM_002648	0,0356291	0,190145	0,0356291	0,800	-1,251	rapid down vs Slow	-0,323	4,624	-2,150	4,624	1,561	1,585	2,141
47057	11762147_at	HMGA2	high mobility group AT-hook 2	NM_003483 /// NM_003484	0,0356611	0,00520927	0,0356611	1,106	1,106	rapid up vs Slow	0,145	4,623	2,150	4,623	0,314	4,118	1,119
28080	11743170_a_at	RCSD1	RCSD domain containing 1	NM_052862	0,0357265	0,0126978	0,0357265	0,799	-1,252	rapid down vs Slow	-0,324	4,619	-2,149	4,619	1,573	3,487	4,748
36147	11751237_a_at	DUSP10	dual specificity phosphatase 10	NM_007207 /// NM_144728 /// NM_144729	0,0357865	0,109312	0,0357865	1,089	1,089	rapid up vs Slow	0,123	4,616	2,149	4,616	0,226	1,980	0,388
40612	11755702_x_at	RRP7A	ribosomal RNA processing 7 homolog A (S. cerevisiae)	NM_015703	0,035821	0,065083	0,035821	1,226	1,226	rapid up vs Slow	0,293	4,614	2,148	4,614	1,291	2,344	2,622
43110	11758200_x_at	CKS1B	CDC28 protein kinase regulatory subunit 1B	NM_001826 /// NR_024163	0,0358303	0,840553	0,0358303	0,900	-1,111	rapid down vs Slow	-0,152	4,614	-2,148	4,614	0,344	0,354	0,105
8027	11723117_a_at	PCNT	pericentrin	NM_006031	0,0359027	0,027694	0,0359027	0,803	-1,246	rapid down vs Slow	-0,317	4,610	-2,147	4,610	1,505	2,940	3,840
13459	11728549_at	ZNF536	zinc finger protein 536	NM_014717	0,0359385	0,347038	0,0359385	1,073	1,073	rapid up vs Slow	0,101	4,608	2,147	4,608	0,154	1,139	0,152
18235	11733325_a_at	FBXW8	F-box and WD repeat domain containing 8	NM_012174 /// NM_153348	0,0359801	0,363756	0,0359801	1,094	1,094	rapid up vs Slow	0,130	4,606	2,146	4,606	0,253	1,103	0,242
23393	11738483_a_at	TNFRSF13B	tumor necrosis factor receptor superfamily, member 13B	NM_012452	0,0359925	0,121448	0,0359925	0,855	-1,170	rapid down vs Slow	-0,226	4,606	-2,146	4,606	0,768	1,906	1,270
15579	11730669_at	OPHN1	oligophrenin 1	NM_002547	0,0360421	0,380397	0,0360421	1,107	1,107	rapid up vs Slow	0,146	4,603	2,145	4,603	0,320	1,068	0,297
41325	11756415_x_at	RBM6	RNA binding motif protein 6	NM_001167582 /// NM_005777	0,0360446	0,0257427	0,0360446	0,840	-1,191	rapid down vs Slow	-0,252	4,603	-2,145	4,603	0,953	2,991	2,476
12217	11727307_x_at	ZNF320	zinc finger protein 320	NM_207333	0,0360702	0,786491	0,0360702	1,094	1,094	rapid up vs Slow	0,130	4,602	2,145	4,602	0,252	0,430	0,094
23177	11738267_at	C7orf33	chromosome 7 open reading frame 33	NM_145304	0,0360848	0,0113209	0,0360848	1,091	1,091	rapid up vs Slow	0,125	4,601	2,145	4,601	0,236	3,567	0,732
15654	11730744_x_at	AKIRIN1	akirin 1	NM_001136275 /// NM_024595	0,0361295	0,0433452	0,0361295	0,795	-1,257	rapid down vs Slow	-0,330	4,599	-2,144	4,599	1,634	2,628	3,734
11757	11726847_a_at	SEMA5B	sema domain, seven thrombospondin repeats (type 1 and type 1-like), transmembrane domai	NM_001031702	0,0361528	0,0357889	0,0361528	1,105	1,105	rapid up vs Slow	0,144	4,597	2,144	4,597	0,313	2,761	0,751
24465	11739555_a_at	FAM171B	family with sequence similarity 171, member B	NM_177454	0,0361553	0,108291	0,0361553	1,140	1,140	rapid up vs Slow	0,190	4,597	2,144	4,597	0,538	1,987	0,930

42579	11757669_s_at	PPIL1	peptidylprolyl isomerase (cyclophilin)-like 1	NM_016059	0,0361659	0,0155083	0,0361659	0,835	-1,198	rapid down vs Slow	-0,261	4,597	-2,144	4,597	1,018	3,346	2,965
42400	11757490_s_at	TSPYL1	TSPY-like 1	NM_003309	0,0361832	0,0229745	0,0361832	0,786	-1,272	rapid down vs Slow	-0,347	4,596	-2,144	4,596	1,802	3,071	4,816
15779	11730869_s_at	PPY	pancreatic polypeptide	NM_002722	0,0362212	0,0899828	0,0362212	1,128	1,128	rapid up vs Slow	0,174	4,594	2,143	4,594	0,453	2,117	0,834
22157	11737247_at	ZNF141	zinc finger protein 141	NM_003441	0,0362641	0,174617	0,0362641	1,082	1,082	rapid up vs Slow	0,114	4,592	2,143	4,592	0,194	1,647	0,279
48443	11763533_a_at	ZDHHC19	zinc finger, DHHC-type containing 19	NM_001039617	0,0362909	0,945084	0,0362909	1,241	1,241	rapid up vs Slow	0,311	4,590	2,142	4,590	1,452	0,185	0,235
39281	11754371_a_at	FAM102A	family with sequence similarity 102, member A	NM_001035254 /// NM_203305	0,0363264	0,158494	0,0363264	0,810	-1,234	rapid down vs Slow	-0,304	4,588	-2,142	4,588	1,382	1,716	2,068
36729	11751819_a_at	PPP2R3A	protein phosphatase 2, regulatory subunit B'', alpha	NM_001190447 /// NM_002718 /// NM_181897	0,0363767	0,241723	0,0363767	1,073	1,073	rapid up vs Slow	0,102	4,586	2,141	4,586	0,155	1,410	0,190
11462	11726552_x_at	ULBP2	UL16 binding protein 2	NM_025217	0,0363812	0,808602	0,0363812	1,077	1,077	rapid up vs Slow	0,107	4,586	2,141	4,586	0,173	0,399	0,060
22806	11737896_s_at	TAF13	TAF13 RNA polymerase II, TATA box binding protein (TBP)-associated factor, 18kDa	NM_005645	0,0364026	0,644298	0,0364026	1,075	1,075	rapid up vs Slow	0,105	4,585	2,141	4,585	0,164	0,628	0,090
6002	11721092_a_at	THBS1	thrombospondin 1	NM_003246	0,0364239	0,385445	0,0364239	1,076	1,076	rapid up vs Slow	0,105	4,583	2,141	4,583	0,166	1,058	0,154
23546	11738636_x_at	PCDHGA3	protocadherin gamma subfamily A, 3	NM_018916 /// NM_032011	0,0364347	0,392724	0,0364347	1,076	1,076	rapid up vs Slow	0,105	4,583	2,141	4,583	0,166	1,043	0,151
25593	11740683_a_at	PRKG1	protein kinase, cGMP-dependent, type I	NM_001098512 /// NM_006258	0,0364412	0,465325	0,0364412	1,082	1,082	rapid up vs Slow	0,113	4,583	2,141	4,583	0,192	0,908	0,152
17230	11732320_a_at	ZNF92	zinc finger protein 92	NM_007139 /// NM_152626	0,0364552	0,100021	0,0364552	0,886	-1,129	rapid down vs Slow	-0,175	4,582	-2,141	4,582	0,457	2,043	0,815
12369	11727459_x_at	KRT14	keratin 14	NM_000526	0,0366205	0,221126	0,0366205	1,075	1,075	rapid up vs Slow	0,105	4,574	2,139	4,574	0,165	1,476	0,213
30010	11745100_a_at	CDH19	cadherin 19, type 2	NM_021153	0,0366206	0,159886	0,0366206	1,068	1,068	rapid up vs Slow	0,095	4,574	2,139	4,574	0,136	1,710	0,204
31513	11746603_a_at	ACAP3	ArfGAP with coiled-coil, ankyrin repeat and PH domains 3	NM_030649	0,0367353	0,122616	0,0367353	1,129	1,129	rapid up vs Slow	0,175	4,568	2,137	4,568	0,459	1,899	0,763
35826	11750916_a_at	FAM73A	family with sequence similarity 73, member A	NM_198549	0,0367575	0,108397	0,0367575	1,091	1,091	rapid up vs Slow	0,126	4,567	2,137	4,567	0,236	1,986	0,411
4115	11719205_at	ANKLE2	ankyrin repeat and LEM domain containing 2	NM_015114	0,0367628	0,157153	0,0367628	0,904	-1,106	rapid down vs Slow	-0,145	4,566	-2,137	4,566	0,316	1,722	0,477
16109	11731199_at	LHFPL3	lipoma HMGIC fusion partner-like 3	NM_199000	0,0367746	0,308022	0,0367746	1,184	1,184	rapid up vs Slow	0,243	4,566	2,137	4,566	0,887	1,230	0,956
31411	11746501_x_at	ANKRD30BP2	ankyrin repeat domain 30B pseudogene 2	NR_026916	0,0368649	0,205191	0,0368649	1,074	1,074	rapid up vs Slow	0,103	4,561	2,136	4,561	0,158	1,530	0,211
16889	11731979_	FNDC7	fibronectin type	NM_0011449	0,036898	0,036132	0,0368983	0,912	-1,096	rapid down	-0,132	4,560	-2,135	4,560	0,263	2,754	0,635

	at		III domain containing 7	37	3	8				vs Slow							
8230	11723320_at	CALY	calcyon neuron-specific vesicular protein	NM_015722	0,0369169	0,0310828	0,0369169	1,085	1,085	rapid up vs Slow	0,118	4,559	2,135	4,559	0,209	2,860	0,524
25548	11740638_at	NOG	noggin	NM_005450	0,0370373	0,376817	0,0370373	0,837	-1,195	rapid down vs Slow	-0,257	4,553	-2,134	4,553	0,988	1,076	0,933
42577	11757667_x_at	APOA2	apolipoprotein A-II	NM_001643	0,0370483	0,0644115	0,0370483	1,093	1,093	rapid up vs Slow	0,128	4,552	2,134	4,552	0,247	2,351	0,511
32154	11747244_a_at	KCTD20	potassium channel tetramerisation domain containing 20	NM_173562	0,0370829	0,00720438	0,0370829	1,092	1,092	rapid up vs Slow	0,128	4,550	2,133	4,550	0,244	3,887	0,833
33800	11748890_a_at	C9orf7	chromosome 9 open reading frame 7	NM_001135775 /// NM_017586	0,0371468	0,0111687	0,0371468	1,120	1,120	rapid up vs Slow	0,163	4,547	2,132	4,547	0,400	3,577	1,260
35178	11750268_a_at	C12orf33	chromosome 12 open reading frame 33	NR_034140	0,0372121	0,0268097	0,0372121	1,071	1,071	rapid up vs Slow	0,098	4,544	2,132	4,544	0,145	2,963	0,378
43610	11758700_s_at	SMC3	structural maintenance of chromosomes 3	NM_005445	0,0372843	0,0312112	0,0372843	0,845	-1,184	rapid down vs Slow	-0,244	4,540	-2,131	4,540	0,889	2,857	2,237
7521	11722611_at	TBC1D25	TBC1 domain family, member 25	NM_002536	0,0372865	0,701716	0,0372865	0,872	-1,147	rapid down vs Slow	-0,197	4,540	-2,131	4,540	0,584	0,547	0,282
47616	11762706_at	LCA5	Leber congenital amaurosis 5	NM_001122769 /// NM_181714	0,0373798	0,036598	0,0373798	1,082	1,082	rapid up vs Slow	0,113	4,536	2,130	4,536	0,193	2,746	0,466
11243	11726333_s_at	LEF1	lymphoid enhancer-binding factor 1	NM_001130713 /// NM_001130714 /// NM_001166119 /// NM_016269	0,0373842	0,0949316	0,0373842	0,749	-1,336	rapid down vs Slow	-0,418	4,535	-2,130	4,535	2,612	2,079	4,790
45466	11760556_at	MGC24103	hypothetical MGC24103	XR_108934 /// XR_113198 /// XR_114250	0,0374298	0,527002	0,0374298	1,148	1,148	rapid up vs Slow	0,199	4,533	2,129	4,533	0,596	0,805	0,423
33057	11748147_a_at	MRPL2	mitochondrial ribosomal protein L2	NM_015950	0,0374481	0,15389	0,0374481	1,091	1,091	rapid up vs Slow	0,126	4,532	2,129	4,532	0,239	1,737	0,366
3311	11718401_a_at	NUDT21	nudix (nucleoside diphosphate linked moiety X)-type motif 21	NM_007006	0,03749	0,00270854	0,03749	0,921	-1,086	rapid down vs Slow	-0,119	4,530	-2,128	4,530	0,211	4,589	0,853
11129	11726219_x_at	EPB49	erythrocyte membrane protein band 4.9 (dematin)	NM_001114135 /// NM_001114136 /// NM_001114137 /// NM_001114138 /// NM_001114139 /// NM	0,0375184	0,022961	0,0375184	0,772	-1,295	rapid down vs Slow	-0,372	4,529	-2,128	4,529	2,079	3,071	5,638
1650	11716740_a_at	SLC6A8	solute carrier family 6 (neurotransmitter transporter, creatine), member 8	NM_001142805 /// NM_001142806 /// NM_005629	0,0375611	0,294469	0,0375611	0,705	-1,419	rapid down vs Slow	-0,505	4,527	-2,128	4,527	3,816	1,264	4,261
25179	11740269_at	TTLL11	tubulin tyrosine ligase-like family, member	NM_001139442 /// NM_194252	0,0375703	0,207129	0,0375703	1,101	1,101	rapid up vs Slow	0,139	4,526	2,127	4,526	0,291	1,523	0,391

			11														
588	11715678_s_at	NAP1L4	nucleosome assembly protein 1-like 4	NM_005969	0,0376625	0,202996	0,0376625	0,887	-1,127	rapid down vs Slow	-0,173	4,522	-2,126	4,522	0,446	1,538	0,607
22212	11737302_at	CHST5	carbohydrate (N-acetylglucosamine 6-O) sulfotransferase 5	NM_024533	0,0376787	0,00537204	0,0376787	1,090	1,090	rapid up vs Slow	0,124	4,521	2,126	4,521	0,229	4,096	0,831
34229	11749319_a_at	PGAP1	post-GPI attachment to proteins 1	NM_024989	0,0377518	0,1915	0,0377518	1,092	1,092	rapid up vs Slow	0,127	4,517	2,125	4,517	0,242	1,580	0,339
17586	11732676_at	PKHD1	polycystic kidney and hepatic disease 1 (autosomal recessive)	NM_138694 /// NM_170724	0,0378003	0,0427106	0,0378003	1,092	1,092	rapid up vs Slow	0,127	4,515	2,125	4,515	0,243	2,638	0,568
30742	11745832_a_at	NFKBIB	nuclear factor of kappa light polypeptide gene enhancer in B-cells inhibitor, beta	NM_001001716 /// NM_002503	0,0378944	0,00690545	0,0378944	1,138	1,138	rapid up vs Slow	0,187	4,510	2,124	4,510	0,524	3,917	1,821
31441	11746531_a_at	C2orf60	chromosome 2 open reading frame 60	NM_001039693 /// NR_004862	0,0379087	0,0717252	0,0379087	1,099	1,099	rapid up vs Slow	0,136	4,510	2,124	4,510	0,276	2,276	0,556
1072	11716162_at	LBH	limb bud and heart development homolog (mouse)	NM_030915	0,0379154	0,0565398	0,0379154	0,795	-1,258	rapid down vs Slow	-0,331	4,509	-2,124	4,509	1,640	2,442	3,553
38221	11753311_a_at	C9orf41	chromosome 9 open reading frame 41	NM_152420	0,0379273	0,660865	0,0379273	1,090	1,090	rapid up vs Slow	0,125	4,509	2,123	4,509	0,234	0,605	0,125
15816	11730906_at	PDS5B	PDS5, regulator of cohesion maintenance, homolog B (S. cerevisiae)	NM_015032	0,0379665	0,309411	0,0379665	0,848	-1,179	rapid down vs Slow	-0,238	4,507	-2,123	4,507	0,846	1,226	0,921
45462	11760552_a_at	HEATR7A	HEAT repeat containing 7A	NM_001099280 /// NM_001099281 /// NM_032450	0,0379712	0,00438475	0,0379712	1,137	1,137	rapid up vs Slow	0,185	4,507	2,123	4,507	0,511	4,242	1,923
2456	11717546_s_at	MPRIP	myosin phosphatase Rho interacting protein	NM_015134 /// NM_201274	0,0379755	0,159374	0,0379755	0,853	-1,172	rapid down vs Slow	-0,229	4,506	-2,123	4,506	0,788	1,712	1,198
31991	11747081_a_at	INPP5A	inositol polyphosphate-5-phosphatase, 40kDa	NM_005539	0,0380713	0,999863	0,0380713	1,076	1,076	rapid up vs Slow	0,106	4,502	2,122	4,502	0,169	0,008	0,001
49029	11764119_at	---	---	---	0,0380847	0,293298	0,0380847	1,101	1,101	rapid up vs Slow	0,138	4,501	2,122	4,501	0,287	1,267	0,323
27396	11742486_s_at	PRAMEF18 /// PRAMEF19 /// PRAMEF22 /// PRAMEF3	PRAME family member 18 /// PRAME family member 19 /// PRAME family member 22 /// PRAME	NM_001013692 /// NM_001099790 /// NM_001099850 /// NM_001100631	0,03816	0,153187	0,03816	1,106	1,106	rapid up vs Slow	0,145	4,497	2,121	4,497	0,316	1,740	0,489
38499	11753589_x_at	---	---	---	0,0381675	0,582937	0,0381675	1,086	1,086	rapid up vs Slow	0,119	4,497	2,121	4,497	0,214	0,718	0,137
14305	11729395_s_at	HECTD2	HECT domain containing 2	NM_173497 ///	0,0381859	0,511538	0,0381859	1,090	1,090	rapid up vs Slow	0,124	4,496	2,120	4,496	0,229	0,830	0,169

				NM_182765												
38147	11753237_x_at	LOC728989	phosphodiesterase 4D interacting protein pseudogene	NR_024442	0,0381873	0,9273	0,0381873	1,090	1,090	rapid up vs Slow	0,124	4,496	2,120	4,496	0,232	0,218	0,045
14380	11729470_at	FAM181B	family with sequence similarity 181, member B	NM_175885	0,0382092	0,00733863	0,0382092	1,083	1,083	rapid up vs Slow	0,115	4,495	2,120	4,495	0,198	3,874	0,684
42084	11757174_x_at	SNORA5B /// TBRG4	small nucleolar RNA, H/ACA box 5B /// transforming growth factor beta regulator 4	NM_004749 /// NM_030900 /// NM_199122 /// NR_002990	0,0382208	0,118935	0,0382208	1,094	1,094	rapid up vs Slow	0,130	4,494	2,120	4,494	0,252	1,920	0,431
14162	11729252_a_at	SLC44A3	solute carrier family 44, member 3	NM_001114106 /// NM_152369	0,038277	0,0479498	0,038277	1,104	1,104	rapid up vs Slow	0,143	4,492	2,119	4,492	0,307	2,557	0,700
22125	11737215_a_at	EFCAB4A	EF-hand calcium binding domain 4A	NM_173584	0,038298	0,1812	0,038298	1,120	1,120	rapid up vs Slow	0,164	4,491	2,119	4,491	0,403	1,620	0,582
6549	11721639_a_at	ABHD8	abhydrolase domain containing 8	NM_024527	0,0383066	0,0158588	0,0383066	1,116	1,116	rapid up vs Slow	0,159	4,490	2,119	4,490	0,378	3,330	1,122
6868	11721958_at	YPEL2	yippee-like 2 (Drosophila)	NM_001005404	0,0383262	0,0156492	0,0383262	1,120	1,120	rapid up vs Slow	0,163	4,489	2,119	4,489	0,400	3,340	1,190
44516	11759606_at	YIPF2	Yip1 domain family, member 2	NM_024029	0,0383577	0,510827	0,0383577	0,830	-1,205	rapid down vs Slow	-0,268	4,488	-2,118	4,488	1,080	0,831	0,800
14208	11729298_a_at	C1orf55	chromosome 1 open reading frame 55	NM_152608	0,0383803	0,0136318	0,0383803	0,830	-1,205	rapid down vs Slow	-0,269	4,487	-2,118	4,487	1,087	3,437	3,330
48882	11763972_at	SSR1	signal sequence receptor, alpha	NM_003144	0,0384117	0,288966	0,0384117	1,150	1,150	rapid up vs Slow	0,201	4,485	2,118	4,485	0,607	1,278	0,691
48586	11763676_at	IGHA1 /// IGHG1 /// IGHG3 /// IGHM /// IGHV4-31 /// LOC100510678	immunoglobulin heavy constant alpha 1 /// immunoglobulin heavy constant gamma 1 (G1m ma	XM_003120441	0,0384478	0,359693	0,0384478	0,946	-1,058	rapid down vs Slow	-0,081	4,484	-2,117	4,484	0,098	1,112	0,097
13231	11728321_a_at	NSL1	NSL1, MIND kinetochore complex component, homolog (S. cerevisiae)	NM_001042549 /// NM_015471	0,0384757	0,930092	0,0384757	0,900	-1,111	rapid down vs Slow	-0,152	4,482	-2,117	4,482	0,346	0,213	0,066
43499	11758589_s_at	ERCC4	excision repair cross-complementing rodent repair deficiency, complementation group 4	NM_005236	0,0384839	0,152453	0,0384839	0,905	-1,105	rapid down vs Slow	-0,144	4,482	-2,117	4,482	0,311	1,744	0,484
12284	11727374_s_at	HMGCR	3-hydroxy-3-methylglutaryl-CoA reductase	NM_000859 /// NM_001130996	0,0385036	0,164245	0,0385036	0,843	-1,186	rapid down vs Slow	-0,246	4,481	-2,117	4,481	0,909	1,691	1,372
31403	11746493_a_at	PIP4K2A	phosphatidylinositol-5-phosphate 4-kinase, type II, alpha	NM_005028	0,0385156	0,257249	0,0385156	0,800	-1,250	rapid down vs Slow	-0,322	4,480	-2,117	4,480	1,550	1,364	1,888
21902	11736992_a_at	C3orf55	chromosome 3 open reading frame 55	NM_001099777 /// NM_0011300	0,0385525	0,144175	0,0385525	1,096	1,096	rapid up vs Slow	0,132	4,479	2,116	4,479	0,263	1,784	0,419

				01 /// NM_0011300 02 /// NR_024016													
37556	11752646_a_at	KCNJ5	potassium inwardly-rectifying channel, subfamily J, member 5	NM_000890	0,038581	0,001824 47	0,038581	1,101	1,101	rapid up vs Slow	0,139	4,477	2,116	4,477	0,290	4,876	1,266
26686	11741776_at	TMEM179	transmembrane protein 179	NM_207379	0,038600 5	0,141462	0,0386005	1,093	1,093	rapid up vs Slow	0,128	4,476	2,116	4,476	0,244	1,797	0,393
9433	11724523_a_at	CDK5RAP2	CDK5 regulatory subunit associated protein 2	NM_0010116 49 /// NM_018249	0,038643 6	0,059850 9	0,0386436	1,161	1,161	rapid up vs Slow	0,216	4,474	2,115	4,474	0,699	2,402	1,501
44462	11759552_at	MPI	mannose phosphate isomerase	NM_002435	0,038650 6	0,168591	0,0386506	0,899	-1,112	rapid down vs Slow	-0,154	4,474	-2,115	4,474	0,354	1,672	0,529
28831	11743921_at	TMEM125	transmembrane protein 125	NM_144626	0,038685 7	0,014762 9	0,0386857	1,097	1,097	rapid up vs Slow	0,133	4,472	2,115	4,472	0,267	3,381	0,806
17570	11732660_s_at	HSFY1 /// HSFY2	heat shock transcription factor, Y-linked 1 /// heat shock transcription factor, Y link	NM_0010018 77 /// NM_033108 /// NM_152584 /// NM_153716 /// NR_003509 /// NR_003510	0,038686 9	0,024164 1	0,0386869	1,081	1,081	rapid up vs Slow	0,112	4,472	2,115	4,472	0,188	3,035	0,509
13631	11728721_x_at	PDE1B	phosphodiesterase 1B, calmodulin-dependent	NM_000924 /// NM_0011659 75	0,038784 3	0,141852	0,0387843	1,099	1,099	rapid up vs Slow	0,136	4,467	2,114	4,467	0,279	1,795	0,449
2246	11717336_a_at	RBM6	RNA binding motif protein 6	NM_0011675 82 /// NM_005777	0,038808 6	0,077748 4	0,0388086	0,847	-1,181	rapid down vs Slow	-0,240	4,466	-2,113	4,466	0,863	2,219	1,715
8335	11723425_at	IFNAR1	interferon (alpha, beta and omega) receptor 1	NM_000629	0,038819 7	0,499889	0,0388197	0,843	-1,187	rapid down vs Slow	-0,247	4,466	-2,113	4,466	0,913	0,849	0,694
11141	11726231_at	NR0B2	nuclear receptor subfamily 0, group B, member 2	NM_021969	0,038883 3	0,001011 73	0,0388833	1,116	1,116	rapid up vs Slow	0,158	4,463	2,113	4,463	0,375	5,311	1,785
14302	11729392_a_at	TRIM3	tripartite motif-containing 3	NM_006458 /// NM_033278	0,038903 9	0,232839	0,0389039	1,065	1,065	rapid up vs Slow	0,090	4,462	2,112	4,462	0,122	1,438	0,158
20666	11735756_a_at	DNAH14	dynein, axonemal, heavy chain 14	NM_0011451 54 /// NM_001373 /// NM_144989	0,038923 1	0,093035 8	0,0389231	1,126	1,126	rapid up vs Slow	0,171	4,461	2,112	4,461	0,437	2,093	0,821
24586	11739676_a_at	C4orf40	chromosome 4 open reading frame 40	NM_214711	0,038966 1	0,037529 9	0,0389661	1,064	1,064	rapid up vs Slow	0,089	4,459	2,112	4,459	0,119	2,728	0,290
44127	11759217_at	WNT3	wingless-type MMTV integration site family, member 3	NM_030753	0,038966 3	0,040227 4	0,0389663	1,128	1,128	rapid up vs Slow	0,173	4,459	2,112	4,459	0,451	2,680	1,083
20194	11735284_x_at	MLL5	myeloid/lymphoid or mixed-lineage leukemia 5 (trithorax homolog, Drosophila)	NM_018682 /// NM_182931	0,039019 2	0,010304 2	0,0390192	0,812	-1,231	rapid down vs Slow	-0,300	4,456	-2,111	4,456	1,346	3,634	4,392

504	11715594_a_at	RBM38	RNA binding motif protein 38	NM_017495 /// NM_183425	0,0390716	0,070273	0,0390716	0,750	-1,333	rapid down vs Slow	-0,414	4,454	-2,110	4,454	2,570	2,290	5,287
37341	11752431_a_at	ANAPC2 /// LOC650621	anaphase promoting complex subunit 2 /// anaphase-promoting complex subunit 2-like	NM_013366 /// XM_939710	0,0390944	0,0960754	0,0390944	1,114	1,114	rapid up vs Slow	0,156	4,453	2,110	4,453	0,363	2,071	0,675
16197	11731287_at	GRXCR1	glutaredoxin, cysteine rich 1	NM_001080476	0,0391033	0,138801	0,0391033	1,084	1,084	rapid up vs Slow	0,117	4,452	2,110	4,452	0,204	1,811	0,332
9808	11724898_a_at	SNPH	syntaphilin	NM_014723	0,0391314	0,0869023	0,0391314	0,904	-1,106	rapid down vs Slow	-0,146	4,451	-2,110	4,451	0,319	2,141	0,613
6146	11721236_a_at	LHFPL2	lipoma HMGIC fusion partner-like 2	NM_005779	0,0391819	0,0580599	0,0391819	1,107	1,107	rapid up vs Slow	0,147	4,449	2,109	4,449	0,322	2,424	0,701
32217	11747307_a_at	GDI1	GDP dissociation inhibitor 1	NM_001493	0,0391901	0,104416	0,0391901	0,925	-1,081	rapid down vs Slow	-0,112	4,448	-2,109	4,448	0,187	2,012	0,339
5276	11720366_at	FDX1L	ferredoxin 1-like	NM_001031734	0,0391949	0,39943	0,0391949	0,903	-1,107	rapid down vs Slow	-0,147	4,448	-2,109	4,448	0,324	1,030	0,300
22682	11737772_x_at	ZNF93	zinc finger protein 93	NM_031218	0,039242	0,27016	0,039242	1,110	1,110	rapid up vs Slow	0,151	4,446	2,109	4,446	0,340	1,328	0,406
25508	11740598_x_at	WDR31	WD repeat domain 31	NM_001012361 /// NM_145241	0,039247	0,15458	0,039247	1,116	1,116	rapid up vs Slow	0,158	4,446	2,108	4,446	0,375	1,734	0,585
20427	11735517_a_at	TRIM60	tripartite motif-containing 60	NM_152620	0,0392806	0,046311	0,0392806	1,111	1,111	rapid up vs Slow	0,152	4,444	2,108	4,444	0,347	2,581	0,806
47069	11762159_at	EHBP1	EH domain binding protein 1	NM_001142614 /// NM_001142615 /// NM_001142616 /// NM_015252	0,0392876	0,037617	0,0392876	1,095	1,095	rapid up vs Slow	0,131	4,444	2,108	4,444	0,257	2,726	0,630
5652	11720742_a_at	ZNF605	zinc finger protein 605	NM_001164715 /// NM_183238	0,0393105	0,04808	0,0393105	0,880	-1,137	rapid down vs Slow	-0,185	4,443	-2,108	4,443	0,514	2,555	1,183
16315	11731405_s_at	ATP6V1G1	ATPase, H+ transporting, lysosomal 13kDa, V1 subunit G1	NM_004888	0,0393911	0,798078	0,0393911	0,913	-1,096	rapid down vs Slow	-0,132	4,439	-2,107	4,439	0,260	0,414	0,097
15545	11730635_a_at	GRHL3	grainyhead-like 3 (Drosophila)	NM_001195010 /// NM_021180 /// NM_198173 /// NM_198174	0,0393949	0,538224	0,0393949	1,112	1,112	rapid up vs Slow	0,153	4,439	2,107	4,439	0,349	0,787	0,248
4142	11719232_x_at	PTPRS	protein tyrosine phosphatase, receptor type, S	NM_002850 /// NM_130853 /// NM_130854 /// NM_130855	0,039402	0,0837186	0,039402	1,101	1,101	rapid up vs Slow	0,138	4,438	2,107	4,438	0,287	2,168	0,560
649	11715739_s_at	PPAP2B	phosphatidic acid phosphatase type 2B	NM_003713	0,0394314	0,180093	0,0394314	1,076	1,076	rapid up vs Slow	0,105	4,437	2,106	4,437	0,166	1,624	0,243
6668	11721758_at	TMEM47	transmembrane protein 47	NM_031442	0,0394742	0,244772	0,0394742	1,096	1,096	rapid up vs Slow	0,133	4,435	2,106	4,435	0,264	1,401	0,333
48750	11763840_a_at	MEGF10	multiple EGF-like-domains 10	NM_032446	0,0395649	0,302117	0,0395649	1,095	1,095	rapid up vs Slow	0,130	4,431	2,105	4,431	0,255	1,244	0,287

34620	11749710_s_at	ABLIM1	actin binding LIM protein 1	NM_001003407 /// NM_001003408 /// NM_002313 /// NM_006720	0,0396148	0,488038	0,0396148	0,824	-1,213	rapid down vs Slow	-0,279	4,428	-2,104	4,428	1,163	0,869	0,913
31723	11746813_a_at	PRKG2	protein kinase, cGMP-dependent, type II	NM_006259	0,0396149	0,4437	0,0396149	1,089	1,089	rapid up vs Slow	0,123	4,428	2,104	4,428	0,228	0,946	0,195
15485	11730575_at	ZNF470	zinc finger protein 470	NM_001001668	0,0396334	0,128563	0,0396334	1,100	1,100	rapid up vs Slow	0,137	4,428	2,104	4,428	0,283	1,865	0,477
11443	11726533_a_at	CCDC148	coiled-coil domain containing 148	NM_001171637 /// NM_138803	0,039668	0,0138931	0,039668	1,070	1,070	rapid up vs Slow	0,097	4,426	2,104	4,426	0,142	3,423	0,438
36458	11751548_a_at	C10orf10	chromosome 10 open reading frame 10	NM_007021	0,0396727	0,179574	0,0396727	1,123	1,123	rapid up vs Slow	0,167	4,426	2,104	4,426	0,417	1,626	0,612
28559	11743649_a_at	DCAF6	DDB1 and CUL4 associated factor 6	NM_001017977 /// NM_018442	0,0397065	0,155566	0,0397065	0,777	-1,287	rapid down vs Slow	-0,364	4,424	-2,103	4,424	1,989	1,729	3,110
16067	11731157_at	ERCC4	excision repair cross-complementing rodent repair deficiency, complementation group 4	NM_005236	0,039727	0,0538342	0,039727	1,069	1,069	rapid up vs Slow	0,097	4,423	2,103	4,423	0,141	2,476	0,315
2573	11717663_a_at	KLHL12	kelch-like 12 (Drosophila)	NM_021633	0,0397387	0,038445	0,0397387	0,907	-1,102	rapid down vs Slow	-0,140	4,423	-2,103	4,423	0,295	2,711	0,722
1945	11717035_s_at	KIAA0141	KIAA0141	NM_001142603 /// NM_014773	0,039763	0,87643	0,039763	1,085	1,085	rapid up vs Slow	0,118	4,422	2,103	4,422	0,208	0,301	0,057
39812	11754902_a_at	POLH	polymerase (DNA directed), eta	NM_006502	0,0397634	0,52047	0,0397634	1,101	1,101	rapid up vs Slow	0,139	4,422	2,103	4,422	0,289	0,815	0,213
22176	11737266_x_at	RHD	Rh blood group, D antigen	NM_001127691 /// NM_016124	0,0397656	0,117761	0,0397656	0,873	-1,146	rapid down vs Slow	-0,196	4,421	-2,103	4,421	0,577	1,927	1,006
35677	11750767_x_at	SLC17A7	solute carrier family 17 (sodium-dependent inorganic phosphate cotransporter), member 7	NM_020309	0,0397681	0,263193	0,0397681	1,079	1,079	rapid up vs Slow	0,109	4,421	2,103	4,421	0,180	1,347	0,219
43115	11758205_x_at	CAPNS1	calpain, small subunit 1	NM_001003962 /// NM_001749	0,0397715	0,110827	0,0397715	1,114	1,114	rapid up vs Slow	0,156	4,421	2,103	4,421	0,365	1,970	0,651
3936	11719026_a_at	PSD3	pleckstrin and Sec7 domain containing 3	NM_015310 /// NM_206909	0,0398564	0,10407	0,0398564	1,068	1,068	rapid up vs Slow	0,095	4,417	2,102	4,417	0,134	2,015	0,245
28686	11743776_a_at	GLT8D2	glycosyltransferase 8 domain containing 2	NM_031302	0,0399166	0,052778	0,0399166	1,113	1,113	rapid up vs Slow	0,154	4,414	2,101	4,414	0,356	2,490	0,803
5601	11720691_at	DIRAS1	DIRAS family, GTP-binding RAS-like 1	NM_145173	0,0399222	0,120233	0,0399222	1,112	1,112	rapid up vs Slow	0,153	4,414	2,101	4,414	0,350	1,913	0,606
16223	11731313_a_at	GCFC1	GC-rich sequence DNA-binding factor 1	NM_013329 /// NM_016631 /// NR_027873	0,039958	0,841638	0,039958	0,908	-1,101	rapid down vs Slow	-0,139	4,413	-2,101	4,413	0,288	0,352	0,092
10521	11725611_a_at	TMEM200B	transmembrane protein 200B	NM_001003682 ///	0,0399644	0,362174	0,0399644	0,830	-1,205	rapid down vs Slow	-0,269	4,412	-2,101	4,412	1,082	1,106	1,086

				NM_001171868													
42213	11757303_s_at	BNIP3L	BCL2/adenovirus E1B 19kDa interacting protein 3-like	NM_004331	0,0399723	0,951354	0,0399723	0,636	-1,573	rapid down vs Slow	-0,653	4,412	-2,100	4,412	6,394	0,173	1,003
21717	11736807_at	GABRA4	gamma-aminobutyric acid (GABA) A receptor, alpha 4	NM_000809	0,0400025	0,0927533	0,0400025	1,100	1,100	rapid up vs Slow	0,138	4,411	2,100	4,411	0,284	2,096	0,540
47819	11762909_at	LOC100127951	ISPF6484	XM_001724438 /// XM_001724497 /// XM_001726747	0,0400619	0,412016	0,0400619	1,112	1,112	rapid up vs Slow	0,153	4,408	2,099	4,408	0,351	1,005	0,320
21017	11736107_at	ENTPD4	ectonucleoside triphosphate diphosphohydrolase 4	NM_001128930 /// NM_004901	0,0400843	0,133342	0,0400843	0,902	-1,109	rapid down vs Slow	-0,149	4,407	-2,099	4,407	0,332	1,839	0,554
12355	11727445_a_at	FAM70A	family with sequence similarity 70, member A	NM_001104544 /// NM_001104545 /// NM_017938	0,040121	0,539787	0,040121	1,080	1,080	rapid up vs Slow	0,111	4,405	2,099	4,405	0,183	0,785	0,130
44290	11759380_at	KRTAP6-2	keratin associated protein 6-2	NM_181604	0,0401248	0,00301389	0,0401248	1,090	1,090	rapid up vs Slow	0,124	4,405	2,099	4,405	0,231	4,512	0,946
38467	11753557_a_at	CCDC7	coiled-coil domain containing 7	NM_001026383 /// NM_145023	0,0401387	0,904591	0,0401387	1,092	1,092	rapid up vs Slow	0,126	4,404	2,099	4,404	0,239	0,256	0,056
41687	11756777_a_at	HCRTR2	hypocretin (orexin) receptor 2	NM_001526	0,0401682	0,567754	0,0401682	1,082	1,082	rapid up vs Slow	0,114	4,403	2,098	4,403	0,195	0,741	0,132
38666	11753756_a_at	ZFYVE21	zinc finger, FYVE domain containing 21	NM_024071	0,0401718	0,615478	0,0401718	1,097	1,097	rapid up vs Slow	0,134	4,403	2,098	4,403	0,269	0,670	0,163
13014	11728104_at	HTR2B	5-hydroxytryptamine (serotonin) receptor 2B	NM_000867	0,0402339	0,0974488	0,0402339	1,201	1,201	rapid up vs Slow	0,264	4,400	2,098	4,400	1,045	2,061	1,958
7914	11723004_x_at	F2	coagulation factor II (thrombin)	NM_000506	0,0402386	0,306884	0,0402386	1,094	1,094	rapid up vs Slow	0,130	4,400	2,098	4,400	0,252	1,233	0,283
44077	11759167_at	HIST1H2BE	histone cluster 1, H2be	NM_003523	0,0403548	0,133188	0,0403548	1,093	1,093	rapid up vs Slow	0,129	4,394	2,096	4,394	0,249	1,840	0,417
21292	11736382_at	ZNF697	zinc finger protein 697	NM_001080470	0,04039	0,721621	0,04039	1,112	1,112	rapid up vs Slow	0,154	4,393	2,096	4,393	0,353	0,520	0,167
40717	11755807_a_at	DNA2	DNA replication helicase 2 homolog (yeast)	NM_001080449	0,0404094	0,0221613	0,0404094	1,106	1,106	rapid up vs Slow	0,146	4,392	2,096	4,392	0,319	3,096	0,900
20505	11735595_a_at	SH2D4B	SH2 domain containing 4B	NM_001145719 /// NM_207372	0,040424	0,246191	0,040424	1,128	1,128	rapid up vs Slow	0,174	4,391	2,096	4,391	0,452	1,397	0,575
29958	11745048_a_at	ACSBG1	acyl-CoA synthetase bubblegum family member 1	NM_015162	0,0404366	0,186765	0,0404366	1,104	1,104	rapid up vs Slow	0,143	4,391	2,095	4,391	0,308	1,598	0,448
26961	11742051_at	TCHHL1	trichohyalin-like 1	NM_001008536	0,0404902	0,0862213	0,0404902	1,150	1,150	rapid up vs Slow	0,201	4,388	2,095	4,388	0,608	2,147	1,190
16584	11731674_a_at	AGMAT	agmatine ureohydrolase (agmatinase)	NM_024758	0,0405101	0,0500372	0,0405101	0,899	-1,112	rapid down vs Slow	-0,153	4,387	-2,095	4,387	0,353	2,527	0,813
22905	11737995_at	LRRC3	leucine rich repeat containing 3	NM_030891	0,0405108	0,00941437	0,0405108	1,095	1,095	rapid up vs Slow	0,131	4,387	2,095	4,387	0,255	3,698	0,861

16993	11732083_a_at	STOML3	stomatin (EPB72)-like 3	NM_001144033 /// NM_145286	0,0405153	0,0111756	0,0405153	1,118	1,118	rapid up vs Slow	0,161	4,387	2,095	4,387	0,387	3,577	1,262
11621	11726711_x_at	PFAS	phosphoribosylformylglycinamidine synthase	NM_012393	0,0405515	0,093817	0,0405515	0,823	-1,216	rapid down vs Slow	-0,282	4,385	-2,094	4,385	1,188	2,088	2,262
24073	11739163_a_at	ALDOB	aldolase B, fructose-bisphosphate	NM_000035	0,040552	0,186413	0,040552	1,068	1,068	rapid up vs Slow	0,095	4,385	2,094	4,385	0,136	1,600	0,199
40509	11755599_x_at	POU5F1	POU class 5 homeobox 1	NM_001173531 /// NM_002701 /// NM_203289	0,040568	0,233585	0,040568	1,116	1,116	rapid up vs Slow	0,158	4,385	2,094	4,385	0,375	1,435	0,492
47288	11762378_a_at	VPRBP	Vpr (HIV-1) binding protein	NM_001171904 /// NM_014703	0,040588	0,0556196	0,040588	0,878	-1,139	rapid down vs Slow	-0,188	4,384	-2,094	4,384	0,530	2,454	1,186
12386	11727476_s_at	SAP30	Sin3A-associated protein, 30kDa	NM_003864	0,0405927	0,0152056	0,0405927	1,123	1,123	rapid up vs Slow	0,168	4,384	2,094	4,384	0,421	3,360	1,292
7628	11722718_a_at	BAIAP2	BAI1-associated protein 2	NM_001144888 /// NM_006340 /// NM_017450 /// NM_017451	0,0406277	0,00231745	0,0406277	1,087	1,087	rapid up vs Slow	0,121	4,382	2,093	4,382	0,219	4,702	0,941
4670	11719760_at	ARRDC4	arrestin domain containing 4	NM_183376	0,0406534	0,494075	0,0406534	1,085	1,085	rapid up vs Slow	0,117	4,381	2,093	4,381	0,206	0,859	0,161
5880	11720970_at	TOP2A	topoisomerase (DNA) II alpha 170kDa	NM_001067	0,0406808	0,00191347	0,0406808	0,841	-1,188	rapid down vs Slow	-0,249	4,380	-2,093	4,380	0,929	4,842	4,108
30300	11745390_a_at	PC	pyruvate carboxylase	NM_000920 /// NM_001040716 /// NM_022172	0,0406939	0,500247	0,0406939	1,097	1,097	rapid up vs Slow	0,134	4,379	2,093	4,379	0,268	0,848	0,208
17380	11732470_a_at	C12orf50	chromosome 12 open reading frame 50	NM_152589	0,0407003	0,41676	0,0407003	1,080	1,080	rapid up vs Slow	0,111	4,379	2,093	4,379	0,186	0,996	0,169
45615	11760705_a_at	NDUFS2	NADH dehydrogenase (ubiquinone) Fe-S protein 2, 49kDa (NADH-coenzyme Q reductase)	NM_001166159 /// NM_004550	0,0407146	0,114832	0,0407146	0,854	-1,171	rapid down vs Slow	-0,228	4,378	-2,092	4,378	0,779	1,945	1,385
35187	11750277_a_at	POLR3D	polymerase (RNA) III (DNA directed) polypeptide D, 44kDa	NM_001722	0,040717	0,2874	0,040717	0,899	-1,112	rapid down vs Slow	-0,154	4,378	-2,092	4,378	0,354	1,282	0,415
11076	11726166_at	ZNF518B	zinc finger protein 518B	NM_053042	0,0408295	0,09089	0,0408295	0,896	-1,116	rapid down vs Slow	-0,159	4,373	-2,091	4,373	0,378	2,110	0,730
32575	11747665_a_at	LYSMD4	LysM, putative peptidoglycan-binding, domain containing 4	NM_152449	0,040842	0,052375	0,040842	1,084	1,084	rapid up vs Slow	0,116	4,372	2,091	4,372	0,201	2,496	0,459
42803	11757893_s_at	TNKS2	tankyrase, TRF1-interacting ankyrin-related ADP-ribose polymerase 2	NM_025235	0,0409253	0,802425	0,0409253	0,903	-1,107	rapid down vs Slow	-0,147	4,369	-2,090	4,369	0,324	0,408	0,121
30761	11745851_x_at	HSPD1	heat shock 60kDa protein 1 (chaperonin)	NM_002156 /// NM_199440	0,0409466	0,0030257	0,0409466	0,897	-1,115	rapid down vs Slow	-0,157	4,368	-2,090	4,368	0,369	4,509	1,524

20819	11735909_at	OR10H2	olfactory receptor, family 10, subfamily H, member 2	NM_013939	0,0410452	0,704809	0,0410452	1,106	1,106	rapid up vs Slow	0,145	4,363	2,089	4,363	0,316	0,543	0,157
33068	11748158_a_at	PRR4	proline rich 4 (lacrimal)	NM_001098538 /// NM_007244	0,0410503	0,200682	0,0410503	1,073	1,073	rapid up vs Slow	0,101	4,363	2,089	4,363	0,154	1,546	0,218
5298	11720388_s_at	C1QC	complement component 1, q subcomponent, C chain	NM_001114101 /// NM_172369	0,0410535	0,188014	0,0410535	1,124	1,124	rapid up vs Slow	0,168	4,363	2,089	4,363	0,423	1,593	0,618
18309	11733399_a_at	XG	Xg blood group	NM_001141919 /// NM_001141920 /// NM_175569	0,0410948	0,0311863	0,0410948	1,096	1,096	rapid up vs Slow	0,132	4,361	2,088	4,361	0,260	2,857	0,680
19122	11734212_at	CT45A5 /// LOC100133581	cancer/testis antigen family 45, member A5 /// cancer/testis antigen family 45 member A	NM_001007551 /// NM_001172288 /// XM_001716002	0,0410978	0,085793	0,0410978	1,100	1,100	rapid up vs Slow	0,138	4,361	2,088	4,361	0,286	2,150	0,563
16673	11731763_s_at	RPS6KA2	ribosomal protein S6 kinase, 90kDa, polypeptide 2	NM_001006932 /// NM_021135	0,0411318	0,0182106	0,0411318	1,114	1,114	rapid up vs Slow	0,156	4,359	2,088	4,359	0,363	3,233	1,077
16907	11731997_at	TRIM58	tripartite motif-containing 58	NM_015431	0,0411664	0,0444587	0,0411664	0,655	-1,527	rapid down vs Slow	-0,611	4,358	-2,088	4,358	5,591	2,610	13,394
31554	11746644_a_at	C1orf93	chromosome 1 open reading frame 93	NM_001195736 /// NM_001195737 /// NM_001195738 /// NM_001195740 /// NM_001195741 /// NM	0,0411712	0,00726466	0,0411712	1,100	1,100	rapid up vs Slow	0,138	4,358	2,087	4,358	0,284	3,881	1,011
44137	11759227_at	CLEC4C	C-type lectin domain family 4, member C	NM_130441 /// NM_203503	0,0411777	0,0616337	0,0411777	1,123	1,123	rapid up vs Slow	0,168	4,357	2,087	4,357	0,422	2,382	0,922
26368	11741458_a_at	NR4A3	nuclear receptor subfamily 4, group A, member 3	NM_006981 /// NM_173199 /// NM_173200	0,0411835	0,0191454	0,0411835	1,083	1,083	rapid up vs Slow	0,115	4,357	2,087	4,357	0,197	3,198	0,579
21175	11736265_x_at	FBXO44	F-box protein 44	NM_001014765 /// NM_033182 /// NM_183412 /// NM_183413	0,0412134	0,00440939	0,0412134	1,116	1,116	rapid up vs Slow	0,158	4,356	2,087	4,356	0,374	4,238	1,455
15829	11730919_a_at	SLC10A7	solute carrier family 10 (sodium/bile acid cotransporter family), member 7	NM_001029998 /// NM_032128	0,0412375	0,0859959	0,0412375	1,067	1,067	rapid up vs Slow	0,093	4,355	2,087	4,355	0,130	2,149	0,257
9325	11724415_at	DISP1	dispatched homolog 1 (Drosophila)	NM_032890	0,0413566	0,330876	0,0413566	1,076	1,076	rapid up vs Slow	0,106	4,349	2,086	4,349	0,167	1,176	0,180
42335	11757425_s_at	VCAM1	vascular cell adhesion molecule 1	NM_001078 /// NM_080682	0,0413732	0,159936	0,0413732	1,094	1,094	rapid up vs Slow	0,130	4,349	2,085	4,349	0,251	1,710	0,395
20193	11735283_	MLL5	myeloid/lymphoi	NM_018682	0,041417	0,007870	0,0414179	0,816	-1,225	rapid down	-0,293	4,347	-2,085	4,347	1,286	3,824	4,527

	a_at		d or mixed-lineage leukemia 5 (trithorax homolog, Drosophila)	/// NM_182931	9	51				vs Slow							
47032	11762122_a_at	GLS2	glutaminase 2 (liver, mitochondrial)	NM_013267	0,0414228	0,00551078	0,0414228	1,096	1,096	rapid up vs Slow	0,133	4,346	2,085	4,346	0,263	4,078	0,987
264	11715354_a_at	COL1A2	collagen, type I, alpha 2	NM_000089	0,041434	0,372132	0,041434	1,095	1,095	rapid up vs Slow	0,131	4,346	2,085	4,346	0,258	1,085	0,257
12452	11727542_a_at	MPDZ	multiple PDZ domain protein	NM_003829	0,0414344	0,0121466	0,0414344	1,108	1,108	rapid up vs Slow	0,149	4,346	2,085	4,346	0,331	3,518	1,071
38409	11753499_a_at	KRTAP1-1	keratin associated protein 1-1	NM_030967	0,0415221	0,115923	0,0415221	1,094	1,094	rapid up vs Slow	0,129	4,342	2,084	4,342	0,251	1,938	0,448
41495	11756585_a_at	AQP3	aquaporin 3 (Gill blood group)	NM_004925	0,0415553	0,0928815	0,0415553	0,792	-1,262	rapid down vs Slow	-0,336	4,341	-2,083	4,341	1,692	2,095	3,267
20502	11735592_a_at	MYO3B	myosin IIIB	NM_001083615 /// NM_001171642 /// NM_138995	0,041621	0,0375813	0,041621	1,110	1,110	rapid up vs Slow	0,151	4,338	2,083	4,338	0,340	2,727	0,855
10296	11725386_a_at	HOMER1	homer homolog 1 (Drosophila)	NM_004272	0,0416217	0,0136575	0,0416217	1,097	1,097	rapid up vs Slow	0,134	4,338	2,083	4,338	0,268	3,435	0,848
38326	11753416_a_at	ABCA2	ATP-binding cassette, sub-family A (ABC1), member 2	NM_001606 /// NM_212533	0,0416556	0,127213	0,0416556	0,915	-1,093	rapid down vs Slow	-0,128	4,336	-2,082	4,336	0,245	1,873	0,423
27253	11742343_at	OR4K5	olfactory receptor, family 4, subfamily K, member 5	NM_001005483	0,0416649	0,219487	0,0416649	1,079	1,079	rapid up vs Slow	0,110	4,336	2,082	4,336	0,182	1,481	0,249
35842	11750932_a_at	EPHX2	epoxide hydrolase 2, cytoplasmic	NM_001979	0,0416787	0,144556	0,0416787	0,834	-1,199	rapid down vs Slow	-0,262	4,335	-2,082	4,335	1,025	1,782	1,686
31634	11746724_a_at	BTN2A3	butyrophilin, subfamily 2, member A3	NR_027795	0,0416934	0,0687222	0,0416934	1,092	1,092	rapid up vs Slow	0,127	4,335	2,082	4,335	0,242	2,306	0,515
29422	11744512_a_at	C2orf89	chromosome 2 open reading frame 89	NM_001080824	0,0417679	0,227512	0,0417679	0,722	-1,384	rapid down vs Slow	-0,469	4,331	-2,081	4,331	3,296	1,455	4,428
8250	11723340_at	TTC28	tetratricopeptide repeat domain 28	NM_001145418	0,0418207	0,0601357	0,0418207	1,091	1,091	rapid up vs Slow	0,125	4,329	2,081	4,329	0,235	2,399	0,521
29230	11744320_at	PIGZ	phosphatidylinositol glycan anchor biosynthesis, class Z	NM_025163	0,0418396	0,0146388	0,0418396	0,912	-1,096	rapid down vs Slow	-0,133	4,328	-2,080	4,328	0,265	3,387	0,828
7262	11722352_s_at	ACTA2	actin, alpha 2, smooth muscle, aorta	NM_001141945 /// NM_001613	0,0418748	0,589259	0,0418748	1,280	1,280	rapid up vs Slow	0,356	4,327	2,080	4,327	1,900	0,709	1,245
12457	11727547_s_at	RBBP8	retinoblastoma binding protein 8	NM_002894 /// NM_203291 /// NM_203292	0,0419016	0,322227	0,0419016	0,852	-1,173	rapid down vs Slow	-0,230	4,325	-2,080	4,325	0,795	1,196	0,879
5820	11720910_a_at	NELL2	NEL-like 2 (chicken)	NM_001145107 /// NM_001145108 /// NM_001145109 /// NM_001145110 /// NM_006159	0,0419218	0,231005	0,0419218	0,786	-1,273	rapid down vs Slow	-0,348	4,325	-2,080	4,325	1,813	1,444	2,421

29862	11744952_at	MGC4294	hypothetical MGC4294	XR_109628 /// XR_112170 /// XR_115388	0,0419277	0,0159436	0,0419277	1,089	1,089	rapid up vs Slow	0,123	4,324	2,079	4,324	0,226	3,327	0,696
6310	11721400_a_at	ELOF1	elongation factor 1 homolog (S. cerevisiae)	NM_032377	0,0419944	0,101501	0,0419944	0,837	-1,194	rapid down vs Slow	-0,256	4,321	-2,079	4,321	0,982	2,032	1,847
48838	11763928_at	FAM24B	family with sequence similarity 24, member B	NM_152644	0,0420623	0,327049	0,0420623	1,118	1,118	rapid up vs Slow	0,161	4,318	2,078	4,318	0,389	1,184	0,427
10409	11725499_at	GOLGA7B	golgin A7 family, member B	NM_001010917	0,0420784	0,201337	0,0420784	0,871	-1,148	rapid down vs Slow	-0,199	4,318	-2,078	4,318	0,592	1,544	0,846
34502	11749592_x_at	GFAP	glial fibrillary acidic protein	NM_001131019 /// NM_002055	0,0421202	0,535324	0,0421202	0,913	-1,095	rapid down vs Slow	-0,132	4,316	-2,077	4,316	0,259	0,792	0,190
48045	11763135_at	---	---	---	0,0421469	0,496991	0,0421469	1,088	1,088	rapid up vs Slow	0,122	4,315	2,077	4,315	0,224	0,854	0,177
36707	11751797_a_at	IL11	interleukin 11	NM_000641	0,0421756	0,424872	0,0421756	1,123	1,123	rapid up vs Slow	0,167	4,313	2,077	4,313	0,418	0,981	0,380
28696	11743786_a_at	THAP9	THAP domain containing 9	NM_024672	0,0422094	0,925819	0,0422094	0,909	-1,100	rapid down vs Slow	-0,138	4,312	-2,077	4,312	0,284	0,221	0,058
36210	11751300_a_at	CASQ1	calsequestrin 1 (fast-twitch, skeletal muscle)	NM_001231	0,0422267	0,285999	0,0422267	1,083	1,083	rapid up vs Slow	0,115	4,311	2,076	4,311	0,199	1,286	0,237
33679	11748769_a_at	POLL	polymerase (DNA directed), lambda	NM_001174084 /// NM_001174085 /// NM_013274 /// NR_033406	0,0423113	0,0348369	0,0423113	0,777	-1,288	rapid down vs Slow	-0,365	4,308	-2,075	4,308	1,994	2,780	5,148
41248	11756338_x_at	DHRS11	dehydrogenase/reductase (SDR family) member 11	NM_024308	0,042317	0,00693502	0,042317	1,117	1,117	rapid up vs Slow	0,159	4,307	2,075	4,307	0,380	3,914	1,380
32055	11747145_x_at	BRF1	BRF1 homolog, subunit of RNA polymerase III transcription initiation factor IIIB (S. ce	NM_001519 /// NM_145685	0,0423241	0,834689	0,0423241	1,089	1,089	rapid up vs Slow	0,123	4,307	2,075	4,307	0,226	0,362	0,076
27101	11742191_a_at	RUNX1	runt-related transcription factor 1	NM_001001890 /// NM_001122607 /// NM_001754	0,0423344	0,195052	0,0423344	0,823	-1,215	rapid down vs Slow	-0,281	4,307	-2,075	4,307	1,185	1,567	1,725
47371	11762461_x_at	GP1BB	glycoprotein Ib (platelet), beta polypeptide	NM_000407	0,0423481	0,348968	0,0423481	1,118	1,118	rapid up vs Slow	0,160	4,306	2,075	4,306	0,386	1,135	0,407
33109	11748199_a_at	TMEM189	transmembrane protein 189	NM_001162505 /// NM_199129 /// NR_027889	0,0423785	0,152333	0,0423785	1,104	1,104	rapid up vs Slow	0,143	4,305	2,075	4,305	0,306	1,744	0,496
23313	11738403_at	KRT40	keratin 40	NM_182497	0,0423822	0,509662	0,0423822	1,074	1,074	rapid up vs Slow	0,103	4,305	2,075	4,305	0,159	0,833	0,123
20417	11735507_at	C14orf39	chromosome 14 open reading frame 39	NM_174978	0,0423947	0,0377187	0,0423947	1,080	1,080	rapid up vs Slow	0,111	4,304	2,075	4,304	0,184	2,725	0,465
32405	11747495_a_at	USP19	ubiquitin specific peptidase 19	NM_006677	0,0424616	0,0233376	0,0424616	1,127	1,127	rapid up vs Slow	0,172	4,301	2,074	4,301	0,443	3,060	1,262
44111	11759201_at	TMEM38A	transmembrane protein 38A	NM_024074	0,0424875	0,120755	0,0424875	1,150	1,150	rapid up vs Slow	0,201	4,300	2,074	4,300	0,608	1,910	1,079
21687	11736777_at	CBLN1	cerebellin 1 precursor	NM_004352	0,0425154	0,250113	0,0425154	1,085	1,085	rapid up vs Slow	0,118	4,299	2,073	4,299	0,209	1,385	0,270
133	11715223_s_at	RNASE8	ribonuclease, RNase A family, 8	NM_138331	0,0425312	0,00673474	0,0425312	1,110	1,110	rapid up vs Slow	0,151	4,298	2,073	4,298	0,340	3,935	1,246

43417	11758507_s_at	SHC4	SHC (Src homology 2 domain containing) family, member 4	NM_203349	0,0425433	0,994539	0,0425433	1,066	1,066	rapid up vs Slow	0,092	4,298	2,073	4,298	0,128	0,053	0,006
40909	11755999_a_at	DEPDC4	DEP domain containing 4	NM_152317	0,042555	0,219776	0,042555	1,094	1,094	rapid up vs Slow	0,129	4,297	2,073	4,297	0,250	1,480	0,345
4400	11719490_a_at	TMEM189	transmembrane protein 189	NM_001162505 /// NM_199129 /// NR_027889	0,0425618	0,837144	0,0425618	0,871	-1,148	rapid down vs Slow	-0,199	4,297	-2,073	4,297	0,590	0,358	0,197
38080	11753170_a_at	CD300C	CD300c molecule	NM_006678	0,0425857	0,310644	0,0425857	1,163	1,163	rapid up vs Slow	0,218	4,296	2,073	4,296	0,715	1,223	0,815
47624	11762714_at	FILIP1L	filamin A interacting protein 1-like	NM_001042459 /// NM_014890 /// NM_182909	0,0426001	0,321749	0,0426001	1,105	1,105	rapid up vs Slow	0,144	4,295	2,072	4,295	0,312	1,197	0,348
23402	11738492_x_at	KRTAP5-10	keratin associated protein 5-10	NM_001012710	0,0426006	0,0111728	0,0426006	1,137	1,137	rapid up vs Slow	0,186	4,295	2,072	4,295	0,517	3,577	1,720
21679	11736769_a_at	USP45	ubiquitin specific peptidase 45	NM_001080481	0,0426611	0,324803	0,0426611	1,099	1,099	rapid up vs Slow	0,137	4,293	2,072	4,293	0,280	1,190	0,311
16287	11731377_s_at	RABL2A /// RABL2B	RAB, member of RAS oncogene family-like 2A /// RAB, member of RAS oncogene family-like	NM_001003789 /// NM_001130919 /// NM_001130920 /// NM_001130921 /// NM_001130922 /// NM	0,0426691	0,472057	0,0426691	1,086	1,086	rapid up vs Slow	0,119	4,292	2,072	4,292	0,213	0,896	0,178
38030	11753120_x_at	MADCAM1	mucosal vascular addressin cell adhesion molecule 1	NM_130760 /// NM_130762	0,0427132	0,221139	0,0427132	1,091	1,091	rapid up vs Slow	0,126	4,290	2,071	4,290	0,237	1,476	0,326
29602	11744692_s_at	HP1BP3	heterochromatin protein 1, binding protein 3	NM_016287	0,04278	0,00764507	0,04278	0,859	-1,164	rapid down vs Slow	-0,219	4,287	-2,071	4,287	0,721	3,845	2,585
13270	11728360_at	BCDIN3D	BCDIN3 domain containing	NM_181708	0,0427849	0,101531	0,0427849	0,891	-1,123	rapid down vs Slow	-0,167	4,287	-2,071	4,287	0,417	2,032	0,790
27702	11742792_at	ARL9	ADP-ribosylation factor-like 9	NM_206919	0,0427943	0,353605	0,0427943	1,104	1,104	rapid up vs Slow	0,143	4,287	2,070	4,287	0,308	1,125	0,323
44002	11759092_at	USP12	ubiquitin specific peptidase 12	NM_182488	0,0428073	0,457799	0,0428073	0,763	-1,310	rapid down vs Slow	-0,390	4,286	-2,070	4,286	2,273	0,921	1,954
42810	11757900_a_at	VTN	vitronectin	NM_000638	0,0428758	0,857077	0,0428758	1,077	1,077	rapid up vs Slow	0,107	4,283	2,070	4,283	0,173	0,329	0,053
11237	11726327_at	DCUN1D1	DCN1, defective in cullin neddylation 1, domain containing 1 (S. cerevisiae)	NM_020640	0,042923	0,729594	0,042923	0,818	-1,222	rapid down vs Slow	-0,290	4,281	-2,069	4,281	1,257	0,509	0,597
7438	11722528_a_at	PTPN21	protein tyrosine phosphatase, non-receptor type 21	NM_007039	0,0429545	0,0449063	0,0429545	1,093	1,093	rapid up vs Slow	0,129	4,280	2,069	4,280	0,249	2,603	0,606
22302	11737392_at	PROKR2	prokineticin receptor 2	NM_144773	0,0431189	0,00245307	0,0431189	1,114	1,114	rapid up vs Slow	0,156	4,273	2,067	4,273	0,366	4,661	1,597
23086	11738176_at	NKX3-2	NK3 homeobox 2	NM_001189	0,0431249	0,702455	0,0431249	1,094	1,094	rapid up vs Slow	0,130	4,273	2,067	4,273	0,254	0,546	0,130
198	11715288_s_at	CASP14	caspase 14, apoptosis-	NM_012114	0,0431399	0,0768135	0,0431399	1,076	1,076	rapid up vs Slow	0,105	4,272	2,067	4,272	0,166	2,228	0,346

			related cysteine peptidase														
36947	11752037_a_at	MAVS	mitochondrial antiviral signaling protein	NM_020746	0,043179	0,41622	0,043179	1,142	1,142	rapid up vs Slow	0,191	4,270	2,067	4,270	0,547	0,997	0,511
28661	11743751_a_at	NUDT13	nudix (nucleoside diphosphate linked moiety X)-type motif 13	NM_015901	0,0431897	0,0845643	0,0431897	1,079	1,079	rapid up vs Slow	0,110	4,270	2,066	4,270	0,181	2,160	0,366
32225	11747315_a_at	ENTPD6	ectonucleoside triphosphate diphosphohydrolase 6 (putative)	NM_001114089 /// NM_001247	0,0432559	0,772208	0,0432559	1,085	1,085	rapid up vs Slow	0,118	4,267	2,066	4,267	0,208	0,450	0,088
47580	11762670_at	ELP1P	endozepine-like peptide 1 pseudogene	NR_036635	0,0433025	0,15726	0,0433025	1,100	1,100	rapid up vs Slow	0,138	4,265	2,065	4,265	0,285	1,722	0,460
15875	11730965_a_at	MAPK11	mitogen-activated protein kinase 11	NM_002751	0,0433419	0,343046	0,0433419	1,084	1,084	rapid up vs Slow	0,116	4,264	2,065	4,264	0,201	1,148	0,216
14320	11729410_a_at	ZNF415	zinc finger protein 415	NM_001136038 /// NM_001164309 /// NM_018355 /// NR_028343	0,0434361	0,0291953	0,0434361	1,087	1,087	rapid up vs Slow	0,121	4,260	2,064	4,260	0,218	2,903	0,594
33389	11748479_a_at	RAPGEF1	Rap guanine nucleotide exchange factor (GEF) 1	NM_005312 /// NM_198679	0,0434631	0,120493	0,0434631	1,071	1,071	rapid up vs Slow	0,099	4,258	2,064	4,258	0,147	1,911	0,264
16052	11731142_at	NDST3	N-deacetylase/N-sulfotransferase (heparan glucosaminyl) 3	NM_004784	0,0434887	0,12172	0,0434887	1,088	1,088	rapid up vs Slow	0,122	4,257	2,063	4,257	0,224	1,904	0,401
32573	11747663_a_at	C14orf149	chromosome 14 open reading frame 149	NM_144581	0,0435083	0,287397	0,0435083	1,085	1,085	rapid up vs Slow	0,117	4,257	2,063	4,257	0,207	1,282	0,249
21774	11736864_a_at	KIAA1958	KIAA1958	NM_133465	0,0435303	0,13513	0,0435303	1,116	1,116	rapid up vs Slow	0,158	4,256	2,063	4,256	0,375	1,830	0,645
5066	11720156_a_at	ACAD10	acyl-CoA dehydrogenase family, member 10	NM_001136538 /// NM_025247	0,0435639	0,114575	0,0435639	0,883	-1,133	rapid down vs Slow	-0,180	4,254	-2,063	4,254	0,485	1,947	0,887
43687	11758777_x_at	RBM8A	RNA binding motif protein 8A	NM_005105	0,0435957	0,470236	0,0435957	0,817	-1,224	rapid down vs Slow	-0,291	4,253	-2,062	4,253	1,271	0,899	1,075
34286	11749376_a_at	ABHD4	abhydrolase domain containing 4	NM_022060	0,0436117	0,128986	0,0436117	1,085	1,085	rapid up vs Slow	0,118	4,252	2,062	4,252	0,208	1,863	0,365
48841	11763931_x_at	---	---	---	0,0436352	0,120011	0,0436352	1,069	1,069	rapid up vs Slow	0,096	4,251	2,062	4,251	0,137	1,914	0,247
43756	11758846_x_at	NUS1	nuclear undecaprenyl pyrophosphate synthase 1 homolog (S. cerevisiae)	NM_138459	0,0436484	0,248961	0,0436484	0,901	-1,110	rapid down vs Slow	-0,150	4,251	-2,062	4,251	0,339	1,389	0,443
22681	11737771_a_at	PKIG	protein kinase (cAMP-dependent, catalytic) inhibitor gamma	NM_007066 /// NM_181804 /// NM_181805	0,0436563	0,89537	0,0436563	1,108	1,108	rapid up vs Slow	0,147	4,250	2,062	4,250	0,326	0,271	0,083
7800	11722890_a_at	SLC17A7	solute carrier family 17 (sodium-dependent	NM_020309	0,0436583	0,237173	0,0436583	1,113	1,113	rapid up vs Slow	0,154	4,250	2,062	4,250	0,356	1,424	0,477

			inorganic phosphate cotransporter), member 7														
12760	11727850_x_at	NIP7	nuclear import 7 homolog (S. cerevisiae)	NM_016101	0,0436889	0,0657508	0,0436889	0,846	-1,182	rapid down vs Slow	-0,241	4,249	-2,061	4,249	0,872	2,337	1,917
44018	11759108_at	KRIT1	KRIT1, ankyrin repeat containing	NM_001013406 /// NM_004912 /// NM_194454 /// NM_194455 /// NM_194456	0,0437027	0,127818	0,0437027	1,106	1,106	rapid up vs Slow	0,146	4,248	2,061	4,248	0,318	1,869	0,559
26571	11741661_a_at	ARMC8	armadillo repeat containing 8	NM_014154 /// NM_015396 /// NM_213654	0,0437677	0,00239817	0,0437677	1,084	1,084	rapid up vs Slow	0,116	4,246	2,061	4,246	0,203	4,677	0,895
46618	11761708_a_at	CCDC150	coiled-coil domain containing 150	NM_001080539	0,0437824	0,00180062	0,0437824	1,068	1,068	rapid up vs Slow	0,095	4,245	2,060	4,245	0,136	4,886	0,625
18979	11734069_s_at	SSR3	signal sequence receptor, gamma (translocon-associated protein gamma)	NM_007107	0,0438108	0,0012417	0,0438108	0,884	-1,131	rapid down vs Slow	-0,178	4,244	-2,060	4,244	0,476	5,159	2,314
40014	11755104_a_at	KIAA1671	KIAA1671	NM_001145206	0,0438203	0,0365284	0,0438203	0,868	-1,152	rapid down vs Slow	-0,205	4,244	-2,060	4,244	0,628	2,747	1,626
6052	11721142_a_at	MKI67	antigen identified by monoclonal antibody Ki-67	NM_001145966 /// NM_002417	0,0439277	0,287549	0,0439277	1,087	1,087	rapid up vs Slow	0,120	4,239	2,059	4,239	0,216	1,282	0,262
47435	11762525_s_at	---	---	---	0,0439383	0,126313	0,0439383	1,162	1,162	rapid up vs Slow	0,217	4,239	2,059	4,239	0,705	1,878	1,249
41544	11756634_a_at	GSR	glutathione reductase	NM_000637 /// NM_001195102 /// NM_001195103 /// NM_001195104	0,0439433	0,163736	0,0439433	1,171	1,171	rapid up vs Slow	0,228	4,238	2,059	4,238	0,780	1,693	1,247
8064	11723154_s_at	CAMTA1	calmodulin binding transcription activator 1	NM_001195563 /// NM_015215	0,0439493	0,0710584	0,0439493	1,123	1,123	rapid up vs Slow	0,167	4,238	2,059	4,238	0,419	2,282	0,902
18283	11733373_at	JAK2	Janus kinase 2	NM_004972	0,0439519	0,217147	0,0439519	0,826	-1,210	rapid down vs Slow	-0,275	4,238	-2,059	4,238	1,135	1,489	1,595
19711	11734801_a_at	WIPI2	WD repeat domain, phosphoinositide interacting 2	NM_001033518 /// NM_001033519 /// NM_001033520 /// NM_015610 /// NM_016003	0,0439571	0,421018	0,0439571	0,862	-1,160	rapid down vs Slow	-0,214	4,238	-2,059	4,238	0,683	0,988	0,637
35567	11750657_a_at	NRP1	neuropilin 1	NM_001024628 /// NM_001024629 /// NM_003873	0,0439943	0,109879	0,0439943	1,080	1,080	rapid up vs Slow	0,111	4,236	2,058	4,236	0,185	1,976	0,346
46366	11761456_at	AKAP13	A kinase (PRKA) anchor protein	NM_006738 ///	0,044025	0,0241839	0,044025	1,115	1,115	rapid up vs Slow	0,158	4,235	2,058	4,235	0,372	3,035	1,067

			13	NM_007200 /// NM_144767													
36276	11751366_a_at	GEMIN4	gem (nuclear organelle) associated protein 4	NM_015721	0,0440299	0,150094	0,0440299	1,095	1,095	rapid up vs Slow	0,131	4,235	2,058	4,235	0,256	1,755	0,424
1874	11716964_a_at	PPM1F	protein phosphatase, Mg2+/Mn2+ dependent, 1F	NM_014634	0,044051	0,0063924	0,044051	1,075	1,075	rapid up vs Slow	0,104	4,234	2,058	4,234	0,163	3,972	0,611
1695	11716785_at	TRAK2	trafficking protein, kinesin binding 2	NM_015049	0,0440682	0,090667	0,0440682	0,834	-1,199	rapid down vs Slow	-0,262	4,233	-2,057	4,233	1,027	2,112	2,050
15414	11730504_at	ADAMTS2	ADAM metallopeptidase with thrombospondin type 1 motif, 2	NM_014244 /// NM_021599	0,0440774	0,0199935	0,0440774	1,096	1,096	rapid up vs Slow	0,132	4,233	2,057	4,233	0,260	3,168	0,778
44896	11759986_x_at	TMEM161B	transmembrane protein 161B	NM_153354	0,0440779	0,51909	0,0440779	1,101	1,101	rapid up vs Slow	0,139	4,233	2,057	4,233	0,288	0,818	0,223
47185	11762275_x_at	UQCRB	ubiquinol-cytochrome c reductase binding protein	NM_006294	0,044146	0,447286	0,044146	1,101	1,101	rapid up vs Slow	0,139	4,230	2,057	4,230	0,290	0,940	0,258
41173	11756263_s_at	UBE3B	ubiquitin protein ligase E3B	NM_130466 /// NM_183415	0,0442536	0,0386736	0,0442536	0,850	-1,176	rapid down vs Slow	-0,234	4,226	-2,056	4,226	0,824	2,707	2,111
21759	11736849_a_at	FSD1L	fibronectin type III and SPRY domain containing 1-like	NM_001145313 /// NM_031919 /// NM_207647	0,0443934	0,22251	0,0443934	1,089	1,089	rapid up vs Slow	0,124	4,220	2,054	4,220	0,229	1,471	0,319
32658	11747748_a_at	ETV1	ets variant 1	NM_001163147 /// NM_001163148 /// NM_001163149 /// NM_001163150 /// NM_001163151 /// NM	0,0444318	0,20029	0,0444318	1,078	1,078	rapid up vs Slow	0,109	4,218	2,054	4,218	0,177	1,548	0,260
37717	11752807_a_at	EYA2	eyes absent homolog 2 (Drosophila)	NM_005244 /// NM_172110	0,044495	0,0484432	0,044495	1,095	1,095	rapid up vs Slow	0,130	4,216	2,053	4,216	0,255	2,550	0,617
43948	11759038_at	IL6ST	interleukin 6 signal transducer (gp130, oncostatin M receptor)	NM_001190981 /// NM_002184 /// NM_175767	0,044523	0,920742	0,044523	0,891	-1,123	rapid down vs Slow	-0,167	4,214	-2,053	4,214	0,417	0,230	0,091
4149	11719239_at	PANX1	pannexin 1	NM_015368	0,0445468	0,11605	0,0445468	1,092	1,092	rapid up vs Slow	0,127	4,214	2,053	4,214	0,243	1,938	0,446
24873	11739963_x_at	FDXACB1	ferredoxin-fold anticodon binding domain containing 1	NM_138378	0,0445484	0,62053	0,0445484	0,903	-1,107	rapid down vs Slow	-0,147	4,213	-2,053	4,213	0,322	0,662	0,202
3614	11718704_at	IGF1R	insulin-like growth factor 1 receptor	NM_000875	0,0445868	0,736044	0,0445868	0,821	-1,219	rapid down vs Slow	-0,285	4,212	-2,052	4,212	1,220	0,500	0,579
33834	11748924_a_at	MLL2	myeloid/lymphoid or mixed-lineage leukemia 2	NM_003482	0,0446226	0,448681	0,0446226	1,075	1,075	rapid up vs Slow	0,104	4,210	2,052	4,210	0,162	0,937	0,145
35732	11750822_	SHQ1	SHQ1 homolog	NM_018130	0,044703	0,378827	0,0447036	1,107	1,107	rapid up vs	0,146	4,207	2,051	4,207	0,321	1,071	0,327

	a_at		(S. cerevisiae)		6					Slow							
22369	11737459_a_at	ADARB2	adenosine deaminase, RNA-specific, B2	NM_018702	0,0447194	0,221714	0,0447194	1,104	1,104	rapid up vs Slow	0,143	4,206	2,051	4,206	0,306	1,474	0,429
40508	11755598_a_at	PPP2R3C	protein phosphatase 2, regulatory subunit B'', gamma	NM_017917	0,0447999	0,267305	0,0447999	0,861	-1,161	rapid down vs Slow	-0,215	4,203	-2,050	4,203	0,694	1,336	0,882
1880	11716970_a_at	NRP2	neuropilin 2	NM_003872 /// NM_018534 /// NM_201264 /// NM_201266 /// NM_201267 /// NM_201279	0,0448062	0,651274	0,0448062	1,122	1,122	rapid up vs Slow	0,166	4,203	2,050	4,203	0,412	0,618	0,243
2047	11717137_s_at	CDC42EP4	CDC42 effector protein (Rho GTPase binding) 4	NM_012121	0,0448472	0,234635	0,0448472	1,152	1,152	rapid up vs Slow	0,204	4,201	2,050	4,201	0,626	1,432	0,853
35477	11750567_a_at	MTOR	mechanistic target of rapamycin (serine/threonine kinase)	NM_004958	0,0449054	0,573592	0,0449054	1,079	1,079	rapid up vs Slow	0,110	4,199	2,049	4,199	0,181	0,732	0,126
19819	11734909_s_at	WDR26	WD repeat domain 26	NM_001115113 /// NM_025160	0,0449117	0,063253	0,0449117	0,843	-1,186	rapid down vs Slow	-0,247	4,199	-2,049	4,199	0,911	2,364	2,052
43659	11758749_at	KRTCAP2	keratinocyte associated protein 2	NM_173852	0,0449231	0,693388	0,0449231	1,084	1,084	rapid up vs Slow	0,116	4,198	2,049	4,198	0,202	0,559	0,107
29215	11744305_a_at	LRRK1	leucine-rich repeat kinase 1	NM_024652	0,0449466	0,295032	0,0449466	1,116	1,116	rapid up vs Slow	0,159	4,197	2,049	4,197	0,377	1,262	0,453
32634	11747724_a_at	NUDT12	nudix (nucleoside diphosphate linked moiety X)-type motif 12	NM_031438	0,044992	0,385761	0,044992	1,062	1,062	rapid up vs Slow	0,087	4,195	2,048	4,195	0,113	1,057	0,114
47617	11762707_a_at	C14orf182	chromosome 14 open reading frame 182	NM_001012706	0,0449966	0,307603	0,0449966	0,923	-1,083	rapid down vs Slow	-0,115	4,195	-2,048	4,195	0,199	1,231	0,233
31704	11746794_a_at	C5orf33	chromosome 5 open reading frame 33	NM_001085411 /// NM_153013	0,0450266	0,0311549	0,0450266	1,093	1,093	rapid up vs Slow	0,128	4,194	2,048	4,194	0,246	2,858	0,671
8691	11723781_at	AGPS	alkylglycerone phosphate synthase	NM_003659	0,0450392	0,0216822	0,0450392	0,916	-1,092	rapid down vs Slow	-0,127	4,193	-2,048	4,193	0,243	3,111	0,720
40379	11755469_x_at	MCM7	minichromosome maintenance complex component 7	NM_005916 /// NM_182776	0,0450478	0,0712798	0,0450478	0,873	-1,145	rapid down vs Slow	-0,195	4,193	-2,048	4,193	0,572	2,280	1,243
30728	11745818_x_at	H1F0	H1 histone family, member 0	NM_005318	0,0450776	0,303346	0,0450776	1,118	1,118	rapid up vs Slow	0,160	4,192	2,047	4,192	0,386	1,241	0,457
9368	11724458_at	HCN4	hyperpolarization activated cyclic nucleotide-gated potassium channel 4	NM_005477	0,0451464	0,241203	0,0451464	1,105	1,105	rapid up vs Slow	0,144	4,189	2,047	4,189	0,312	1,412	0,421
33047	11748137_a_at	SEC31B	SEC31 homolog B (S. cerevisiae)	NM_015490	0,0452101	0,0426678	0,0452101	1,090	1,090	rapid up vs Slow	0,124	4,187	2,046	4,187	0,229	2,639	0,578
25923	11741013_	LRRN3	leucine rich	NM_0010996	0,045217	0,120493	0,0452179	0,825	-1,212	rapid down	-0,278	4,186	-2,046	4,186	1,157	1,911	2,113

	a_at		repeat neuronal 3	58 /// NM_0010996 60 /// NM_018334	9					vs Slow								
24814	11739904_ a_at	ABCA6	ATP-binding cassette, sub-family A (ABC1), member 6	NM_080284	0,045227 1	0,140623	0,0452271	1,087	1,087	rapid up vs Slow	0,120	4,186	2,046	4,186	0,217	1,801	0,373
43562	11758652_s _at	KANK1	KN motif and ankyrin repeat domains 1	NM_015158 /// NM_153186	0,045254 7	0,036645 2	0,0452547	0,855	-1,170	rapid down vs Slow	-0,226	4,185	-2,046	4,185	0,767	2,745	2,012
2077	11717167_ a_at	KDM2A	lysine (K)-specific demethylase 2A	NM_012308 /// NR_027473	0,045265 3	0,043368 6	0,0452653	0,860	-1,163	rapid down vs Slow	-0,218	4,184	-2,046	4,184	0,710	2,627	1,783
40138	11755228_ a_at	PTK6	PTK6 protein tyrosine kinase 6	NM_005975	0,045282 8	0,009195 35	0,0452828	1,137	1,137	rapid up vs Slow	0,186	4,184	2,045	4,184	0,516	3,714	1,833
7638	11722728_ a_at	EGR2	early growth response 2	NM_000399 /// NM_0011361 77 /// NM_0011361 78 /// NM_0011361 79	0,045287 8	0,152985	0,0452878	1,088	1,088	rapid up vs Slow	0,121	4,183	2,045	4,183	0,221	1,741	0,368
26026	11741116_ at	FLG	filaggrin	NM_002016	0,045316 8	0,248102	0,0453168	1,091	1,091	rapid up vs Slow	0,125	4,182	2,045	4,182	0,235	1,391	0,312
39174	11754264_ a_at	KHSRP	KH-type splicing regulatory protein	NM_003685	0,045336 1	0,630857	0,0453361	1,135	1,135	rapid up vs Slow	0,183	4,182	2,045	4,182	0,503	0,648	0,312
8457	11723547_ a_at	PEX1	peroxisomal biogenesis factor 1	NM_000466	0,045336 8	0,002771 45	0,0453368	0,881	-1,135	rapid down vs Slow	-0,182	4,181	-2,045	4,181	0,497	4,572	2,173
18305	11733395_ a_at	PPFIA1	protein tyrosine phosphatase, receptor type, f polypeptide (PTPRF), interacting protein	NM_003626 /// NM_177423	0,045349 3	0,060876 1	0,0453493	1,106	1,106	rapid up vs Slow	0,145	4,181	2,045	4,181	0,317	2,391	0,724
13940	11729030_ a_at	SPATS1 /// TMEM15 1B	spermatogenesis associated, serine-rich 1 /// transmembrane protein 151B	NM_0011375 60 /// NM_145026	0,045397 1	0,022011 9	0,0453971	1,086	1,086	rapid up vs Slow	0,119	4,179	2,044	4,179	0,212	3,101	0,628
13952	11729042_ a_at	DLEC1	deleted in lung and esophageal cancer 1	NM_007335 /// NM_007337	0,045522 5	0,911634	0,0455225	1,133	1,133	rapid up vs Slow	0,180	4,174	2,043	4,174	0,486	0,245	0,114
20165	11735255_ at	RNF122	ring finger protein 122	NM_024787	0,045568 7	0,034881 1	0,0455687	1,120	1,120	rapid up vs Slow	0,163	4,172	2,043	4,172	0,399	2,779	1,062
45847	11760937_x _at	CYP19A1	cytochrome P450, family 19, subfamily A, polypeptide 1	NM_000103 /// NM_031226	0,045605 6	0,296336	0,0456056	1,098	1,098	rapid up vs Slow	0,135	4,171	2,042	4,171	0,273	1,259	0,329
37182	11752272_ a_at	MAN1B1	mannosidase, alpha, class 1B, member 1	NM_016219	0,045660 6	0,347573	0,0456606	0,881	-1,135	rapid down vs Slow	-0,182	4,169	-2,042	4,169	0,497	1,138	0,542
6463	11721553_ a_at	GABBR1	gamma-aminobutyric acid (GABA) B receptor, 1	NM_001470 /// NM_021903 /// NM_021904	0,045664 3	0,533523	0,0456643	0,852	-1,174	rapid down vs Slow	-0,232	4,168	-2,042	4,168	0,804	0,794	0,613
46262	11761352_ a_at	CCM2	cerebral cavernous malformation 2	NM_0010298 35 /// NM_0011679 34 /// NM_0011679	0,045676 9	0,184135	0,0456769	1,149	1,149	rapid up vs Slow	0,200	4,168	2,042	4,168	0,600	1,608	0,927

				35 /// NM_031443 /// NR_030770												
9201	11724291_a_at	TRIT1	tRNA isopentenyltransferase 1	NM_017646	0,0456813	0,177001	0,0456813	0,929	-1,076	rapid down vs Slow	-0,106	4,168	-2,042	4,168	0,168	1,637	0,264
25069	11740159_x_at	FGFR2	fibroblast growth factor receptor 2	NM_000141 /// NM_001144913 /// NM_001144914 /// NM_001144915 /// NM_001144916 /// NM_00	0,045697	0,367283	0,045697	1,087	1,087	rapid up vs Slow	0,120	4,167	2,041	4,167	0,217	1,095	0,228
10801	11725891_a_at	ABHD14A /// ACY1	abhydrolase domain containing 14A /// aminoacylase 1	NM_000666 /// NM_015407	0,0458303	0,898407	0,0458303	1,132	1,132	rapid up vs Slow	0,179	4,162	2,040	4,162	0,479	0,266	0,123
40557	11755647_a_at	DHX37	DEAH (Asp-Glu-Ala-His) box polypeptide 37	NM_032656	0,0458304	0,0542761	0,0458304	1,098	1,098	rapid up vs Slow	0,135	4,162	2,040	4,162	0,271	2,471	0,644
5287	11720377_s_at	SAP30L	SAP30-like	NM_001131062 /// NM_001131063 /// NM_024632 /// NR_024084	0,0458627	0,0162197	0,0458627	0,871	-1,148	rapid down vs Slow	-0,200	4,161	-2,040	4,161	0,597	3,314	1,901
41876	11756966_a_at	FAM194A	family with sequence similarity 194, member A	NM_152394	0,0458786	0,0347288	0,0458786	1,085	1,085	rapid up vs Slow	0,117	4,160	2,040	4,160	0,205	2,782	0,550
25753	11740843_a_at	VWDE	von Willebrand factor D and EGF domains	NM_001135924	0,0459023	0,0931417	0,0459023	1,095	1,095	rapid up vs Slow	0,131	4,159	2,039	4,159	0,256	2,093	0,515
16908	11731998_s_at	CCDC88C	coiled-coil domain containing 88C	NM_001080414	0,0459085	0,0258247	0,0459085	0,802	-1,247	rapid down vs Slow	-0,318	4,159	-2,039	4,159	1,519	2,989	4,367
16496	11731586_at	DOK3	docking protein 3	NM_001144875 /// NM_001144876 /// NM_024872	0,0459612	0,537215	0,0459612	1,195	1,195	rapid up vs Slow	0,257	4,157	2,039	4,157	0,992	0,789	0,753
30082	11745172_a_at	SYNPR	synaptoporin	NM_001130003 /// NM_144642	0,0460337	0,281873	0,0460337	1,075	1,075	rapid up vs Slow	0,105	4,154	2,038	4,154	0,164	1,296	0,205
23733	11738823_a_at	SLC26A5	solute carrier family 26, member 5 (prestin)	NM_001167962 /// NM_198999 /// NM_206883 /// NM_206884 /// NM_206885	0,0460566	0,0736319	0,0460566	1,065	1,065	rapid up vs Slow	0,090	4,153	2,038	4,153	0,123	2,257	0,267
39900	11754990_a_at	EPB41	erythrocyte membrane protein band 4.1 (elliptocytosis 1, RH-linked)	NM_001166005 /// NM_001166006 /// NM_001166007 /// NM_004437 /// NM_203342	0,0461059	0,0189357	0,0461059	0,822	-1,216	rapid down vs Slow	-0,282	4,151	-2,037	4,151	1,194	3,206	3,689

				/// NM_20334													
23446	11738536_at	WNT1	wingless-type MMTV integration site family, member 1	NM_005430	0,0461173	0,0174775	0,0461173	1,075	1,075	rapid up vs Slow	0,104	4,150	2,037	4,150	0,161	3,262	0,507
11379	11726469_s_at	C1orf106	chromosome 1 open reading frame 106	NM_001142569 /// NM_018265	0,0461233	0,480644	0,0461233	1,113	1,113	rapid up vs Slow	0,154	4,150	2,037	4,150	0,356	0,881	0,303
49070	11764160_at	---	---	---	0,0461271	0,00226326	0,0461271	1,112	1,112	rapid up vs Slow	0,153	4,150	2,037	4,150	0,351	4,719	1,595
5073	11720163_at	VEGFC	vascular endothelial growth factor C	NM_005429	0,046151	0,276672	0,046151	1,080	1,080	rapid up vs Slow	0,111	4,149	2,037	4,149	0,183	1,310	0,231
14846	11729936_a_at	CLCNKA	chloride channel Ka	NM_001042704 /// NM_004070	0,0461815	0,000983418	0,0461815	1,100	1,100	rapid up vs Slow	0,138	4,148	2,037	4,148	0,284	5,332	1,458
15444	11730534_x_at	COL13A1	collagen, type XIII, alpha 1	NM_001130103 /// NM_080798 /// NM_080800 /// NM_080801 /// NM_080802 /// NM_080805	0,0462339	0,136899	0,0462339	1,113	1,113	rapid up vs Slow	0,154	4,146	2,036	4,146	0,355	1,821	0,623
25793	11740883_a_at	RWDD3	RWD domain containing 3	NM_001128142 /// NM_015485	0,0462341	0,233231	0,0462341	0,939	-1,065	rapid down vs Slow	-0,091	4,146	-2,036	4,146	0,125	1,437	0,173
27904	11742994_x_at	CSH1	chorionic somatomammotropin hormone 1 (placental lactogen)	NM_001317 /// NM_022640 /// NM_022641	0,0463099	0,548706	0,0463099	1,076	1,076	rapid up vs Slow	0,106	4,143	2,035	4,143	0,169	0,771	0,126
29641	11744731_at	NCRNA00292	non-protein coding RNA 292	NR_027285 /// NR_027286	0,0463133	0,0535555	0,0463133	1,144	1,144	rapid up vs Slow	0,194	4,143	2,035	4,143	0,565	2,480	1,352
20767	11735857_at	IFNE	interferon, epsilon	NM_176891	0,0463614	0,0787885	0,0463614	1,090	1,090	rapid up vs Slow	0,125	4,141	2,035	4,141	0,233	2,210	0,498
47669	11762759_at	---	---	---	0,0463882	0,402764	0,0463882	1,122	1,122	rapid up vs Slow	0,166	4,140	2,035	4,140	0,412	1,023	0,407
39394	11754484_a_at	COPS7B	COP9 constitutive photomorphogenic homolog subunit 7B (Arabidopsis)	NM_022730	0,0463929	0,161687	0,0463929	0,862	-1,161	rapid down vs Slow	-0,215	4,140	-2,035	4,140	0,691	1,702	1,136
22341	11737431_x_at	PAPL	iron/zinc purple acid phosphatase-like protein	NM_001004318	0,0464185	0,00979965	0,0464185	1,095	1,095	rapid up vs Slow	0,131	4,139	2,034	4,139	0,256	3,669	0,909
46991	11762081_at	DMC1	DMC1 dosage suppressor of mck1 homolog, meiosis-specific homologous recombination (yeas	NM_007068	0,0464586	0,0796721	0,0464586	1,074	1,074	rapid up vs Slow	0,102	4,137	2,034	4,137	0,157	2,202	0,334
24910	11740000_a_at	CPEB3	cytoplasmic polyadenylation element binding protein 3	NM_001178137 /// NM_014912	0,0464738	0,227062	0,0464738	0,864	-1,157	rapid down vs Slow	-0,211	4,136	-2,034	4,136	0,667	1,456	0,939
44664	11759754_a_at	LOC100507741	hypothetical LOC100507741	XR_111351 /// XR_111352 /// XR_111353 ///	0,0465124	0,0807772	0,0465124	1,074	1,074	rapid up vs Slow	0,103	4,135	2,033	4,135	0,158	2,193	0,334

				XR_111354 /// XR_114631 /// XR_114632 /// XR_													
5802	11720892_at	SOS1	son of sevenless homolog 1 (Drosophila)	NM_005633	0,0465695	0,405832	0,0465695	1,091	1,091	rapid up vs Slow	0,126	4,133	2,033	4,133	0,237	1,017	0,233
27367	11742457_s_at	PTBP1	polypyrimidine tract binding protein 1	NM_002819 /// NM_031990 /// NM_031991 /// NM_175847	0,0466058	0,216262	0,0466058	0,900	-1,112	rapid down vs Slow	-0,153	4,131	-2,033	4,131	0,349	1,492	0,504
26474	11741564_a_at	CNNM3	cyclin M3	NM_017623 /// NM_199078	0,0466065	0,113579	0,0466065	0,876	-1,142	rapid down vs Slow	-0,191	4,131	-2,033	4,131	0,547	1,953	1,035
21450	11736540_s_at	TMEM170A	transmembrane protein 170A	NM_145254	0,04665	0,00410119	0,04665	0,929	-1,077	rapid down vs Slow	-0,106	4,130	-2,032	4,130	0,170	4,290	0,706
32688	11747778_x_at	RAB6A	RAB6A, member RAS oncogene family	NM_002869 /// NM_198896	0,0466704	0,682616	0,0466704	0,913	-1,096	rapid down vs Slow	-0,132	4,129	-2,032	4,129	0,260	0,574	0,144
41166	11756256_a_at	FANCG	Fanconi anemia, complementation group G	NM_004629	0,0466715	0,104298	0,0466715	0,872	-1,146	rapid down vs Slow	-0,197	4,129	-2,032	4,129	0,582	2,013	1,135
5251	11720341_at	NDUFA13	NADH dehydrogenase (ubiquinone) 1 alpha subcomplex, 13	NM_015965	0,0467025	0,0854484	0,0467025	1,118	1,118	rapid up vs Slow	0,161	4,128	2,032	4,128	0,387	2,153	0,808
14498	11729588_at	KLK2	kallikrein-related peptidase 2	NM_001002231 /// NM_005551	0,0467134	0,477817	0,0467134	1,096	1,096	rapid up vs Slow	0,133	4,127	2,032	4,127	0,264	0,886	0,226
12356	11727446_a_at	PTPN5	protein tyrosine phosphatase, non-receptor type 5 (striatum-enriched)	NM_001039970 /// NM_006906 /// NM_032781	0,0467941	0,0142329	0,0467941	1,107	1,107	rapid up vs Slow	0,147	4,124	2,031	4,124	0,325	3,406	1,072
16642	11731732_x_at	CD1B	CD1b molecule	NM_001764	0,0468968	0,275264	0,0468968	1,110	1,110	rapid up vs Slow	0,151	4,120	2,030	4,120	0,340	1,314	0,434
28940	11744030_a_at	MMP7	matrix metallopeptidase 7 (matrilysin, uterine)	NM_002423	0,0468995	0,0418618	0,0468995	1,055	1,055	rapid up vs Slow	0,077	4,120	2,030	4,120	0,088	2,652	0,227
16990	11732080_a_at	FSD1L	fibronectin type III and SPRY domain containing 1-like	NM_001145313 /// NM_031919 /// NM_207647	0,0469203	0,494886	0,0469203	1,087	1,087	rapid up vs Slow	0,120	4,119	2,030	4,119	0,217	0,857	0,181
18083	11733173_at	C9orf100	chromosome 9 open reading frame 100	NM_032818	0,0469993	0,0243145	0,0469993	1,094	1,094	rapid up vs Slow	0,130	4,116	2,029	4,116	0,252	3,031	0,741
3157	11718247_a_at	KIAA0430	KIAA0430	NM_001184998 /// NM_001184999 /// NM_014647	0,0470436	0,00770198	0,0470436	0,869	-1,151	rapid down vs Slow	-0,202	4,114	-2,028	4,114	0,614	3,840	2,291
31492	11746582_a_at	ZNF558	zinc finger protein 558	NM_144693	0,0470541	0,077398	0,0470541	1,068	1,068	rapid up vs Slow	0,095	4,114	2,028	4,114	0,135	2,223	0,292
8198	11723288_at	CRISPLD1	cysteine-rich secretory protein LCCL domain containing 1	NM_031461	0,0471024	0,0468692	0,0471024	1,122	1,122	rapid up vs Slow	0,167	4,112	2,028	4,112	0,415	2,573	1,040
19392	11734482_at	ETV3L	ets variant 3-like	NM_001004341	0,0471204	0,102286	0,0471204	1,118	1,118	rapid up vs Slow	0,161	4,111	2,028	4,111	0,390	2,027	0,770
35114	11750204_x	TRUB1	TruB	NM_139169	0,047231	0,615802	0,0472316	1,075	1,075	rapid up vs	0,104	4,107	2,027	4,107	0,163	0,669	0,106

	_at		pseudouridine (psi) synthase homolog 1 (E. coli)		6					Slow							
48000	11763090_at	---	---	---	0,0472664	0,430232	0,0472664	1,084	1,084	rapid up vs Slow	0,117	4,106	2,026	4,106	0,204	0,971	0,193
44078	11759168_x_at	HIST1H2BE	histone cluster 1, H2be	NM_003523	0,0472868	0,624821	0,0472868	1,096	1,096	rapid up vs Slow	0,132	4,105	2,026	4,105	0,263	0,656	0,168
8055	11723145_a_at	RANBP17	RAN binding protein 17	NM_022897	0,0472969	0,0332796	0,0472969	1,065	1,065	rapid up vs Slow	0,091	4,105	2,026	4,105	0,123	2,812	0,338
19844	11734934_a_at	FGD2	FYVE, RhoGEF and PH domain containing 2	NM_173558	0,0473309	0,484405	0,0473309	0,897	-1,115	rapid down vs Slow	-0,157	4,103	-2,026	4,103	0,371	0,875	0,317
36856	11751946_a_at	ARHGAP21	Rho GTPase activating protein 21	NM_020824	0,0473313	0,599788	0,0473313	1,105	1,105	rapid up vs Slow	0,144	4,103	2,026	4,103	0,310	0,693	0,209
45746	11760836_at	---	---	---	0,0473644	0,147259	0,0473644	1,104	1,104	rapid up vs Slow	0,143	4,102	2,025	4,102	0,307	1,769	0,529
19144	11734234_at	HIST1H2AG	histone cluster 1, H2ag	NM_021064	0,047431	0,157884	0,047431	1,081	1,081	rapid up vs Slow	0,112	4,099	2,025	4,099	0,188	1,719	0,316
8246	11723336_a_at	GMIP	GEM interacting protein	NM_016573	0,0474355	0,0414181	0,0474355	1,084	1,084	rapid up vs Slow	0,117	4,099	2,025	4,099	0,204	2,659	0,529
33388	11748478_a_at	TAB1	TGF-beta activated kinase 1/MAP3K7 binding protein 1	NM_006116 /// NM_153497	0,0474587	0,270222	0,0474587	1,102	1,102	rapid up vs Slow	0,141	4,098	2,024	4,098	0,296	1,328	0,384
34040	11749130_a_at	TAF6	TAF6 RNA polymerase II, TATA box binding protein (TBP)-associated factor, 80kDa	NM_001190415 /// NM_005641 /// NM_139315 /// NR_033792	0,0475341	0,00746465	0,0475341	1,107	1,107	rapid up vs Slow	0,147	4,096	2,024	4,096	0,325	3,862	1,226
46860	11761950_x_at	NALCN	sodium leak channel, non-selective	NM_052867	0,0476183	0,231571	0,0476183	1,119	1,119	rapid up vs Slow	0,162	4,092	2,023	4,092	0,393	1,442	0,554
48548	11763638_x_at	RALGAPA1	Ral GTPase activating protein, alpha subunit 1 (catalytic)	NM_014990 /// NM_194301	0,047619	0,282477	0,047619	1,117	1,117	rapid up vs Slow	0,160	4,092	2,023	4,092	0,383	1,295	0,485
35746	11750836_a_at	EFHC1	EF-hand domain (C-terminal) containing 1	NM_001172420 /// NM_018100 /// NR_033327	0,0476275	0,217639	0,0476275	0,910	-1,099	rapid down vs Slow	-0,136	4,092	-2,023	4,092	0,277	1,487	0,402
43415	11758505_s_at	HOXD13	homeobox D13	NM_000523	0,0476283	0,178525	0,0476283	1,118	1,118	rapid up vs Slow	0,160	4,092	2,023	4,092	0,386	1,631	0,615
24053	11739143_a_at	VPS39	vacuolar protein sorting 39 homolog (S. cerevisiae)	NM_015289	0,0477178	0,00121316	0,0477178	0,903	-1,108	rapid down vs Slow	-0,147	4,089	-2,022	4,089	0,326	5,176	1,651
35137	11750227_a_at	RSAD1	radical S-adenosyl methionine domain containing 1	NM_018346	0,0477341	0,924497	0,0477341	1,111	1,111	rapid up vs Slow	0,152	4,088	2,022	4,088	0,344	0,223	0,075
19734	11734824_at	SPRED1	sprouty-related, EVH1 domain containing 1	NM_152594	0,047744	0,19251	0,047744	1,066	1,066	rapid up vs Slow	0,092	4,088	2,022	4,088	0,128	1,576	0,198
7011	11722101_a_at	TYRO3	TYRO3 protein tyrosine kinase	NM_006293	0,0477552	0,324389	0,0477552	1,095	1,095	rapid up vs Slow	0,131	4,087	2,022	4,087	0,258	1,191	0,301
11174	11726264_s_at	STRADB	STE20-related kinase adaptor beta	NM_018571	0,0477652	0,237386	0,0477652	0,730	-1,371	rapid down vs Slow	-0,455	4,087	-2,022	4,087	3,100	1,424	4,319
47267	11762357_x_at	RGPD4	RANBP2-like and GRIP	NM_182588	0,0477751	0,011409	0,0477751	0,839	-1,192	rapid down vs Slow	-0,254	4,086	-2,021	4,086	0,965	3,562	3,363

			domain containing 4														
14960	11730050_a_at	CA13	carbonic anhydrase XIII	NM_198584	0,0478241	0,138081	0,0478241	1,066	1,066	rapid up vs Slow	0,092	4,084	2,021	4,084	0,126	1,814	0,223
23752	11738842_a_at	ADRA1A	adrenergic, alpha-1A-, receptor	NM_000680 /// NM_033302 /// NM_033303 /// NM_033304	0,0479089	0,161319	0,0479089	1,129	1,129	rapid up vs Slow	0,175	4,081	2,020	4,081	0,459	1,703	0,766
4155	11719245_a_at	LARGE	like-glycosyltransferase	NM_004737 /// NM_133642	0,0479458	0,754231	0,0479458	1,100	1,100	rapid up vs Slow	0,138	4,080	2,020	4,080	0,286	0,475	0,133
19834	11734924_a_at	ATP6V1G2	ATPase, H+ transporting, lysosomal 13kDa, V1 subunit G2	NM_130463 /// NM_138282	0,0479806	0,246774	0,0479806	1,077	1,077	rapid up vs Slow	0,107	4,079	2,020	4,079	0,170	1,395	0,233
17686	11732776_a_at	DNMT3A	DNA (cytosine-5-)-methyltransferase 3 alpha	NM_022552 /// NM_153759 /// NM_175629 /// NM_175630	0,0480142	0,277038	0,0480142	0,868	-1,152	rapid down vs Slow	-0,204	4,077	-2,019	4,077	0,621	1,309	0,797
31419	11746509_a_at	TDG	thymine-DNA glycosylase	NM_003211	0,0480218	0,30294	0,0480218	1,067	1,067	rapid up vs Slow	0,093	4,077	2,019	4,077	0,131	1,242	0,159
36615	11751705_a_at	NOTCH4	notch 4	NM_004557	0,0480656	0,00719418	0,0480656	1,096	1,096	rapid up vs Slow	0,133	4,075	2,019	4,075	0,264	3,888	1,006
5952	11721042_at	NSMCE2	non-SMC element 2, MMS21 homolog (S. cerevisiae)	NM_173685	0,048097	0,0762153	0,048097	0,865	-1,156	rapid down vs Slow	-0,209	4,074	-2,018	4,074	0,654	2,233	1,435
43986	11759076_at	HIST1H3H	histone cluster 1, H3h	NM_003536	0,0481203	0,6992	0,0481203	1,123	1,123	rapid up vs Slow	0,168	4,073	2,018	4,073	0,422	0,551	0,228
43285	11758375_s_at	RNF20	ring finger protein 20	NM_019592	0,0481792	0,300327	0,0481792	0,825	-1,212	rapid down vs Slow	-0,278	4,071	-2,018	4,071	1,157	1,249	1,420
21418	11736508_at	MAGEE2	melanoma antigen family E, 2	NM_138703	0,0481845	0,125199	0,0481845	1,069	1,069	rapid up vs Slow	0,096	4,071	2,018	4,071	0,139	1,884	0,257
45564	11760654_a_at	LRP3	low density lipoprotein receptor-related protein 3	NM_002333	0,048223	0,0222955	0,048223	1,111	1,111	rapid up vs Slow	0,152	4,069	2,017	4,069	0,344	3,092	1,047
41525	11756615_x_at	SHMT2	serine hydroxymethyltransferase 2 (mitochondrial)	NM_001166356 /// NM_001166357 /// NM_001166358 /// NM_001166359 /// NM_005412 /// NR_02	0,0482517	0,453574	0,0482517	0,830	-1,205	rapid down vs Slow	-0,268	4,068	-2,017	4,068	1,080	0,929	0,986
37053	11752143_a_at	ABCB11	ATP-binding cassette, sub-family B (MDR/TAP), member 11	NM_003742	0,0482695	0,00814312	0,0482695	1,075	1,075	rapid up vs Slow	0,105	4,068	2,017	4,068	0,165	3,800	0,616
15021	11730111_a_at	DEPDC1	DEP domain containing 1	NM_001114120 /// NM_017779	0,048304	0,152615	0,048304	1,064	1,064	rapid up vs Slow	0,089	4,066	2,017	4,066	0,119	1,743	0,204
19323	11734413_a_at	TRIM23	tripartite motif-containing 23	NM_001656 /// NM_033227	0,0483516	0,543087	0,0483516	0,917	-1,090	rapid down vs Slow	-0,125	4,065	-2,016	4,065	0,234	0,779	0,179

				/// NM_033228													
38677	11753767_a_at	ZBTB4	zinc finger and BTB domain containing 4	NM_0011288 33 /// NM_020899	0,048415 3	0,050783 6	0,0484153	1,124	1,124	rapid up vs Slow	0,169	4,062	2,015	4,062	0,427	2,517	1,058
41837	11756927_a_at	SMARCA L1	SWI/SNF related, matrix associated, actin dependent regulator of chromatin, subfamily a	NM_0011272 07 /// NM_014140	0,048459 7	0,089326 1	0,0484597	0,880	-1,136	rapid down vs Slow	-0,184	4,061	-2,015	4,061	0,507	2,122	1,059
13579	11728669_a_at	UBE2H	ubiquitin-conjugating enzyme E2H (UBC8 homolog, yeast)	NM_003344 /// NM_182697	0,048523 1	0,069494 3	0,0485231	0,791	-1,264	rapid down vs Slow	-0,338	4,058	-2,015	4,058	1,709	2,298	3,872
13358	11728448_a_at	ADCK5	aarF domain containing kinase 5	NM_174922	0,048584 2	0,495905	0,0485842	0,882	-1,134	rapid down vs Slow	-0,182	4,056	-2,014	4,056	0,494	0,856	0,417
12825	11727915_a_at	NCAM1	neural cell adhesion molecule 1	NM_000615 /// NM_0010766 82 /// NM_181351	0,048705 9	0,171293	0,0487059	1,071	1,071	rapid up vs Slow	0,099	4,051	2,013	4,051	0,148	1,660	0,242
21970	11737060_a_at	MTMR3	myotubularin related protein 3	NM_021090 /// NM_153050 /// NM_153051	0,048741 2	0,761037	0,0487412	0,917	-1,091	rapid down vs Slow	-0,125	4,050	-2,012	4,050	0,234	0,465	0,108
32967	11748057_x_at	SOX15	SRY (sex determining region Y)-box 15	NM_006942	0,048754 3	0,027166	0,0487543	1,088	1,088	rapid up vs Slow	0,121	4,050	2,012	4,050	0,220	2,954	0,642
38412	11753502_x_at	PTCRA	pre T-cell antigen receptor alpha	NM_138296	0,048772 6	0,013096 8	0,0487726	1,111	1,111	rapid up vs Slow	0,152	4,049	2,012	4,049	0,344	3,465	1,178
37130	11752220_a_at	VAV3	vav 3 guanine nucleotide exchange factor	NM_0010798 74 /// NM_006113	0,048865 8	0,302888	0,0488658	1,073	1,073	rapid up vs Slow	0,101	4,045	2,011	4,045	0,154	1,243	0,189
20010	11735100_a_at	C2orf73	chromosome 2 open reading frame 73	NM_0011003 96	0,048889 4	0,153345	0,0488894	1,074	1,074	rapid up vs Slow	0,103	4,045	2,011	4,045	0,159	1,740	0,273
28620	11743710_a_at	RGS22	regulator of G-protein signaling 22	NM_015668	0,048891	0,255161	0,048891	1,086	1,086	rapid up vs Slow	0,119	4,045	2,011	4,045	0,210	1,370	0,285
11877	11726967_a_at	COL22A1	collagen, type XXII, alpha 1	NM_152888	0,048903 9	0,2361	0,0489039	1,104	1,104	rapid up vs Slow	0,142	4,044	2,011	4,044	0,303	1,428	0,428
29660	11744750_a_at	LGI4	leucine-rich repeat LGI family, member 4	NM_139284	0,048911 6	0,257666	0,0489116	1,106	1,106	rapid up vs Slow	0,145	4,044	2,011	4,044	0,317	1,363	0,427
40059	11755149_x_at	MEF2D	myocyte enhancer factor 2D	NM_005920	0,048986 5	0,750668	0,0489865	0,890	-1,124	rapid down vs Slow	-0,169	4,041	-2,010	4,041	0,426	0,479	0,202
44060	11759150_at	CNOT4	CCR4-NOT transcription complex, subunit 4	NM_0010082 25 /// NM_0011908 47 /// NM_0011908 48 /// NM_0011908 49 /// NM_0011908 50 /// NM	0,049040 1	0,189208	0,0490401	0,875	-1,142	rapid down vs Slow	-0,192	4,039	-2,010	4,039	0,552	1,589	0,868
41480	11756570_a_at	TMEM50 B	transmembrane protein 50B	NM_006134	0,049049 6	0,206333	0,0490496	0,840	-1,191	rapid down vs Slow	-0,252	4,039	-2,010	4,039	0,952	1,526	1,438

26656	11741746_a_at	LIN54	lin-54 homolog (C. elegans)	NM_001115007 /// NM_001115008 /// NM_194282	0,0490767	0,431775	0,0490767	1,092	1,092	rapid up vs Slow	0,126	4,038	2,009	4,038	0,239	0,968	0,229
7227	11722317_a_at	PTPN13	protein tyrosine phosphatase, non-receptor type 13 (APO-1/CD95 (Fas)-associated phospha	NM_006264 /// NM_080683 /// NM_080684 /// NM_080685	0,04912	0,00635695	0,04912	1,078	1,078	rapid up vs Slow	0,108	4,036	2,009	4,036	0,174	3,976	0,686
28654	11743744_at	FAM26E	family with sequence similarity 26, member E	NM_153711	0,0491417	0,635435	0,0491417	1,098	1,098	rapid up vs Slow	0,134	4,035	2,009	4,035	0,271	0,641	0,172
15302	11730392_at	GPR4	G protein-coupled receptor 4	NM_005282	0,0491518	0,29612	0,0491518	1,109	1,109	rapid up vs Slow	0,149	4,035	2,009	4,035	0,332	1,260	0,414
20131	11735221_a_at	BTLA	B and T lymphocyte associated	NM_001085357 /// NM_181780	0,0491756	0,0703877	0,0491756	0,844	-1,185	rapid down vs Slow	-0,245	4,034	-2,008	4,034	0,901	2,289	2,046
26007	11741097_a_at	C10orf26	chromosome 10 open reading frame 26	NM_001083913 /// NM_017787	0,0492354	0,00683902	0,0492354	1,110	1,110	rapid up vs Slow	0,150	4,032	2,008	4,032	0,338	3,924	1,315
35644	11750734_a_at	ZNF782	zinc finger protein 782	NM_001001662	0,0492958	0,134771	0,0492958	1,085	1,085	rapid up vs Slow	0,117	4,030	2,007	4,030	0,207	1,832	0,376
45040	11760130_a_at	HDAC8	histone deacetylase 8	NM_001166418 /// NM_001166419 /// NM_001166420 /// NM_001166422 /// NM_001166448 /// NM	0,0493154	0,0629483	0,0493154	1,085	1,085	rapid up vs Slow	0,118	4,029	2,007	4,029	0,208	2,367	0,489
36647	11751737_s_at	C1orf91	chromosome 1 open reading frame 91	NM_019118	0,0493163	0,195618	0,0493163	1,105	1,105	rapid up vs Slow	0,144	4,029	2,007	4,029	0,310	1,565	0,482
20429	11735519_a_at	ART5	ADP-ribosyltransferase 5	NM_001079536 /// NM_053017	0,049361	0,0293481	0,049361	1,082	1,082	rapid up vs Slow	0,114	4,027	2,007	4,027	0,195	2,900	0,562
9919	11725009_a_at	EPB42	erythrocyte membrane protein band 4.2	NM_000119 /// NM_001114134	0,0493689	0,103338	0,0493689	0,697	-1,435	rapid down vs Slow	-0,521	4,027	-2,007	4,027	4,061	2,020	8,146
13582	11728672_x_at	UBE2H	ubiquitin-conjugating enzyme E2H (UBC8 homolog, yeast)	NM_003344 /// NM_182697	0,0493851	0,509737	0,0493851	0,806	-1,241	rapid down vs Slow	-0,311	4,026	-2,007	4,026	1,452	0,833	1,201
2951	11718041_at	SNRPE	small nuclear ribonucleoprotein polypeptide E	NM_003094	0,0493894	0,266228	0,0493894	0,928	-1,078	rapid down vs Slow	-0,108	4,026	-2,007	4,026	0,175	1,339	0,233
1112	11716202_a_at	PFKM	phosphofructokinase, muscle	NM_000289 /// NM_001166686 /// NM_001166687 /// NM_001166688	0,0493971	0,379328	0,0493971	0,850	-1,176	rapid down vs Slow	-0,234	4,026	-2,006	4,026	0,821	1,070	0,873
34986	11750076_a_at	SNAP25	synaptosomal-associated protein, 25kDa	NM_003081 /// NM_130811	0,049403	0,186535	0,049403	1,066	1,066	rapid up vs Slow	0,092	4,026	2,006	4,026	0,126	1,599	0,200

25160	11740250_a_at	SPTLC1	serine palmitoyltransferase, long chain base subunit 1	NM_006415 /// NM_178324	0,0494191	0,0337494	0,0494191	0,849	-1,178	rapid down vs Slow	-0,236	4,025	-2,006	4,025	0,837	2,802	2,331
17201	11732291_a_at	FAM186B	family with sequence similarity 186, member B	NM_032130 /// NR_027450	0,0494209	0,0979732	0,0494209	1,123	1,123	rapid up vs Slow	0,167	4,025	2,006	4,025	0,419	2,057	0,856
31483	11746573_s_at	RHOB	ras homolog gene family, member B	NM_004040	0,0494251	0,241725	0,0494251	0,906	-1,104	rapid down vs Slow	-0,142	4,025	-2,006	4,025	0,303	1,410	0,425
25307	11740397_at	SSTR3	somatostatin receptor 3	NM_001051	0,049453	0,0769676	0,049453	1,109	1,109	rapid up vs Slow	0,150	4,024	2,006	4,024	0,335	2,226	0,742
138	11715228_s_at	OSTBETA	organic solute transporter beta	NM_178859	0,049468	0,175279	0,049468	1,102	1,102	rapid up vs Slow	0,140	4,023	2,006	4,023	0,295	1,644	0,482
42560	11757650_s_at	FN1	fibronectin 1	NM_002026 /// NM_054034 /// NM_212474 /// NM_212476 /// NM_212478 /// NM_212482	0,0494689	0,00309209	0,0494689	1,070	1,070	rapid up vs Slow	0,098	4,023	2,006	4,023	0,144	4,493	0,644
48715	11763805_a_at	LSM14B	LSM14B, SCD6 homolog B (S. cerevisiae)	NM_144703	0,0494744	0,253666	0,0494744	1,079	1,079	rapid up vs Slow	0,110	4,023	2,006	4,023	0,182	1,375	0,249
26584	11741674_a_at	NTRK1	neurotrophic tyrosine kinase, receptor, type 1	NM_001007792 /// NM_001012331 /// NM_002529	0,0494824	0,0570036	0,0494824	1,091	1,091	rapid up vs Slow	0,126	4,023	2,006	4,023	0,237	2,436	0,574
8995	11724085_at	DAPL1	death associated protein-like 1	NM_001017920	0,0494937	0,0119423	0,0494937	1,096	1,096	rapid up vs Slow	0,132	4,022	2,006	4,022	0,262	3,530	0,921
35944	11751034_a_at	NLE1	notchless homolog 1 (Drosophila)	NM_001014445 /// NM_018096	0,0496005	0,0732132	0,0496005	0,879	-1,138	rapid down vs Slow	-0,187	4,018	-2,005	4,018	0,521	2,261	1,174
11431	11726521_a_at	SPATA17	spermatogenesis associated 17	NM_138796	0,0496017	0,156847	0,0496017	1,082	1,082	rapid up vs Slow	0,113	4,018	2,005	4,018	0,192	1,724	0,329
25173	11740263_a_at	ETV3	ets variant 3	NM_001145312 /// NM_005240	0,0496328	0,368268	0,0496328	1,095	1,095	rapid up vs Slow	0,131	4,017	2,004	4,017	0,255	1,093	0,278
26274	11741364_a_at	KCNJ1	potassium inwardly-rectifying channel, subfamily J, member 1	NM_000220 /// NM_153764 /// NM_153765 /// NM_153766 /// NM_153767	0,0496498	0,0190036	0,0496498	1,086	1,086	rapid up vs Slow	0,119	4,017	2,004	4,017	0,211	3,203	0,673
49177	11764267_at	---	---	---	0,0496645	0,523472	0,0496645	1,090	1,090	rapid up vs Slow	0,125	4,016	2,004	4,016	0,233	0,811	0,188
40565	11755655_a_at	MICAL3	microtubule associated monoxygenase, calponin and LIM domain containing 3	NM_001122731 /// NM_001136004 /// NM_015241	0,0496693	0,063223	0,0496693	1,071	1,071	rapid up vs Slow	0,099	4,016	2,004	4,016	0,146	2,364	0,344
11233	11726323_a_at	ACOX1	acyl-CoA oxidase 1, palmitoyl	NM_001185039 /// NM_004035 /// NM_007292	0,0496786	0,613663	0,0496786	0,802	-1,247	rapid down vs Slow	-0,319	4,016	-2,004	4,016	1,522	0,673	1,019

19005	11734095_x_at	ZNF845	zinc finger protein 845	NM_138374	0,0496911	0,00159337	0,0496911	1,076	1,076	rapid up vs Slow	0,106	4,015	2,004	4,015	0,167	4,976	0,829
36124	11751214_a_at	RGS7	regulator of G-protein signaling 7	NM_002924	0,0497393	0,824488	0,0497393	1,070	1,070	rapid up vs Slow	0,097	4,013	2,003	4,013	0,141	0,377	0,053
290	11715380_s_at	LAPTM4B	lysosomal protein transmembrane 4 beta	NM_018407	0,049752	0,0831133	0,049752	0,816	-1,225	rapid down vs Slow	-0,293	4,013	-2,003	4,013	1,288	2,173	2,790
16357	11731447_x_at	SNCA	synuclein, alpha (non A4 component of amyloid precursor)	NM_000345 /// NM_001146054 /// NM_001146055 /// NM_007308	0,0497756	0,125622	0,0497756	0,699	-1,432	rapid down vs Slow	-0,518	4,012	-2,003	4,012	4,013	1,882	7,529
46336	11761426_a_at	PHLDB1	pleckstrin homology-like domain, family B, member 1	NM_001144758 /// NM_001144759 /// NM_015157	0,0497884	0,037537	0,0497884	1,082	1,082	rapid up vs Slow	0,114	4,012	2,003	4,012	0,193	2,728	0,526
20921	11736011_s_at	MGAT2	mannosyl (alpha-1,6-)-glycoprotein beta-1,2-N-acetylglucosaminyltransferase	NM_002408	0,0498016	0,314827	0,0498016	1,085	1,085	rapid up vs Slow	0,118	4,011	2,003	4,011	0,208	1,213	0,251
7984	11723074_x_at	BCL9L	B-cell CLL/lymphoma 9-like	NM_182557	0,0498047	0,294768	0,0498047	0,900	-1,111	rapid down vs Slow	-0,152	4,011	-2,003	4,011	0,346	1,263	0,435
45762	11760852_at	TRIML1	tripartite motif family-like 1	NM_178556	0,0498713	0,602917	0,0498713	1,064	1,064	rapid up vs Slow	0,090	4,009	2,002	4,009	0,121	0,688	0,083
15672	11730762_s_at	CRIPT	cysteine-rich PDZ-binding protein	NM_014171	0,0499437	0,395538	0,0499437	1,086	1,086	rapid up vs Slow	0,119	4,006	2,002	4,006	0,213	1,038	0,221
19266	11734356_a_at	DRP2	dystrophin related protein 2	NM_001171184 /// NM_001939	0,049956	0,280159	0,049956	1,06996	1,06996	rapid up vs Slow	0,0975634	4,00557	2,00139	4,00557	0,142623	1,30098	0,185292
