# Supplementary material for: Gene Expression Differences in Peripheral Blood of Parkinson’s Disease Patients with Distinct Progression Profiles
Source: PLoS One. 2016 Jun 20;11(6):e0157852. doi: 10.1371/journal.pone.0157852 (PMC4913914; doi:10.1371/journal.pone.0157852)
Supplement: S5 Table — The analysis was conducted on averaged gene expression values based on the summarized and normalized expression data following covariate correction. (PDF) [file pone.0157852.s010.pdf]

S5 Table. Genes differentially expressed detected by bootstrapping (n=1000 iterations) t-test having p<0.005. The analysis was conducted on averaged gene expression values based on the summarized and normalized expression data following covariate correction.

Gene Symbol	*p-value*	T score	Degrees Freedom
FBXO11	0.0050	62.7438	2.8997
TCEA1	0.0050	50.0802	2.8999
CTRC	0.0050	65.9558	-2.9003
PCBP3	0.0050	63.4353	-2.9016
NCRNA00277	0.0050	60.7746	-2.9021
SEC24B	0.0050	56.1323	2.9023
ZUFSP	0.0050	60.8928	2.9024
FYTTD1	0.0049	51.9790	2.9030
GPR52	0.0049	65.2054	-2.9031
UFSP2	0.0049	63.9278	2.9033
PDCD10	0.0049	48.2445	2.9034
ART1	0.0049	65.6976	-2.9036
TMCO1	0.0049	48.0334	2.9038
ZNF282	0.0049	65.9757	-2.9046
NAPG	0.0049	51.8755	2.9053
C20orf30	0.0049	56.2500	2.9054
FAM13B	0.0049	58.8007	2.9054
DCBLD1	0.0049	63.8904	-2.9055
FLJ16126	0.0049	65.3787	-2.9056
SH3BP5L	0.0049	65.9129	-2.9060
POP5	0.0049	65.7527	2.9061
SYNCRIP	0.0049	64.0525	2.9067
ARPC5L	0.0049	65.3073	2.9068
LYRM7	0.0049	58.5641	2.9071
CHMP2B	0.0049	62.0334	2.9071
KLRF1	0.0049	43.0991	2.9073
KCND2	0.0049	65.6507	-2.9094
QRSL1	0.0049	62.6596	2.9095
NOTCH1	0.0048	65.0428	-2.9115
SH3YL1	0.0048	57.4684	2.9125
RBM7	0.0048	53.5971	2.9126
SF3B5	0.0048	64.9109	2.9126
STX2	0.0048	49.6353	2.9127
BCCIP	0.0048	65.9373	2.9129
ATP10D	0.0048	48.8775	2.9132

Gene Symbol	*p-value*	T score	Degrees Freedom
SELT	0.0048	46.5235	2.9133
MATR3	0.0048	42.7760	2.9136
UBQLN1	0.0048	52.8950	2.9138
AGPAT1	0.0048	64.3404	-2.9145
TBCK	0.0048	65.9992	2.9148
LOC390940	0.0048	64.5361	-2.9151
CLNS1A	0.0048	58.5309	2.9151
KATNB1	0.0048	63.9486	-2.9154
SLC36A4	0.0048	59.2274	2.9156
WDR36	0.0047	55.4005	2.9171
FAM153A	0.0047	62.8346	-2.9171
LTV1	0.0047	61.1021	2.9176
MRPL32	0.0047	51.1908	2.9181
SLC4A5	0.0047	63.3766	-2.9187
ATAD1	0.0047	47.5735	2.9188
SLC25A47	0.0047	62.0249	-2.9193
TMEM175	0.0047	61.3961	-2.9196
RAB28	0.0047	47.4366	2.9202
TDG	0.0047	65.4321	2.9223
ANKIB1	0.0047	57.8950	2.9223
GPR97	0.0047	65.5049	-2.9229
FAM181A	0.0046	64.7793	-2.9243
SYTL2	0.0046	60.4831	2.9248
CPNE4	0.0046	65.9976	-2.9250
ALPP	0.0046	63.0956	-2.9250
INTS2	0.0046	64.4906	2.9252
PSMA7	0.0046	65.5593	2.9254
OR8D1	0.0046	62.9737	-2.9255
B3GNT6	0.0046	62.1158	-2.9256
ZZZ3	0.0046	61.6054	2.9256
DNAJC19	0.0046	57.4663	2.9260
ERGIC2	0.0046	58.3581	2.9263
R3HDM1	0.0046	63.6308	2.9269
EID2	0.0046	65.6268	2.9273
DDX1	0.0046	61.0690	2.9280

Gene Symbol	*p-value*	T score	Degrees Freedom
UBR1	0.0046	64.4625	2.9280
C20orf123	0.0046	65.9980	-2.9281
PLEKHA3	0.0046	58.9061	2.9282
TROVE2	0.0046	59.2895	2.9286
TCEB1	0.0046	63.0118	2.9287
ZNF202	0.0046	61.3168	2.9290
MCM5	0.0046	64.9758	-2.9290
LRPPRC	0.0046	60.5696	2.9294
OSGIN1	0.0046	64.9587	-2.9300
SLC7A3	0.0046	65.9852	-2.9301
ANKRD36	0.0046	65.9923	2.9306
ARF4	0.0046	58.8207	2.9314
RIN3	0.0045	64.0170	-2.9320
SAR1B	0.0045	49.4637	2.9327
SORL1	0.0045	65.9567	-2.9329
MAP3K7	0.0045	53.6295	2.9331
HYLS1	0.0045	65.6064	2.9333
LCE2B	0.0045	62.5800	-2.9346
ZNF655	0.0045	64.0391	2.9347
OTX1	0.0045	59.8086	-2.9351
TMEM14A	0.0045	64.1675	2.9357
RPS11	0.0045	65.3456	2.9359
CHD9	0.0045	45.4256	2.9361
PGRMC1	0.0045	42.9112	2.9386
VPS29	0.0044	49.3744	2.9397
RPL26	0.0044	57.2095	2.9399
CLDND1	0.0044	51.1521	2.9405
PIK3C2G	0.0044	65.8985	-2.9405
MAP7D1	0.0044	64.5609	-2.9414
FAM149B1	0.0044	62.3922	2.9419
RASSF5	0.0044	64.7263	-2.9419
TF	0.0044	55.3565	-2.9423
NALCN	0.0044	64.5383	-2.9437
LOC100130071	0.0044	65.9503	-2.9441
SLC39A1	0.0044	60.7833	-2.9445
PLEKHF2	0.0044	44.7999	2.9446
WBP4	0.0044	65.1759	2.9450
NT5DC1	0.0044	64.9475	2.9451
DCAF4L2	0.0044	65.5748	-2.9451
ACSL4	0.0044	50.0527	2.9453
EPM2AIP1	0.0044	64.3354	2.9454
ZCCHC17	0.0044	65.6856	2.9459
HCLS1	0.0044	61.9042	-2.9460

Gene Symbol	*p-value*	T score	Degrees Freedom
TBC1D9	0.0044	46.7877	2.9461
MRPL33	0.0044	64.7784	2.9466
SLC9A1	0.0044	62.3600	-2.9470
LOC150786	0.0043	59.2917	2.9476
DES	0.0043	65.7587	-2.9478
LGALS8	0.0043	65.9926	2.9480
USH1G	0.0043	65.8606	-2.9482
FASTKD3	0.0043	60.0572	2.9482
NASP	0.0043	63.9095	2.9483
CPNE1	0.0043	65.9011	-2.9485
C8orf44	0.0043	62.6218	2.9491
BTG3	0.0043	65.1782	2.9491
USPL1	0.0043	59.0556	2.9507
PLCB2	0.0043	65.4001	-2.9513
CCDC6	0.0043	61.9166	2.9514
BDH2	0.0043	56.3944	2.9517
DCLRE1C	0.0043	65.2218	2.9522
GLT8D1	0.0043	64.2824	2.9523
FRG1	0.0043	65.9198	2.9527
NUP54	0.0043	51.4031	2.9531
GTF2F2	0.0043	58.8706	2.9532
TMEM123	0.0043	43.4891	2.9535
CHPF	0.0043	65.8955	-2.9543
IDH3A	0.0042	58.3262	2.9555
BPIL1	0.0042	48.9514	-2.9559
CWC27	0.0042	62.7995	2.9566
ATP2A2	0.0042	65.0486	2.9578
ARMC10	0.0042	64.5194	2.9581
KRTAP10-5	0.0042	64.3298	-2.9585
LENEP	0.0042	64.2528	-2.9602
FHL3	0.0042	65.1143	-2.9605
LSM14A	0.0042	57.1410	2.9606
PLDN	0.0042	53.4677	2.9613
PDZD11	0.0042	65.4635	2.9623
PIK3CD	0.0042	65.9041	-2.9628
TFB2M	0.0041	60.2195	2.9633
UBE2E3	0.0041	56.0336	2.9638
EPS15	0.0041	55.9766	2.9639
LPPR4	0.0041	64.9595	-2.9650
OSTM1	0.0041	47.4206	2.9651
CCL14	0.0041	62.5661	-2.9652
CLINT1	0.0041	54.1029	2.9653
MAP7D3	0.0041	60.3362	2.9657

Gene Symbol	*p-value*	T score	Degrees Freedom
TRPC7	0.0041	62.6899	-2.9663
DDHD2	0.0041	62.9144	2.9665
IYD	0.0041	65.0492	-2.9671
SEPHS1	0.0041	63.0425	2.9673
PQLC3	0.0041	55.7568	2.9674
OR2T6	0.0041	56.9531	-2.9679
PDCD4	0.0041	62.5535	2.9685
SCAMP1	0.0041	53.4885	2.9688
RNF7	0.0041	63.2892	2.9701
LEPREL2	0.0041	60.7497	-2.9702
DIRC1	0.0041	61.2098	-2.9712
LOC100131217	0.0040	65.3006	-2.9718
KIAA0319L	0.0040	65.6570	-2.9718
COPS4	0.0040	58.8519	2.9722
ATG5	0.0040	46.4964	2.9729
DPY30	0.0040	59.8164	2.9730
SLC6A12	0.0040	63.4554	-2.9739
NUDT19	0.0040	53.7469	2.9739
NR1D1	0.0040	64.5586	-2.9740
OR4F15	0.0040	65.5957	-2.9745
SULT1C2	0.0040	65.2868	-2.9746
CSNK1A1	0.0040	61.8881	2.9750
CAPN2	0.0040	61.3292	2.9751
GFM1	0.0040	58.4577	2.9753
GIMAP4	0.0040	60.6562	2.9754
PPP1CC	0.0040	49.2826	2.9755
SMC5	0.0040	65.1165	2.9776
USP14	0.0040	56.3668	2.9776
C1orf70	0.0040	63.5914	-2.9777
LIPT1	0.0040	62.2916	2.9792
EFR3A	0.0040	50.7794	2.9794
STK10	0.0039	60.6999	-2.9805
GALNT5	0.0039	65.1635	-2.9810
CCDC91	0.0039	65.8277	2.9811
BANP	0.0039	65.9933	-2.9822
CALM2	0.0039	54.5344	2.9827
CYP2C19	0.0039	65.9999	-2.9835
PTGDR	0.0039	50.2317	2.9841
FSTL3	0.0039	64.1680	-2.9843
OTUD3	0.0039	65.6984	2.9850
HMGN3	0.0039	62.8488	2.9851
MRPS23	0.0039	62.1416	2.9852
TIPRL	0.0039	61.5240	2.9854

Gene Symbol	*p-value*	T score	Degrees Freedom
KLHL6	0.0039	61.7260	2.9855
NEDD1	0.0039	49.8163	2.9856
API5	0.0039	47.9470	2.9857
AGPS	0.0039	51.2167	2.9865
PPIA	0.0039	60.4116	2.9865
STAM2	0.0039	56.3793	2.9866
LOC100287098	0.0038	55.4551	-2.9889
LACTB2	0.0038	62.7390	2.9897
IPO5	0.0038	62.5817	2.9899
INHA	0.0038	64.9795	-2.9900
KIFAP3	0.0038	58.3040	2.9902
RAP2C	0.0038	43.2962	2.9922
UFM1	0.0038	48.9672	2.9923
PBX3	0.0038	65.4331	2.9931
CCT2	0.0038	58.6504	2.9934
NOL11	0.0038	62.7315	2.9937
CSF3	0.0038	61.5800	-2.9939
MCM8	0.0038	61.4783	2.9940
ARID4A	0.0038	51.7853	2.9943
PLAA	0.0038	65.9998	2.9959
LOC139201	0.0038	61.0185	2.9961
RINT1	0.0037	58.6397	2.9976
AK3	0.0037	54.8882	2.9977
IDI1	0.0037	49.8557	2.9979
LOC100289424	0.0037	65.4369	-2.9980
B3GNT3	0.0037	64.1317	-2.9995
OPA1	0.0037	53.4700	2.9996
PSMG4	0.0037	61.3671	3.0004
FAM189A1	0.0037	64.9094	-3.0008
GNPTAB	0.0037	60.1600	3.0009
RAB4A	0.0037	63.3164	3.0016
MRPL18	0.0037	62.9525	3.0018
RPL13A	0.0037	65.8973	3.0023
C14orf142	0.0037	53.2550	3.0028
RTCD1	0.0037	54.1854	3.0029
FAM35A	0.0037	47.9025	3.0029
TNNT2	0.0037	65.4147	-3.0030
PYHIN1	0.0037	60.4380	3.0038
FBXO30	0.0037	54.6074	3.0039
ZNF213	0.0037	61.9347	-3.0047
MDH1	0.0036	60.5889	3.0062
DENR	0.0036	57.3370	3.0065
PIGY	0.0036	57.2496	3.0083

Gene Symbol	*p-value*	T score	Degrees Freedom
PTGES3	0.0036	53.8829	3.0086
C7orf23	0.0036	51.1179	3.0098
TAS2R38	0.0036	63.5274	-3.0116
BARD1	0.0036	66.0000	3.0118
NNT	0.0036	53.1966	3.0129
HSPD1	0.0036	59.3799	3.0134
SCYL2	0.0036	49.7458	3.0139
CD164	0.0036	47.2346	3.0147
DNAJB14	0.0036	54.5976	3.0148
DIMT1L	0.0035	59.5284	3.0152
FIBCD1	0.0035	60.2296	-3.0156
ZNF277	0.0035	60.0084	3.0157
TBC1D15	0.0035	53.7259	3.0162
TMEM181	0.0035	54.9037	3.0179
ALG13	0.0035	50.7642	3.0180
CCDC74A	0.0035	63.8726	-3.0181
ABCG8	0.0035	66.0000	-3.0184
TUBE1	0.0035	54.5558	3.0196
LRP8	0.0035	60.4618	3.0196
CACYBP	0.0035	61.4156	3.0217
PION	0.0035	52.0244	3.0219
GLUD1	0.0035	63.7162	3.0238
SEC61G	0.0035	58.2636	3.0240
RNF138	0.0035	49.3550	3.0244
C6orf130	0.0034	61.2961	3.0248
LOC100134391	0.0034	63.4355	-3.0257
SNRPD1	0.0034	54.5874	3.0262
EPS8L3	0.0034	65.8347	-3.0274
ZNF561	0.0034	62.6258	3.0280
CILP2	0.0034	60.6261	-3.0281
C18orf25	0.0034	63.7632	3.0286
IKZF5	0.0034	52.4384	3.0289
PLA2G12A	0.0034	63.3273	3.0295
TNFRSF11B	0.0034	62.8746	-3.0303
SRBD1	0.0034	63.6721	3.0309
CBX3	0.0034	52.6195	3.0309
WDSUB1	0.0034	63.6910	3.0318
CSE1L	0.0034	59.9395	3.0328
SLC12A4	0.0034	60.5904	-3.0331
TRAM1	0.0033	46.4788	3.0369
XPO6	0.0033	65.3437	-3.0371
DHX37	0.0033	64.8319	-3.0375
FEM1C	0.0033	50.9627	3.0375

Gene Symbol	*p-value*	T score	Degrees Freedom
SERPINI1	0.0033	65.6514	3.0388
FAM114A2	0.0033	64.7568	3.0389
HSPA9	0.0033	61.8435	3.0393
TPRKB	0.0033	56.6787	3.0399
DPF1	0.0033	65.8932	-3.0404
CHRNA4	0.0033	65.9728	-3.0409
TPD52	0.0033	43.0695	3.0417
SEPT12	0.0033	60.6040	-3.0419
NOP16	0.0033	65.7299	3.0426
RAP1A	0.0033	49.9718	3.0429
IAH1	0.0033	61.0418	3.0437
CDH2	0.0032	65.0759	-3.0454
LOC653712	0.0032	64.2055	-3.0466
OR5K3	0.0032	65.9965	-3.0468
ATPAF1	0.0032	60.8748	3.0470
CNO	0.0032	65.8579	3.0473
CUL5	0.0032	41.9317	3.0481
CARKD	0.0032	65.9185	3.0482
NRAP	0.0032	63.8152	-3.0508
SRP19	0.0032	55.4829	3.0509
ATP5L	0.0032	60.2778	3.0518
RYK	0.0032	62.5578	3.0523
PDCD5	0.0032	49.3311	3.0529
UBE2E1	0.0032	56.3407	3.0532
ZNF664	0.0032	50.6410	3.0534
TOMM70A	0.0032	53.3068	3.0535
SRP72	0.0032	63.7821	3.0552
PIK3CA	0.0031	58.0446	3.0568
TP53INP2	0.0031	65.7985	-3.0572
MRPS16	0.0031	64.4214	3.0572
ATXN3	0.0031	64.2288	3.0583
DCHS1	0.0031	49.4880	-3.0587
TSNAX	0.0031	55.1277	3.0601
TMED10	0.0031	56.3647	3.0604
GHITM	0.0031	65.4627	3.0612
C1orf25	0.0031	59.5105	3.0618
C2orf69	0.0031	50.3890	3.0625
PDE12	0.0031	51.4063	3.0626
TOP2B	0.0031	60.8774	3.0631
TAS2R30	0.0031	64.0195	-3.0633
RASGRP1	0.0031	62.9975	3.0649
CASC3	0.0031	65.6323	-3.0666
SSX7	0.0030	65.4769	-3.0668

Gene Symbol	*p-value*	T score	Degrees Freedom
TOM1	0.0030	63.9181	-3.0669
FAM76B	0.0030	47.9664	3.0678
ADAM8	0.0030	65.3979	-3.0685
WDR44	0.0030	46.5586	3.0686
SPANXD	0.0030	59.4617	-3.0687
CHRNB3	0.0030	65.8574	-3.0705
GOLT1B	0.0030	45.0527	3.0705
STARD3NL	0.0030	49.5703	3.0707
ZMYND11	0.0030	47.8028	3.0731
VPS13C	0.0030	49.9254	3.0732
PI4K2B	0.0030	55.4134	3.0732
ALG5	0.0030	61.7541	3.0735
TSC22D1	0.0030	52.1975	3.0738
NKD2	0.0030	59.9481	-3.0758
RPL7	0.0030	41.0479	3.0759
C4orf52	0.0030	59.1188	3.0764
EFNA4	0.0030	65.9253	3.0769
MBNL1	0.0030	43.5681	3.0779
KRTAP9-3	0.0029	63.7739	-3.0785
ZNHIT3	0.0029	50.5687	3.0786
TMED2	0.0029	51.0836	3.0786
ZNF296	0.0029	65.6592	-3.0787
RAB5A	0.0029	62.0129	3.0790
CDC42SE2	0.0029	49.9107	3.0798
DPYD	0.0029	55.2289	3.0799
ZFP161	0.0029	48.5702	3.0802
CISD1	0.0029	65.7835	3.0813
HNRNPA1	0.0029	65.8119	3.0813
MRPS36	0.0029	65.4976	3.0817
SEC61B	0.0029	58.0005	3.0818
ZNF292	0.0029	57.2537	3.0826
CACNG7	0.0029	59.9998	-3.0831
GPR65	0.0029	59.7068	3.0832
HOXD10	0.0029	65.5550	-3.0832
MAST2	0.0029	63.3550	-3.0834
GFRA4	0.0029	64.2028	-3.0838
DENND4C	0.0029	47.6010	3.0845
CLN5	0.0029	60.7207	3.0864
C14orf166	0.0029	64.7020	3.0870
IRF2BP2	0.0029	57.8648	3.0889
ITK	0.0028	60.2594	3.0897
EIF2S2	0.0028	65.8954	3.0908
C10orf32	0.0028	55.6357	3.0913

Gene Symbol	*p-value*	T score	Degrees Freedom
SLC30A1	0.0028	46.2215	3.0920
ARL6IP1	0.0028	53.8041	3.0921
MRPL40	0.0028	58.4405	3.0925
MTHFD2	0.0028	48.5807	3.0928
YTHDC2	0.0028	64.2497	3.0935
CBR4	0.0028	64.6096	3.0940
FBXO36	0.0028	63.8656	-3.0948
SRP14	0.0028	57.2647	3.0956
SUZ12	0.0028	48.2443	3.0956
TNFAIP8	0.0028	51.3163	3.0958
IL15	0.0028	51.6852	3.0960
TRAF5	0.0028	63.5702	3.0972
GZMK	0.0028	55.7646	3.0992
PRSS48	0.0028	65.9962	-3.0995
C17orf95	0.0028	60.9116	3.0999
DCUN1D5	0.0028	50.8823	3.1004
UTP23	0.0028	65.3020	3.1010
CNOT6	0.0028	54.9313	3.1010
C1D	0.0027	58.4379	3.1012
PTP4A1	0.0027	54.3469	3.1016
RECQL4	0.0027	62.6118	-3.1029
PAPOLA	0.0027	52.6614	3.1032
FNDC3A	0.0027	48.7290	3.1032
CCL21	0.0027	63.2530	-3.1037
DEFB134	0.0027	62.9851	-3.1046
EIF2AK3	0.0027	52.2587	3.1048
TMEM9B	0.0027	61.5469	3.1053
TAS2R4	0.0027	65.7203	-3.1054
EPO	0.0027	65.4487	-3.1063
PCMT1	0.0027	59.4157	3.1089
SYT5	0.0027	65.9097	-3.1109
SMC3	0.0027	65.6071	3.1118
UBE2V2	0.0027	60.3077	3.1120
ETFA	0.0027	61.3304	3.1126
GOLPH3	0.0027	52.0867	3.1131
TEAD2	0.0026	63.3639	-3.1137
OLA1	0.0026	58.1473	3.1137
DYNLL2	0.0026	61.8425	3.1151
SERPINB9	0.0026	63.7508	3.1162
HDHD2	0.0026	57.6859	3.1168
SLC16A5	0.0026	59.2614	-3.1177
KIAA0391	0.0026	64.3507	3.1179
TUBD1	0.0026	62.6231	3.1179

Gene Symbol	*p-value*	T score	Degrees Freedom
PDYN	0.0026	65.9960	-3.1179
ERN2	0.0026	55.9114	-3.1194
RAD17	0.0026	57.6780	3.1195
NAA20	0.0026	60.6073	3.1196
DDC	0.0026	59.8821	-3.1211
VCPIP1	0.0026	44.9256	3.1219
PRRX2	0.0026	63.9020	-3.1232
ADAM20	0.0026	65.1983	-3.1233
ZFYVE21	0.0026	64.1582	3.1241
SRP9	0.0026	51.1598	3.1250
ANKRD36	0.0026	59.4114	3.1252
PAX4	0.0026	65.9182	-3.1255
WSB2	0.0025	54.2429	3.1259
OR2A1	0.0025	60.8471	-3.1259
UBE2Q2	0.0025	43.5892	3.1262
CCDC90B	0.0025	50.2783	3.1267
ADAMTS2	0.0025	65.9860	-3.1274
CCDC122	0.0025	65.9055	-3.1284
PHKB	0.0025	62.1737	3.1292
NADK	0.0025	65.7199	-3.1297
TMEM50B	0.0025	54.5185	3.1299
ARAP2	0.0025	42.0768	3.1308
OR6V1	0.0025	65.7159	-3.1309
RNF6	0.0025	43.4631	3.1312
COQ5	0.0025	63.1103	3.1319
SSFA2	0.0025	59.3506	3.1321
HEPACAM	0.0025	62.6230	-3.1327
PHAX	0.0025	60.3630	3.1327
VPS54	0.0025	59.0070	3.1331
ZNF511	0.0025	65.5689	3.1352
C9orf163	0.0025	65.7591	-3.1372
CSF3R	0.0025	65.5208	-3.1375
RCOR2	0.0024	65.4035	-3.1398
CEP192	0.0024	65.9876	3.1406
PAQR4	0.0024	64.5935	-3.1408
C14orf178	0.0024	62.4735	-3.1410
CRABP2	0.0024	65.7155	-3.1413
PTGER2	0.0024	58.8511	3.1413
LOC284889	0.0024	65.9684	-3.1449
EIF2S1	0.0024	65.1145	3.1450
SSX3	0.0024	50.1665	-3.1451
TNPO1	0.0024	55.8493	3.1461
KRTAP8-1	0.0024	65.1849	-3.1462

Gene Symbol	*p-value*	T score	Degrees Freedom
DNAJC12	0.0024	62.8752	-3.1476
AKAP11	0.0024	49.5965	3.1480
CYP4F2	0.0024	65.5344	-3.1487
YWHAG	0.0024	56.7705	3.1489
TMEM209	0.0024	61.9979	3.1502
UBE2N	0.0024	54.1970	3.1509
LOC647979	0.0024	51.9882	3.1513
VPS36	0.0024	46.8455	3.1524
ABCB7	0.0023	65.5718	3.1530
POT1	0.0023	55.5573	3.1534
HNRPLL	0.0023	46.9518	3.1535
SHCBP1	0.0023	60.0380	3.1539
SEPT7	0.0023	56.3818	3.1546
RPP30	0.0023	65.9524	3.1559
ITGA11	0.0023	61.6218	-3.1561
RARG	0.0023	64.9843	-3.1568
BAIAP2	0.0023	61.1990	-3.1569
SVIP	0.0023	52.1831	3.1580
DCAF13	0.0023	61.6533	3.1605
RBM16	0.0023	62.5897	3.1608
SLC37A1	0.0023	62.6039	3.1610
MYLK3	0.0023	64.5433	-3.1613
STT3B	0.0023	52.9887	3.1615
GCH1	0.0023	47.7919	3.1616
IL17C	0.0023	54.9474	-3.1622
ING1	0.0023	62.5304	3.1632
COX7B2	0.0023	59.1633	-3.1633
EIF2B1	0.0023	64.4217	3.1654
C9orf95	0.0023	62.8233	3.1658
SGTB	0.0023	49.6351	3.1658
OSBPL6	0.0022	65.4972	-3.1677
OR2T1	0.0022	65.1519	-3.1686
CRX	0.0022	65.6939	-3.1687
ZNF12	0.0022	55.6401	3.1693
WDR75	0.0022	60.1544	3.1698
ZRANB2	0.0022	53.0476	3.1699
C12orf75	0.0022	53.2600	3.1706
LOC100289585	0.0022	63.4281	-3.1710
GPSM1	0.0022	64.2134	-3.1711
ZCCHC7	0.0022	59.6990	3.1717
INMT	0.0022	63.0919	-3.1728
RECQL	0.0022	48.9812	3.1737
C1orf131	0.0022	65.9487	3.1739

Gene Symbol	*p-value*	T score	Degrees Freedom
LEFTY1	0.0022	56.1316	3.1740
SGPP2	0.0022	65.4170	-3.1745
SSB	0.0022	55.0839	3.1757
MRFAP1L1	0.0022	52.8163	3.1768
GDPD4	0.0022	63.6891	-3.1769
MTO1	0.0022	60.9572	3.1772
ABCE1	0.0022	48.0449	3.1774
SLC25A40	0.0022	60.3854	3.1774
OSTC	0.0022	49.0147	3.1776
MORC3	0.0022	47.5485	3.1784
ANKRD49	0.0022	52.3288	3.1792
MARCH1	0.0022	47.7164	3.1795
AKAP5	0.0022	58.2019	-3.1795
TXNDC9	0.0022	44.1140	3.1798
SYP	0.0022	64.0156	-3.1800
TTC22	0.0022	64.6189	-3.1807
HMP19	0.0022	64.8209	-3.1808
GABPB1	0.0022	52.1723	3.1815
CTDSPL2	0.0021	53.0621	3.1834
VAV1	0.0021	63.2791	-3.1842
KRCC1	0.0021	65.9138	3.1842
SLC25A36	0.0021	56.1962	3.1846
CSTF3	0.0021	63.5724	3.1846
LOC100287042	0.0021	65.6536	-3.1847
TNFRSF1A	0.0021	65.1614	-3.1877
SEC23A	0.0021	49.4960	3.1894
DUSP5	0.0021	62.9470	3.1896
DERA	0.0021	57.8522	3.1896
HEPHL1	0.0021	50.4101	-3.1904
C8A	0.0021	58.8802	-3.1905
CYP2A13	0.0021	64.7748	-3.1911
ARHGEF11	0.0021	61.9811	-3.1913
CHCHD7	0.0021	65.3599	3.1918
BTBD1	0.0021	55.6734	3.1918
UBE2G1	0.0021	60.1699	3.1920
ARAP3	0.0021	57.7948	-3.1925
SNTB1	0.0021	57.2472	3.1932
CCL22	0.0021	64.7308	-3.1940
PDE8A	0.0021	62.7025	3.1943
FAM166B	0.0021	61.4521	-3.1950
CAMTA1	0.0021	58.9069	3.1954
ANP32E	0.0021	48.0522	3.1962
C10orf18	0.0021	58.3532	3.1966

Gene Symbol	*p-value*	T score	Degrees Freedom
PPCS	0.0021	59.5748	3.1967
ANKRD32	0.0020	52.5861	3.1975
ZSCAN29	0.0020	61.4064	3.1989
APIP	0.0020	52.4335	3.1991
GNAI2	0.0020	56.6617	-3.1992
PSMC2	0.0020	63.9791	3.1995
TNKS2	0.0020	65.8140	3.1996
TXNL4A	0.0020	65.8634	3.1997
NUPL2	0.0020	59.1235	3.2002
NOLC1	0.0020	58.8450	3.2007
C6orf146	0.0020	62.1778	-3.2009
PCDHA6	0.0020	65.9430	-3.2010
NXF5	0.0020	56.0626	-3.2010
KRTAP10-3	0.0020	65.7826	-3.2012
AGK	0.0020	65.9611	3.2014
TTC35	0.0020	61.9627	3.2016
FAM91A1	0.0020	54.1080	3.2016
ERBB2IP	0.0020	46.9866	3.2027
G3BP2	0.0020	48.3254	3.2030
C3orf17	0.0020	60.9808	3.2037
CCDC50	0.0020	52.3905	3.2038
TMEM54	0.0020	65.5541	-3.2043
SLA	0.0020	65.2451	-3.2043
VAMP4	0.0020	51.8816	3.2048
SCARNA20	0.0020	65.9461	-3.2083
RAB1B	0.0020	63.2045	-3.2087
CCNG1	0.0020	42.7298	3.2092
MIS12	0.0020	59.5380	3.2094
KCNS1	0.0020	65.1268	-3.2095
GCOM1	0.0020	51.5116	3.2115
BZW1	0.0020	44.4275	3.2118
SLC35A1	0.0020	52.7199	3.2121
PRNP	0.0020	48.9772	3.2128
PPP1R8	0.0020	64.3989	3.2130
EMX1	0.0020	59.0255	-3.2132
NIPA2	0.0019	65.9867	3.2149
WWP2	0.0019	64.0051	-3.2151
LYSMD3	0.0019	47.7032	3.2153
DHRS2	0.0019	65.9165	-3.2176
C9orf43	0.0019	65.9624	-3.2176
ATAD2	0.0019	60.7030	3.2180
KIR2DL4	0.0019	65.7391	-3.2196
MED4	0.0019	57.9504	3.2204

Gene Symbol	*p-value*	T score	Degrees Freedom
COMMD2	0.0019	65.6397	3.2208
SRCIN1	0.0019	65.9389	-3.2217
RTP3	0.0019	65.9435	-3.2233
IPO7	0.0019	57.0884	3.2235
GALP	0.0019	52.9208	-3.2245
ETAA1	0.0019	62.9641	3.2248
C12orf53	0.0019	63.9066	-3.2250
RAPGEF6	0.0019	53.6719	3.2262
VPREB1	0.0019	64.1393	-3.2273
RBM45	0.0019	65.5512	3.2275
FGL1	0.0019	61.3109	-3.2275
MOBKL1A	0.0019	60.1205	3.2276
SERP1	0.0019	58.8474	3.2286
MYBL1	0.0019	45.9698	3.2291
ENSA	0.0019	64.5244	3.2296
C4orf33	0.0018	64.9094	3.2308
CLPS	0.0018	59.0287	-3.2328
AZI2	0.0018	47.7012	3.2330
GBP3	0.0018	57.5167	3.2332
NAA25	0.0018	63.5626	3.2356
FBLIM1	0.0018	65.8955	-3.2360
ATPIF1	0.0018	63.8421	3.2370
MTERFD3	0.0018	56.9399	3.2377
ACADSB	0.0018	55.6777	3.2383
ATP8B5P	0.0018	52.0496	3.2386
MYLPF	0.0018	60.3766	-3.2404
SLAMF9	0.0018	65.8541	-3.2416
DPEP1	0.0018	65.9503	-3.2421
KRTAP1-1	0.0018	55.1436	-3.2422
RSPH6A	0.0018	62.0032	-3.2425
HPS3	0.0018	57.1341	3.2433
OR56A4	0.0018	64.2326	-3.2445
ZNF189	0.0018	58.4015	3.2447
ARL8B	0.0018	55.9747	3.2478
ADO	0.0018	58.2097	3.2480
PPP2CA	0.0017	58.3673	3.2485
ARL5A	0.0017	47.7848	3.2519
ZBTB1	0.0017	49.0439	3.2520
HLA-DPB2	0.0017	65.9520	-3.2525
MRVI1	0.0017	65.8789	-3.2540
CCT8	0.0017	65.5443	3.2546
LSM10	0.0017	64.8994	3.2558
HDAC7	0.0017	65.7135	-3.2560

Gene Symbol	*p-value*	T score	Degrees Freedom
SNX27	0.0017	65.9401	-3.2563
SLC33A1	0.0017	58.1238	3.2568
ERH	0.0017	51.4944	3.2571
ZNF330	0.0017	57.7962	3.2583
TGS1	0.0017	64.5263	3.2585
BZW2	0.0017	63.1342	3.2599
FLOT2	0.0017	64.6030	-3.2600
KLHL9	0.0017	55.7420	3.2610
C11orf84	0.0017	65.6163	-3.2620
COPS2	0.0017	44.2753	3.2625
GIT1	0.0017	63.0335	-3.2628
TMEM206	0.0017	59.0710	3.2649
KIAA0196	0.0017	65.2206	3.2651
CRLS1	0.0017	50.9282	3.2664
C12orf32	0.0017	65.9714	3.2670
NOP58	0.0016	61.1209	3.2676
NUDT21	0.0016	58.1626	3.2678
RG9MTD1	0.0016	65.6325	3.2678
FAM122B	0.0016	65.4558	3.2681
SEPT2	0.0016	56.8836	3.2703
ELN	0.0016	65.8750	-3.2711
FAM110C	0.0016	63.5184	-3.2713
CASP8AP2	0.0016	46.0910	3.2720
AMDHD1	0.0016	62.7856	3.2728
PSMD12	0.0016	60.8542	3.2732
MLF2	0.0016	65.3968	-3.2732
SNRPA1	0.0016	63.1337	3.2737
TCF23	0.0016	63.7332	-3.2772
LOC100505603	0.0016	46.5479	3.2781
CDON	0.0016	64.4491	-3.2783
GNA13	0.0016	43.9107	3.2785
WDR11	0.0016	51.8753	3.2788
NCRNA00176	0.0016	65.4307	-3.2788
GABRR2	0.0016	65.0779	-3.2796
MRPS28	0.0016	49.9048	3.2800
PAIP1	0.0016	47.1123	3.2805
MINPP1	0.0016	53.8035	3.2808
FBXO45	0.0016	63.1908	3.2820
CCDC43	0.0016	53.2845	3.2824
SMEK2	0.0016	43.4761	3.2829
RGS21	0.0016	65.7475	-3.2835
KIAA1045	0.0016	64.7464	-3.2840
MST1R	0.0016	58.3653	-3.2842

Gene Symbol	*p-value*	T score	Degrees Freedom
LOC150568	0.0016	62.0657	-3.2846
MSH2	0.0016	53.5766	3.2849
SLC30A5	0.0016	48.9177	3.2852
PRKAB2	0.0016	58.6180	3.2856
FAM96A	0.0016	44.1044	3.2857
CDC37L1	0.0016	53.6239	3.2865
NPPB	0.0016	54.9541	-3.2870
HMGN1	0.0015	60.7394	3.2880
C2orf55	0.0015	42.0753	-3.2884
HAT1	0.0015	57.6373	3.2887
DCTD	0.0015	64.5954	3.2894
DYNC1I2	0.0015	65.9508	3.2913
C3orf38	0.0015	48.2006	3.2917
TAS1R1	0.0015	65.9860	-3.2922
MRPL36	0.0015	63.0866	3.2924
KIAA0427	0.0015	65.7430	-3.2934
BANP	0.0015	63.5445	3.2934
FAM126A	0.0015	47.0117	3.2939
PSMD14	0.0015	58.0834	3.2947
FKBP3	0.0015	54.2386	3.2948
FAM188B	0.0015	65.9771	-3.2955
ADAMTSL4	0.0015	64.4973	-3.2989
ERI1	0.0015	59.1285	3.2993
COG5	0.0015	62.7950	3.3033
EFHA1	0.0015	54.5253	3.3060
EIF3M	0.0015	64.6337	3.3061
C5orf47	0.0015	65.9620	-3.3070
ADD3	0.0015	56.1472	3.3079
TUBB4Q	0.0014	64.9116	-3.3096
DPH5	0.0014	62.1786	3.3097
SUB1	0.0014	45.4157	3.3118
FRMD8	0.0014	64.4813	-3.3166
CD34	0.0014	54.0351	-3.3168
FAM60A	0.0014	61.8379	3.3172
KIAA0195	0.0014	65.3824	-3.3182
TBCE	0.0014	65.9678	3.3186
HSPB11	0.0014	53.4964	3.3198
INTS5	0.0014	64.3578	-3.3199
PTPMT1	0.0014	64.8976	3.3206
CX3CL1	0.0014	65.8611	-3.3215
ATP5C1	0.0014	56.6813	3.3226
TERT	0.0014	62.6698	-3.3231
ARMS2	0.0014	63.9069	-3.3237

Gene Symbol	*p-value*	T score	Degrees Freedom
RCHY1	0.0014	50.9931	3.3246
ZNF25	0.0014	65.0811	3.3253
RAD50	0.0014	58.7254	3.3257
C14orf106	0.0014	52.0260	3.3262
SUMO2	0.0014	51.2008	3.3270
ANO6	0.0014	48.4288	3.3273
RRAS2	0.0014	41.5492	3.3280
LOC283999	0.0014	62.8175	-3.3280
C3orf10	0.0014	65.8885	3.3281
NIPSNAP3A	0.0014	49.7699	3.3283
FATE1	0.0014	63.0059	-3.3288
PTPLB	0.0014	48.6450	3.3290
MKKS	0.0014	52.2402	3.3295
KIF2A	0.0013	48.0473	3.3317
LRRIQ4	0.0013	65.2007	-3.3319
ZNF791	0.0013	60.4644	3.3320
PTP4A2	0.0013	59.1578	3.3321
EEF1A2	0.0013	53.4998	-3.3324
TAF9	0.0013	54.8371	3.3327
CMPK1	0.0013	45.6092	3.3330
SRSF1	0.0013	52.8875	3.3358
ZNF146	0.0013	49.7492	3.3359
RAVER2	0.0013	61.1868	-3.3372
RPS6KA1	0.0013	59.2629	-3.3374
TUBGCP4	0.0013	65.6834	3.3393
LEPREL1	0.0013	64.9463	-3.3409
HMG20A	0.0013	59.6234	3.3418
GTF3C6	0.0013	63.3766	3.3418
TNFSF8	0.0013	65.8035	3.3430
USP1	0.0013	52.1776	3.3435
GNPAT	0.0013	60.3179	3.3485
TEAD3	0.0013	65.2912	-3.3501
TMED5	0.0013	43.7737	3.3503
KIAA0284	0.0013	65.9421	-3.3506
GIMAP2	0.0013	47.5161	3.3512
VBP1	0.0013	49.2912	3.3516
HMHA1	0.0013	61.8930	-3.3527
EPT1	0.0013	65.6714	3.3528
LYPLAL1	0.0013	60.0930	3.3530
FBXO3	0.0013	55.2113	3.3545
C4orf34	0.0012	54.7403	3.3557
HS3ST2	0.0012	65.9940	-3.3557
PCDHAC2	0.0012	60.2079	-3.3571

Gene Symbol	*p-value*	T score	Degrees Freedom
UTRN	0.0012	51.4106	3.3590
ZNF541	0.0012	64.6420	-3.3606
KRTAP10-2	0.0012	65.6864	-3.3610
VPS26A	0.0012	61.7309	3.3611
DDX52	0.0012	64.3843	3.3635
C9orf21	0.0012	65.7083	3.3647
CYP4F2	0.0012	65.9773	-3.3648
DEK	0.0012	57.9251	3.3653
B9D2	0.0012	65.2326	-3.3658
MED23	0.0012	54.9994	3.3720
MCTS1	0.0012	65.9848	3.3722
TM9SF3	0.0012	46.6771	3.3723
PHTF2	0.0012	45.3003	3.3727
DNAJC10	0.0012	57.5758	3.3732
TIMP2	0.0012	64.9842	-3.3736
UPK2	0.0012	57.1109	-3.3741
SRSF2IP	0.0012	54.1231	3.3762
SKIV2L2	0.0012	64.1939	3.3779
ECHDC1	0.0011	46.7619	3.3801
KRTAP10-10	0.0011	65.6739	-3.3804
ZBED5	0.0011	51.2208	3.3810
LMX1A	0.0011	65.2393	-3.3819
GOLGA4	0.0011	59.6160	3.3829
ROPN1	0.0011	65.3695	-3.3840
RHOXF2	0.0011	59.4611	-3.3857
SRSF11	0.0011	55.9411	3.3858
EPC2	0.0011	64.7665	3.3883
SUCLA2	0.0011	50.7248	3.3889
MED21	0.0011	50.6613	3.3898
DEGS2	0.0011	63.2969	-3.3902
VEZT	0.0011	54.7803	3.3915
OBSL1	0.0011	63.4397	-3.3917
MRPS35	0.0011	52.9168	3.3941
GRM4	0.0011	65.2342	-3.3965
GCLM	0.0011	56.3175	3.4005
SH2D1A	0.0011	46.8796	3.4011
PRPS2	0.0011	53.7755	3.4017
SUPT7L	0.0011	61.7943	3.4023
TOM1L2	0.0011	56.3261	-3.4024
APOBEC3G	0.0011	64.3528	3.4033
ARAP1	0.0011	64.4676	-3.4036
SLC38A9	0.0011	53.8095	3.4039
LOC100287428	0.0011	60.3954	-3.4054

Gene Symbol	*p-value*	T score	Degrees Freedom
SNRNP48	0.0011	59.9711	3.4061
HAUS1	0.0011	58.4624	3.4072
FGF21	0.0010	65.2188	-3.4120
PTGER4	0.0010	47.8117	3.4138
GNPNAT1	0.0010	62.8543	3.4147
ISLR2	0.0010	58.7567	-3.4169
ASF1A	0.0010	52.6153	3.4170
METTL5	0.0010	56.7530	3.4171
YWHAQ	0.0010	47.5172	3.4179
ZC3H15	0.0010	58.7563	3.4179
ELK3	0.0010	42.3575	3.4205
NARS	0.0010	61.5210	3.4208
KLF10	0.0010	46.0093	3.4221
C2orf3	0.0010	64.6112	3.4225
CHUK	0.0010	58.5277	3.4235
ENOPH1	0.0010	58.1521	3.4240
XPOT	0.0010	63.1102	3.4254
COX7A1	0.0010	64.2347	-3.4262
C11orf61	0.0010	58.0573	3.4303
CAPN12	0.0010	63.8620	3.4303
AGGF1	0.0010	50.2258	3.4313
SIRT7	0.0010	65.1388	-3.4322
NOL8	0.0010	65.1815	3.4327
ABCB10	0.0010	42.4386	3.4332
MGAT1	0.0010	65.9846	-3.4339
TLK1	0.0010	42.8228	3.4342
HTR3B	0.0010	60.1646	-3.4369
RAB11FIP2	0.0010	63.9869	3.4384
CRYZL1	0.0010	65.7268	3.4385
MAT2A	0.0010	55.5631	3.4391
BTAF1	0.0010	44.5792	3.4394
SAAL1	0.0009	65.5401	3.4416
CEP57	0.0009	53.1280	3.4429
RAP1B	0.0009	41.8222	3.4431
NRAS	0.0009	48.3077	3.4447
ORC4	0.0009	54.3903	3.4487
SMARCAD1	0.0009	49.8968	3.4498
THEMIS	0.0009	46.3350	3.4513
BARX1	0.0009	65.2153	-3.4515
OXR1	0.0009	46.3427	3.4540
KTN1	0.0009	57.4686	3.4549
ACAT1	0.0009	63.1725	3.4553
NAA15	0.0009	44.5580	3.4582

Gene Symbol	*p-value*	T score	Degrees Freedom
PPIP5K2	0.0009	47.6210	3.4618
C14orf129	0.0009	43.3857	3.4621
GRIK5	0.0009	64.7430	-3.4624
CEP170	0.0009	65.6304	3.4629
ANKRD40	0.0009	65.5654	3.4636
CPNE6	0.0009	61.5056	-3.4644
C19orf2	0.0009	53.6714	3.4648
HCK	0.0009	65.9978	-3.4661
MAST3	0.0009	65.5963	-3.4675
MNAT1	0.0009	66.0000	3.4691
CRY1	0.0009	55.4599	3.4697
KRT80	0.0009	65.7022	-3.4705
GPR183	0.0009	60.6432	3.4710
FSCN1	0.0009	65.4379	-3.4744
EIF3J	0.0009	54.6387	3.4748
MFAP4	0.0009	65.8040	-3.4751
MCTS1	0.0008	60.9462	3.4756
TXNDC15	0.0008	55.4465	3.4760
DENND1B	0.0008	62.6006	3.4770
WDR41	0.0008	59.0486	3.4797
STYX	0.0008	51.6263	3.4811
KPNA7	0.0008	62.2822	-3.4818
MALT1	0.0008	55.4311	3.4819
BTBD2	0.0008	64.5607	-3.4836
PPIL1	0.0008	56.5101	3.4841
ZNF226	0.0008	59.6484	3.4843
FAM98B	0.0008	60.5666	3.4850
TMEM156	0.0008	63.2056	3.4859
AASDHPPT	0.0008	55.0580	3.4875
RSPO1	0.0008	65.4173	-3.4880
ZNF700	0.0008	62.4048	3.4880
RHOU	0.0008	47.9999	3.4905
CYCS	0.0008	42.4668	3.4960
C1GALT1C1	0.0008	51.1765	3.4961
LEMD1	0.0008	65.0917	-3.4964
NME1-NME2	0.0008	63.5225	3.4997
NRIP1	0.0008	53.9206	3.5000
CCDC103	0.0008	63.8335	-3.5022
PPP2R4	0.0008	65.9991	-3.5027
TSEN15	0.0008	54.8873	3.5034
NCBP2	0.0008	54.6102	3.5041
MADCAM1	0.0008	65.9979	-3.5041
PNMA1	0.0008	63.5100	3.5049

Gene Symbol	*p-value*	T score	Degrees Freedom
SEC63	0.0008	52.4116	3.5070
C18orf55	0.0008	61.0127	3.5072
C15orf27	0.0008	65.0344	-3.5093
DCK	0.0008	48.9604	3.5104
MTRR	0.0008	54.7373	3.5123
UPRT	0.0007	63.0074	3.5139
AGER	0.0007	61.3630	-3.5159
ZNF644	0.0007	52.8122	3.5163
MRS2	0.0007	57.9992	3.5273
KCND1	0.0007	65.9185	-3.5284
PIK3C2A	0.0007	51.0487	3.5285
RAI14	0.0007	60.0045	-3.5310
CD226	0.0007	57.8950	3.5331
MXD3	0.0007	57.2897	-3.5341
SMAD5	0.0007	48.5104	3.5342
UBA2	0.0007	51.2807	3.5350
PPP2R5E	0.0007	65.5937	3.5353
HNRPDL	0.0007	63.6967	3.5371
C12orf76	0.0007	54.6377	3.5383
DLD	0.0007	57.2419	3.5393
CEP68	0.0007	65.0743	3.5399
C9orf80	0.0007	65.0013	3.5406
BCAP29	0.0007	51.8325	3.5411
DARS	0.0007	62.8275	3.5426
MTF2	0.0007	63.4742	3.5426
USP16	0.0007	65.2360	3.5442
MRPL30	0.0007	65.4945	3.5456
MT1G	0.0007	45.9137	-3.5467
MGC45922	0.0007	65.6492	-3.5513
RFK	0.0007	52.9341	3.5515
ACVR1	0.0007	55.1226	3.5524
ATAD2B	0.0007	57.0588	3.5533
CXCR1	0.0007	63.4859	-3.5540
PPA2	0.0006	57.3874	3.5558
HIBCH	0.0006	61.7133	3.5574
PCOTH	0.0006	64.4365	-3.5585
INSIG1	0.0006	52.0343	3.5588
PRAM1	0.0006	65.9998	-3.5626
NAP1L1	0.0006	57.9701	3.5626
PCGF2	0.0006	65.9997	-3.5660
MSI1	0.0006	65.1040	-3.5663
RBM3	0.0006	60.6707	3.5669
HTR1B	0.0006	65.5054	-3.5687

Gene Symbol	*p-value*	T score	Degrees Freedom
ACADM	0.0006	44.7641	3.5732
PRDM16	0.0006	65.4760	-3.5744
C5orf43	0.0006	61.3464	3.5759
KRTAP13-4	0.0006	65.2751	-3.5788
ATP13A3	0.0006	50.7627	3.5800
ITGA4	0.0006	50.0518	3.5809
ITGA5	0.0006	65.9746	-3.5821
C10orf99	0.0006	64.5353	-3.5834
LOC729815	0.0006	65.1289	-3.5839
USP47	0.0006	65.9373	3.5843
IBTK	0.0006	60.1201	3.5862
DDN	0.0006	64.3621	-3.5903
C3orf63	0.0006	46.1740	3.5929
KCNJ4	0.0006	64.3509	-3.5930
HSD3B7	0.0005	65.2018	-3.6059
ZNF187	0.0005	58.3463	3.6070
SNHG11	0.0005	65.4978	3.6078
RAX	0.0005	65.9241	-3.6080
ICOS	0.0005	56.5710	3.6155
C1orf103	0.0005	56.1491	3.6166
N4BP2L1	0.0005	47.6759	3.6225
LRRC8C	0.0005	64.3152	3.6260
KLF11	0.0005	58.4913	3.6323
MRPS10	0.0005	65.0949	3.6349
SAR1A	0.0005	62.5506	3.6362
SLC45A1	0.0005	64.5408	-3.6372
CAPRIN1	0.0005	56.9115	3.6389
ATF2	0.0005	42.9218	3.6391
PADI1	0.0005	64.9225	-3.6406
ATG4C	0.0005	61.9096	3.6471
JAK3	0.0005	62.1367	-3.6523
TRIM46	0.0005	64.3493	-3.6607
FGF11	0.0004	65.5910	-3.6643
CSK	0.0004	64.2557	-3.6649
CLIP4	0.0004	49.4582	3.6672
OR10G7	0.0004	65.9958	-3.6677
TGDS	0.0004	55.6959	3.6715
ARFGEF2	0.0004	41.4744	3.6731
RPL21	0.0004	61.2487	3.6731
DONSON	0.0004	62.8177	3.6861
PRPF4B	0.0004	48.9697	3.6868
CEBPZ	0.0004	55.4180	3.6872
PKDREJ	0.0004	65.2382	-3.6881

Gene Symbol	*p-value*	T score	Degrees Freedom
AGPAT5	0.0004	45.1180	3.6920
DNAJC21	0.0004	56.1310	3.6925
ATP2C1	0.0004	55.4903	3.6931
ITGB1	0.0004	48.1117	3.7015
LOC199899	0.0004	65.9969	-3.7055
PNPT1	0.0004	50.6733	3.7080
PLRG1	0.0004	60.5286	3.7104
GLO1	0.0004	53.3164	3.7160
PDSS1	0.0004	57.1488	3.7187
TIMM17A	0.0004	61.5019	3.7195
XRN1	0.0004	47.2792	3.7246
SMC4	0.0004	57.6188	3.7264
KRT35	0.0004	65.8371	-3.7265
DBF4	0.0004	50.2776	3.7271
FMNL1	0.0004	65.4912	-3.7348
PRKRIR	0.0003	46.1902	3.7375
UBA5	0.0003	56.9895	3.7455
CLIP3	0.0003	64.8660	-3.7456
MRPL19	0.0003	59.7733	3.7462
C14orf147	0.0003	49.3624	3.7504
HIST1H2AH	0.0003	63.1851	-3.7507
NUS1	0.0003	41.3165	3.7573
NLK	0.0003	62.3548	3.7590
PHACTR2	0.0003	65.6228	3.7626
GPN3	0.0003	55.1466	3.7674
KCTD19	0.0003	63.4805	-3.7775
GPRIN1	0.0003	63.7393	-3.7794
ALG6	0.0003	65.2649	3.7875
LARP7	0.0003	56.3229	3.7921
SRSF10	0.0003	55.5858	3.7978
UBAP2L	0.0003	65.9940	-3.7981
COX11	0.0003	45.1650	3.8077
OR9G4	0.0003	65.8433	-3.8244
KANK3	0.0002	65.8527	-3.8377
AQP12A	0.0002	65.9831	-3.8420
ATP11C	0.0002	48.4431	3.8426
GPR123	0.0002	62.3933	-3.8445
C2orf70	0.0002	63.1841	-3.8486
NEU4	0.0002	65.2248	-3.8506
ZNF75A	0.0002	63.4110	3.8516
MRPL50	0.0002	60.7344	3.8531
TSN	0.0002	59.3652	3.8562
CDRT4	0.0002	65.3391	3.8567

Gene Symbol	*p-value*	T score	Degrees Freedom
FAM57B	0.0002	56.4707	-3.8609
TRMT61B	0.0002	57.5298	3.8617
C18orf32	0.0002	44.9176	3.8619
CCDC88A	0.0002	50.0466	3.8667
TPP2	0.0002	63.6548	3.8714
TWISTNB	0.0002	57.7037	3.8803
IER3IP1	0.0002	53.7878	3.8825
SCT	0.0002	63.7660	-3.8826
SYN1	0.0002	60.7104	-3.8939
SLMO2	0.0002	50.1358	3.9128
MEF2C	0.0002	54.7562	3.9141
CTCFL	0.0002	65.2917	3.9203
NCBP1	0.0002	64.0481	3.9219
SRSF3	0.0002	57.5499	3.9228
C12orf11	0.0002	55.3197	3.9241
C15orf61	0.0002	65.9836	3.9330
C7orf36	0.0002	65.1141	3.9361
ZNF639	0.0001	61.5162	3.9720
EBAG9	0.0001	59.3970	3.9811
CPSF2	0.0001	65.8669	3.9816
C21orf7	0.0001	47.6053	3.9842
HRH2	0.0001	64.2872	-3.9958
KCNJ11	0.0001	63.3879	-4.0000
IL1F7	0.0001	63.6998	-4.0088
PRLHR	0.0001	61.3599	-4.0211
FTSJD1	0.0001	41.8730	4.0353
SLAIN1	0.0001	49.8644	4.0380
NUP37	0.0001	63.9362	4.0404
ARMC8	0.0001	59.7008	4.0439
MUDENG	0.0001	44.9598	4.0640
TYW3	0.0001	64.8236	4.0714
PYROXD1	0.0001	53.0787	4.0775
TMEM150B	0.0001	64.5907	-4.0775
HMGB1	0.0001	48.9584	4.0930

Gene Symbol	*p-value*	T score	Degrees Freedom
MAGEE2	0.0001	59.7063	-4.1132
SCFD1	0.0001	60.4648	4.1137
ARMC1	0.0001	53.6358	4.1236
GNB2	0.0001	65.5920	-4.1308
KRR1	0.0001	56.7425	4.1405
SNRPE	0.0001	61.7317	4.1449
ENTPD2	0.0001	64.5691	-4.2028
ZFAND6	0.0001	54.5143	4.2143
C6orf120	0.0001	46.1868	4.2308
DNM1L	0.0001	57.1690	4.2465
ZNF540	0.0000	58.2169	4.2627
SS18	0.0000	49.0729	4.2930
FABP5	0.0000	60.9945	4.2938
PLCH2	0.0000	61.0007	-4.2984
HGS	0.0000	65.8498	-4.3157
GPD2	0.0000	65.1618	4.3178
ARL6IP5	0.0000	60.9455	4.3474
KIRREL	0.0000	65.4452	-4.3555
ATP5S	0.0000	64.0755	4.3766
FASTKD1	0.0000	55.7748	4.3937
SNORA32	0.0000	55.7749	4.4591
KCNK7	0.0000	65.9468	-4.4928
CCNH	0.0000	65.7302	4.5326
RNF128	0.0000	64.7575	-4.5720
BIRC7	0.0000	62.4460	-4.6481
GLMN	0.0000	64.9082	4.7414
COQ3	0.0000	63.8692	4.8036
LOC91316	0.0000	48.7706	-5.1154
