# Supplementary material for: Gene Expression Differences in Peripheral Blood of Parkinson’s Disease Patients with Distinct Progression Profiles
Source: PLoS One. 2016 Jun 20;11(6):e0157852. doi: 10.1371/journal.pone.0157852 (PMC4913914; doi:10.1371/journal.pone.0157852)
Supplement: S6 Table — (PDF) [file pone.0157852.s011.pdf]

S6 Table. Genes detected as differentially expressed between slow and rapid progression PD patients identified by T-test with permutations.

Gene Symbol	*p-value*	T score	Degrees Freedom
TOP1	61,2270	3,4396	0,0011
RNF165	59,0486	3,4409	0,0011
C11orf42	65,8680	3,2787	0,0017
RBKS	65,0934	-3,1487	0,0025
ASB18	50,2673	3,0450	0,0037
WDR72	60,9019	-2,9507	0,0045
TPD52L1	65,9835	-2,9103	0,0049
C1orf57	63,9309	-2,8794	0,0054
FAM5C	65,9968	-2,8606	0,0057
PLA2G12B	65,6354	-2,8338	0,0061
RELL2	62,5825	-2,8341	0,0062
ADIPOR2	55,5687	-2,8429	0,0062
COX6A1	63,0467	-2,8233	0,0064
HSP90AB6P	64,8788	-2,7994	0,0067
ZFP41	65,4827	-2,7972	0,0068
RAD18	63,3168	2,7883	0,0070
ZNF284	65,8577	2,7831	0,0070
RSPH10B2	55,7316	-2,7819	0,0074
OR2G3	63,6039	-2,7521	0,0077
PCDHGB7	65,7603	2,7276	0,0082
LOC729815	64,2220	-2,7296	0,0082
CBWD1	55,5128	2,7296	0,0085
ZNF221	62,4366	2,6818	0,0094
SPNS2	65,9605	-2,6741	0,0094
HEATR1	53,8731	-2,6713	0,0100
TRIM65	60,7050	-2,6570	0,0101
PLAGL2	65,7821	-2,6501	0,0101
RFESD	53,9763	-2,6531	0,0105
TBX2	59,4826	2,6316	0,0108
POLR2C	63,0221	2,6254	0,0108
DCTN2	62,5472	-2,5969	0,0117
C22orf34	64,0710	-2,5910	0,0118
HIST1H2BJ	53,8469	2,5910	0,0123
ZNF383	60,1479	-2,5720	0,0126
FDX1L	65,7994	-2,5360	0,0136
MRM1	64,8558	-2,5245	0,0140
ATP4A	61,3776	-2,5247	0,0142
ENKUR	61,7512	2,5141	0,0146

Gene Symbol	*p-value*	T score	Degrees Freedom
LHX5	62,6382	2,5128	0,0146
FTCD	58,4678	2,5160	0,0146
OR2L13	57,2665	2,5074	0,0150
CCDC144C	60,8114	2,4986	0,0152
MND1	54,0286	2,4916	0,0158
LOC100287301	65,4172	-2,4743	0,0160
ANUBL1	65,5053	-2,4381	0,0175
YY2	65,5499	2,4321	0,0178
ABCC11	55,5234	-2,4349	0,0181
C16orf79	64,5141	-2,4244	0,0181
HP1BP3	65,9537	-2,4227	0,0182
TEKT1	51,0770	2,4357	0,0184
UBL4B	65,9266	-2,4083	0,0188
SMPD2	65,9223	-2,3989	0,0193
CILP	65,9932	2,3964	0,0194
BHLHE23	59,1935	-2,4026	0,0194
CPT1A	63,0919	-2,3886	0,0199
SCG5	58,3503	2,3870	0,0202
TMEM132C	60,0191	2,3781	0,0206
MED31	65,9999	-2,3720	0,0206
ROM1	65,9886	2,3718	0,0206
MRPS15	62,6466	-2,3732	0,0207
GTF2A1	64,8052	-2,3666	0,0209
IGFALS	65,5337	-2,3630	0,0211
AVPR1B	59,2307	2,3676	0,0212
SYCE2	51,7182	2,3689	0,0216
S100P	47,6773	-2,3637	0,0222
CC2D2B	55,8382	-2,3511	0,0223
PPAPDC3	58,8119	-2,3466	0,0223
SCLY	60,0739	-2,3351	0,0229
HOMER2	56,5108	2,3355	0,0231
GRAPL	60,4457	-2,3287	0,0232
HIST2H4A	63,1094	2,3208	0,0235
SENP2	64,9704	2,3087	0,0242
NMUR1	65,7426	2,3043	0,0244
SLC35D1	65,0559	-2,3034	0,0245
WASH3P	65,9794	-2,3018	0,0245
USP31	54,3313	-2,3122	0,0246

Gene Symbol	*p-value*	T score	Degrees Freedom
C15orf48	65,7088	-2,3007	0,0246
DOK7	65,6643	-2,2984	0,0247
C1QTNF6	65,9243	-2,2969	0,0248
TXNDC17	65,8547	-2,2942	0,0250
OLFM2	62,2706	-2,2962	0,0250
ZNF334	57,5153	2,2994	0,0251
BCO2	61,5984	2,2901	0,0255
FAM125A	64,4071	-2,2841	0,0257
GALK1	65,1379	-2,2802	0,0259
DCBLD1	55,2065	-2,2867	0,0261
HGSNAT	63,3368	2,2775	0,0261
NMNAT3	65,9733	-2,2714	0,0264
ELMO1	65,0687	2,2717	0,0264
RSPH6A	65,7898	-2,2708	0,0264
PVALB	56,9925	2,2786	0,0265
SNRNP25	55,6348	-2,2758	0,0267
GLB1L	63,2591	-2,2664	0,0269
C20orf152	65,5520	2,2617	0,0270
UBIAD1	64,2397	-2,2553	0,0275
F8	60,3542	2,2535	0,0279
C1orf69	64,8596	-2,2480	0,0280
HIST1H2BC	65,9465	2,2458	0,0281
RDH16	57,8907	-2,2510	0,0282
HMHB1	64,8833	2,2447	0,0282
ACTA2	63,6464	2,2442	0,0283
FAM194B	62,7096	2,2445	0,0283
RSPH9	56,9343	2,2461	0,0286
OR5M1	59,2983	-2,2424	0,0287
VMO1	60,3653	-2,2381	0,0289
IPO13	65,5418	-2,2311	0,0291
EPHX4	63,2092	2,2308	0,0292
LOC100131234	64,4239	2,2297	0,0293
RGS3	65,3163	2,2252	0,0295
ACTRT2	65,9187	-2,2113	0,0305
OR10AD1	65,8074	2,2071	0,0308
RLBP1	56,9982	-2,2047	0,0315
ZNF673	58,7474	2,2027	0,0316
MAS1L	52,5722	2,2090	0,0316
MIPEP	63,8787	-2,1900	0,0322
CENPE	65,8502	-2,1861	0,0324
LCNL1	55,5553	2,1936	0,0325
ATAD3A	60,7199	-2,1888	0,0325
CDC42BPG	63,4623	-2,1756	0,0333

Gene Symbol	*p-value*	T score	Degrees Freedom
SIGLEC11	64,4072	-2,1698	0,0337
CPA6	65,1281	2,1674	0,0339
HES4	65,6005	-2,1653	0,0340
INSC	51,0578	2,1770	0,0341
CHTF18	65,8707	-2,1567	0,0347
PSTPIP2	55,8054	2,1632	0,0348
GUCA2A	65,9959	2,1533	0,0350
RRN3	61,7803	-2,1490	0,0356
CCR1	61,6880	-2,1480	0,0356
CCDC7	49,9950	2,1591	0,0357
SLC16A14	61,8622	2,1447	0,0359
CD24	66,0000	-2,1388	0,0362
AQP7	65,7881	-2,1375	0,0363
KRT16P1	53,3805	2,1453	0,0365
RNASEH2C	59,7589	-2,1386	0,0366
TRIM31	65,8232	-2,1334	0,0366
COL23A1	65,2066	2,1334	0,0367
NUDT7	60,2859	2,1326	0,0370
ZNF404	64,4336	-2,1271	0,0372
C18orf56	62,5188	-2,1257	0,0375
PFKM	65,9165	-2,1233	0,0375
ANKRD20A1	63,5924	2,1245	0,0375
ABCC1	62,1749	-2,1253	0,0375
ABCC6	54,7024	2,1278	0,0379
KIAA1671	63,7586	-2,1189	0,0380
COL2A1	53,3692	-2,1227	0,0384
PIK3R5	64,0589	2,1138	0,0384
CALY	63,8191	2,1135	0,0385
C20orf144	51,7667	2,1221	0,0386
CCBP2	64,0118	-2,1099	0,0388
PTOV1	61,5686	-2,1097	0,0390
OR1F1	56,0647	2,1141	0,0390
TTC7B	65,8089	2,1061	0,0390
TRIM49	63,8621	2,1064	0,0391
ZNF256	64,9687	2,1051	0,0392
LOC100287497	61,6122	-2,1020	0,0396
ST3GAL1	65,6427	-2,0989	0,0397
DEFB116	63,4471	2,0998	0,0397
VSTM1	60,2405	-2,1003	0,0399
FANCI	62,3506	-2,0983	0,0399
MRPS5	65,0633	-2,0954	0,0400
HIST3H3	59,9418	2,0970	0,0402
GRHL3	57,1749	2,0981	0,0403

Gene Symbol	*p-value*	T score	Degrees Freedom
LOC645954	65,3209	2,0790	0,0415
PSPH	63,0480	-2,0805	0,0416
C12orf44	55,0616	-2,0846	0,0418
NDUFA8	64,6004	-2,0757	0,0419
VPS35	61,7644	-2,0713	0,0425
HEATR2	65,6147	-2,0659	0,0428
C5orf45	65,3551	-2,0656	0,0428
TCEAL3	65,7728	-2,0652	0,0428
RPH3A	63,3115	2,0656	0,0430
GPT2	64,4005	-2,0642	0,0430
CROCC	65,5650	-2,0632	0,0431
CNTNAP1	60,6133	-2,0652	0,0432
SCARF1	65,9228	2,0598	0,0434
PNMA1	65,9519	2,0568	0,0437
CALM3	65,2159	2,0569	0,0437
PDZD7	57,0182	-2,0617	0,0438
CCDC99	63,0810	-2,0525	0,0443
SNORA33	65,9988	-2,0504	0,0443
WDR53	62,6156	2,0505	0,0445
DNMT3A	65,9450	-2,0470	0,0446
SHPK	65,9515	-2,0470	0,0447
OR8B4	54,1329	2,0524	0,0450
STAB1	58,6819	-2,0482	0,0450
BMP2K	65,9954	-2,0417	0,0452
FAM155B	65,4670	-2,0403	0,0454
TSC2	65,9260	-2,0386	0,0455
ATP11A	64,4344	2,0382	0,0456
SSTR2	61,4255	2,0385	0,0458
AKR1CL1	65,8869	-2,0339	0,0460
MFSD6L	52,1824	-2,0417	0,0463

Gene Symbol	*p-value*	T score	Degrees Freedom
IFNA7	65,4780	2,0294	0,0465
DUOX1	48,2871	2,0409	0,0467
LOC100287314	62,3937	2,0271	0,0469
RESP18	55,3021	2,0303	0,0471
SYCP2L	65,8209	-2,0217	0,0473
CDK9	65,9558	-2,0193	0,0475
OR6C74	63,6301	2,0201	0,0476
GSR	65,9999	2,0152	0,0480
CCDC134	54,9172	2,0228	0,0480
PIWIL3	62,9610	2,0168	0,0480
HIST1H4H	53,5535	2,0217	0,0482
INO80D	65,9882	2,0127	0,0482
GOLGA8C	65,9420	2,0115	0,0484
HLA-H	65,8970	2,0100	0,0485
VAMP2	65,8463	-2,0095	0,0486
WISP2	65,9832	-2,0092	0,0486
ZBTB12	60,2837	-2,0108	0,0488
ABL2	65,9986	-2,0039	0,0492
FLJ44881	59,4884	2,0072	0,0493
LOC100131642	65,9020	2,0027	0,0493
TMEM110	61,1851	2,0043	0,0495
LTF	64,9529	-2,0019	0,0495
MEIG1	52,8924	2,0105	0,0495
TXNDC2	53,8957	-2,0079	0,0497
LEUTX	61,6579	-2,0015	0,0497
DEFB125	65,6011	-1,9990	0,0498
RNF20	61,5760	-2,0013	0,0498
LYPD6	56,4265	2,0038	0,0499
PCDHGB3	60,6312	-2,0005	0,0499
