# Supplementary material for: Gene Expression Differences in Peripheral Blood of Parkinson’s Disease Patients with Distinct Progression Profiles
Source: PLoS One. 2016 Jun 20;11(6):e0157852. doi: 10.1371/journal.pone.0157852 (PMC4913914; doi:10.1371/journal.pone.0157852)
Supplement: S7 Table — (PDF) [file pone.0157852.s012.pdf]

S7 Table: Genes changed in PD patients with rapid vs slow progression, and in MPTP/SNCA data sets microarray meta analysis.

Gene Symbol	Gene Title
ARFGEF2	ADP-ribosylation factor guanine nucleotide-exchange factor 2 (brefeldin A-inhibited)
ARMC1	armadillo repeat containing 1
ATP13A3	ATPase type 13A3
CPSF2	cleavage and polyadenylation specific factor 2, 100kDa
CYCS	cytochrome c, somatic
DBF4	DBF4 homolog (S. cerevisiae)
DCK	deoxycytidine kinase
DONSON	downstream neighbor of SON
FABP5	fatty acid binding protein 5 (psoriasis-associated)
FAM57B	family with sequence similarity 57, member B
FAM98B	family with sequence similarity 98, member B
GLO1	glyoxalase I
GPR123	G protein-coupled receptor 123
HMGB1	high-mobility group box 1
HSD3B7	hydroxy-delta-5-steroid dehydrogenase, 3 beta- and steroid delta-isomerase 7
ICOS	inducible T-cell co-stimulator
ITGA4	integrin, alpha 4 (antigen CD49D, alpha 4 subunit of VLA-4 receptor)
ITGB1	integrin, beta 1 (fibronectin receptor, beta polypeptide, antigen CD29 includes MDF2, MSK12)
KANK3	KN motif and ankyrin repeat domains 3
KCND1	potassium voltage-gated channel, Shal-related subfamily, member 1
LRRC8C	leucine rich repeat containing 8 family, member C
MRPL19	mitochondrial ribosomal protein L19
MRPL30	mitochondrial ribosomal protein L30
MRPS10	mitochondrial ribosomal protein S10

Gene Symbol	Gene Title
MSI1	musashi homolog 1 (Drosophila)
MXD3	MAX dimerization protein 3
N4BP2L1	NEDD4 binding protein 2-like 1
NAP1L1	nucleosome assembly protein 1-like 1
NCBP2	nuclear cap binding protein subunit 2, 20kDa
NLK	nemo-like kinase
PADI1	peptidyl arginine deiminase, type I
PHACTR2	phosphatase and actin regulator 2
PLRG1	pleiotropic regulator 1 (PRL1 homolog, Arabidopsis)
RAI14	retinoic acid induced 14
RBM3	RNA binding motif (RNP1, RRM) protein 3
RFK	riboflavin kinase
SAR1A	SAR1 homolog A (S. cerevisiae)
TPP2	tripeptidyl peptidase II
TRIM46	tripartite motif-containing 46
TYW3	tRNA-yW synthesizing protein 3 homolog (S. cerevisiae)
WDR41	WD repeat domain 41
XRN1	5'-3' exoribonuclease 1
