# Supplementary material for: Gene Expression Differences in Peripheral Blood of Parkinson’s Disease Patients with Distinct Progression Profiles
Source: PLoS One. 2016 Jun 20;11(6):e0157852. doi: 10.1371/journal.pone.0157852 (PMC4913914; doi:10.1371/journal.pone.0157852)
Supplement: S8 Table — Analysis was conducted through DAVID[55]. (PDF) [file pone.0157852.s013.pdf]

S8 Table. GO analysis, conducted through the DAVID, on genes selected for qPCR validation

Gene Symbol	Affy ID	Gene Name	Biological Function	Cellular Localization	Molecular Function
ABCA1	11737559 _a_at	ATP-Binding Cassette, Sub-Family A (ABC1), Member	Transport, small GTPase mediated signal transduction, protein transport, regulation of ARF GTPase activity	Plasma membrane, adherens junction, membrane, integral to membrane, basolateral plasma membrane, apical plasma membrane, cell junction	Nucleotide binding, protein kinase activity, protein serine/threonine kinase activity, protein binding, ATP binding, kinase activity, transferase activity
RAD18	11728257 _at	Postreplication Repair Protein RAD18	DNA repair, response to DNA damage stimulus, spermatogenesis, response to UV, negative regulation of DNA recombination	Chromatin, XY body, nucleus,	Y-form DNA binding, nucleic acid binding, DNA binding, damaged DNA binding, protein binding, zinc ion binding, ligase activity, ubiquitin protein ligase binding, metal ion binding
FOXP1	11732279 _at	Forkhead Box P1	Negative regulation of transcription from RNA polymerase II promoter, positive regulation of mesenchymal cell proliferation, pre-B cell differentiation, positive regulation of immunoglobulin production, transcription, regulation of transcription, DNA-dependent, pattern specification process, skeletal muscle tissue development, embryo development, regulation of gene-specific transcription from RNA polymerase II promoter, lung development, negative regulation of gene-specific transcription, immunoglobulin V(D)J recombination,regulation of transcription, smooth muscle tissue development, positive regulation of epithelial cell proliferation, regulation of transcription factor activity, cardiac muscle cell differentiation, positive regulation of epithelial cell proliferation involved in lung morphogenesis	Intracellular, nucleus, transcription factor complex, cytoplasm	DNA binding, chromatin binding, double-stranded DNA binding, sequence-specific DNA binding transcription factor activity, sequence-specific DNA binding transcription factor activity, specific RNA polymerase II transcription factor activity, sequence-specific enhancer binding RNA polymerase II transcription factor activity, transcription factor binding, zinc ion binding, DNA bending activity, promoter binding, transcription activator activity, specific transcriptional repressor activity , protein homodimerization activity, sequence-specific DNA binding, metal ion binding, protein heterodimerization activity
AGAP1	11740492 _a_at	ArfGAP With GTPase Domain, Ankyrin Repeat And PH Domain 1	Transport, small GTPase mediated signal transduction, protein transport, regulation of ARF GTPase activity	Intracellular, cytoplasm	Nucleotide binding,GTPase activator activity, GTP binding, ARF GTPase activator activity, zinc ion binding, metal ion binding

PPAT	11731899 _s_at	Glutamine Phosphoribosylp yrophosphate	Purine nucleotide biosynthetic process, purine nucleotide biosynthetic process, glutamine metabolic process, lactation, metabolic process, purine base biosynthetic process, nucleoside metabolic process, purine ribonucleoside monophosphate biosynthetic process, organ regeneration, cellular response to insulin stimulus, protein homotetramerization, maternal process involved in female pregnancy	Soluble fraction	Amidophosphoribosyltransferase activity, transferase activity, transferase activity, transferring glycosyl groups, metal ion binding, iron-sulfur cluster binding, 4 iron, 4 sulfur cluster binding
NUB1	11762262 _a_at	Negative Regulator Of Ubiquitin-Like Proteins	Ubiquitin-dependent protein catabolic process, protein ubiquitination, positive regulation of proteasomal ubiquitin-dependent protein catabolic process, response to interferon-gamma, response to tumor necrosis factor	Nucleus	Protein binding
AKT2	11731470 _at	V-Akt Murine Thymoma Viral Oncogene Homolog 2	Positive regulation of protein phosphorylation, protein modification process, protein phosphorylation, response to osmotic stress, insulin receptor signaling pathway, positive regulation of signal transduction, positive regulation of gene expression, negative regulation of plasma membrane long-chain fatty acid transport, positive regulation of sodium ion transport, positive regulation of glucose metabolic process, response to muscle activity, positive regulation of cell migration, positive regulation of fatty acid beta-oxidation, response to insulin stimulus, cellular response to insulin stimulus inferred, cellular response to insulin stimulus, cellular response to hormone stimulus, negative regulation of RNA splicing, positive regulation of peptidyl-serine phosphorylation, negative regulation of apoptosis, negative regulation of caspase activity, protein kinase B signaling cascade, positive regulation of nitric oxide biosynthetic process, positive regulation of glycogen	Soluble fraction, nucleus, mitochondrion, microsome, cytosol, plasma membrane, lamellipodium, vesicle, insulin-responsive compartment	Binding, protein binding, beta-catenin binding, microtubule binding, protein kinase regulator activity , protein kinase binding,gamma-catenin binding, cadherin binding, microtubule plus-end binding

			biosynthetic process, positive regulation of transcription from RNA polymerase II promoter, positive regulation of glucose import, positive regulation of glucose import i, regulation of JNK cascade, positive regulation of positive chemotaxis, cellular response to chemical stimulus		
ABI2	11746842 _x_at	Abl-Interactor 2	Cellular component movement, cytoskeleton organization, learning or memory,actin polymerization or depolymerization,dendrite development, cell migration,peptidyl-tyrosine phosphorylation,camera-type eye development	Cytoplasm, cytosol, cytoskeleton,cell-cell adherens junction, lamellipodium, filopodium,dendrite,cell projection	DNA binding,cytoskeletal adaptor activity, SH3 domain binding, kinase binding, proline-rich region binding
FHL1	11753338 _x_at	Four and a half LIM domains 1	Multicellular organismal development, muscle organ development, organ morphogenesis, cell growth, cell differentiation	Nucleus, cytoplasm, cytosol	Protein binding, zinc ion binding, metal ion binding

APC	11757692_s_at	adenomatous polyposis coli	cytokinesis after mitosis,kidney development, hair follicle development, protein complex assembly, response to DNA damage stimulus, negative regulation of microtubule depolymerization, negative regulation of microtubule depolymerization, cell cycle arrest, mitotic metaphase/anaphase transition, mitotic cell cycle spindle assembly checkpoint, cell adhesion, establishment or maintenance of cell polarity, pattern specification process, axonogenesis, negative regulation of cell proliferation, negative regulation of cell proliferation, axis specification, anterior/posterior pattern formation, dorsal/ventral pattern formation, proximal/distal pattern formation, Wnt receptor signaling pathway, cell migration, stem cell maintenance, negative regulation of Wnt receptor signaling pathway, regulation of cell migration, positive regulation of cell migration, positive regulation of cell migration, regulation of epithelial cell differentiation, positive regulation of epithelial cell differentiation, positive regulation of microtubule polymerization, neuron projection development, positive regulation of pseudopodium assembly, T cell differentiation in thymus, somatic stem cell maintenance, negative regulation of odontogenesis, response to drug, regulation of apoptosis, positive regulation of apoptosis, negative regulation of apoptosis, negative regulation of MAPKKK cascade , skin development, regulation of cell differentiation, positive regulation of cell differentiation , regulation of osteoblast differentiation, positive regulation of protein catabolic process, positive regulation of protein catabolic process, negative regulation of cyclin-	kinetochore, nucleus, cytoplasm, centrosome, cytosol, microtubule, cytoplasmic microtubule, plasma membrane, adherens junction, cell-cell adherens junction, tight junction, lateral plasma membrane, cell junction, growth cone, beta-catenin destruction complex, cell projection membrane, axon part, APC-Axin-1-beta-catenin complex, cell projection, neuron projection	binding

			dependent protein kinase activity, positive regulation of cell adhesion, muscle cell homeostasis, thymus development, negative regulation of epithelial cell proliferation, regulation of nitrogen compound metabolic process, chromosome organization, regulation of cell cycle, positive regulation of cell division, regulation of attachment of spindle microtubules to kinetochore, regulation of attachment of spindle microtubules to kinetochore, regulation of attachment of spindle microtubules to kinetochore, retina development in camera-type eye, canonical Wnt receptor signaling pathway, negative regulation of epithelial cell proliferation involved in prostate gland development, tight junction assembly, negative regulation of canonical Wnt receptor signaling pathway		
